# Asymmetric construction of acyclic quaternary stereocenters via direct enantioselective additions of α-alkynyl ketones to allenamides

**DOI:** 10.1038/s41467-021-27028-7

**Published:** 2021-11-18

**Authors:** Jiawen Wang, Faqian He, Xiaoyu Yang

**Affiliations:** School of Physical Science and Technology, ShanghaiTech University, 201210 Shanghai, China

**Keywords:** Synthetic chemistry methodology, Asymmetric catalysis

## Abstract

Acyclic quaternary stereocenters are widely present in a series of biologically active natural products and pharmaceuticals. However, development of highly efficient asymmetric catalytic methods for the construction of these privileged motifs represents a longstanding challenge in organic synthesis. Herein, an efficient chiral phosphoric acid catalyzed direct asymmetric addition of α-alkynyl acyclic ketones with allenamides has been developed, furnishing the acyclic all-carbon quaternary stereocenters with excellent regioselectivities and high enantioselectivities. Extensive and detailed experimental mechanistic studies were performed to investigate the mechanism of this reaction. Despite a novel covalent allyl phosphate intermediate was found in these reactions, further studies indicated that a S_N_2-type mechanism with the ketone nucleophiles is not very possible. Instead, a more plausible mechanism involving the elimination of the allyl phosphate to give the α,β-unsaturated iminium intermediate, which underwent the asymmetric conjugate addition with the enol form of ketone nucleophiles under chiral anion catalysis, was proposed. In virtue of the fruitful functional groups bearing in the chiral products, the diverse derivatizations of the chiral products provided access to a wide array of chiral scaffolds with quaternary stereocenters.

## Abstract (web summary)

Acyclic quaternary stereocenters are widely present in a series of biologically active natural products and pharmaceuticals. Here the authors show an efficient chiral phosphoric acid catalyzed direct asymmetric addition of α-alkynyl acyclic ketones with allenamides to produce acyclic all-carbon quaternary stereocenters with excellent regioselectivities and high enantioselectivities:

## Introduction

**Fig. 1:**
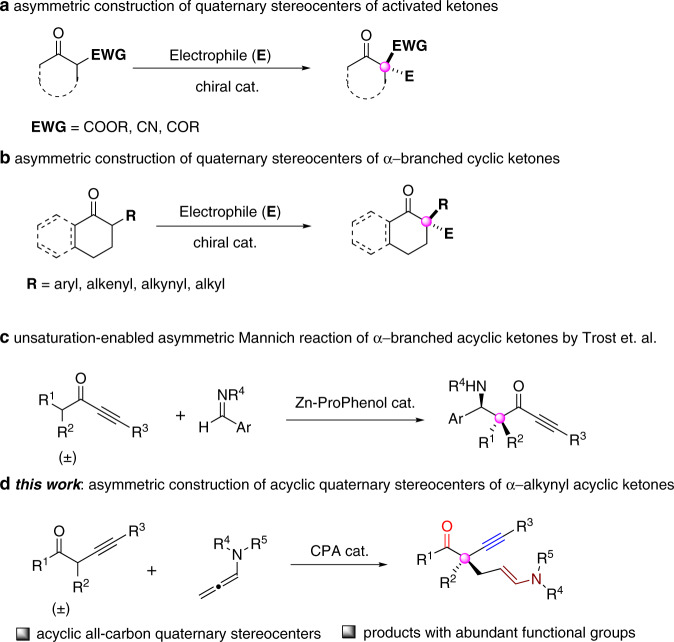
Asymmetric construction of all-carbon quaternary stereocenters via enantioselective functionalizations of α-branched ketones. **a** Asymmetric construction of quaternary stereocenters of activated ketones. **b** Asymmetric construction of quaternary stereocenters of α-branched cyclic ketones. **c** Unsaturation-enabled asymmetric Mannich reaction of α-branched acyclic ketones. **d** Asymmetric construction of acyclic quaternary stereocenters of α-alkynyl acyclic ketones.

All-carbon quaternary stereocenters, carbon centers with four distinct carbon substituents, are abundant in many biologically active small molecules and natural products^1^. However, the catalytic enantioselective construction of these important structural motifs remains synthetically challenging, due to their congested nature and required stereoselectivities in forming the new C–C bond. Despite these challenges, the last two decades have witnessed tremendous progress in the field of enantioselective construction of all-carbon quaternary stereocenters, and a number of elegant strategies have been developed^2–4^. To this end, the asymmetric functionalizations of α,α-disubstituted carbonyl compounds represent one of the most productive strategies. However, in contrast to the well-developed methods for asymmetric construction of quaternary stereocenters of α-branched aldehydes via enamine catalysis^5–9^ and others^10–14^, the analogous methods for α-branched ketones are more challenging, probably due to their low reactivity and poor regioselectivity issues. To date, existing methods are mostly limited to activated ketone substrates (i.e., ketones with strong α-electron-withdrawing groups), such as β-ketoesters^15–23^ and others^24–26^ (Fig. 1a). For α-branched ketones without these strong activation groups, their relatively low reactivity is a major obstacle in these reactions. Recently, several ingenious methods have been developed through direct asymmetric functionalizations of α-branched cyclic ketones, such as Michael additions^27–31^, allylic alkylation^32–34^, arylation^35–38^, and others^39,40^ (Fig. 1b). Notably, the chiral phosphoric acids (CPA) catalyzed regio- and enantioselective functionalizations of α-branched cyclic ketones have become a powerful strategy for the construction of α-quaternary stereocenters for cyclic ketones^28,32,41–46^. In these reactions, the formation of thermodynamic favored more-substituted enol intermediate under acid-catalysis well addressed the issue of regioselectivity.

The direct asymmetric functionalizations of α-branched acyclic ketones with less-stabilized enol/enolate intermediates is still an unconquered challenge^47–50^, since the extra requirement for the formation of geometrically defined enol/enolate intermediate in the ketone activation step. To overcome this hurdle, some elegant indirect methods employing stereodefined acyclic enolate as nucleophiles have been developed via asymmetric alkylation^51^ and allylic alkylations^52–54^. To the best of our knowledge, only one example of direct asymmetric functionalization of acyclic α-branched ketones for the construction of quaternary stereocenter has been disclosed by Trost and co-workers, who developed the unsaturation-enabled asymmetric Mannich reactions using Zn-Prophenol catalysis^55^ (Fig. 1c). The unsaturation introduced in the ketone substrates was believed to play two roles: (1) serving as a blocking group to overcome the issue of regioselectivity; (2) modifying the activity of substrates by adjusting the p*K*a of the α-protons. With our continuous interest in CPA-catalyzed asymmetric addition reactions^56–59^, we explored the possibility of using acyclic α-branched ketones as the nucleophiles in these reactions.

Herein, we disclose an efficient method for the construction of acyclic all-carbon quaternary stereocenters via enantioselective additions of α-alkynyl branched ketones with allenamides^60–64^, which gives access to ketones bearing α-quaternary stereocenters with broad substrate scope and high enantioselectivities (Fig. 1d).

## Results

### Reaction condition optimizations

Since the diverse transformations of the alkynyl groups and their weak ability to activate the α-position of carbonyl compounds due to the high *s* character of alkynyl groups^65,66^, we started our study by employing racemic α-alkynyl ketone **1a** as the model substrate. Encouragingly, when the reaction between **1a** and allenamide **2a** was investigated under the catalysis of CPA **A1** (10 mol%) in CCl_4_ at ambient temperature, the desired product **3a** bearing an all-carbon quaternary stereocenter was exclusively generated in 61% yield with 71:29 enantiomeric ratio (er), without detecting the potential α’- or γ-regioisomers (Table 1, entry 1). Next, a series of BINOL-derived CPA catalysts were screened in this reaction (entries 2–8), which indicated that the sterically bulky CPA catalyst **A7** (TRIP) to be the optimal one (70%, 95:5 er, entry 7). Furthermore, switching the chiral scaffold of TRIP from BINOL-type to H8-BINOL-type led to further improved enantioselectivity (75%, 97.5:2.5 er, entry 9). In addition, 4 Å molecular sieves (MS) was found to be effective in promoting the reaction with higher yield (95%, entry 10), probably because of the inhibition of hydrolysis of allenamide under these conditions. A range of solvents was also examined in this reaction (entries 11–14). Interestingly, generally high enantioselectivities were observed with these solvents; however, none of them could provide superior results than CCl_4_.

**Table 1.** Optimizations of the reaction conditions^a^.


Entry	Cat.	Solvents	Yield (%)^b^	Er^c^
1	**A1**	CCl_4_	61	71:29
2	**A2**	CCl_4_	44	85:15
3	**A3**	CCl_4_	47	72:28
4	**A4**	CCl_4_	65	94.5:5.5
5	**A5**	CCl_4_	42	92:8
6	**A6**	CCl_4_	65	95:5
7	**A7**	CCl_4_	70	95:5
8	**A8**	CCl_4_	41	91:9
9	**A9**	CCl_4_	75	97.2:2.5
10^d^	**A9**	CCl_4_	89	97.5:2.5
11^d^	**A9**	Toluene	81	96.5:3.5
12^d^	**A9**	DCM	47	90:10
13^d^	**A9**	Et_2_O	71	95:5
14^d^	**A9**	THF	56	93.5:6.5

*TIPS* triisopropylsilyl, *Piv* pivaloyl, *THF* tetrahydrofuran.

^a^Reactions were performed with **1a** (0.1 mmol), **2a** (0.11 mmol), CPA (0.01 mmol) in solvents (1 mL) at room temperature.

^b^Isolated yields.

^c^Er values were determined by HPLC analysis on a chiral stationary phase.

^d^Activated 4 Å MS (100 mg) was added.

### Substrates scope investigation

**Fig. 2:**
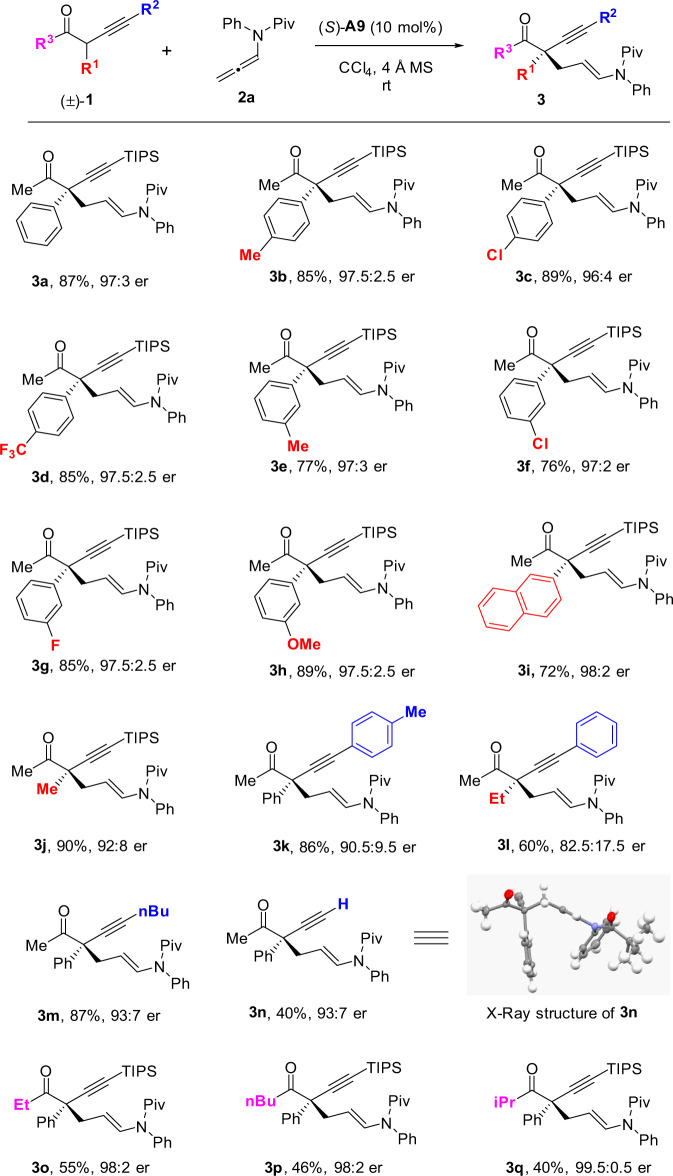
Substrate scope of α-alkynyl ketones. Reactions were performed with **1** (0.2 mmol), **2a** (0.22 mmol), CPA (*S*)-**A9** (0.02 mmol), activated 4 Å MS (200 mg) in CCl_4_ (2 mL) at room temperature. Isolated yields. Er values were determined by HPLC analysis on a chiral stationary phase.

With the optimal conditions in hand, we set out to explore the scope of α-alkynyl ketones for the asymmetric construction of acyclic quaternary stereocenters (Fig. 2). A range of substituted phenyl groups (with various substituents at the *para*- and *meta*-positions) at the α-position of ketones were examined, which were well tolerated with the optimal reaction conditions (**3b**–**3h**), as well as a 2-naphthyl group (**3i**). Moreover, switching the α-substitution from an aryl group to an alkyl group was also amenable, providing the addition product in high yield and enantioselectivity (**3j**). Subsequently, the scope of the alkynyl groups was also studied, which suggested that both the arylacetylene- and alkylacetylene-substituted acyclic ketones were compatible with the optimal conditions, albeit generating products with slightly diminished enantioselectivities (**3k**–**3m**). Additionally, using an α-acetylene-substituted ketone as substrate under the standard conditions afforded the desired product with 92.5:7.5 er and moderate yield (**3n**). The absolute configuration of these addition products was assigned as (*S*) by analogy to the structure of **3n**, which was unambiguously determined by X-ray crystallography. Finally, the variants on the other side of the ketone substrates were also investigated. Replacing the Me group with other alkyl groups (e.g., Et, *n*-Bu, and *i*Pr group) was feasible, which afforded the addition products with excellent stereoselectivities, albeit in moderate yields due to their relatively low reactivities (**3o**–**3q**).

**Fig. 3:**
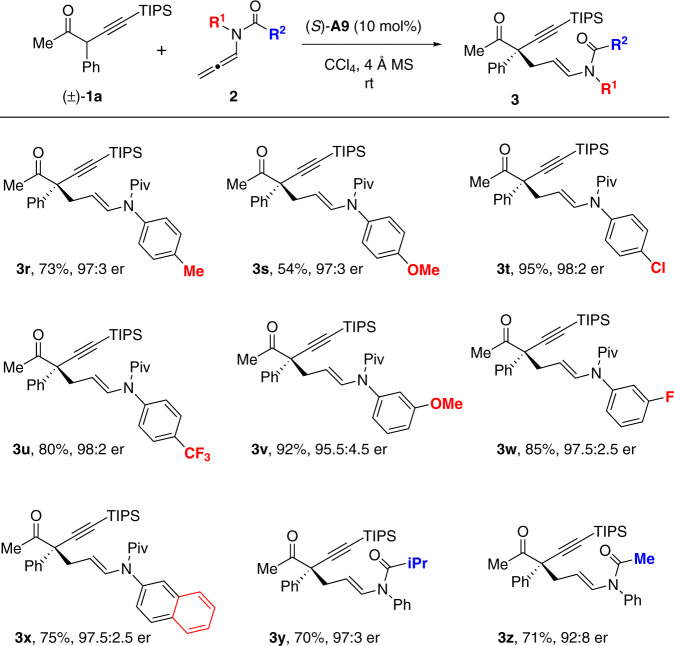
Substrate scope of allenamides. Reactions were performed with **1a** (0.2 mmol), **2** (0.22 mmol), CPA (*S*)-**A9** (0.02 mmol), activated 4 Å MS (200 mg) in CCl_4_ (2 mL) at room temperature. Isolated yields. Er values were determined by HPLC analysis on a chiral stationary phase.

With the excellent compatibility of α-alkynyl ketone substrates, we turned our attention to the scope of allenamides (Fig. 3). A series of substituted *N*-aryl groups were examined in this reaction, which were feasible variants under the standard conditions, regardless of the electronic nature and substitution sites of these substituents (**3r**–**3x**). Next, the scope of the amide moieties was also studied. Switching the alkylacyl group from Piv group to the less sterically demanding groups were amenable in these reactions, and the desired products could be uniformly obtained with high enantioselectivities (**3y** and **3z**).

### Mechanistic studies

**Fig. 4:**
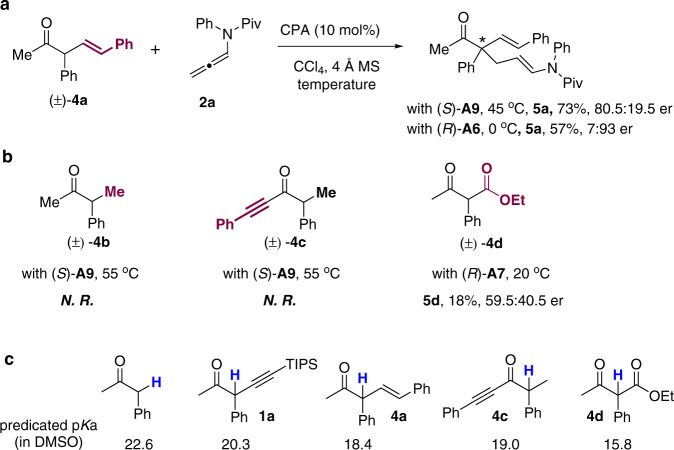
Control experiments. **a** Reaction of α-alkenyl ketone substrate. **b** Reactions of α-alkyl ketone **4b**, α-branched ynone **4c** and β-ketoester **4d** in this reaction. **c** Predicted p*K*a values of ketone substrates.

In order to probe the effect of the α-alkynyl groups in these reactions, a number of α-substituted acyclic ketones were prepared and investigated in this reaction (Fig. 4). Although relatively low reactivity of the α-styrenyl-substituted ketone **4a** was observed under the standard conditions (with CPA (*S*)-**A9**), raising the reaction temperature to 45 °C provided the acyclic quaternary stereocenter containing product **5a** in 73% yield with 80.5:19.5 er. Brief optimizations of the CPA catalyst indicated that high enantioselectivity could be afforded in the presence of CPA (*R*)-**A6** at 0 °C, which generated **5a** in 57% yield with 7:93 er. These results indicated that this reaction is not only feasible for α-alkynyl-substituted ketones, but could also be extended to broader ketone scope. However, switching the alkynyl group to alkyl group (**4b**) or using Trost’s α-branched ynone **4c**^55^ as substrates could not provide the desired adducts, even at elevated reaction temperature (55 °C, Fig. 4b). Surprisingly, the activated ketone substrate (e.g., β-ketoester **4d**) also exhibited low reactivity under these conditions, which only gave the desired addition product **5d** in 18% yield, with poor enantioselectivity (59.5:40.5 er) as well. These results together with the predicted p*K*a values^67^ (obtained from http://pka.luoszgroup.com/) of these acyclic ketone substrates indicated that although the α-alkynyl substitutions could increase the acidity of the α-protons, the reactivities of these ketones in this reaction were not positively correlated with the p*K*a values of the α-protons (Fig. 4c). We believe that both the electronic effect and the steric effect of the α-substituents play important roles in modulating the reactivities of these α-branched ketones in this reaction.

**Fig. 5:**
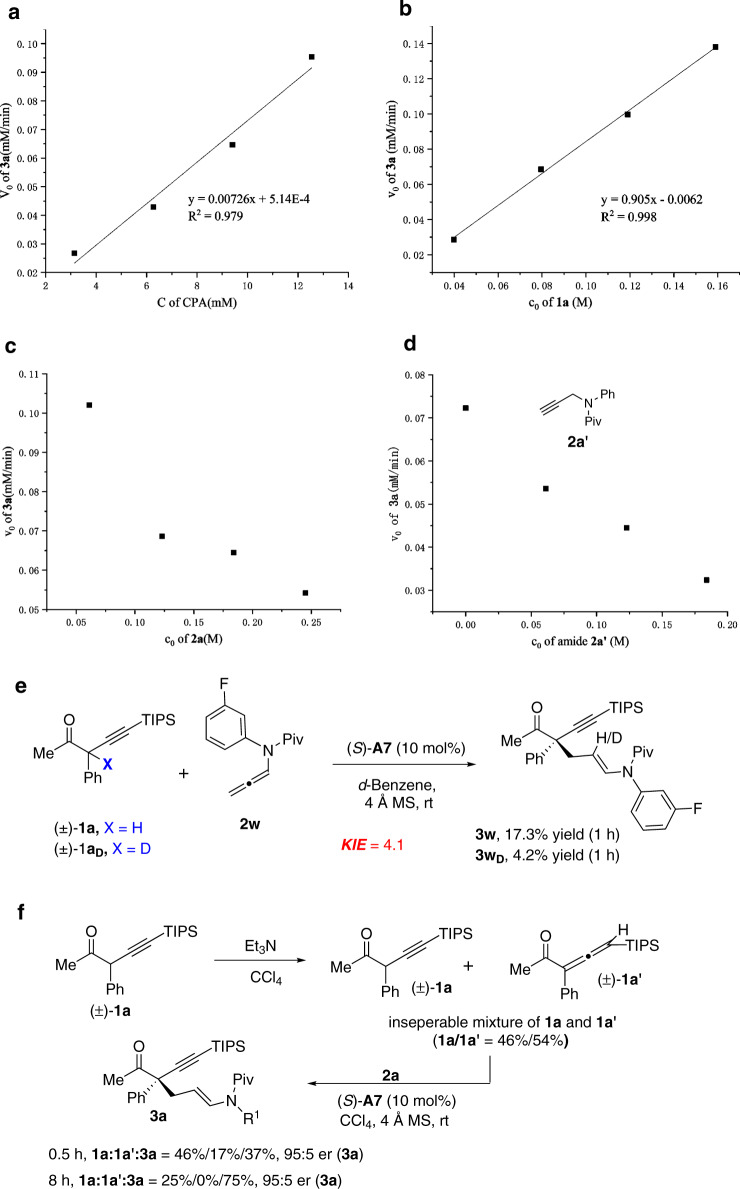
Kinetic experiments. **a** Relationship between the initial reaction rate and concentration of the CPA catalyst **A7. b** Relationship between the initial reaction rate and concentration of ketone **1a. c** Relationship between the initial reaction rate and concentration of the allenamide **2a. d** Relationship between the initial reaction rate and concentration of the amide additive **2a’. e** KIE experiment of the α-H of ketone **1a. f** Isomerization of **1a** into allenyl ketone **1a’** and its reaction with allenamide **2a** under the standard conditions.

The kinetic profile experiments were conducted on the model reaction to study the mechanism of these reactions. A linear relationship between the initial reaction rate and the concentration of CPA **A7** and ketone **1a** was observed, respectively, which suggested a first-order dependence on CPA catalyst and ketone substrate (Fig. 5a, b). In addition, a negative correlation between the initial reaction rate and concentration of allenamide **2a** and an amide additive **2a’** was found respectively, which suggested that the allenamide is probably not involved in the rate-determining step and the basicity of the amide group may inhibit this acid-catalyzed reaction (Fig. 5c, d). Moreover, a parallel kinetic isotopic effect (KIE) experiment of the α-H of the ketone substrate **1a** was performed and gave a KIE value of 4.1, which suggested that the cleavage of this α-C–H bond is probably involved in the rate-determining step (Fig. 5e). Interestingly, treatment of **1a** with Et_3_N (0.2 equiv.) led to the formation of an inseparable mixture of **1a** and allenyl ketones **1a’** (**1a**:**1a’** = 0.46:0.54), in which **1a** and **1a’** were in equilibrium (Fig. 5f). Monitoring the reaction of this **1a**/**1a’** mixture with allenamide **2a** under the standard conditions using ^1^H NMR suggested that the allenyl ketone **1a’** was actually more reactive in this reaction, as indicated by the conversion results at 0.5 h and 8 h. Moreover, the enantioselectivities at the early stage (0.5 h) and late stage (8 h) of this reaction retained the same value, which indicated that both reactions of **1a** and **1a’** could go through the same reaction intermediate, namely the enol form of the ketone. The different reaction rates of **1a** and **1a’** also indicated that the ability to form the active enol intermediate may determine the reaction rate of this reaction.

**Fig. 6:**
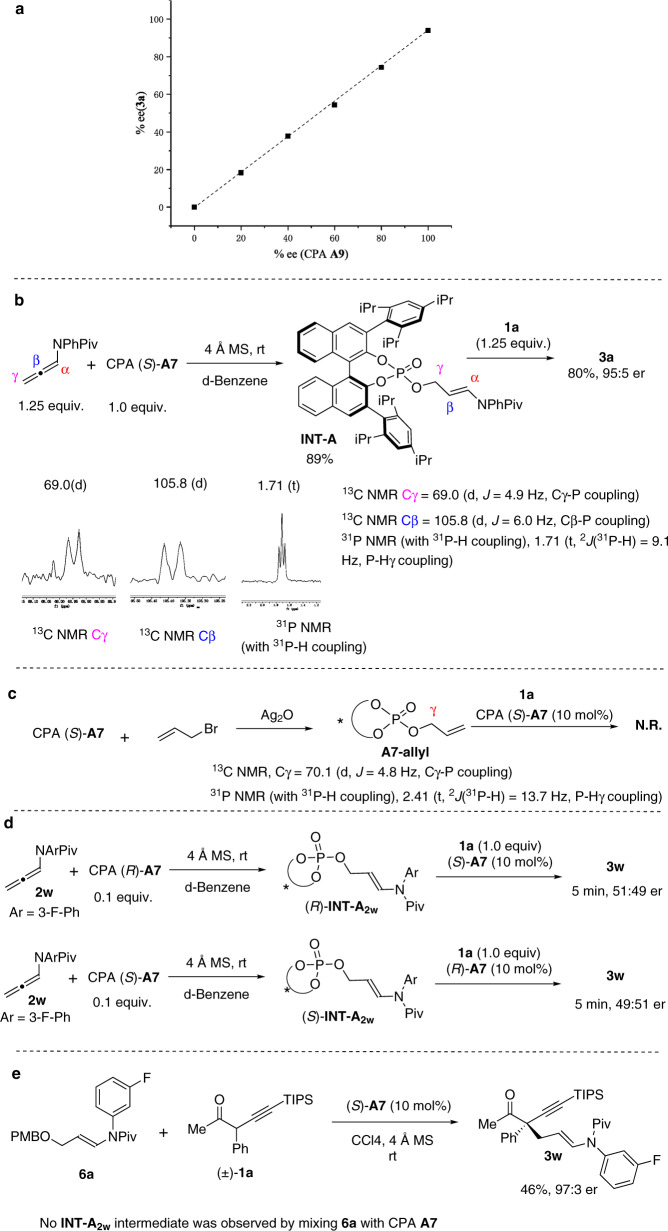
Investigation of the asymmetric induction mechanism. **a** Nonlinear effect study. **b** Detection and characterization of the reaction intermediate. **c** Preparation of allyl phosphate of CPA **A7** and its reaction with ketone **1a** under the standard conditions. **d** Cross experiment of **INT-A**_**2w**_. **e** Asymmetric reaction of allyl benzyl ether **6a** with ketone **1a** under the standard conditions.

In order to gain more details of the asymmetric induction model^68^, the nonlinear effect study was first performed. A highly linear relationship between the ee of product **3a** and the ee of CPA **A9** was observed, which indicated the presence of only one chiral catalyst (intermediate) in the enantio-determining step (Fig. 6a). We also studied the potential reaction intermediate by mixing allenamide **2a** with stoichiometric amount of CPA **A7** in D6-benzene, which readily afforded a mixture of acrolein (the hydrolysis product of **2a**) and an unknown product, as detected by ^1^H NMR (Fig. 6b). However, an attempt to isolate this compound by silica gel chromatography resulted in no success. Nevertheless, HRMS analysis of the mixture implied the formation of an adduct between allenamide and CPA. A set of NMR spectroscopies (including ^1^H NMR, ^13^C NMR, 2D NMR, ^31^P NMR) were directly performed on this mixture, which suggested the formation of allyl phosphate adduct **INT-A** (see Supplementary Information for details). The characteristic NMR signals include the doublet ^13^C NMR peaks of Cγ and Cβ through the coupling with the phosphorus atom of phosphate moiety and the triplet ^31^P NMR peak via the coupling with the two H atoms of the Cγ-methylene moiety. Furthermore, the addition of α-alkynyl ketone **1a** into this mixture provided the same addition product **3a** in 80% yield with 95:5 er after 20 h. In addition, another allyl phosphate intermediate (**INT-A**_**2w**_) formed between CPA **A7** and the *N*-fluorobenzene-substituted allenamide **2w** could be clearly observed by ^19^F NMR at −110.9 ppm, which facilitated the monitoring of this reaction. Interestingly, the **INT-A**_**2w**_ could be detected at the beginning of this reaction, however, which disappeared after 2 h while the reaction was still proceeding (see Supplementary Information for details).

Although the covalent phosphate **INT-A** was unambiguously assigned and it could undergo the addition reaction with α-alkynyl ketone **1a** to give the enantioenriched addition product **3a**, there are still two plausible reaction pathways of **INT-A**. The first one is that the enol form of the ketone substrate would directly attack the **INT-A** through a S_N_2 substitution reaction. While the second one is that the **INT-A** would go through the elimination reaction to give the α,β-unsaturated iminium intermediate pairing with the chiral phosphate anion, which would receive the conjugate addition of the ketone to give the corresponding addition product. To figure out which reaction pathway **INT-A** is taking part in, some control experiments were performed. First, an allyl phosphate of CPA **A7** (**A7-allyl**) was prepared, whose characteristic NMR signals are well matched with that of **INT-A**, again confirming the structure of **INT-A** (Fig. 6c). However, **A7-allyl** is rather stable and could be readily purified by silica gel column chromatography. Moreover, the subjection of **A7-allyl** into the standard conditions with ketone **1a** did not provide the expected ketone α-allylation product. Second, a cross experiment was performed (Fig. 6d). Mixing allenamide **2w** with (*R*)-CPA **A7** gave the mixture containing (*R*)-**INT-A**_**2w**_, which was followed by adding a mixture of ketone **1a** and CPA (*S*)-**A7**. On the other hand, another experiment was conducted by mixing **2w** with (*S*)-**A7** first to generate (*S*)-**INT-A**_**2w**_, which was followed by adding the mixture of ketone **1a** and CPA (*R*)-**A7**. We anticipated that if the covalent **INT-A**_**2w**_ would directly participate in the S_N_2 reaction with the enol form of the ketone, these two reactions would provide products with distinct er values at the early stage of the reactions, since the enantioselectivities would be mainly controlled by the corresponding chiral covalent **INT-A**_**2w**_. However, both reactions afforded products **3w** in almost racemic version at the early stage of the reactions (at 5 min). Furthermore, allyl benzyl ether **6a** was prepared, which was anticipated to undergo the facile E1cb-type elimination to give the α,β-unsaturated iminium under acidic conditions. The subjection of the allyl benzyl ether **6a** into the standard conditions with ketone **1a** afforded the same addition product **3w** in 46% yield with 97:3 er, which suggested that **6a** probably underwent the same reaction intermediate as allenamide **2w** (Fig. 6e). However, it is worth mentioning that no covalent intermediate **INT-A**_**2w**_ was observed in ^31^P NMR and ^19^F NMR by mixing **6a** with CPA (*S*)-**A7** in D6-benzene.

**Fig. 7:**
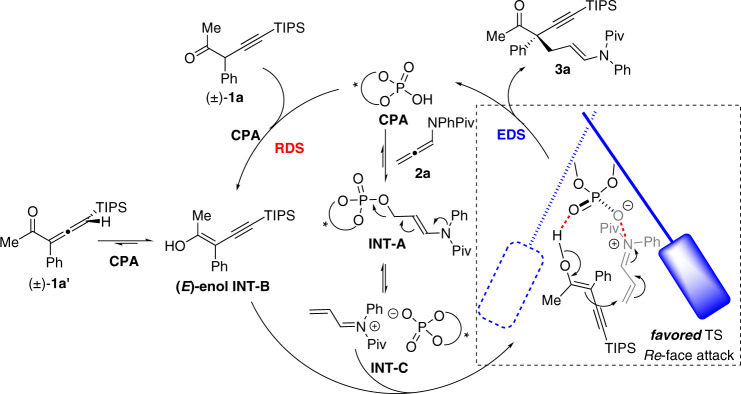
Proposed reaction mechanism and asymmetric transition state. CPA catalyzed enolization of ketone as the rate-determining step and the conjugate addition with the α,β-unsaturated iminium as the enantio-determining step.

Based on these experimental results, a plausible reaction mechanism for this reaction was proposed, in which the CPA catalyst played two roles (Fig. 7). First, CPA catalyzed the isomerization of ketone **1a** to generate the *E*-configurated enol intermediate **INT-B** (the less sterically hindered enol intermediate), which is the rate-determining step (RDS) of this reaction. The isomerization of allenyl ketone **1a’** to **INT-B** was also feasible and more efficient, which may be available to develop new asymmetric reactions^65^. The second role of CPA is the facile addition with the allenamide **2a** to form the allyl phosphate adduct **INT-A**, however, which is not the real active intermediate and not involved in the key asymmetric transition state. We anticipated that the covalent phosphate **INT-A** would undergo the facile E1cb-type elimination to give the α,β-unsaturated iminium **INT-C**^69^, paired with the corresponding chiral phosphate anion, which was believed to be the real active intermediate. The asymmetric conjugate addition of the enol **INT-B** with this α,β-unsaturated iminium **INT-C** under the guidance of chiral phosphate anion was proposed to be the enantio-determining step (EDS) of this reaction, and the hydrogen-bonding between the enol −OH group and the P = O moiety of phosphate **INT-C** was believed to be crucial. Consequently, the *Re*-face attack of the enol intermediate **INT-B** to the α,β-unsaturated iminium under the dual-activation of the phosphate anion of CPA (*S*)-**A9** afforded the enantioenriched addition product **3a** with *S*-configuration.

### Derivatizations of chiral products

**Fig. 8:**
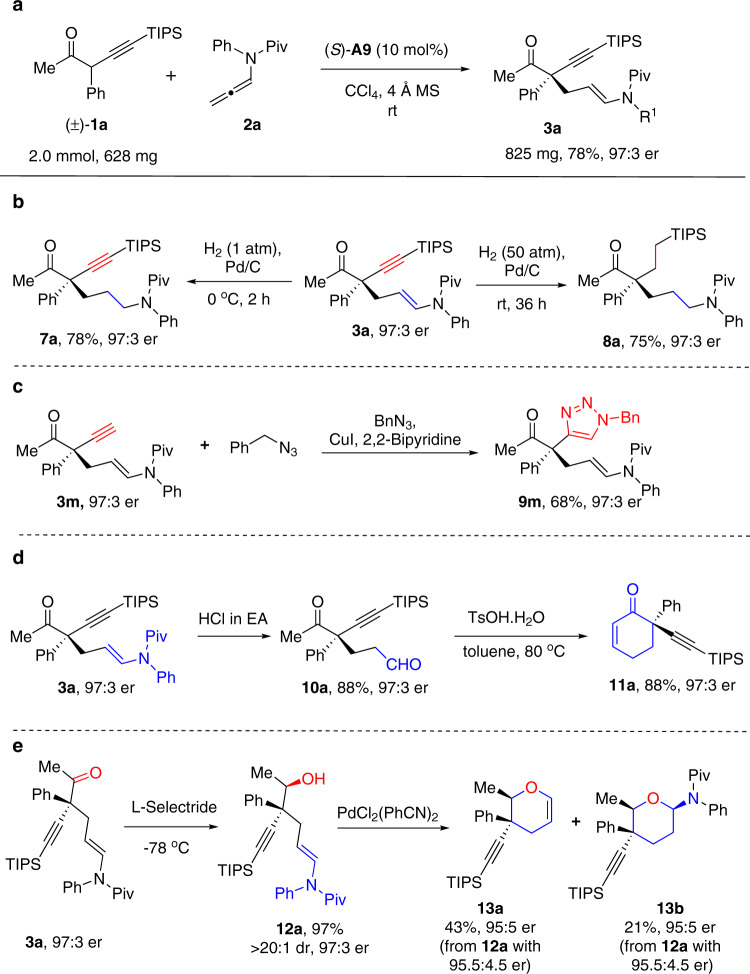
Large-scale reaction and derivatizations of the chiral products. **a** Gram-scale reaction. **b** Hydrogenation of **3a. c** Cycloaddition of **3a** with organic azide. **d** Hydrolysis and aldol reaction of **3a**. **e** Reduction and cyclization of **3a**.

A large-scale asymmetric reaction of **1a** (2.0 mmol) with allenamide **2a** was performed under the optimal conditions to showcase the practicability of this method, which readily provided the desired addition product **3a** in 78% yield with 97:3 er (Fig. 8a). To demonstrate the synthetic utilities of these chiral products bearing fruitful functional groups, a range of transformations were studied. Hydrogenation of **3a** using Pd/C as catalyst under 1 atm of H_2_ at 0 °C provided the enamide reduced product **7a**; while performing the hydrogenation under 50 atm of H_2_ provided both enamide and alkyne hydrogenated product **8a** (Fig. 8b). Cu-catalyzed cycloaddition between the acetylene motif of **3** **m** and benzyl azide proceeded smoothly to give the triazole product **9** **m** in 68% yield with 97:3 er (Fig. 8c). Treatment of **3a** with HCl/EA solution facilely hydrolyzed the enamide moiety, generating the 5-keto aldehyde **10a** in high yield, which upon treatment with TsOH at 80 °C provided the aldol reaction product cyclohexenone **11a** in 88% yield with retained optical purity (Fig. 8d). The ketone moiety in product **3a** could be stereoselectively reduced by l-selectride at −78 °C to give the alcohol **12a** in 97% yield with >20:1 dr (Fig. 8e). Further cyclization of **12a** under the catalysis of PdCl_2_(PhCN)_2_ provided access to dihydropyran derivative **13a** in 43% yield and cyclic aminal **13b** in 21% yield, without erosion of enantiomeric purity. The relative configurations of the products were assigned by NOE analysis of **13a** and **13b**.

## Discussion

In conclusion, we have disclosed a practical method for asymmetric construction of acyclic all-carbon quaternary stereocenters via direct enantioselective additions of α-alkynyl ketones to allenamides enabled by chiral phosphoric acid catalysis. A broad range of α-substitutions, alkynyl groups, and substituents on allenamide were well compatible within these reactions, generating acyclic ketones bearing α-quaternary stereocenters with high enantioselectivities. Detailed mechanistic studies were performed to elucidate the mechanism of these reactions, and a CPA catalyzed enol formation step was proposed as the rate-determining step. Although a novel covalent allyl phosphate **INT-A** was well identified by various spectroscopic methods in these reactions, more mechanistic studies suggested that it was probably not involved in the crucial asymmetric transition state. A more plausible mechanism involving the enantioselective addition of the enol form of ketones with the α,β-unsaturated iminium under the guidance of chiral phosphate anion as the enantio-determining step was proposed. The abundance of various functional groups within the chiral products facilitated the diverse derivatizations of the chiral products, demonstrating the synthetic utilities of these methods.

## Methods

To a dried sealed tube was added 4 Å molecular sieves (200 mg), allenamide **2** (0.22 mmol) and (*S*)-**A9** (15.2 mg 0.02 mmol) sequentially, which was followed by adding a solution of ketone **1** (0.2 mmol) in CCl_4_ (2 mL). After stirring at room temperature for 20 h, the mixture was filtered through Celite and the filtrate was concentrated under vacuum to give a residue, which was purified by column chromatography on silica gel (petroleum ether:EtOAc, 30:1 ~10:1) to give the products **3**.

## Supplementary information

Supplementary Information

## Data Availability

The authors declare that the data supporting the findings of this study are available within the article and Supplementary Information file, or from the corresponding author upon request. The X-ray crystallographic coordinates for structures reported in this study have been deposited at the Cambridge Crystallographic Data Centre (CCDC), under deposition numbers CCDC 2047788 (**3n**). These data can be obtained free of charge from The Cambridge Crystallographic Data Centre via www.ccdc.cam.ac.uk/data_request/cif.
