# Supplementary material for: Asymmetric construction of acyclic quaternary stereocenters via direct enantioselective additions of α-alkynyl ketones to allenamides
Source: Nat Commun. 2021 Nov 18;12:6700. doi: 10.1038/s41467-021-27028-7 (PMC8602376; doi:10.1038/s41467-021-27028-7)
Supplement: Supplementary file 1 — Supplementary Information [file 41467_2021_27028_MOESM1_ESM.pdf]

Supplementary Information

Asymmetric Construction of Acyclic Quaternary Stereocenters via Direct Enantioselective Additions of α-Alkynyl Ketones to Allenamides

Wang et al.

Table of contents

Supplementary Methods.....3
1. General Information.....3
2. Synthesis of ketone substrates.....4
3. Synthesis of allenamide substrates.....10
4. Asymmetric synthesis of chiral products.....13
5. Mechanistic studies.....24
6. Large scale asymmetric synthesis of 3a.....53
7. Transformations of chiral products.....53
8. X-ray structures.....59
9. HPLC traces.....61
10. NMR spectrums.....98
Suppelmentary References.....168

Supplementary Methods

1. General Information

Unless specified otherwise, all of the commercial reagents were used directly without further purification. Chloroform was dried by activated 5Å molecular sieves, and dichloromethane, toluene, ether, THF were purified by passage through an activated alumina column under nitrogen. Thin-layer chromatography (TLC) analysis of reaction systems was performed using Huanghai silica gel HSGF254 TLC plates, and visualized under UV or by staining with ceric ammonium molybdate. Flash column chromatography was carried out on Huanghai Silica Gel HHGJ-300, 300-400 mesh. Nuclear magnetic resonance (NMR) spectra were recorded using a Bruker Avance III HD spectrometer (FT, 500 MHz or 400 MHz for ^{1}H, 126 MHz or 101 MHz for ^{13}C, 471 MHz for ^{19}F). Data for ^{1}H NMR were reported as follows: chemical shift (δ ppm downfield from tetramethylsilane and referenced to residual solvent peaks), multiplicity (s = singlet, d = doublet, t = triplet, q = quartet, m = multiplet, br = broad resonance), integration, coupling constant (Hz). Data for ^{13}C NMR were reported in terms of chemical shift. FT-IR spectra were recorded on a Bruker VERTEX 70 Spectrometer and are reported in terms of frequency of absorption (cm^{-1}). Mass spectral data were obtained from a Thermo ultimate 3000 Ultra Performance Liquid Chromatography associated with Q Exactive Focus mass spectrometer in electrospray ionization (ESI^{+}) mode or atmospheric pressure chemical ionization (APCI^{+}) mode. Optical rotation was measured by an Autopol V Plus/VI digital polarimeter. X-ray structure analysis was performed using a Bruker D8 Venture X-ray single crystal diffractometer. Enantiomeric excess was determined on an Agilent 1260 Chiral HPLC using IA, IB, IC, ID and IG columns.

2. Synthesis of ketone substrates

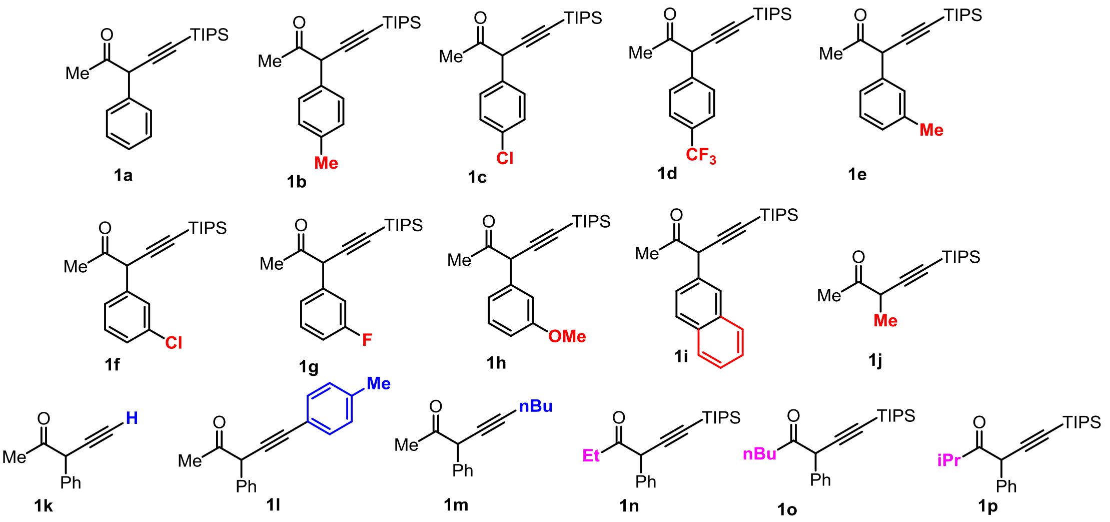

General procedure for the synthesis of ketone 1:

Step 1 and 2:

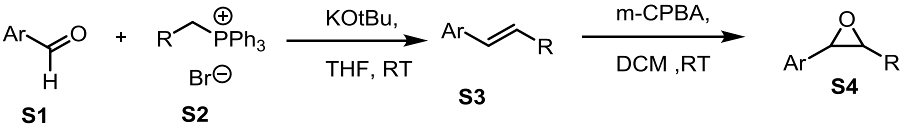

The epoxides **S4** were prepared form aldehyde **S1** and phosphonium bromide **S2** in two steps according to literature[1]. For the synthesis of ketone **1j**, the corresponding epoxide (cis-2,3-epoxybutane) is commercial available.

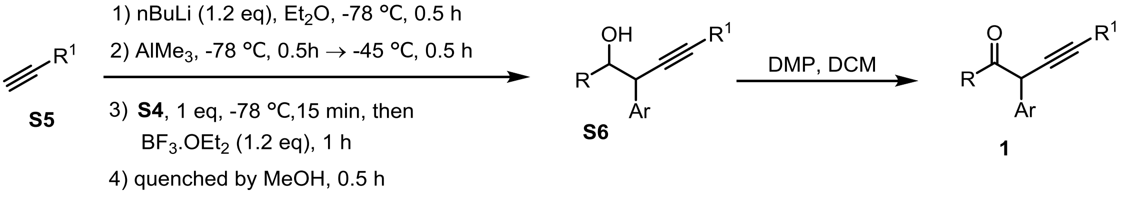

Step 3: The procedure for addition of acetylenes **S5** with epoxide **S4** was adopted from the literature[2]. To a solution of acetylene **S5** (3.6 mmol) in Et_2O (10 mL) at –78 ℃ was added n-BuLi (2.5 M in hexane, 1.5 mL, 3.6 mmol) over 2 min. After stirring at –78 ℃ for additional 30 min, trimethylalumina (2.0 M in toluene, 1.8 mL, 3.6 mmol) was added and the mixture was stirred at –78 ℃ for 30 min. The mixture was warmed to –45 ℃ for 30 min and cooled to –78 ℃ again. Epoxides **S4** (3 mmol) was added and stirred for 15 min before $BF_3{\cdot}Et_2O$ (3.6 mmol) was added. The mixture was stirred at –78 ℃ for 2 h, whereupon methanol (5 mL) was added. The reaction was allowed to warm to 0 ℃ over 15 min before saturated aqueous NH_4Cl (5 mL) was

added. After stirring at room temperature for additional 30 min, the content was diluted with NH_4Cl (aq), extracted with EtOAc, dried over Na_2SO4, filtered, and concentrated. The residue was purified by column chromatography on silica gel (petroleum ether:EtOAc = 40:1) to give the alcohol **S6** as clear oil.

Step 4: To a solution of alcohol **S6** in DCM (20 mL) was added Dess-Martin periodinane (2.0 eq). After stirring for 2 h at room temperature, the mixture was diluted with DCM, washed by water and extracted with DCM for 3 times. The combined organic layer was dried over Na_2SO_4, filtered, and concentrated to give a residue. After adding hexane (5 mL) to this residue, the mixture was filtered through Celite and the filtrate was concentrated to give the ketone **1** as colorless oil without further purification.

3-phenyl-5-(triisopropylsilyl)pent-4-yn-2-one (**1a**)

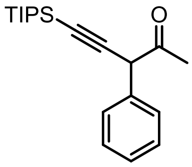

^{1}H NMR (400 MHz, Chloroform-*d*) δ 7.5 (d, *J* = 7.4 Hz, 2H), 7.4 (t, *J* = 7.3 Hz, 2H), 7.3 (t, *J* = 7.2 Hz, 1H), 4.6 (s, 1H), 2.2 (s, 3H), 1.1 (s, 21H). ^{13}C NMR (101 MHz, $CDCl_3$) δ 202.7, 134.6, 128.8, 127.9, 102.8, 89.0, 53.9, 25.7, 18.7, 11.3. HRMS-ESI (m/z) calculated for $C_{20}H_{31}OSi^+$ $[M+H]^+$: 315.2139; found: 315.2135.

3-(p-tolyl)-5-(triisopropylsilyl)pent-4-yn-2-one (**1b**)

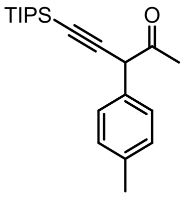

^{1}H NMR (500 MHz, Chloroform-*d*) δ 7.28 (d, *J* = 7.8 Hz, 2H), 7.10 (d, *J* = 7.8 Hz, 2H), 4.50 (s, 1H), 2.27 (s, 3H), 2.15 (s, 3H), 1.04 (s, 21H). ^{13}C NMR (126 MHz, $CDCl_3$) δ 201.9, 136.6, 130.6, 128.5, 126.7, 102.0, 87.7, 52.5, 24.6, 20.1, 17.6, 10.3. HRMS-ESI (m/z) calculated for $C_{21}H_{33}OSi^+$ $[M+H]^+$: 329.2295; found: 329.2289.

3-(4-chlorophenyl)-5-(triisopropylsilyl)pent-4-yn-2-one (**1c**)

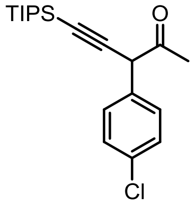

^{1}H NMR (400 MHz, Chloroform-*d*) δ 7.4 (m, 4H), 4.6 (s, 1H), 2.3 (s, 3H), 1.1 (s, 21H). ^{13}C NMR (101 MHz, $CDCl_3$) δ 202.2, 133.9, 133.2, 129.3, 129.0, 102.3, 89.4, 53.1, 25.9, 18.6, 11.3. HRMS-ESI (m/z) calculated for $C_{20}H_{30}ClOSi^+$ $[M+H]^+$: 349.1749; found: 349.1744.

3-(4-(trifluoromethyl)phenyl)-5-(triisopropylsilyl)pent-4-yn-2-one (**1d**)

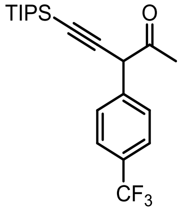

1d was obtained as a mixture with 11% allene isomer. ^{1}H NMR (500 MHz, Chloroform-*d*) δ 7.6 (d, *J* = 8.1 Hz, 2H), 7.5 (d, *J* = 9.7 Hz, 2H), 4.6 (s, 1H), 2.2 (s, 3H), 1.0 (m, 21H). ^{13}C NMR (126 MHz, Chloroform-*d*) δ 201.9, 138.8, 129.0, 128.5, 125.9 (q, *J* = 3.7 Hz), 124.1 (q, *J* = 263.4 Hz), 101.9, 90.0, 53.6, 26.3, 18.8, 11.4. HRMS-ESI (m/z) calculated for $C_{21}H_{30}F_3OSi^+$: 383.2013; found: 383.2006.

3-(m-tolyl)-5-(triisopropylsilyl)pent-4-yn-2-one (**1e**)

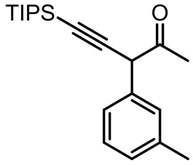

^{1}H NMR (500 MHz, Chloroform-*d*) δ 7.23 (s, 1H), 7.19 (s, 2H), 7.07 – 7.03 (m, 1H), 4.50 (s, 1H), 2.28 (s, 3H), 2.16 (s, 3H), 1.05 (s, 21H). ^{13}C NMR (126 MHz, $CDCl_3$) δ 202.9, 138.5, 134.5, 128.7, 128.6, 128.6, 124.8, 102.9, 88.9, 53.8, 25.7, 21.4, 18.6, 11.3. HRMS-ESI (m/z) calculated for $C_{21}H_{33}OSi^+$ $[M+H]^+$: 329.2295; found: 329.2288.

3-(3-chlorophenyl)-5-(triisopropylsilyl)pent-4-yn-2-one (**1f**)

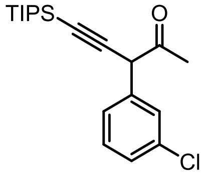

^{1}H NMR (400 MHz, Chloroform-*d*) δ 7.4 (s, 1H), 7.2 (m, 3H), 4.5 (s, 1H), 2.2 (s, 3H), 1.1 (s, 21H).^{13}C NMR (101 MHz, $CDCl_3$) δ 202.0, 136.6, 134.8, 130.0, 128.2, 128.1, 126.1, 102.0, 89.8, 53.3, 26.0, 18.6, 11.3. HRMS-ESI (m/z) calculated for $C_{20}H_{30}ClOSi^+$ $[M+H]^+$: 349.1749; found: 349.1745.

3-(3-fluorophenyl)-5-(triisopropylsilyl)pent-4-yn-2-one (**1g**)

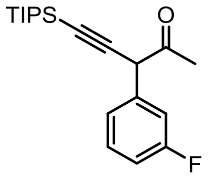

^{1}H NMR (400 MHz, Chloroform-*d*) δ 7.3 (m, 1H), 7.2 (m, 2H), 7.0 (m, 1H), 4.6 (s, 1H), 2.3 (s, 3H), 1.1 (d, J = 2.0 Hz, 21H). ^{13}C NMR (101 MHz, $CDCl_3$) δ 201.0, 162.0 (d, J = 246.8 Hz), 136.0 (d, J = 7.6 Hz), 129.2 (d, J = 8.2 Hz), 122.5 (d, J = 3.0 Hz), 114.0 (d, J = 20.2 Hz), 113.8 (d, J = 18.4 Hz), 101.0, 88.5, 52.4, 24.8, 17.6, 10.2. ^{19}F NMR (376 MHz, $CDCl_3$) δ -112.2. HRMS-ESI (m/z) calculated for $C_{20}H_{30}FOSi^+$ $[M+H]^+$: 333.2044; found: 333.2040.

3-(3-methoxyphenyl)-5-(triisopropylsilyl)pent-4-yn-2-one (**1h**)

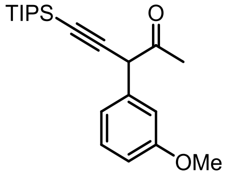

^{1}H NMR (500 MHz, Chloroform-*d*) δ 7.2 (t, J = 7.9 Hz, 1H), 7.0 (s, 1H), 7.0 (d, J = 7.7 Hz, 1H), 6.8 (dd, J = 8.2, 2.5 Hz, 1H), 4.5 (s, 1H), 3.7 (s, 3H), 2.2 (s, 3H), 1.0 (s, 21H). ^{13}C NMR (126 MHz, $CDCl_3$) δ 202.6, 160.0, 136.1, 129.8, 120.1, 113.9, 113.0, 113.0, 102.7, 89.0, 55.3, 53.9, 25.6, 18.7, 11.3. HRMS-ESI (m/z) calculated for $C_{21}H_{33}O_2Si^+$ $[M+H]^+$: 345.2244; found: 345.2238.

3-(naphthalen-2-yl)-5-(triisopropylsilyl)pent-4-yn-2-one (**1i**)

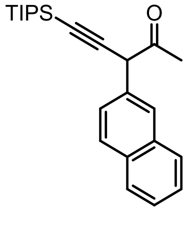

^1H NMR (400 MHz, Chloroform-*d*) δ 8.0 (s, 1H), 7.8 (t, 3H), 7.4 (m, 3H), 4.7 (s, 1H), 2.2 (s, 3H), 1.1 (s, 21H). ^{13}C NMR (101 MHz, $CDCl_3$) δ 201.7, 132.4, 131.9, 131.1, 127.5, 126.9, 126.7, 126.0, 125.4, 125.2, 124.5, 101.8, 88.3, 53.0, 24.7, 17.7, 10.3. HRMS-ESI (m/z) calculated for $C_{24}H_{33}OSi^+$ $[M+H]^+$: 365.2295; found:365.2291.

3-methyl-5-(triisopropylsilyl)pent-4-yn-2-one (**1j**)

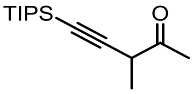

^1H NMR (400 MHz, Chloroform-*d*) δ 3.3 (q, *J* = 7.0 Hz, 1H), 2.3 (s, 3H), 1.3 (d, *J* = 7.0 Hz, 3H), 1.0 (m, 21H). ^{13}C NMR (101 MHz, $CDCl_3$) δ 205.3, 106.3, 84.8, 41.0, 27.3, 18.6, 16.7, 11.2. HRMS-ESI (m/z) calculated for $C_{15}H_{29}OSi^+$ $[M+H]^+$: 253.1982; found:253.1976.

3-phenyl-5-(p-tolyl)pent-4-yn-2-one (**1k**)

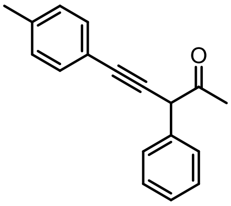

^1H NMR (500 MHz, Chloroform-*d*) δ 7.5 (d, *J* = 7.4 Hz, 2H), 7.4 (m, 4H), 7.3 (t, *J* = 7.3 Hz, 1H), 7.1 (d, *J* = 7.9 Hz, 2H), 4.8 (s, 1H), 2.4 (s, 3H), 2.3 (s, 3H). ^{13}C NMR (126 MHz, $CDCl_3$) δ 202.9, 138.7, 135.0, 131.6, 129.1, 128.9, 128.0, 127.9, 119.7, 87.4, 84.1, 53.3, 26.1, 21.5. HRMS-ESI (m/z) calculated for $C_{18}H_{17}O^+$ $[M+H]^+$: 249.1274; found:249.1268.

3-ethyl-5-phenylpent-4-yn-2-one **(1l)**

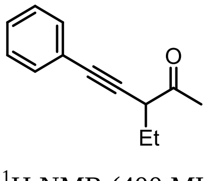

^1H NMR (400 MHz, Chloroform-*d*) δ 7.49 – 7.39 (m, 2H), 7.34 – 7.27 (m, 3H), 3.36 (dd, *J* = 8.1, 5.9 Hz, 1H), 2.37 (s, 3H), 1.96 – 1.73 (m, 2H), 1.07 (t, *J* = 7.4 Hz, 3H). ^{13}C NMR (101 MHz, Chloroform-*d*) δ 205.6, 131.7, 128.4, 128.3, 123.2, 86.7, 85.2, 48.1, 27.9, 24.9. m/z HRMS (ESI) found $[M+H]^+$ 187.1116, $C_{13}H_{15}O^+$ requires 187.1117.

3-phenylnon-4-yn-2-one (**1m**)

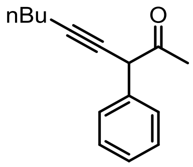

^{1}H NMR (500 MHz, Chloroform-*d*) δ 7.4 (d, *J* = 7.6 Hz, 2H), 7.4 (t, *J* = 7.5 Hz, 2H), 7.2 (t, *J* = 7.8 Hz, 1H), 4.5 (d, *J* = 2.8 Hz, 1H), 2.3 (td, *J* = 7.1, 2.3 Hz, 2H), 2.2 (s, 3H), 1.6 (m, 2H), 1.5 (m, 2H), 0.9 (t, *J* = 7.3 Hz, 3H). HRMS-ESI (m/z) calculated for $C_{15}H_{19}O^+$ $[M+H]^+$: 215.1430; found:215.1425.

3-phenylpent-4-yn-2-one (**1n**)

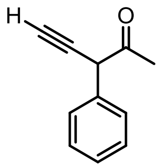

^{1}H NMR (500 MHz, Chloroform-*d*) δ 7.4 (d, *J* = 7.5 Hz, 2H), 7.4 (t, *J* = 7.4 Hz, 3H), 7.3 (d, *J* = 7.2 Hz, 1H), 4.6 (d, *J* = 2.6 Hz, 1H), 2.6 (d, *J* = 2.6 Hz, 1H), 2.2 (s, 3H). HRMS-ESI (m/z) calculated for $C_{11}H_{11}O^+$: 159.0804; found: 159.0807.

4-phenyl-6-(triisopropylsilyl)hex-5-yn-3-one (**1o**)

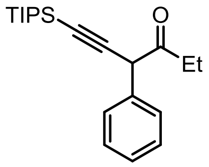

^{1}H NMR (500 MHz, Chloroform-*d*) δ 7.4 (d, *J* = 7.5 Hz, 2H), 7.3 (t, *J* = 7.4 Hz, 2H), 7.2 (t, *J* = 7.3 Hz, 1H), 4.6 (s, 1H), 2.7 (dq, *J* = 18.1, 7.3 Hz, 1H), 2.5 (dq, *J* = 18.2, 7.3 Hz, 1H), 1.0 (s, 21H), 0.9 (t, *J* = 7.2 Hz, 3H). ^{13}C NMR (101 MHz, $CDCl_3$) δ 204.5, 134.0, 127.7, 126.9, 126.7, 102.0, 87.5, 52.1, 30.5, 17.6, 10.3, 7.1. HRMS-ESI (m/z) calculated for $C_{21}H_{33}OSi^+$ $[M+H]^+$: 329.2295; found:329.2288.

3-phenyl-1-(triisopropylsilyl)oct-1-yn-4-one (**1p**)

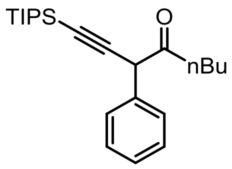

^{1}H NMR (500 MHz, Chloroform-*d*) δ 7.4 (d, *J* = 7.6 Hz, 2H), 7.3 (t, *J* = 7.5 Hz, 2H), 7.2 (t, *J* = 7.4 Hz, 1H), 4.6 (s, 1H), 2.7 (ddd, *J* = 17.4, 8.5, 6.2 Hz, 1H), 2.4 (ddd, *J* = 17.4, 8.4, 6.4 Hz, 1H), 1.4 (m, 2H), 1.1 (qd, *J* = 7.2, 2.9 Hz, 1H), 1.0 (m, 22H), 0.7 (t, *J* = 7.4 Hz, 3H). ^{13}C NMR (126 MHz, $CDCl_3$) δ 205.0, 134.9, 128.7, 127.9, 127.7, 103.0, 88.6, 53.4, 38.0, 25.9, 22.1, 18.7, 13.7, 11.3. HRMS-ESI (m/z) calculated for $C_{23}H_{37}OSi^+$ $[M+H]^+$: 357.2608; found:357.2601.

2-methyl-4-phenyl-6-(triisopropylsilyl)hex-5-yn-3-one (**1q**)

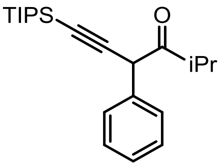

^{1}H NMR (400 MHz, Chloroform-*d*) δ 7.4 (d, *J* = 7.4 Hz, 2H), 7.3 (t, *J* = 7.4 Hz, 2H), 7.2 (m, 1H), 4.6 (s, 1H), 3.1 (p, *J* = 6.8 Hz, 1H), 1.1 (d, *J* = 6.9 Hz, 3H), 1.0 (s, 21H), 0.8 (d, *J* = 6.8 Hz, 3H). ^{13}C NMR (101 MHz, Chloroform-*d*) δ 208.6, 134.9, 128.8, 128.3, 127.8, 102.9, 88.4, 52.5, 37.5, 20.0, 19.7, 18.8, 11.4. HRMS-ESI (m/z) calculated for $C_{22}H_{35}OSi^+$ $[M+H]^+$: 343.2452; found:343.2446.

3. Synthesis of allenamide substrates

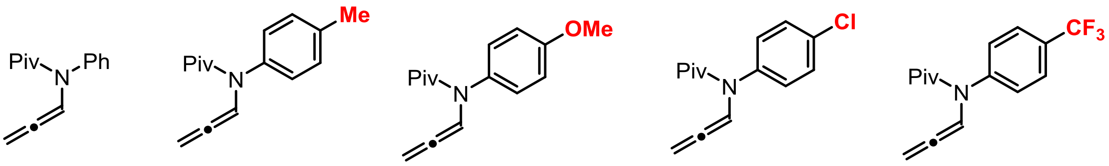

2a **2r** **2s** **2t** **2u**

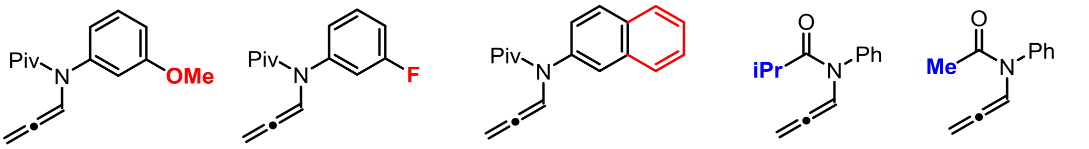

2v **2w** **2x** **2y** **2z**

General procedure for the synthesis of allenamide 2:

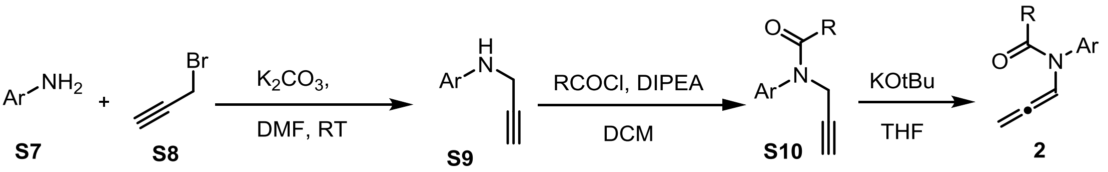

Allenamides **2** were prepared from a known procedure from aniline **S7** and propargyl bromide **S8** in three steps[3]. The NMR date of allenamide **2r** matched with literature[4].

N-(propa-1,2-dien-1-yl)-N-(p-tolyl)pivalamide (**2r**)

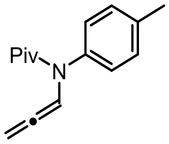

^{1}H NMR (500 MHz, Chloroform-*d*) δ 7.7 (t, *J* = 6.4 Hz, 1H), 7.2 (d, *J* = 6.5 Hz, 2H), 7.1 (d, *J* = 6.6 Hz, 2H), 4.9 (dd, *J* = 6.4, 1.8 Hz, 2H), 2.4 (s, 3H), 1.1 (s, 9H). ^{13}C NMR (126 MHz, $CDCl_3$) δ 202.8, 176.0, 138.3, 137.8, 129.9, 129.2, 104.2, 85.8, 41.2, 29.3, 21.2. IR: ν = 3033, 2970, 2931, 2873, 1728, 1645, 1620 cm^{-1}. HRMS-ESI (m/z) calculated for $C_{15}H_{20}NO^+$ [M+H]$^+$: 230.1539; found:230.1542.

N-(4-chlorophenyl)-N-(propa-1,2-dien-1-yl)pivalamide (**2t**)

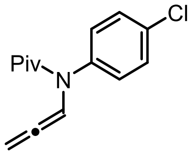

^{1}H NMR (500 MHz, Chloroform-*d*) δ 7.6 (t, *J* = 6.3 Hz, 1H), 7.4 (d, *J* = 7.2 Hz, 2H), 7.1 (d, *J* = 7.5 Hz, 2H), 4.9 (m, 2H), 1.1 (s, 9H). ^{13}C NMR (126 MHz, $CDCl_3$) δ 202.5, 175.8, 139.1, 134.2, 131.5, 129.0, 104.1, 86.4, 41.2, 29.3. IR: ν = 3089, 3057, 2972, 2935, 2906, 2873, 1726, 1647, 1595 cm^{-1}.HRMS-ESI (m/z) calculated for $C_{14}H_{17}ClNO^+$ [M+H]$^+$: 250.0993; found:250.0994.

N-(propa-1,2-dien-1-yl)-N-(4-(trifluoromethyl)phenyl)pivalamide (**2u**)

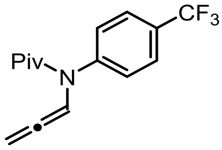

^{1}H NMR (500 MHz, Chloroform-*d*) δ 7.7 (d, *J* = 8.2 Hz, 2H), 7.6 (t, *J* = 6.4 Hz, 1H), 7.3 (d, *J* = 8.1 Hz, 2H), 4.9 (d, *J* = 6.4 Hz, 2H), 1.1 (s, 9H). ^{13}C NMR (126 MHz, $CDCl_3$) δ 202.4, 175.8, 144.0, 130.5, 130.4 (q, *J* = 32.8 Hz) 126.0 (q, *J* = 3.7 Hz), 123.7 (d, *J* = 272.3 Hz), 103.9, 86.5, 41.2, 29.2. ^{19}F NMR (471 MHz, Chloroform-*d*) δ -62.5. IR: ν = 3057, 2970, 2933, 2871, 1650, 1614 cm^{-1}. HRMS-ESI (m/z) calculated for $C_{15}H_{17}F_3NO^+$ [M+H]$^+$: 284.1257; found:284.1259.

N-(3-methoxyphenyl)-N-(propa-1,2-dien-1-yl)pivalamide (**2v**)

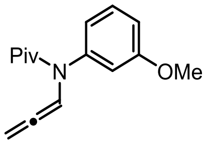

^{1}H NMR (500 MHz, Chloroform-*d*) δ 7.7 (t, *J* = 6.4 Hz, 1H), 7.3 (t, *J* = 8.1 Hz, 1H), 6.9 (dd, *J* = 8.4, 2.6 Hz, 1H), 6.8 (d, *J* = 7.9 Hz, 0H), 6.7 (t, *J* = 2.3 Hz, 1H), 4.9 (d, *J* = 6.4 Hz, 2H), 3.8 (s, 3H), 1.1 (s, 9H). ^{13}C NMR (126 MHz, $CDCl_3$) δ 202.7, 175.9, 159.8, 141.6, 129.2, 122.5, 116.0, 113.9, 103.9, 85.9, 55.4, 41.3, 29.3. IR: ν = 3060, 2964, 2935, 2904, 2935, 2904, 2877, 2835, 1726, 1645, 1601 cm^{-1}. HRMS-ESI (m/z) calculated for $C_{15}H_{20}NO_2^+$ $[M+H]^+$: 246.1489; found:246.1490.

N-(3-fluorophenyl)-N-(propa-1,2-dien-1-yl)pivalamide (**2w**)

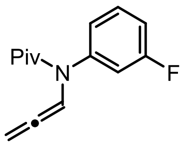

^{1}H NMR (500 MHz, Chloroform-*d*) δ 7.6 (t, *J* = 6.4 Hz, 1H), 7.4 (m, 1H), 7.1 (td, *J* = 8.4, 1.6 Hz, 1H), 7.0 (m, 1H), 6.9 (d, *J* = 9.3 Hz, 1H), 4.9 (d, *J* = 6.4 Hz, 1H), 1.1 (s, 9H). ^{13}C NMR (126 MHz, $CDCl_3$) δ 202.5, 175.7, 162.5 (d, *J* = 248.1 Hz), 142.0 (d, *J* = 9.6 Hz), 129.7 (d, *J* = 9.0 Hz), 126.0 (d, *J* = 3.3 Hz), 117.5 (d, *J* = 21.9 Hz), 115.5 (d, *J* = 20.8 Hz), 103.9, 86.2, 41.3, 29.2. ^{19}F NMR (471 MHz, Chloroform-*d*) δ -111.7 (d, *J* = 2.3 Hz). IR: ν = 3060, 2960, 2935, 2910, 2875, 1732, 1650, 1607, 1590 cm^{-1}. HRMS-ESI (m/z) calculated for $C_{14}H_{17}FNO^+$ $[M+H]^+$: 234.1289; found:234.1291.

N-(naphthalen-2-yl)-N-(propa-1,2-dien-1-yl)pivalamide (**2x**)

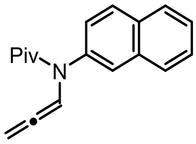

^{1}H NMR (500 MHz, Chloroform-*d*) δ 7.9 (q, *J* = 7.8 Hz, 3H), 7.7 (t, *J* = 6.3 Hz, 1H), 7.7 (s, 1H), 7.5 (m, 2H), 7.3 (d, *J* = 8.5 Hz, 1H), 4.8 (d, *J* = 6.4 Hz, 2H), 1.1 (s, 9H). ^{13}C NMR (126 MHz, $CDCl_3$) δ 202.8, 176.2, 137.9, 133.0, 132.7, 128.6, 128.5, 128.2, 128.1, 127.8, 126.8, 126.5, 104.3, 86.1, 41.3, 29.4. IR: ν = 3055, 2964, 2931, 2868, 1645, 1629, 1597 cm^{-1}.HRMS-ESI (m/z) calculated for $C_{18}H_{20}NO^+$ $[M+H]^+$: 266.1539; found: 266.1537.

4. Asymmetric synthesis of chiral products

General procedure for asymmetric synthesis of 3:

To a dried sealed tube was added 4Å molecular sieves (200 mg), allenamide **2** (0.22 mmol) and (*S*)-**A9** (15.2 mg 0.02 mmol) sequentially, which was followed by adding a solution of ketone **1** (0.2 mmol) in CCl_4 (2 mL). After stirring at the room temperature for 20 h, the mixture was filtered through Celite and the filtrate was concentrated under vacuum to give a residue, which was purified by column chromatography on silica gel (petroleum ether:EtOAc, 30:1 ~10:1) to give the products **3**.

(*S*,*E*)-N-(4-acetyl-4-phenyl-6-(triisopropylsilyl)hex-1-en-5-yn-1-yl)-N-phenylpivalamide (**3a**)

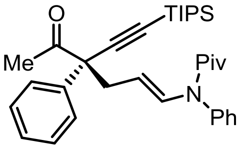

92 mg, 87% yield. ^{1}H NMR (500 MHz, Chloroform-*d*) δ 7.3 (m, 9H), 7.0 (d, *J* = 7.1 Hz, 2H), 4.1 (dt, *J* = 14.6, 7.5 Hz, 1H), 2.8 (dd, *J* = 13.9, 6.7 Hz, 1H), 2.6 (dd, *J* = 13.9, 8.4 Hz, 1H), 2.1 (s, 3H), 1.1 (s, 21H), 1.0 (s, 9H). ^{13}C NMR (126 MHz, $CDCl_3$) δ 203.9, 175.8, 140.2, 137.8, 134.1, 130.2, 128.9, 128.6, 128.1, 127.5, 126.8, 109.0, 106.1, 90.0, 59.9, 40.8, 39.8, 29.1, 26.7, 18.7, 11.2. $[\alpha]_D^{23}$ = 22.8 (c 1.0, $CHCl_3$). IR: ν = 3084, 3060, 2960, 2943, 2893, 2867, 2164, 1721, 1661, 1645, 1597 cm^{-1}. HRMS-APCI (m/z) calculated for $C_{34}H_{48}NO_2Si^+$ $[M+H]^+$: 530.3449; found: 530.3452. HPLC: Chiralpak IC column, 95:05 hexanes/isopropanol, 1 mL/min; t_R = 9.7 min (major), 11.9 min (minor); 97:3 er.

(*S*,*E*)-N-(4-acetyl-4-(p-tolyl)-6-(triisopropylsilyl)hex-1-en-5-yn-1-yl)-N-phenylpivalamide (**3b**)

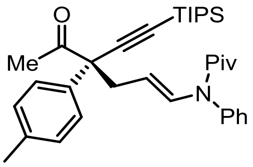

92 mg, 85% yield. ^{1}H NMR (400 MHz, Chloroform-*d*) δ 7.3 (m, 3H), 7.2 (m, 3H), 7.1 (d, *J* = 8.0 Hz, 2H), 7.0 (d, *J* = 6.2 Hz, 2H), 4.1 (dt, *J* = 14.5, 7.6 Hz, 1H), 2.7 (dd, *J* = 13.4, 6.4 Hz, 1H), 2.6 (dd, *J* = 13.8, 8.4 Hz, 1H), 2.3 (s, 3H), 2.1 (s, 3H), 1.1 (s, 21H), 1.0 (s, 9H). ^{13}C NMR (101 MHz, $CDCl_3$) δ 204.0, 175.8, 140.3, 137.2, 134.9, 134.0, 130.2, 129.3, 129.0, 128.0, 126.7, 109.1, 106.4, 89.7, 59.6, 40.8, 39.8, 29.1, 26.6, 21.0, 18.7, 11.3. $[\alpha]_D^{23}$ = -11.6 (c 1.0, $CHCl_3$). IR: ν = 3080, 3053, 2960, 2943, 2924, 2893, 2865, 2168, 1719, 1659, 1642, 1595 cm^{-1}. HRMS-APCI (m/z)

calculated for $C_{35}H_{50}NO_2Si^+$ [M+H]$^+$: 544.3605; found: 544.3610. HPLC: Chiralpak ID column, 90:10 hexanes/isopropanol, 1 mL/min; t_R = 8.1 min (minor), 9.2 min (major); 97.5:2.5 er.

(*S*,*E*)-N-(4-acetyl-4-(4-chlorophenyl)-6-(triisopropylsilyl)hex-1-en-5-yn-1-yl)-N-phenylpivalamide (**3c**)

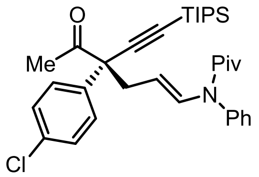

100 mg, 89% yield. ^{1}H NMR (500 MHz, Chloroform-*d*) δ 7.2 (m, 8H), 6.9 (d, *J* = 6.7 Hz, 2H), 4.0 (dt, *J* = 14.6, 7.6 Hz, 1H), 2.7 (dd, *J* = 14.0, 6.7 Hz, 1H), 2.5 (dd, *J* = 14.0, 8.4 Hz, 1H), 2.1 (s, 3H), 1.0 (s, 21H), 0.9 (s, 9H). ^{13}C NMR (126 MHz, $CDCl_3$) δ 202.4, 174.8, 139.1, 135.4, 133.4, 132.5, 129.2, 128.0, 127.7, 127.3, 127.2, 107.4, 104.7, 89.5, 58.4, 39.9, 38.8, 28.1, 25.7, 17.7, 10.2. $[\alpha]_D^{23}$ = -11.6 (c 1.0, $CHCl_3$). IR: ν = 3062, 2960, 2941, 2891, 2866, 2166, 1719, 1665, 1651, 1594 cm^{-1}. HRMS-APCI (m/z) calculated for $C_{34}H_{47}ClNO_2Si^+$ [M+H]$^+$: 564.3059; found: 564.3064. HPLC: Chiralpak ID column, 90:10 hexanes/isopropanol, 1 mL/min; t_R = 7.8 min (minor), 8.4 min (major); 97:3 er.

(*S*,*E*)-N-(4-acetyl-4-(4-(trifluoromethyl)phenyl)-6-(triisopropylsilyl)hex-1-en-5-yn-1-yl)-N-phenyl pivalamide (**3d**)

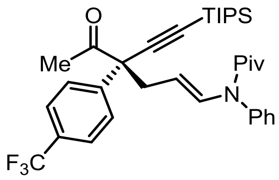

102 mg, 85% yield. ^{1}H NMR (400 MHz, Chloroform-*d*) δ 7.6 (d, *J* = 8.3 Hz, 2H), 7.5 (d, *J* = 8.2 Hz, 2H), 7.3 (m, 4H), 6.9 (d, *J* = 7.7 Hz, 1H), 4.0 (dt, *J* = 14.6, 7.6 Hz, 1H), 2.8 (dd, *J* = 14.2, 6.5 Hz, 1H), 2.6 (dd, *J* = 13.9, 8.3 Hz, 1H), 2.1 (s, 3H), 1.1 (s, 21H), 1.0 (s, 9H). ^{13}C NMR (101 MHz, $CDCl_3$) δ 203.1, 175.8, 141.9, 140.1, 134.6, 130.2, 129.8 (d, *J* = 32.6 Hz), 129.0, 128.2, 127.4, 125.6 (q, *J* = 3.9 Hz), 124.0 (d, *J* = 272.0 Hz), 108.1, 105.4, 91.0, 59.9, 40.9, 39.9, 29.1, 26.9, 18.7, 11.2. ^{19}F NMR (376 MHz, $CDCl_3$) δ -62.54. $[\alpha]_D^{23}$ = -25.2 (c 1.0, $CHCl_3$). IR: ν = 3053, 2989, 2960, 2945, 2893, 2868, 2170, 1721, 1661, 1645, 1620, 1597 cm^{-1}. HRMS-APCI (m/z) calculated for $C_{35}H_{47}F_3NO_2Si^+$ [M+H]$^+$: 598.3323; found: 598.3329. HPLC: Chiralpak IC column, 90:10 hexanes/isopropanol, 1 mL/min; t_R = 5.9 min (minor), 6.5 min (major); 97.5:2.5 er.

(*S*,*E*)-N-(4-acetyl-4-(m-tolyl)-6-(triisopropylsilyl)hex-1-en-5-yn-1-yl)-N-phenylpivalamide (**3e**)

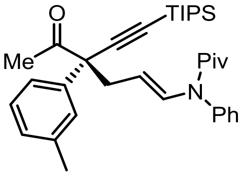

84 mg, 77% yield. ^{1}H NMR (500 MHz, Chloroform-*d*) δ 7.3 (m, 4H), 7.2 (m, 2H), 7.1 (m, 2H), 7.0 (d, *J* = 7.2 Hz, 2H), 4.1 (dt, *J* = 14.7, 7.5 Hz, 1H), 2.7 (dd, *J* = 13.9, 6.8 Hz, 1H), 2.6 (dd, *J* = 13.9, 8.3 Hz, 1H), 2.3 (s, 3H), 2.1 (s, 3H), 1.1 (s, 21H), 1.0 (s, 9H). ^{13}C NMR (101 MHz, $CDCl_3$) δ 204.0, 175.8, 140.3, 138.1, 137.8, 134.0, 130.1, 128.9, 128.5, 128.2, 128.1, 127.7, 123.8, 109.1, 106.3, 89.9, 59.9, 40.8, 39.8, 29.1, 26.7, 21.5, 18.7, 11.3.$[\alpha]_D^{23}$ = -22.0 (c 1.0, $CHCl_3$). IR: ν = 3062, 2958, 2943, 2891, 2864, 2166, 1719, 1663, 1647, 1597 cm^{-1}. HRMS-APCI (m/z) calculated for $C_{35}H_{50}NO_2Si^+$ $[M+H]^+$: 544.3605; found: 544.3609. HPLC: Chiralpak IC column, 90:10 hexanes/isopropanol, 1 mL/min; t_R = 6.5 min (major), 7.8 min (minor); 97:3 er.

(*S*,*E*)-N-(4-acetyl-4-(3-chlorophenyl)-6-(triisopropylsilyl)hex-1-en-5-yn-1-yl)-N-phenylpivalamid e (**3f**)

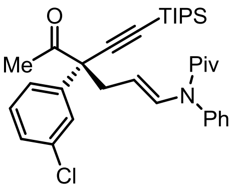

86 mg, 76% yield. ^{1}H NMR (500 MHz, Chloroform-*d*) δ 7.5 (s, 1H), 7.3 (m, 3H), 7.2 (m, 3H), 7.1 (m, 1H), 7.0 (d, *J* = 7.3 Hz, 2H), 4.0 (dt, *J* = 14.5, 7.6 Hz, 1H), 2.7 (dd, *J* = 13.8, 6.6 Hz, 1H), 2.6 (dd, *J* = 13.8, 8.5 Hz, 1H), 2.1 (s, 3H), 1.1 (s, 21H), 1.0 (s, 9H). ^{13}C NMR (126 MHz, $CDCl_3$) δ 203.2, 175.8, 140.1, 140.0, 134.6, 134.5, 130.2, 129.9, 129.0, 128.2, 127.8, 127.7, 124.6, 108.3, 105.4, 90.8, 59.8, 40.9, 39.8, 29.1, 26.8, 18.7, 11.2. $[\alpha]_D^{23}$ = -23.0 (c 1.0, $CHCl_3$). IR: ν = 3064, 3080, 2964, 2943, 2923, 2898, 2866, 2166, 1721, 1662, 1643, 1595, 1570 cm^{-1}. HRMS-APCI (m/z) calculated for $C_{34}H_{47}ClNO_2Si^+$ $[M+H]^+$: 564.3059; found: 564.3064. HPLC: Chiralpak ID column, 95:05hexanes/isopropanol, 1 mL/min; t_R = 10.5 min (minor), 11.7 min (major); 97:3 er.

(*S*,*E*)-N-(4-acetyl-4-(3-fluorophenyl)-6-(triisopropylsilyl)hex-1-en-5-yn-1-yl)-N-phenylpivalamid e (**3g**)

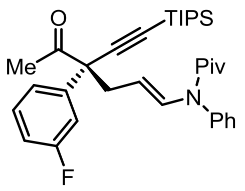

93 mg, 85% yield. ^{1}H NMR (400 MHz, Chloroform-*d*) δ 7.2 (m, 4H), 7.2 (m, 1H), 7.1 (d, *J* = 10.2 Hz, 1H), 6.9 (m, 4H), 3.9 (dt, *J* = 14.6, 7.6 Hz, 1H), 2.7 (dd, *J* = 13.9, 6.2 Hz, 1H), 2.5 (dd, *J* = 13.9, 8.4 Hz, 1H), 2.1 (s, 3H), 1.0 (s, 21H), 0.9 (s, 9H). ^{13}C NMR (101 MHz, $CDCl_3$) δ 202.2, 174.8, 161.8 (d, *J* = 246.4 Hz), 139.5 (d, *J* = 7.0 Hz), 139.1, 133.4, 129.2, 129.1 (d, *J* = 8.0 Hz), 128.0, 127.2, 121.1 (d, *J* = 2.9 Hz), 113.5 (d, *J* = 37.7 Hz), 113.5 (d, *J* = 6.6 Hz), 107.4, 104.5, 89.4, 58.8 (d, *J* = 1.7 Hz), 39.9, 38.8, 28.1, 25.7, 17.7, 10.2. ^{19}F NMR (376 MHz, $CDCl_3$) δ -112.3. $[\alpha]_D^{23}$ = -30.2 (c 1.0, $CHCl_3$). IR: ν = 3053, 2991, 2960, 2943, 2868, 2164, 1723, 1659, 1645, 1611, 1590 cm^{-1}. HRMS-APCI (m/z) calculated for $C_{34}H_{47}FNO_2Si^+$ $[M+H]^+$: 548.3355; found: 548.3359. HPLC: Chiralpak IC column, 90:10 hexanes/isopropanol, 1 mL/min; t_R = 6.2min (minor), 6.8 min 83 mg (major); 97.5:2.5 er.

(*S,E*)-N-(4-acetyl-4-(3-methoxyphenyl)-6-(triisopropylsilyl)hex-1-en-5-yn-1-yl)-N-phenylpivalamide (**3h**)

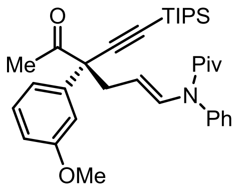

100 mg, 89% yield. ^{1}H NMR (500 MHz, Chloroform-*d*) δ 7.3 (m, 4H), 7.2 (t, *J* = 7.9 Hz, 1H), 7.0 (m, 3H), 6.8 (d, *J* = 7.8 Hz, 1H), 6.8 (d, *J* = 8.4 Hz, 1H), 4.1 (dt, *J* = 14.6, 7.6 Hz, 1H), 3.8 (s, 3H), 2.7 (dd, *J* = 13.9, 6.8 Hz, 1H), 2.6 (dd, *J* = 13.9, 8.4 Hz, 1H), 2.1 (s, 3H), 1.1 (s, 21H), 1.0 (s, 9H). ^{13}C NMR (126 MHz, $CDCl_3$) δ 203.6, 175.8, 159.8, 140.2, 139.4, 134.1, 130.2, 129.6, 128.9, 128.1, 118.9, 113.3, 112.5, 109.0, 106.1, 89.9, 60.0, 55.2, 40.8, 39.8, 29.1, 26.6, 18.7, 11.2. $[\alpha]_D^{23}$ = -23.6 (c 1.0, $CHCl_3$). IR: ν = 3052, 2960, 2943, 2891, 2864, 2164, 1719, 1659, 1642, 1597, 1584 cm^{-1}. HRMS-APCI (m/z) calculated for $C_{35}H_{50}NO_3Si^+$ $[M+H]^+$: 560.3554; found: 560.3562. HPLC: Chiralpak ID column, 90:10 hexanes/isopropanol, 1 mL/min; t_R = 9.6min (minor), 11.1 min, 83 mg (major); 97.5:2.5 er.

(*S*,*E*)-N-(4-acetyl-4-(naphthalen-2-yl)-6-(triisopropylsilyl)hex-1-en-5-yn-1-yl)-N-phenylpivalamide (**3i**)

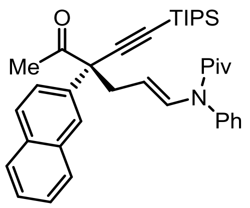

83 mg, 72% yield. ^{1}H NMR (500 MHz, Chloroform-*d*) δ 8.0 (s, 1H), 7.8 (m, 3H), 7.5 (p, *J* = 7.0 Hz, 2H), 7.2 (m, 2H), 7.2 (m, 3H), 6.8 (s, 2H), 4.1 (dt, *J* = 14.7, 7.6 Hz, 1H), 2.8 (dd, *J* = 14.0, 6.6 Hz, 1H), 2.7 (dd, *J* = 14.0, 8.5 Hz, 1H), 2.1 (s, 3H), 1.1 (s, 21H), 0.9 (s, 9H). ^{13}C NMR (126 MHz, $CDCl_3$) δ 203.9, 175.8, 140.1, 135.2, 134.1, 133.2, 132.6, 130.0, 128.8, 128.5, 128.0, 127.6, 126.8, 126.3, 126.3, 123.9, 109.1, 106.2, 90.3, 60.1, 40.8, 39.4, 29.0, 26.7, 18.8, 11.3. $[\alpha]_D^{23}$ = 19.8 (c 1.0, $CHCl_3$). IR: ν = 3080, 3055, 2956, 2922, 2893, 2864, 2164, 1719, 1663, 1646, 1597 cm^{-1}. HRMS-APCI (m/z) calculated for $C_{38}H_{50}NO_2Si^+$ $[M+H]^+$: 580.3605; found: 580.3606. HPLC: Chiralpak IC column, 95:05 hexanes/isopropanol, 1 mL/min; t_R = 10.8 min (major), 13.7 min (minor); 98:2 er.

(*R*,*E*)-N-(4-acetyl-4-methyl-6-(triisopropylsilyl)hex-1-en-5-yn-1-yl)-N-phenylpivalamide (**3j**)

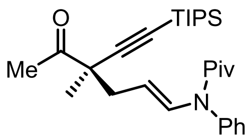

84 mg, 90% yield, ^{1}H NMR (500 MHz, Chloroform-*d*) δ 7.5 (d, *J* = 14.2 Hz, 1H), 7.4 (m, 3H), 7.2 (d, *J* = 7.4 Hz, 2H), 4.3 (dt, *J* = 14.8, 7.7 Hz, 1H), 2.4 (dd, *J* = 13.7, 7.1 Hz, 1H), 2.3 (s, 3H), 2.2 (dd, *J* = 13.7, 8.3 Hz, 1H), 1.2 (s, 3H), 1.1 (s, 9H), 1.0 (d, *J* = 4.8 Hz, 21H). ^{13}C NMR (126 MHz, $CDCl_3$) δ208.0, 176.0, 140.1, 134.5, 130.3, 129.2, 128.5, 109.7, 108.3, 85.2, 49.4, 41.1, 40.1, 29.2, 27.2, 23.9, 18.6, 11.1. $[\alpha]_D^{23}$ = -18.2(c 1.0, $CHCl_3$). IR: ν = 3068, 2958, 2941, 2889, 2868, 2164, 1717, 1663, 1649, 1599 cm^{-1}. HRMS-APCI (m/z) calculated for $C_{29}H_{46}NO_2Si^+$ $[M+H]^+$: 468.3292; found: 468.3294. HPLC: Chiralpak IC column, 95:05 hexanes/isopropanol, 1 mL/min; t_R = 13.5 min (major), 16.9 min (minor); 92:8 er.

(*S*,*E*)-N-(4-acetyl-4-phenyl-6-(p-tolyl)hex-1-en-5-yn-1-yl)-N-phenylpivalamide (**3k**)

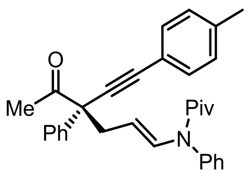

This reaction was performed on 0.1 mmol scale, which provided the product **3k** 40 mg, 86% yield, ^{1}H NMR (400 MHz, Chloroform-*d*) δ 7.2 (m, 11H), 7.0 (d, *J* = 8.0 Hz, 2H), 7.0 (d, *J* = 6.1 Hz, 2H), 4.1 (dt, *J* = 14.4, 7.7 Hz, 1H), 2.8 (dd, *J* = 13.7, 7.1 Hz, 1H), 2.5 (dd, *J* = 13.7, 8.1 Hz, 1H), 2.3 (s, 3H), 2.1 (s, 3H), 1.0 (s, 9H). ^{13}C NMR (101 MHz, $CDCl_3$) δ 203.0, 174.9, 139.4, 136.3, 134.3, 133.1, 130.6, 129.2, 128.4, 128.0, 127.4, 127.3, 127.0, 125.7, 121.8, 108.7, 88.0, 87.3, 58.3, 39.8, 38.5, 28.0, 25.8, 20.0. $[\alpha]_D^{23}$ = -39.0 (c 1.0, $CHCl_3$). IR: ν = 3087, 3062, 3020, 2962, 2929, 2910, 2873, 2160, 1715, 1690, 1659, 1640, 1597 cm^{-1}. HRMS-APCI (m/z) calculated for $C_{32}H_{34}NO_2^+$ $[M+H]^+$: 464.2584; found: 464.2587. HPLC: Chiralpak IC column, 85:15 hexanes/isopropanol, 1 mL/min; t_R = 13.8min (major), 16.0min (minor); 90.5:9.5 er

(*R,E*)-N-(4-acetyl-4-ethyl-6-phenylhex-1-en-5-yn-1-yl)-N-phenylpivalamide (**3l**)

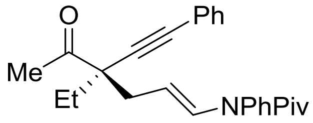

49 mg, 60% yield. ^{1}H NMR (500 MHz, Chloroform-*d*) δ 7.5 (d, *J* = 14.1 Hz, 1H), 7.4 (m, 3H), 7.3 (m, 5H), 7.2 (d, *J* = 7.3 Hz, 2H), 4.3 (dt, *J* = 14.6, 7.7 Hz, 1H), 2.5 (m, 1H), 2.3 (m, 1H), 2.3 (s, 3H), 1.8 (m, 1H), 1.5 (m, 1H), 1.1 (s, 9H), 0.9 (t, *J* = 7.4 Hz, 3H). ^{13}C NMR (126 MHz, $CDCl_3$) δ 208.7, 176.0, 140.3, 134.3, 131.6, 130.5, 129.2, 128.4, 128.2, 128.1, 123.2, 108.7, 90.3, 86.4, 54.8, 41.1, 38.9, 30.6, 29.2, 29.2, 9.6. $[\alpha]_D^{27}$ = 33 (c 1.0, $CHCl_3$). IR: ν = 3061, 2971, 2928, 2875, 2169, 1717, 1644, 1599，1526 cm^{-1}. HRMS-APCI (m/z) calculated for $C_{34}H_{48}NO_2Si^+$ $[M+H]^+$: 402.2428; found: 402.2419. HPLC: Chiralpak ICcolumn, 90:10 hexanes/isopropanol, 1 mL/min; t_R = 13.5 min (major), 14.7 min (minor); 82.5:17.5 er.

(*S,E*)-N-(4-acetyl-4-phenyldec-1-en-5-yn-1-yl)-N-phenylpivalamide (**3m**)

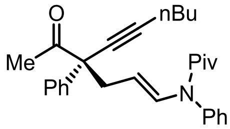

This reaction was performed on 0.1 mmol scale, which provided the product **3l** 37 mg, 87% yield. ^{1}H NMR (500 MHz, Chloroform-*d*) δ 7.3 (m, 9H), 7.0 (m, 2H), 4.1 (dt, *J* = 14.7, 7.6 Hz, 1H), 2.7 (dd, *J* = 13.7, 7.0 Hz, 1H), 2.5 (dd, *J* = 13.7, 8.2 Hz, 1H), 2.2 (t, *J* = 7.0 Hz, 2H), 2.1 (s, 3H), 1.4 (ddt, *J* = 36.8, 14.8, 6.9 Hz, 4H), 1.0 (s, 9H), 0.9 (t, *J* = 7.2 Hz, 3H). ^{13}C NMR (126 MHz, $CDCl_3$) δ 204.8, 175.9, 140.4, 138.6, 133.8, 130.2, 128.9, 128.5, 128.0, 127.4, 126.8, 109.9, 89.4, 78.5, 58.9, 40.8, 39.6, 30.9, 29.0, 26.6, 22.0, 18.5, 13.6. $[\alpha]_D^{23}$ = 3.4(c 1.0, $CHCl_3$). IR: ν = 3062, 2992, 2966, 2931, 2897, 2160, 1719, 1659, 1645, 1595 cm^{-1}. HRMS-APCI (m/z) calculated for

$C_{29}H_{36}NO_2^+$ $[M+H]^+$: 430.2741; found: 430.2744. HPLC: Chiralpak IC column, 85:15 hexanes/isopropanol, 1 mL/min; t_R = 9.1min (major), 10.5min (minor); 93:7 er.

(*R*,*E*)-N-(4-acetyl-4-phenylhex-1-en-5-yn-1-yl)-N-phenylpivalamide (**3n**)

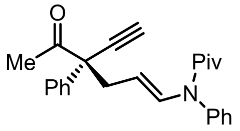

30 mg, 40% yield. ^{1}H NMR (500 MHz, Chloroform-*d*) δ 7.2 (m, 9H), 6.9 (d, *J* = 6.8 Hz, 2H), 4.0 (dt, *J* = 15.2, 7.6 Hz, 1H), 2.7 (dd, *J* = 13.9, 7.3 Hz, 1H), 2.5 (m, 2H), 2.0 (s, 3H), 1.0 (s, 9H). ^{13}C NMR (126 MHz, $CDCl_3$) δ 202.5, 175.0, 139.3, 136.4, 133.3, 129.2, 127.9, 127.6, 127.1, 126.6, 125.7, 108.0, 81.6, 76.2, 57.9, 39.8, 38.2, 28.0, 25.5. $[\alpha]_D^{23}$ = 36.4 (c 1.0, $CHCl_3$). IR: ν = 3299, 3057, 2991,,2962, 2924, 2873, 2854, 2160, 1738, 1659, 1640, 1597 cm^{-1}. HRMS-ESI (m/z) calculated for $C_{25}H_{28}NO_2^+$ $[M+H]^+$: 374.2115; found: 374.2113. HPLC: Chiralpak ID column, 90:10 hexanes/isopropanol, 1 mL/min; t_R = 12.3 min (minor), 15.8 min (major); 93:7 er.

(*S*,*E*)-N-(5-oxo-4-phenyl-4-((triisopropylsilyl)ethynyl)hept-1-en-1-yl)-N-phenylpivalamide (**3o**)

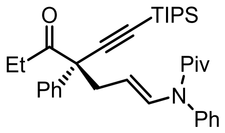

This reaction was performed on 0.1 mmol scale, which provided the product **3n** 29.9 mg, 55% yield. ^{1}H NMR (400 MHz, Chloroform-*d*) δ 7.3 (m, 9H), 7.0 (d, *J* = 6.1 Hz, 2H), 4.1 (m, 1H), 2.7 (m, 3H), 2.3 (dq, *J* = 17.6, 7.2 Hz, 1H), 1.1 (s, 21H), 1.0 (s, 9H), 0.9 (t, *J* = 7.3 Hz, 3H). ^{13}C NMR (101 MHz, $CDCl_3$) δ 207.0, 175.8, 140.2, 138.0, 134.1, 130.2, 128.9, 128.5, 128.1, 127.4, 126.9, 109.1, 106.3, 89.7, 59.7, 40.8, 40.1, 32.3, 29.1, 18.7, 11.3, 8.8. $[\alpha]_D^{23}$ = -25.2 (c 1.0, $CHCl_3$). IR: ν = 3084, 3057, 2956, 2927, 2866, 2162, 1723, 1663, 1649, 1597 cm^{-1}. HRMS-APCI (m/z) calculated for $C_{35}H_{50}NO_2Si^+$ $[M+H]^+$: 544.3605; found: 544.3608. HPLC: Chiralpak IC column, 90:10 hexanes/isopropanol, 1 mL/min; t_R = 5.9 min (major), 7.2 min (minor); 98:2 er.

(*S*,*E*)-N-(5-oxo-4-phenyl-4-((triisopropylsilyl)ethynyl)non-1-en-1-yl)-N-phenylpivalamide (**3p**)

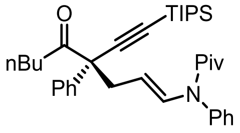

53 mg, 46% yield. ^{1}H NMR (500 MHz, Chloroform-*d*) δ 7.3 (m, 9H), 7.0 (d, *J* = 7.0 Hz, 2H), 4.1 (dt, *J* = 14.6, 7.6 Hz, 1H), 2.7 (m, 2H), 2.6 (dd, *J* = 13.9, 8.4 Hz, 1H), 2.3 (m, 1H), 1.4 (m, 2H), 1.0 (m, 32H), 0.8 (t, *J* = 7.3 Hz, 3H). ^{13}C NMR (126 MHz, $CDCl_3$) δ 206.2, 175.8, 140.2, 137.9, 134.1, 130.1, 128.9, 128.5, 128.1, 127.4, 126.9, 109.1, 106.4, 89.7, 59.8, 40.8, 40.1, 38.8, 29.1, 26.5, 22.1, 18.7, 13.7, 11.3. $[\alpha]_D^{23}$ = -19.4(c 1.0, $CHCl_3$). IR: ν = 3060, 2960, 2943, 2897, 2866, 2164, 1719, 1663, 1649, 1597 cm^{-1}. HRMS-APCI (m/z) calculated for $C_{37}H_{54}NO_2Si^+$ $[M+H]^+$: 572.3918; found: 572.3922. HPLC: Chiralpak IC column, 90:10 hexanes/isopropanol, 1 mL/min; t_R = 5.4 min (major), 6.2min (minor); 98:2 er.

(*S*,*E*)-N-(6-methyl-5-oxo-4-phenyl-4-((triisopropylsilyl)ethynyl)hept-1-en-1-yl)-N-phenylpivalamide (**3q**)

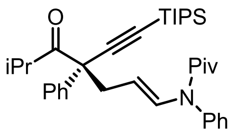

This reaction was performed on 0.1 mmol scale, which provided the product **3o** 22 mg, 40% yield. ^{1}H NMR (400 MHz, Chloroform-*d*) δ 7.3 (m, 9H), 7.0 (d, *J* = 7.8 Hz, 2H), 4.1 (dt, *J* = 14.6, 7.6 Hz, 1H), 3.1 (p, *J* = 6.7 Hz, 1H), 2.8 (dd, *J* = 13.8, 6.5 Hz, 1H), 2.6 (dd, *J* = 13.8, 8.6 Hz, 1H), 1.1 (m, 24H), 1.0 (s, 9H), 0.7 (d, *J* = 6.7 Hz, 3H). ^{13}C NMR (101 MHz, $CDCl_3$) δ 210.2, 175.8, 140.2, 137.2, 134.1, 130.2, 128.9, 128.4, 128.1, 127.4, 127.3, 109.2, 106.0, 89.7, 59.9, 40.8, 40.1, 37.6, 29.1, 21.1, 20.6, 18.7, 11.3. $[\alpha]_D^{23}$ = -25.6 (c 1.0, $CHCl_3$). IR: ν = 3060, 2962, 2941, 2866, 2166, 1717, 1684, 1661, 1649, 1599 cm^{-1}. HRMS-APCI (m/z) calculated for $C_{36}H_{52}NO_2Si^+$ $[M+H]^+$: 558.3762; found: 558.3765. HPLC: Chiralpak IC column, 90:10 hexanes/isopropanol, 1 mL/min; t_R = 5.4 min (major), 6.4min (minor); 99.5:0.5 er.

(*S*,*E*)-N-(4-acetyl-4-phenyl-6-(triisopropylsilyl)hex-1-en-5-yn-1-yl)-N-(p-tolyl)pivalamide (**3r**)

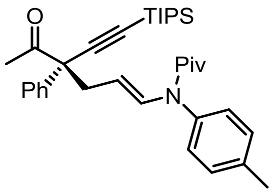

80 mg, 73% yield. ^{1}H NMR (500 MHz, Chloroform-*d*) δ 7.2 (m, 6H), 7.0 (d, *J* = 7.9 Hz, 2H), 6.8 (d, *J* = 7.7 Hz, 2H), 4.0 (m, 1H), 2.7 (dd, *J* = 13.9, 6.6 Hz, 1H), 2.5 (dd, *J* = 13.8, 8.6 Hz, 1H), 2.3 (s, 3H), 2.1 (s, 3H), 1.0 (s, 21H), 0.9 (s, 9H). ^{13}C NMR (126 MHz, $CDCl_3$) δ 202.9, 174.8, 136.8, 136.8, 136.4, 133.1, 128.9, 128.6, 127.6, 126.4, 125.9, 107.7, 105.2, 88.8, 58.9, 39.8, 38.8, 28.1, 25.7, 20.2, 17.6, 10.2. $[\alpha]_D^{23}$ = -18.8 (c 1.0, $CHCl_3$). IR: ν = 3082, 3057, 3030, 2960, 2941, 2895,

2864, 2166, 1721, 1659, 1647, 1609 cm^{-1}. HRMS-APCI (m/z) calculated for $C_{35}H_{50}NO_2Si^+$ $[M+H]^+$: 544.3605; found: 544.3608. HPLC: Chiralpak IC column, 98:02 hexanes/isopropanol, 1 mL/min; t_R = 16.3 min (major), 23.1min (minor); 97:3 er.

(*S,E*)-N-(4-acetyl-4-phenyl-6-(triisopropylsilyl)hex-1-en-5-yn-1-yl)-N-(4-methoxyphenyl)pivalamide (**3s**)

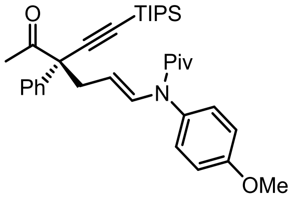

60 mg, 54% yield. ^{1}H NMR (500 MHz, Chloroform-*d*) δ 7.3 (m, 6H), 6.9 (d, *J* = 8.4 Hz, 2H), 6.8 (m, 2H), 4.1 (m, 1H), 3.8 (s, 3H), 2.8 (dd, *J* = 13.9, 6.5 Hz, 1H), 2.6 (dd, *J* = 13.9, 8.6 Hz, 1H), 2.1 (s, 3H), 1.1 (s, 21H), 1.0 (s, 9H). ^{13}C NMR (126 MHz, $CDCl_3$) δ 203.9, 175.9, 159.0, 137.9, 134.3, 132.7, 131.2, 128.6, 127.5, 126.9, 114.0, 108.6, 106.2, 89.9, 60.0, 55.3, 40.8, 39.8, 29.2, 26.7, 18.7, 11.3. $[\alpha]_D^{23}$ = -19.6 (c 1.0, $CHCl_3$). IR: ν = 3080, 3057, 2958, 2941, 2866, 2164, 1717, 1661, 1645, 1595 cm^{-1}. HRMS-APCI (m/z) calculated for $C_{35}H_{50}NO_3Si^+$ $[M+H]^+$: 560.3554; found: 560.3554. HPLC: Chiralpak IC column, 90:10 hexanes/isopropanol, 1 mL/min; t_R = 9.2 min (major), 12.0min (minor); 97:3 er.

(*S,E*)-N-(4-acetyl-4-phenyl-6-(triisopropylsilyl)hex-1-en-5-yn-1-yl)-N-(4-chlorophenyl)pivalamide (**3t**)

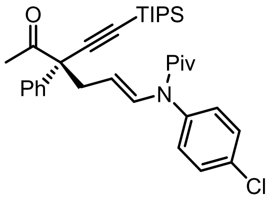

107 mg, 95% yield. ^{1}H NMR (400 MHz, Chloroform-*d*) δ 7.2 (m, 6H), 7.1 (d, *J* = 14.1 Hz, 1H), 6.8 (d, *J* = 8.6 Hz, 2H), 4.1 (m, 1H), 2.7 (dd, *J* = 13.9, 5.8 Hz, 1H), 2.5 (dd, *J* = 13.9, 8.7 Hz, 1H), 2.1 (s, 3H), 1.0 (s, 21H), 1.0 (s, 9H). ^{13}C NMR (101 MHz, $CDCl_3$) δ 202.7, 174.7, 137.9, 136.7, 132.9, 132.8, 130.3, 128.3, 127.6, 126.6, 125.8, 108.3, 105.0, 89.1, 58.8, 39.7, 38.7, 28.0, 25.6, 17.6, 10.2. $[\alpha]_D^{23}$ = -18.8 (c 1.0, $CHCl_3$). IR: ν = 3084, 3057, 2956, 2943, 2922, 2893, 2866, 2164, 1719, 1659, 1645, 1599 cm^{-1}. HRMS-APCI (m/z) calculated for $C_{34}H_{47}ClNO_2Si^+$ $[M+H]^+$: 564.3059; found: 564.3057. HPLC: Chiralpak IC column, 90:10 hexanes/isopropanol, 1 mL/min; t_R = 6.2 min (major), 7.1min (minor); 98:2 er.

(*S*,*E*)-N-(4-acetyl-4-phenyl-6-(triisopropylsilyl)hex-1-en-5-yn-1-yl)-N-(4-(trifluoromethyl)phenyl) pivalamide (**3u**)

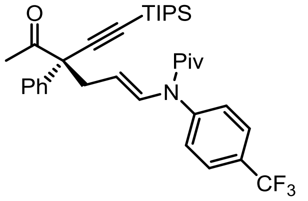

95 mg, 80% yield. ^{1}H NMR (400 MHz, Chloroform-*d*) δ 7.5 (d, *J* = 8.1 Hz, 2H), 7.2 (m, 5H), 7.0 (m, 3H), 4.0 (dt, *J* = 14.7, 8.2 Hz, 1H), 2.7 (dd, *J* = 13.9, 6.7 Hz, 1H), 2.6 (dd, *J* = 13.9, 8.4 Hz, 1H), 2.1 (s, 3H), 1.0 (s, 9H), 1.0 (s, 21H). ^{13}C NMR (126 MHz, $CDCl_3$) δ 203.7, 176.0, 144.0, 137.6, 133.6, 130.2, 130 (d, *J* = 32.8 Hz), 128.7, 127.7, 126.8, 126.2 (q, *J* = 3.7 Hz), 123.8 (d, *J* = 272.3 Hz), 110.0, 105.8, 90.2, 59.8, 40.7, 39.7, 28.8, 26.6, 18.6, 11.2. ^{19}F NMR (376 MHz, Chloroform-*d*) δ -62.5. $[\alpha]_D^{23}$ = -23.6(c 1.0, $CHCl_3$). IR: ν = 3057, 2964, 2945, 2891, 2862, 2166, 1722, 1665, 1647, 1615 cm^{-1}. HRMS-APCI (m/z) calculated for $C_{35}H_{47}F_3NO_2Si^+$ $[M+H]^+$: 598.3323; found: 598.3324. HPLC: Chiralpak IC column, 98:02 hexanes/isopropanol, 1 mL/min; t_R = 7.2 min (major), 8.5min (minor); 98:2 er.

(*S*,*E*)-N-(4-acetyl-4-phenyl-6-(triisopropylsilyl)hex-1-en-5-yn-1-yl)-N-(3-methoxyphenyl)pivalamide (**3v**)

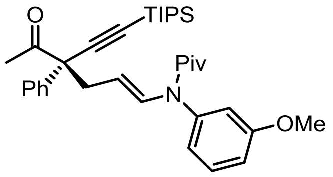

103 mg, 92% yield. ^{1}H NMR (500 MHz, Chloroform-*d*) δ 7.3 (m, 7H), 6.8 (dd, *J* = 8.3, 2.3 Hz, 1H), 6.6 (d, *J* = 7.7 Hz, 1H), 6.5 (s, 1H), 4.2 (dt, *J* = 14.5, 6.9 Hz, 1H), 3.8 (s, 3H), 2.8 (dd, *J* = 13.9, 6.8 Hz, 1H), 2.6 (dd, *J* = 13.9, 8.4 Hz, 1H), 2.1 (s, 3H), 1.1 (s, 21H), 1.0 (s, 9H). ^{13}C NMR (126 MHz, $CDCl_3$) δ 203.9, 175.7, 160.0, 141.2, 137.8, 133.9, 129.5, 128.6, 127.5, 126.9, 122.5, 115.7, 113.9, 108.8, 106.2, 90.0, 60.0, 55.2, 41.0, 39.9, 29.1, 26.7, 18.7, 11.3. $[\alpha]_D^{23}$ = -22.0 (c 1.0, $CHCl_3$). IR: v = 3057, 2962, 2941, 2868, 2162, 1719, 1656, 1641, 1607 cm^{-1}. HRMS-APCI (m/z) calculated for $C_{35}H_{50}NO_3Si^+$ $[M+H]^+$: 560.3554; found: 560.3558. HPLC: Chiralpak IC column, 90:10 hexanes/isopropanol, 1 mL/min; t_R = 8.2 min (major), 8.9min (minor); 95.5:4.5 er.

(*S*,*E*)-N-(4-acetyl-4-phenyl-6-(triisopropylsilyl)hex-1-en-5-yn-1-yl)-N-(3-fluorophenyl)pivalamide (**3w**)

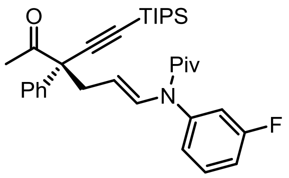

93 mg, 85% yield. ^{1}H NMR (500 MHz, Chloroform-*d*) δ 7.3 (m, 6H), 7.1 (d, *J* = 14.1 Hz, 1H), 7.0 (td, *J* = 8.3, 2.1 Hz, 1H), 6.8 (d, *J* = 7.9 Hz, 1H), 6.7 (d, *J* = 9.3 Hz, 1H), 4.1 (dt, *J* = 14.5, 7.6 Hz, 1H), 2.7 (dd, *J* = 13.9, 6.7 Hz, 1H), 2.6 (m, 1H), 2.1 (s, 3H), 1.1 (s, 21H), 1.0 (s, 9H). ^{13}C NMR (126 MHz, $CDCl_3$) δ 203.8, 175.7, 162.7 (d, *J* = 248.1 Hz), 141.9 (d, *J* = 9.4 Hz), 137.7, 133.7, 130.0 (d, *J* = 9.0 Hz), 128.7, 127.6, 126.8, 125.8 (d, *J* = 3.1 Hz), 117.4 (d, *J* = 21.9 Hz), 115.2 (d, *J* = 20.8 Hz), 109.5, 106.0, 90.1, 59.9, 40.8, 39.8, 28.9, 26.6, 18.7, 11.3. ^{19}F NMR (471 MHz, Chloroform-*d*) δ -111.6. $[\alpha]_D^{23}$ = -22.8 (c 1.0, $CHCl_3$). IR: ν = 3060, 3024, 2958, 2941, 2895, 2866, 2193, 2162,1719, 1666, 1646, 1609, 1596 cm^{-1}. HRMS-APCI (m/z) calculated for $C_{34}H_{47}FNO_2Si^+$ $[M+H]^+$: 548.3355; found: 548.3360. HPLC: Chiralpak IC column, 90:10 hexanes/isopropanol, 1 mL/min; t_R = 5.7 min (major), 6.8min (minor); 97.5:2.5 er.

(*S,E*)-N-(4-acetyl-4-phenyl-6-(triisopropylsilyl)hex-1-en-5-yn-1-yl)-N-(naphthalen-2-yl)pivalamide (**3x**)

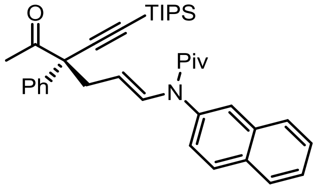

87 mg, 75% yield. ^{1}H NMR (400 MHz, Chloroform-*d*) δ 7.8 (d, *J* = 9.0 Hz, 1H), 7.8 (d, *J* = 8.1 Hz, 2H), 7.5 (m, 3H), 7.3 (m, 6H), 7.0 (d, *J* = 8.0 Hz, 1H), 4.2 (m, 1H), 2.8 (dd, *J* = 13.2, 5.8 Hz, 1H), 2.6 (dd, *J* = 13.9, 8.7 Hz, 1H), 2.1 (s, 3H), 1.0 (s, 9H), 0.9 (m, 21H). ^{13}C NMR (101 MHz, $CDCl_3$) δ 203.8, 176.1, 137.7, 137.5, 134.1, 133.3, 132.7, 128.9, 128.7, 128.6, 128.2, 128.0, 127.7, 127.5, 126.9, 126.7, 126.4, 109.5, 106.0, 90.0, 59.9, 40.9, 39.9, 29.2, 26.7, 18.5, 11.1. $[\alpha]_D^{23}$ = 1.4 (c 1.0, $CHCl_3$). IR: ν = 3057, 2960, 2924, 2862, 2166, 1721, 1661, 1649, 1599 cm^{-1}. HRMS-APCI (m/z) calculated for $C_{38}H_{50}NO_2Si^+$ $[M+H]^+$: 580.3605; found: 580.3607. HPLC: Chiralpak IC column, 90:10 hexanes/isopropanol, 1 mL/min; t_R =11.0 min (major), 12.0 min (minor); 97.5:2.5 er.

(*S,E*)-N-(4-acetyl-4-phenyl-6-(triisopropylsilyl)hex-1-en-5-yn-1-yl)-N-phenylisobutyramide (**3y**)

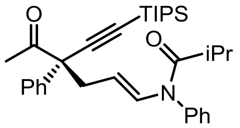

72 mg, 70% yield. ^{1}H NMR (500 MHz, Chloroform-*d*) δ 7.3 (m, 9H), 7.0 (s, 2H), 4.2 (m, 1H), 2.8 (dd, *J* = 13.9, 6.8 Hz, 1H), 2.6 (dd, *J* = 13.9, 8.3 Hz, 1H), 2.3 (m, 1H), 2.1 (s, 3H), 1.1 (s, 21H), 1.0 (m, 6H). ^{13}C NMR (126 MHz, $CDCl_3$) δ 203.9, 174.9, 139.5, 137.8, 131.4, 129.6, 128.8, 128.6, 128.3, 127.5, 126.8, 109.5, 106.1, 90.1, 60.0, 39.7, 31.8, 26.7, 19.6, 19.5, 18.7, 11.2. $[\alpha]_D^{23}$ = -14.2 (c 1.0, $CHCl_3$). IR: ν = 3064, 2964, 2943, 2891, 2864, 2164, 1717, 1676, 1653, 1597 cm^{-1}. HRMS-APCI (m/z) calculated for $C_{33}H_{46}NO_2Si^+$ $[M+H]^+$: 516.3292; found: 516.3296 . HPLC: Chiralpak IC column, 90:10 hexanes/isopropanol, 1 mL/min; t_R =7.4 min (major), 8.2 min (minor); 97:3 er.

(*S,E*)-N-(4-acetyl-4-phenyl-6-(triisopropylsilyl)hex-1-en-5-yn-1-yl)-N-phenylacetamide (**3z**)

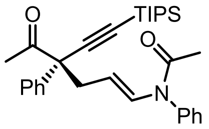

69 mg, 71% yield. ^{1}H NMR (500 MHz, Chloroform-*d*) δ 7.3 (m, 9H), 7.0 (d, *J* = 7.5 Hz, 2H), 4.2 (dt, *J* = 15.0, 7.8 Hz, 1H), 2.8 (dd, *J* = 13.9, 6.6 Hz, 1H), 2.6 (dd, *J* = 13.9, 8.6 Hz, 1H), 2.1 (s, 3H), 1.7 (s, 3H), 1.0 (s, 21H). ^{13}C NMR (126 MHz, $CDCl_3$) δ 203.8, 168.1, 139.9, 137.7, 130.8, 129.7, 128.7, 128.6, 128.4, 127.6, 126.8, 109.2, 106.0, 90.1, 59.9, 39.6, 26.7, 23.1, 18.7, 11.2. $[\alpha]_D^{23}$ = -30.0 (c 1.0, $CHCl_3$). IR: ν = 3051, 2964, 2943, 2929, 2891, 2866, 2166, 1719, 1678, 1659, 1599 cm^{-1}. HRMS-APCI (m/z) calculated for $C_{31}H_{42}NO_2Si^+$ $[M+H]^+$: 488.2979; found: 488.2981. HPLC: Chiralpak IC column, 80:20 hexanes/isopropanol, 1 mL/min; t_R =8.2min (major), 10.8min (minor); 92:8 er.

5. Mechanistic studies

Control experiments

Substrates **4b**[5] and **4c**[6] were prepared according to literatures.

N-((1E,5E)-4-acetyl-4,6-diphenylhexa-1,5-dien-1-yl)-N-phenylpivalamide

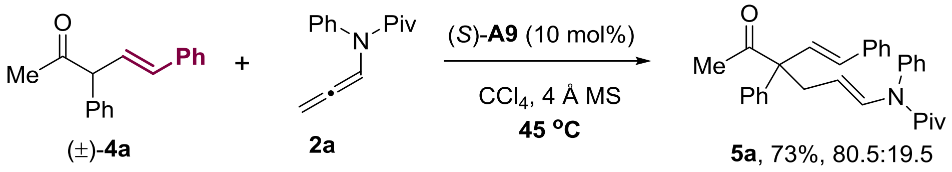

To a dry sealed tube charged with 4Å molecular sieves (50 mg), allenamide **2a** (0.11 mmol) and CPA (*S*)-**A9** was added (*E*)-3,5-diphenylpent-4-en-2-one **4a** (24 mg, 0.1 mmol) and CCl_4 (1 mL). After stirring at 45 ℃ for 36 h, the mixture was cooled to rt and filtered through Celite. The

filtrate was concentrated under vacuum to give a residue, which was purified by column chromatography on silica gel (petroleum ether:DCM = 1:1) to afford the products **5a** (33 mg, 73% yield). ^{1}H NMR (500 MHz, Chloroform-*d*) δ 7.3 (m, 8H), 7.2 (m, 4H), 7.1 (d, *J* = 7.7 Hz, 2H), 7.0 (d, *J* = 7.3 Hz, 2H), 6.6 (d, *J* = 16.5 Hz, 1H), 6.2 (d, *J* = 16.5 Hz, 1H), 4.0 (dt, *J* = 14.6, 7.6 Hz, 1H), 2.8 (m, 2H), 2.0 (s, 3H), 1.0 (s, 9H). ^{13}C NMR (126 MHz, $CDCl_3$) δ 207.3, 175.9, 141.1, 140.2, 136.9, 133.8, 132.6, 130.5, 130.3, 129.0, 128.6, 128.6, 128.2, 127.8, 127.7, 127.1, 126.3, 109.7, 63.3, 40.9, 37.9, 29.1, 27.3. $[\alpha]_D^{23}$ = 14.8 (c 1.0, $CHCl_3$). IR: ν = 3030, 2989, 2974, 2902, 1709, 1655, 1636, 1590 cm^{-1}. HRMS-ESI (m/z) calculated for $C_{31}H_{34}NO_2^+$ $[M+H]^+$: 452.2584; found: 452.2586. HPLC: Chiralpak IC column, 90:10 hexanes/isopropanol, 1 mL/min; t_R = 12.2 min (minor), 14.6 min (major); 80.5:19.5 er.

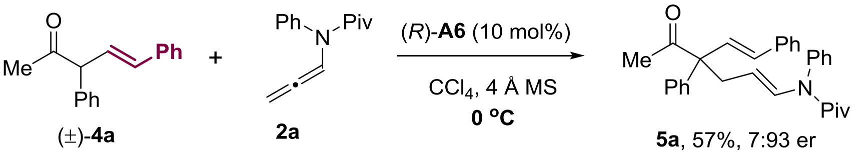

The same procedure was applied, except CPA (*R*)-**A6** and 0 °C was used, which provided **5a** in 57% yield with 7:93 er.

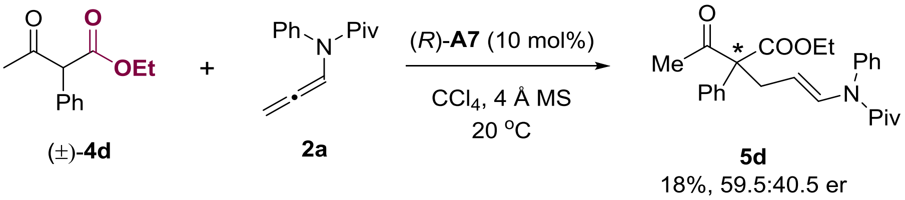

The same procedure was applied, except ketone **4d** was used instead, which provided **5d** in 18% yield with 59.5:40.5 er.

Date for **5d**: ^{1}H NMR (400 MHz, Chloroform-*d*) δ 7.42 – 7.32 (m, 4H), 7.29 – 7.24 (m, 2H), 7.16 (dd, *J* = 8.1, 1.6 Hz, 2H), 7.09 – 7.03 (m, 2H), 4.29 – 4.17 (m, 2H), 4.11 (ddd, *J* = 14.2, 8.1, 7.2 Hz, 1H), 3.01 (ddd, *J* = 14.0, 7.2, 1.3 Hz, 1H), 2.69 (ddd, *J* = 14.0, 8.1, 1.1 Hz, 1H), 2.02 (s, 3H), 1.23 (t, *J* = 7.1 Hz, 3H), 1.04 (s, 9H). ^{13}C NMR (101 MHz, Chloroform-*d*) δ 203.3, 170.6, 140.0, 137.2, 134.5, 130.4, 129.2, 128.6, 128.5, 127.9, 127.7, 108.6, 69.5, 61.6, 41.1, 36.7, 29.2, 27.9, 14.1. HRMS-ESI (m/z) calculated for $C_{26}H_{32}NO_4^+$ $[M+H]^+$: 422.2326; found: 422.2329. HPLC: Chiralpak IC column, 90:10 hexanes/isopropanol, 1 mL/min; t_R = 20.1 min (major), 21.9 min (minor); 59.5:40.5 er.

Kinetic experiments

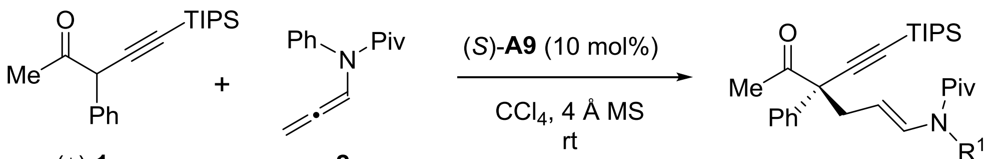

Determination of the order of CPA A9

To four solutions of allenamide **2a** (7 mg, 0.0325 mmol), ketone **1a** (13.3 mg, 0.0424 mmol) and 4 Å MS (10 mg) in *d*-benzene (0.53 mL) was added CPA **A9** 1.25 mg (1.66 μmol), 2.5 mg (3.32 μmol), 3.75 mg (4.98 μmol), 5 mg (6.64 μmol), respectively. The yield of **3a** was determined by ^{1}H NMR analysis of the reaction mixture.

Supplementary Table 1. Kinetic Profile of CPA A9 (5 mol%).

With CPA **A9** (1.25 mg, 1.66 μmol)

Time (min)	62	93	121	154	181	211
yield	2.75%	3.73%	4.67%	5.91%	6.58%	7.78%
C (**3a**) mM	2.20	2.98	3.73	4.72	5.26	6.22

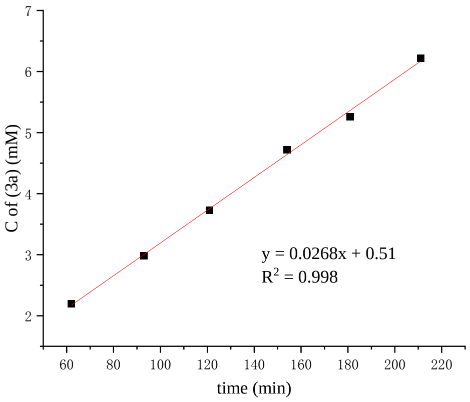

v_0 = 0.0268 mM/min

Supplementary Table 2. Kinetic Profile of CPA A9 (10 mol%).

With CPA **A9** (2.5 mg, 3.32 μmol)

Time (min)	95	157	184	214
yield	5.55%	8.83%	10.22%	11.97%
C (**3a**)	4.43	7.06	8.17	9.56

mM				

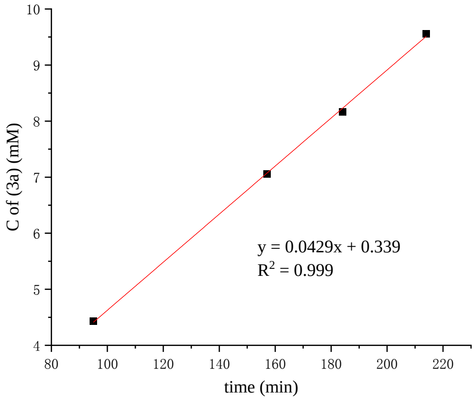

v_0 = 0.0429 mM/min

Supplementary Table 3. Kinetic Profile of CPA A9 (15 mol%).

With CPA **A9** (3.75 mg, 4.98 μmol)

Time (min)	16	45	78	105	135
yield	1.93%	3.69%	6.67%	8.81%	11.42%
C (**3a**) mM	1.54	2.95	5.33	7.04	9.12

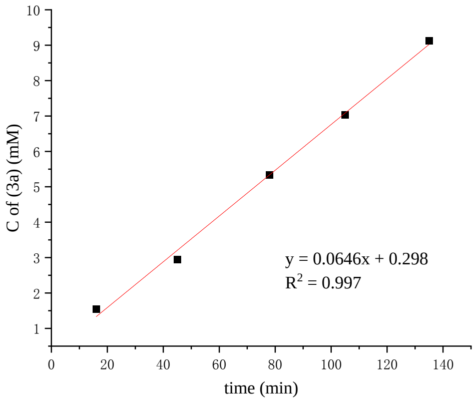

v_0 = 0.0646 mM/min

Supplementary Table 4. Kinetic Profile of CPA A9 (20 mol%).

With CPA **A9** (5 mg, 6.64 μmol)

Time (min)	38	63	93	122	141	181
yield	4.90%	8.01%	11.57%	15.08%	17.06%	22.11%
C (**3a**) mM	3.92	6.40	9.24	12.05	13.63	17.67

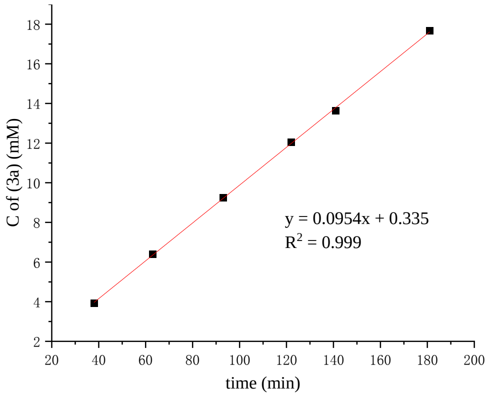

v_0 = 0.0954 mM/min

Supplementary Table5. The relationship between v_0(3a) and C(CPA).

The relationship between v_0(**3a**) and C(CPA)

C of CPA (mM)	3.14	6.27	9.41	12.54
V_0 of **3a** (mM/min)	0.0268	0.0429	0.0646	0.0954

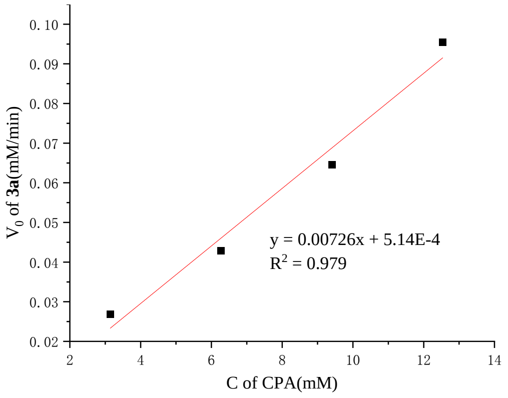

Determination of the order of ketone 1a

To four solutions of allenamide **2a** (14 mg, 0.065 mmol), CPA (*S*)-**A9** (5 mg, 0.0067 mmol), 4 Å MS (10 mg) in *d*-benzene (0.53 mL) was added ketone **1a** 6.7 mg (0.021 mmol), 13.3 mg (0.042 mmol), 20.0 mg (0.064 mmol), 26.6 mg (0.085 mmol), respectively. The yield of **3a** was determined by ^{1}H NMR analysis of the reaction mixture.

Supplementary Table 6. Kinetic Profile of 1a-1.

With **1a** (6.7 mg, 0.021 mmol)

Time (min)	28	40	53	62	74

yield	2.11%	3.01%	3.80%	4.55%	5.46%
C of **3a** (mM)	0.836	1.193	1.506	1.803	2.163

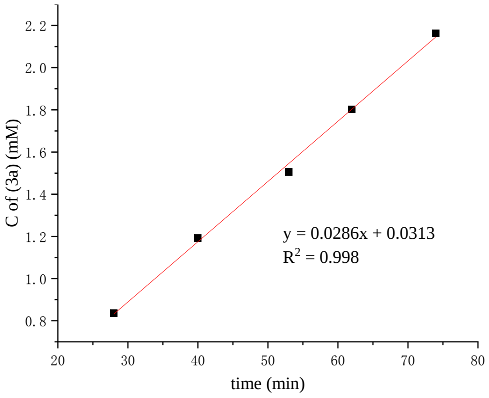

v_0 = 0.0286 mM/min

Supplementary Table 7. Kinetic Profile of 1a-2.

With **1a** (13.3 mg, 0.042 mmol)

Time (min)	12	27	39	51	64
yield	2.65%	3.73%	4.69%	5.87%	7.07%
C of **3a** (mM)	2.13	2.99	3.76	4.71	5.67

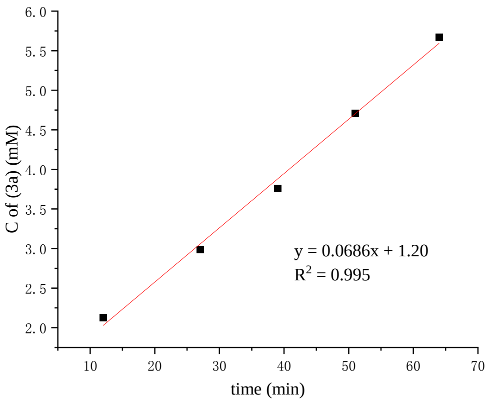

v_0 = 0.0686 mM/min

Supplementary Table 8. Kinetic Profile of 1a-3.

With **1a** (20.0 mg, 0.064 mmol)

Time (min)	30	42	54	63	75
yield	2.22%	2.99%	4.06%	4.84%	5.86%
C of **3a** (mM)	2.68	3.61	4.90	5.84	7.08

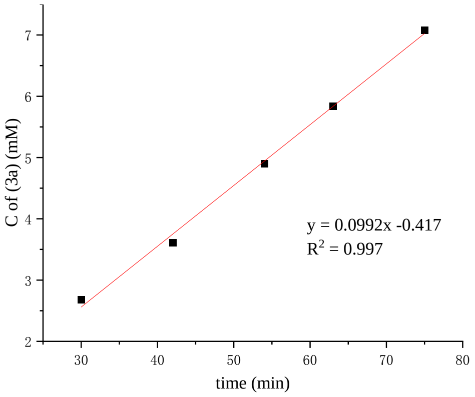

v_0 = 0.0997 mM/min

Supplementary Table 9. Kinetic Profile of 1a-4.

With **1a** (26.6 mg, 0.085 mmol)

Time (s)	13	64	72	85
yield	1.51%	5.67%	6.55%	7.78%
C of **3a** (mM)	2.42	9.09	10.50	12.48

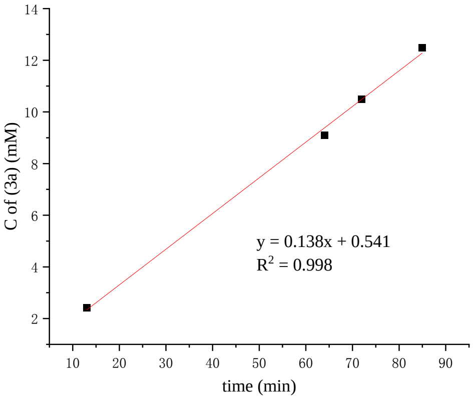

v_0 = 0.138 mM/min

Supplementary Table 10. Relationship between C_0(1a) and v_0(3a)

C_0 (ketone)/M	0.0398	0.0796	0.119	0.159
v_0/mM·min^{-1}	0.0286	0.0686	0.0997	0.138

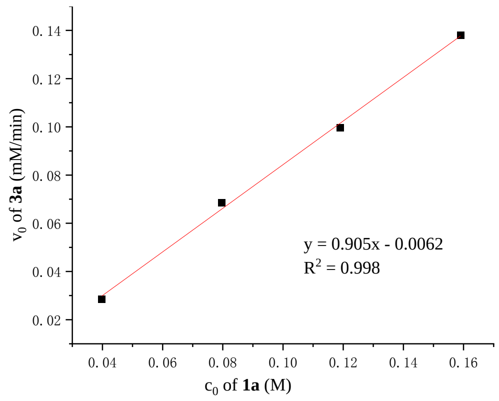

Determination of the order of allenamide 2a

To four solutions of ketone **1a** (13.3 mg, 0.042 mmol), CPA **A9** (5.0 mg, 6.58μmol, 15.7 mmol%) and 4 Å MS (10 mg) in *d*-benzene (0.53 mL) was added allenamide **2a** 7 mg (0.0325 mmol), 14 mg (0.065 mmol), 21 mg (0.098 mmol), 28 mg (0.13 mmol), respectively. The yield of **3a** was determined by ^{1}H NMR analysis of the reaction mixture.

Supplementary Table 11. Kinetic Profile of 2a-1.

With allenamide **2a** (7 mg, 0.0325 mmol)

Time (min)	16	26	34	43	52	60
yield	2.91%	3.83%	4.77%	5.95%	7.18%	8.49%
C of **3a** (mM)	2.33	3.07	3.83	4.77	5.76	6.81

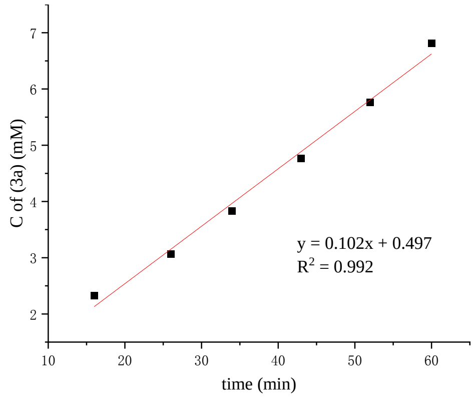

v_0 = 0.102 mM/min

Supplementary Table 12. Kinetic Profile of 2a-2.

With allenamide **2a** (14 mg, 0.065 mmol)

Time (min)	12	27	39	51	64

yield	2.65%	3.73%	4.69%	5.87%	7.07%
C of **3a** (mM)	2.13	2.99	3.76	4.71	5.67

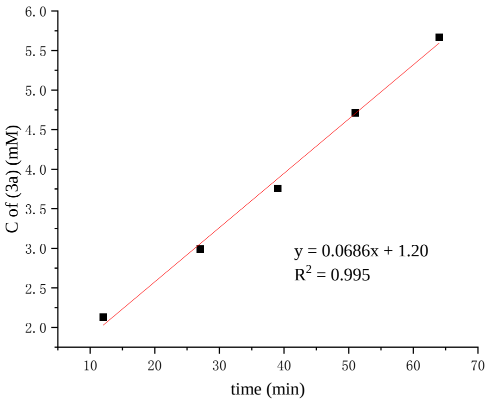

v_0 = 0.0686 mM/min

Supplementary Table 13. Kinetic Profile of 2a-3.

With allenamide **2a** (21 mg, 0.0975 mmol)

Time (s)	26	34	43	52	60	69
yield	3.03%	3.56%	4.20%	5.02%	5.75%	6.41%
C of **3a** (mM)	2.43	2.85	3.37	4.03	4.61	5.14

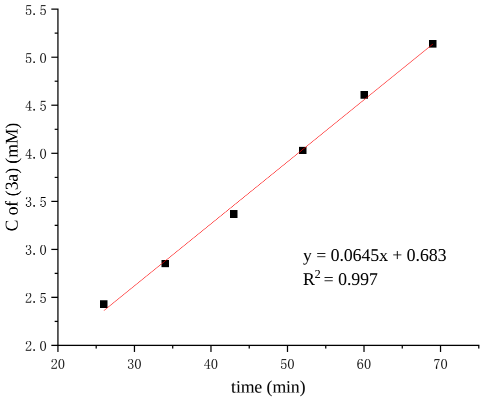

v_0 = 0.0645 mM/min

Supplementary Table 14. Kinetic Profile of 2a-4.

With allenamide **2a** (28 mg, 0.13 mmol)

Time (s)	21	27	37	45	54	63
yield	2.35%	2.72%	3.33%	3.90%	4.63%	5.13%
C of **3a** (mM)	1.88	2.18	2.67	3.13	3.71	4.11

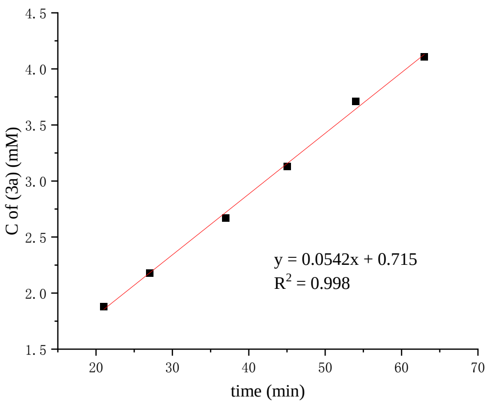

v_0 = 0.0542 mM/min

Supplementary Table 15. Relationship between C_0(2a) and v_0(3a)

C_0 (allenamide **2a**)/M	0.0613	0.123	0.184	0.245
V_0/ mM·min^{-1}	0.102	0.0686	0.0645	0.0542

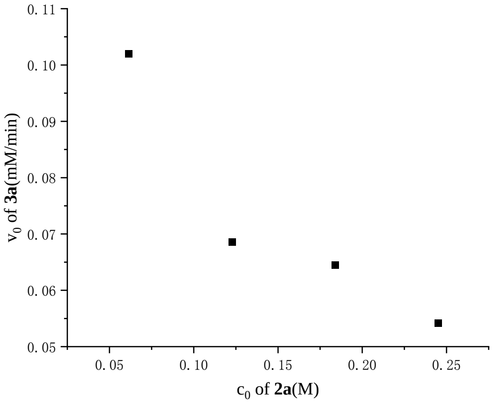

Relationship between v_0(3a) and C(amide additive 2a')

To four solutions of ketone **1a** (13.3 mg, 0.042 mmol), CPA **A9** (5.0 mg, 6.58μmol), **2a** (7 mg, 0.0325 mmol) and 4 Å MS (10 mg) in *d*-benzene (0.53 mL) was added amide **2a'** (7 mg, 0.0325 mmol), 14 mg (0.065 mmol), 21 mg (0.098 mmol), respectively. The yield of **3a** was determined by ^{1}H NMR analysis of the reaction mixture.

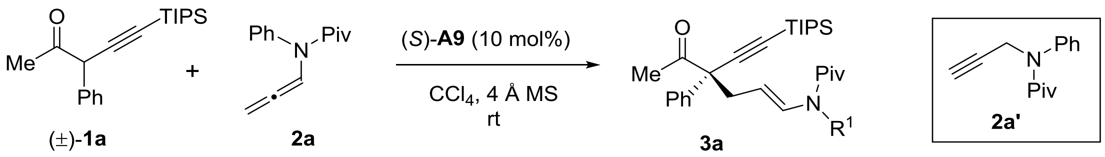

Supplementary Table 16. Kinetic Profile of 2a'-1.

Without adding amide **2a'**

Time (s)	6	20	33	59	71
yield	9.79%	10.72%	11.6%	14.14%	15.23%
C of **3a** (mM)	7.85	8.60	9.30	11.34	12.21

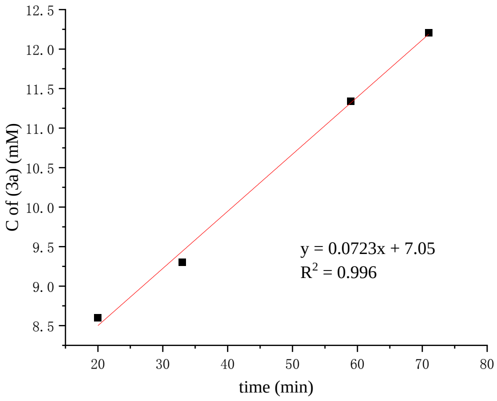

v_0 = 0.0723 mM/min

Supplementary Table 17. Kinetic Profile of 2a'-2.

With **2a'** (7 mg, 0.0325 mmol)

Time (s)	4	17	40	53	65
yield	6.52%	7.79%	8.84%	9.78%	10.89%
C of **3a** (mM)	5.23	6.25	7.09	7.84	8.73

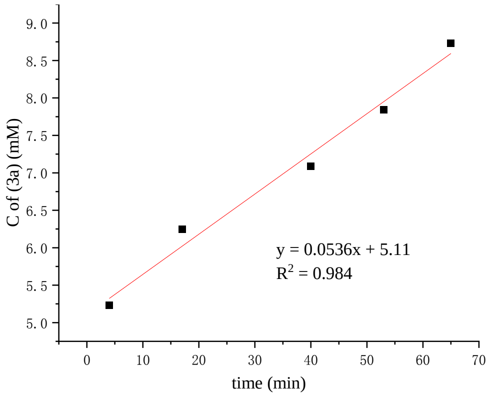

v_0 = 0.0536 mM/min

Supplementary Table 18. Kinetic Profile of 2a'-3.

With **2a'** (14 mg, 0.065 mmol)

Time (min)	17	43	56	68
yield	7.20%	8.49%	9.27%	10.08%
C of **3a** (mM)	5.77	6.81	7.43	8.08

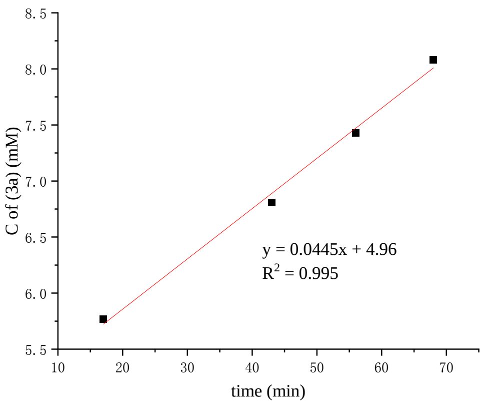

v_0 = 0.0445 mM/min

Supplementary Table 19. Kinetic Profile of 2a'-4.

With **2a'** (21 mg, 0.065 mmol)

Time (s)	17	47	59	72
yield	7.25%	8.62%	9.03%	9.44%
C of **3a** (mM)	5.81	6.91	7.24	7.57

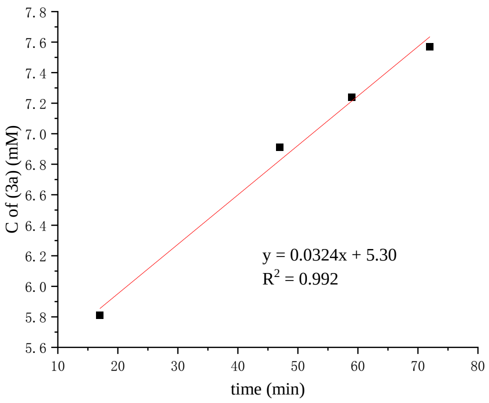

v_0 = 0.0324 mM/min

Supplementary Table 20. Relationship between v_0(3a) and C(amide additive 2a')

C_0 (amide Sx)/M	0	0.0613	0.123	0.184
V_0/ mM·S^{-1}	0.0723	0.0536	0.0445	0.0324

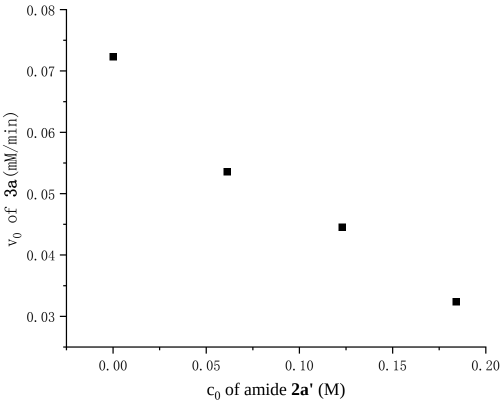

KIE experiment

α-H deuterated substrate **1a**$_{\mathbf{D}}$ was prepared from deuterated benzaldehyde PhCOD using the same general procedure for the synthesis of ketone **1**. ^{1}H NMR (400 MHz, Chloroform-*d*) δ 7.52 – 7.44 (m, 2H), 7.41 – 7.35 (m, 2H), 7.34 – 7.29 (m, 1H), 2.23 (s, 3H), 1.12 (s, 21H). ^{13}C NMR (101 MHz, Chloroform-*d*) δ 202.9, 134.7, 129.0, 128.0, 128.0, 102.9, 89.1, 25.8, 18.8, 11.4.

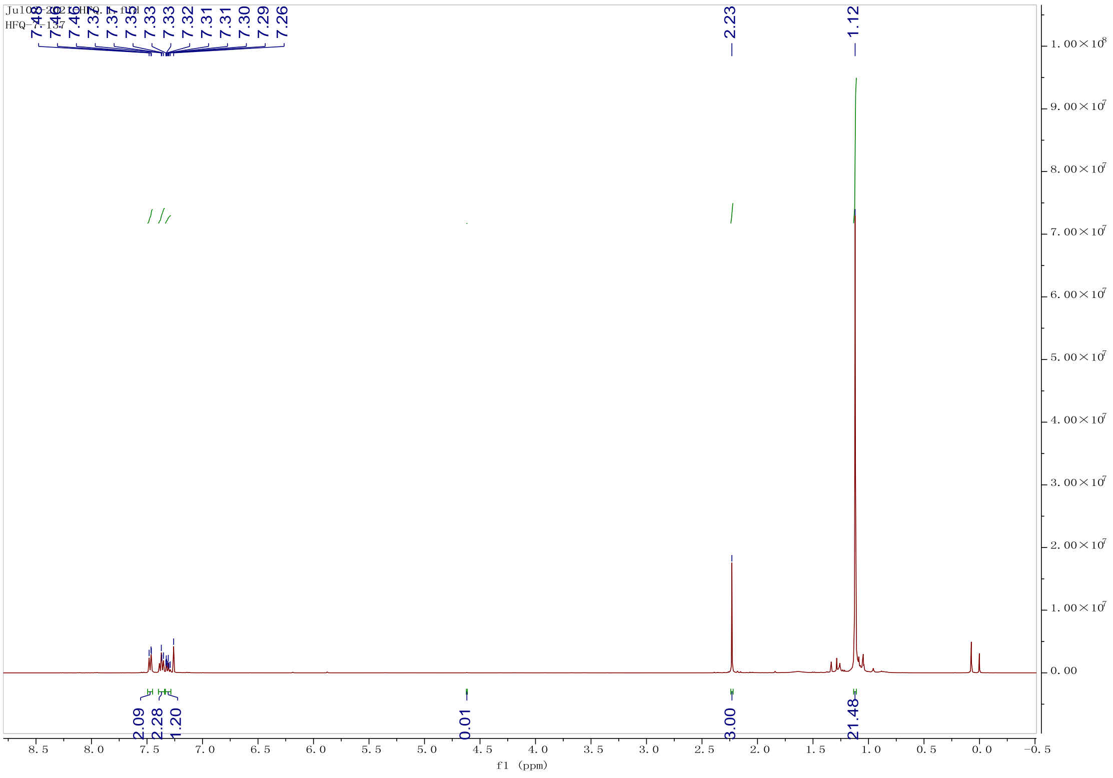

Supplementary Fig. 1 ^{1}H NMR spectrum of **1a**$_{\mathbf{D}}$

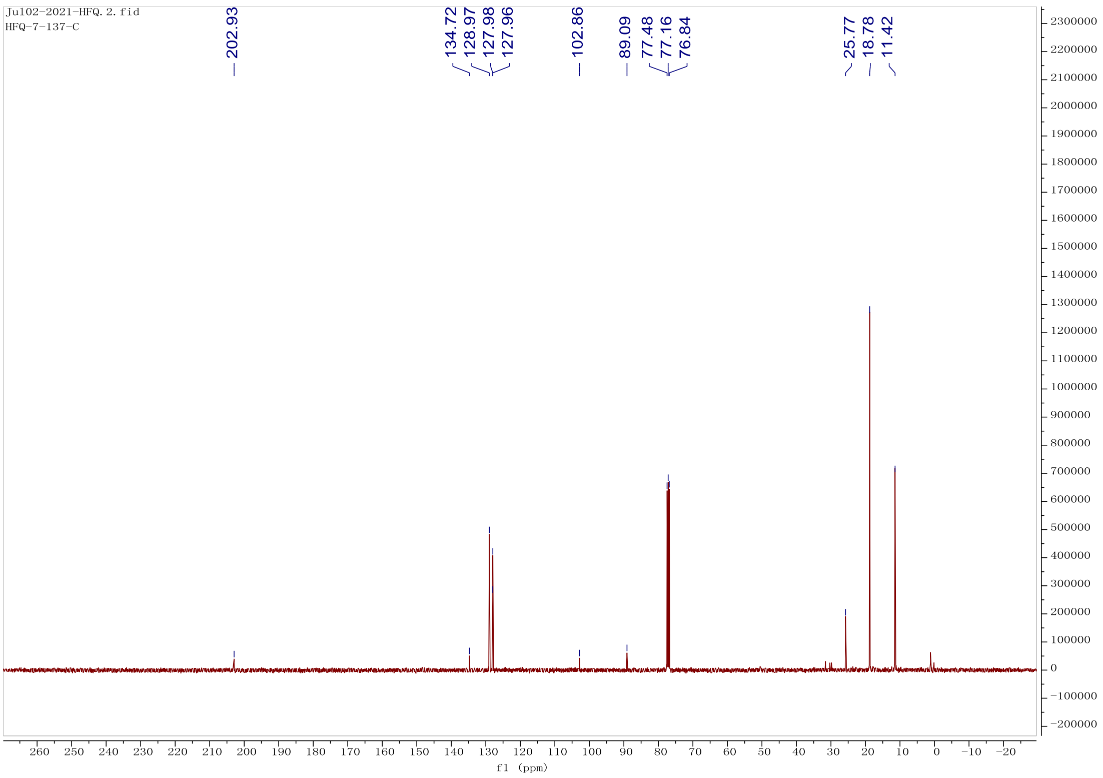

Supplementary Fig. 2 ^{13}C NMR spectrum of $\mathbf{1a_D}$

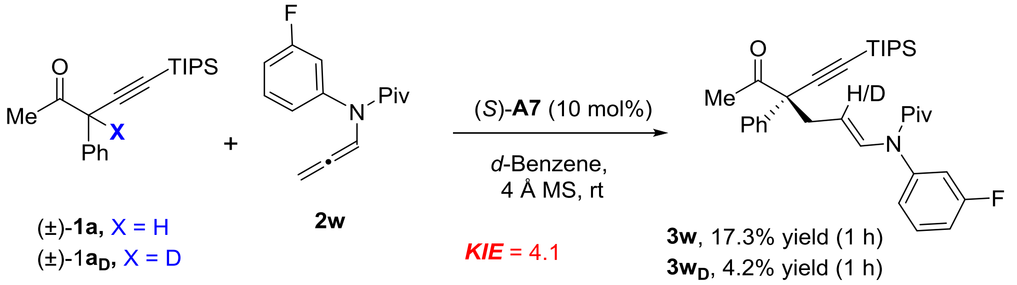

The reaction of **1a** (0.1 mmol) and $\mathbf{1a_D}$ with allenamide **2w** were performed in the presence of (*S*)-**A7** (10 mol%) in D6-benzene at 20 °C, respectively. The yields of **3w** and $\mathbf{3w_D}$ were determined to be 17.3% and 4.2% respectively by ^{1}H NMR after 1 h.

The KIE was calculated as: KIE = 17.3%/4.2% = 4.1.

Isomerization of 1a to 1a'

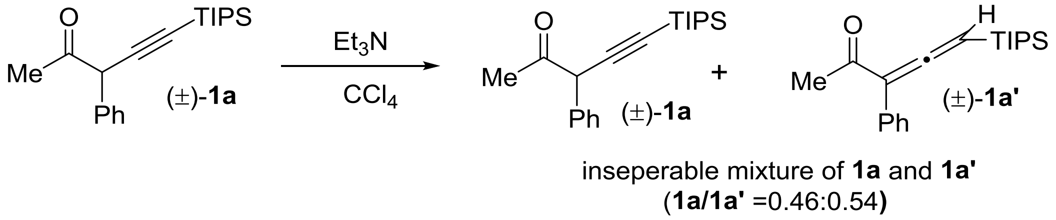

To a solution of **1a** (500 mg) in CCl_4 (2 ml) was added Et_3N (50 ul, 0.2 eq) at rt. After stirring at rt for 20 h, the mixture was concentrated under vacuum to give a mixture of **1a** and **1a'** (**1a/1a'** = 0.46:0.54), which can't be separated by column chromatography. Characteristic ^{1}H NMR signals for **1a'**: ^{1}H NMR (500 MHz, Chloroform-*d)* δ 5.73 (s, 1H), 2.47 (3H). ^{13}C NMR (126 MHz, Chloroform-*d*) δ 215.7, 196.6, 131.5, 127.1, 126.8, 126.0, 104.3, 82.4, 27.3, 17.4, 17.4, 10.7.

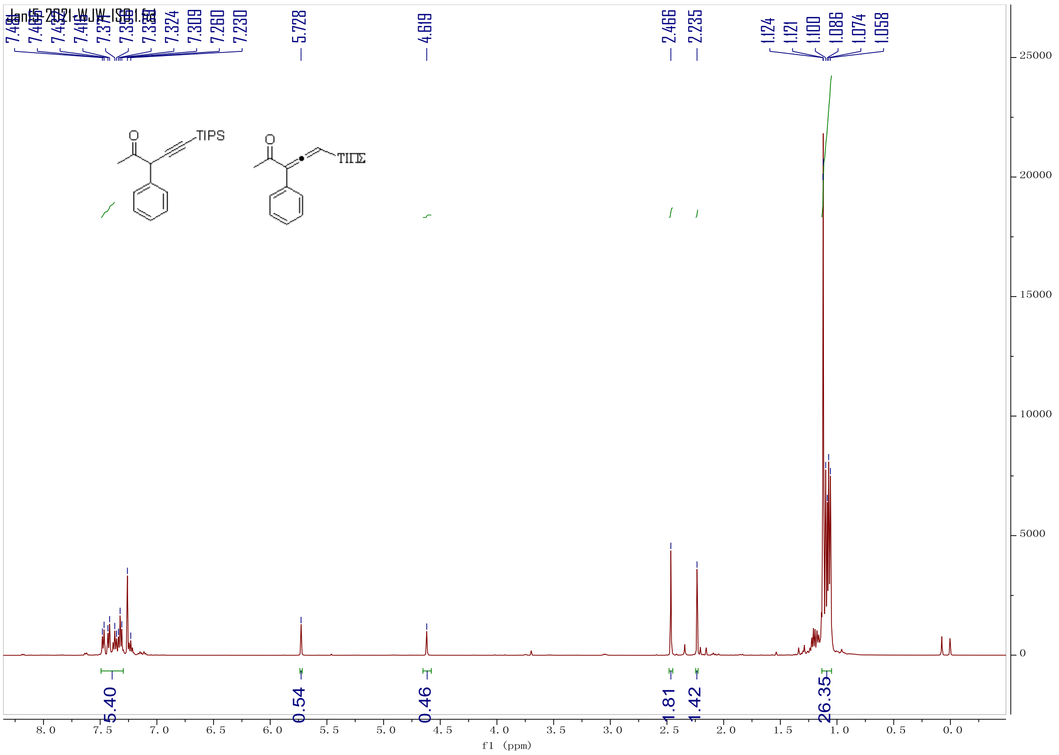

Supplementary Fig. 3 ^{1}H NMR of Mixture of **1a** and 3-phenyl-5-(triisopropylsilyl)penta-3,4-dien-2-one (**1a'**)

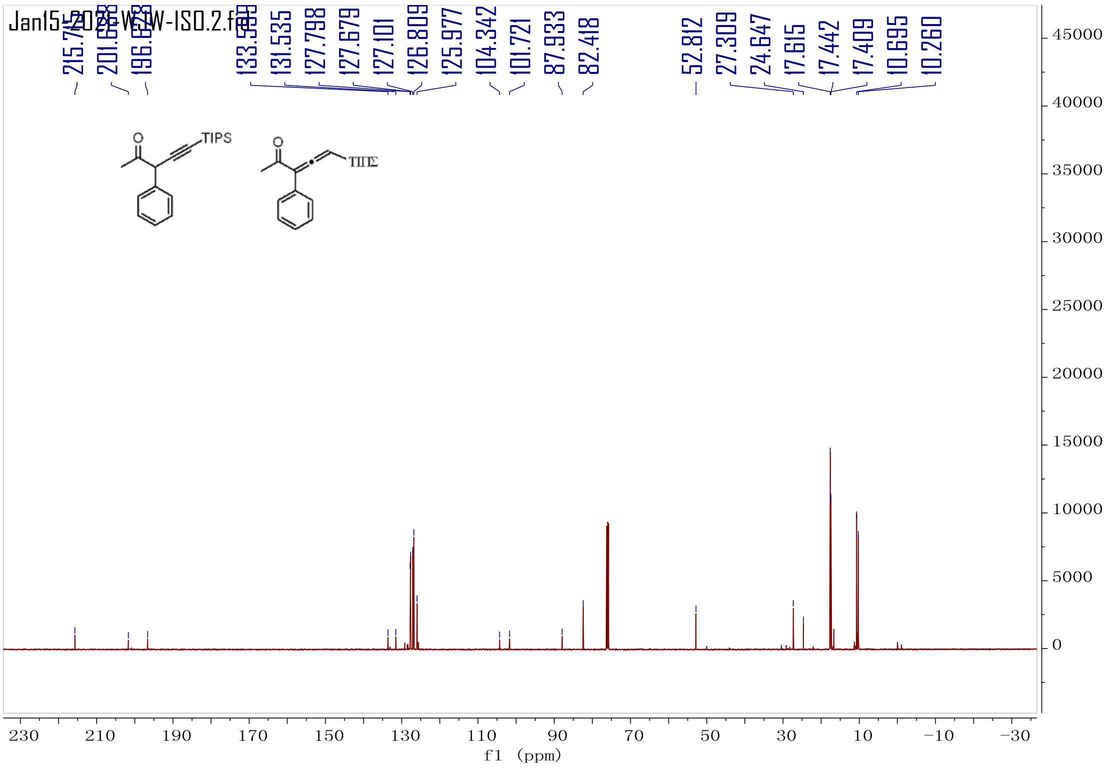

Supplementary Fig. 4 ^{13}C NMR of Mixture of **1a** and 3-phenyl-5-(triisopropylsilyl)penta-3,4-dien-2-one (**1a'**)

Non-linear effect experiments

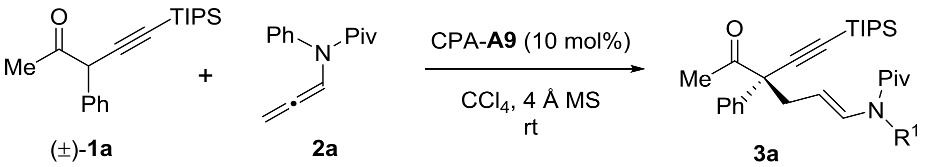

A solution of CPA **A9** (with different ees) in CCl_4 (0.5 mL) was prepared by mixing the solution of (*R*)-**A9** and (*S*)-**A9** in CCl_4.

To a dry sealed tube charged with 4Å molecular sieves (100 mg), allenamide **2a** (0.1 mmol) was added ketone **1a** (0.1 mmol) in CCl_4 (0.5 mL) at rt, which was followed by adding the solution of CPA **A9** (with different ees) in CCl_4 (0.5 mL). After stirring at the room temperature for 20 h, the mixture was filtered through Celite and the filtrate was concentrated under vacuum to give a residue, which was purified by column chromatography on silica gel (petroleum ether:EtOAc, 30:1 ~ 10:1) to give the products **3a**.

Supplementary Table 21. Non-linear effect experiment between 3a and CPA A9.

Ee of CPA **A9** (% ee)	0	20	40	60	80	100
Ee of **3a** (% ee)	0	18.3	37.8	54.3	74.3	94

Characterization of the reactive intermediate INT-A:

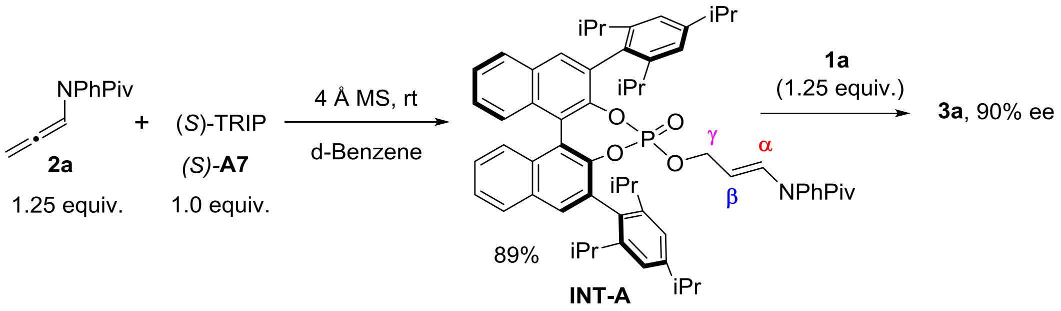

To a solution of (*S*)-**A7** (18 mg, 1.0 eq.) in d-Benzene (0.5 mL) was added allenamide **2a** (6 mg, 1.25 eq.) at rt. After mixing for 30 min, a series of NMR spectroscopy and mass spectroscopy were performed before adding **1a** (9 mg, 1.25 eq.). After reacting for 20 h, the mixture was concentrated to give a residue, which was purified by column chromatography on silica gel (petroleum ether:EtOAc = 10:1) to afford the products **3a** (12 mg, 80%, 90% ee).

Characteristic ^{1}H NMR signals of **INT-A**: ^{1}H NMR (500 MHz, Benzene-d_6) δ 7.65 (d, *J* = 14.1 Hz, 1H, $\mathbf{H_{\alpha}}$) 4.22 (m, 1H, $\mathbf{H_{\gamma}}$), 4.04 (dt, *J* = 14.5, 7.5 Hz, 1H, $\mathbf{H_{\beta}}$), 3.69 (m, 1H, $\mathbf{H_{\gamma}}$). ^{1}H NMR signals of **acrolein**: ^{1}H NMR (500 MHz, Benzene-d_6) δ 9.12 (d, *J* = 7.7 Hz, 0.37 H), 5.93 (ddd, *J* = 17.5, 10.0, 7.7 Hz, 0.37 H), 5.51 (d, *J* = 10.0 Hz, 0.37 H), 5.33 (d, J = 17.3 Hz, 0.37 H). Characteristic ^{13}C NMR signals of **INT-A**: ^{13}C NMR (126 MHz, Benzene-d_6) δ 137.8 ($\mathbf{C_{\alpha}}$), 105.8 (d, *J* = 6.0 Hz, $\mathbf{C_{\beta}}$), 68.9 (d, *J* = 4.9 Hz, $\mathbf{C_{\gamma}}$). ^{31}P NMR (202 MHz, Benzene-d_6) δ 1.71 (t, *J* = 9.1 Hz, without H-P decoupling). m/z HRMS (ESI) found $[M+H]^+$ 986.5386. $C_{64}H_{75}NO_5P^+$ requires 968.5377.

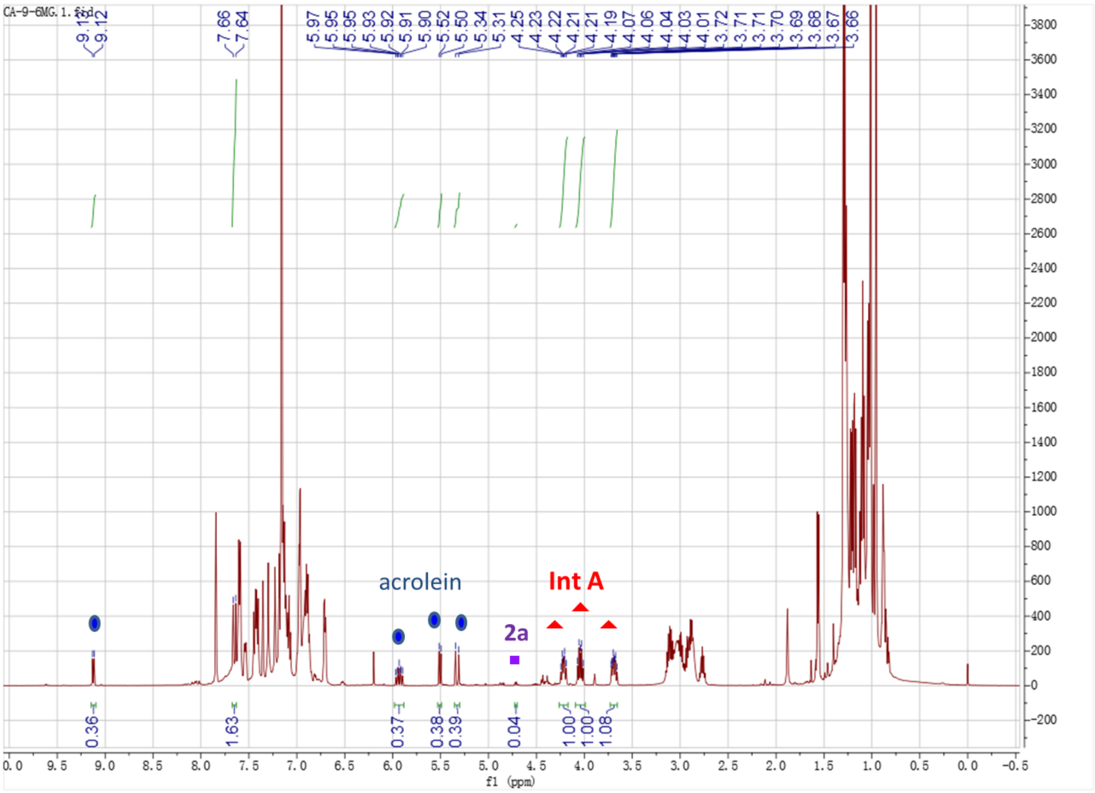

Supplementary Fig. 5 ^{1}H NMR of the mixture of INT A.

^{13}C NMR

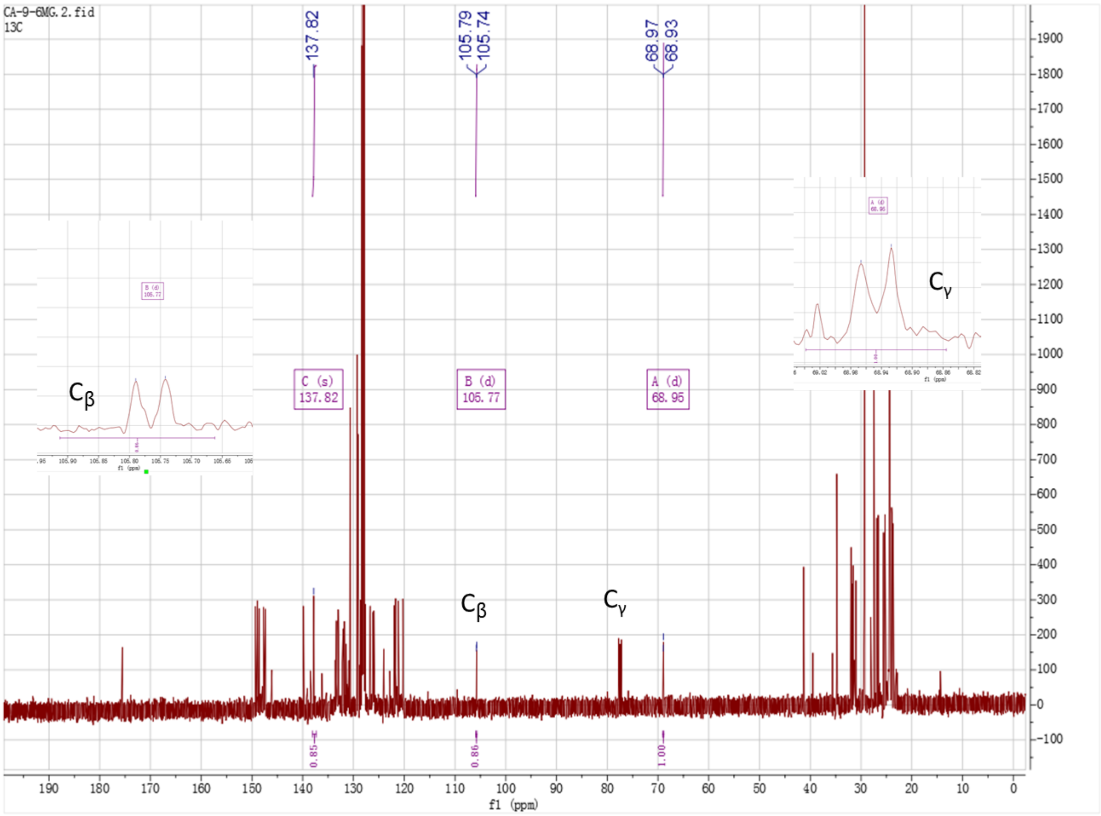

Supplementary Fig. 6 ^{13}C NMR of the mixture of INT A.

^{31}P NMR (with H-P coupling)

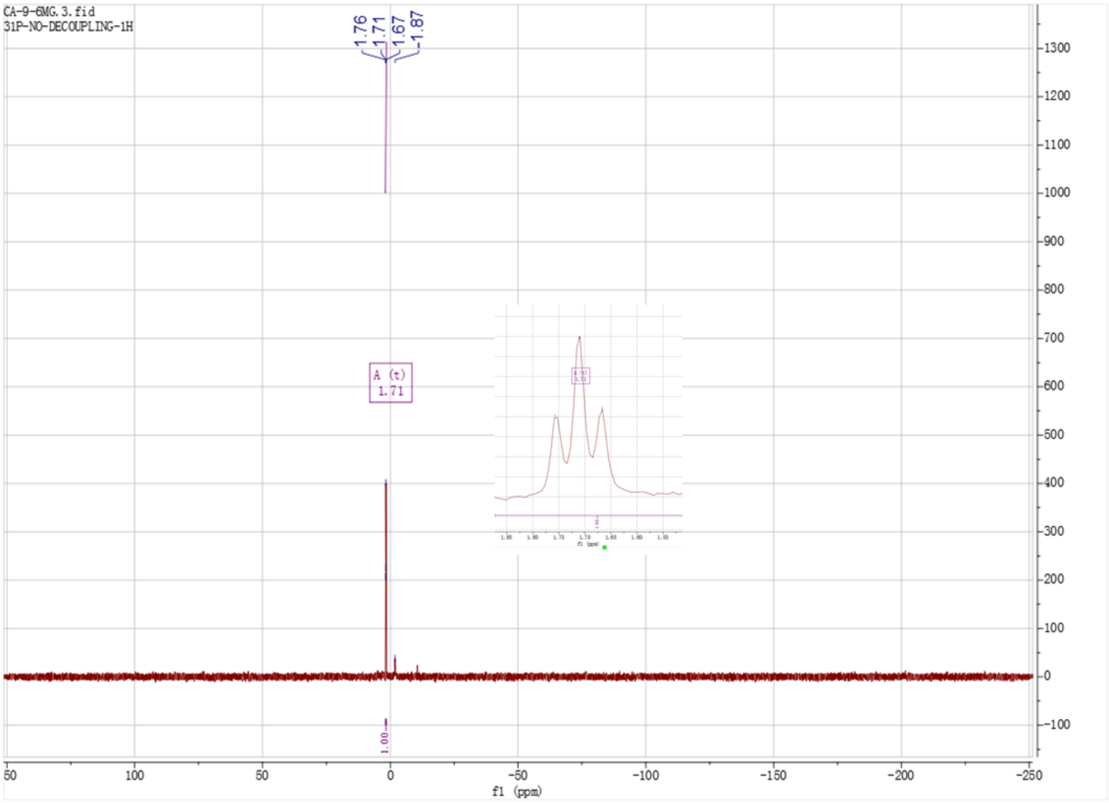

Supplementary Fig. 7 ^{31}P NMR of the mixture of INT A.

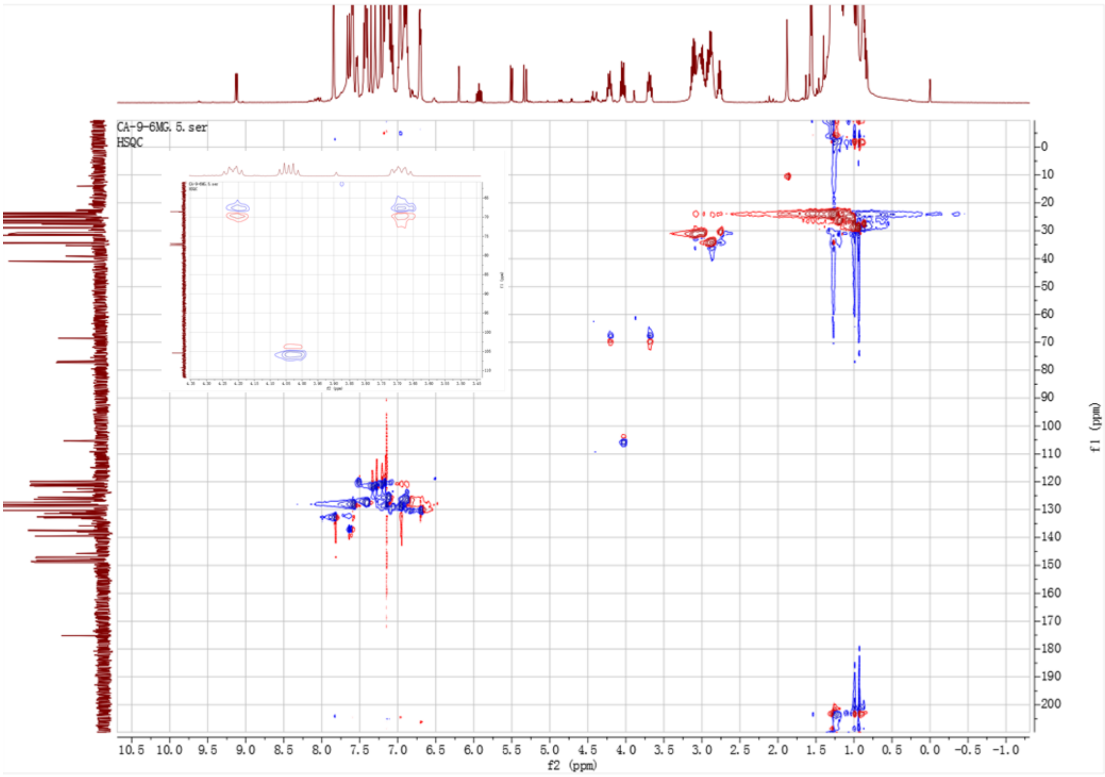

Supplementary Fig. 8 HSQC spectrum of the mixture of INT A.

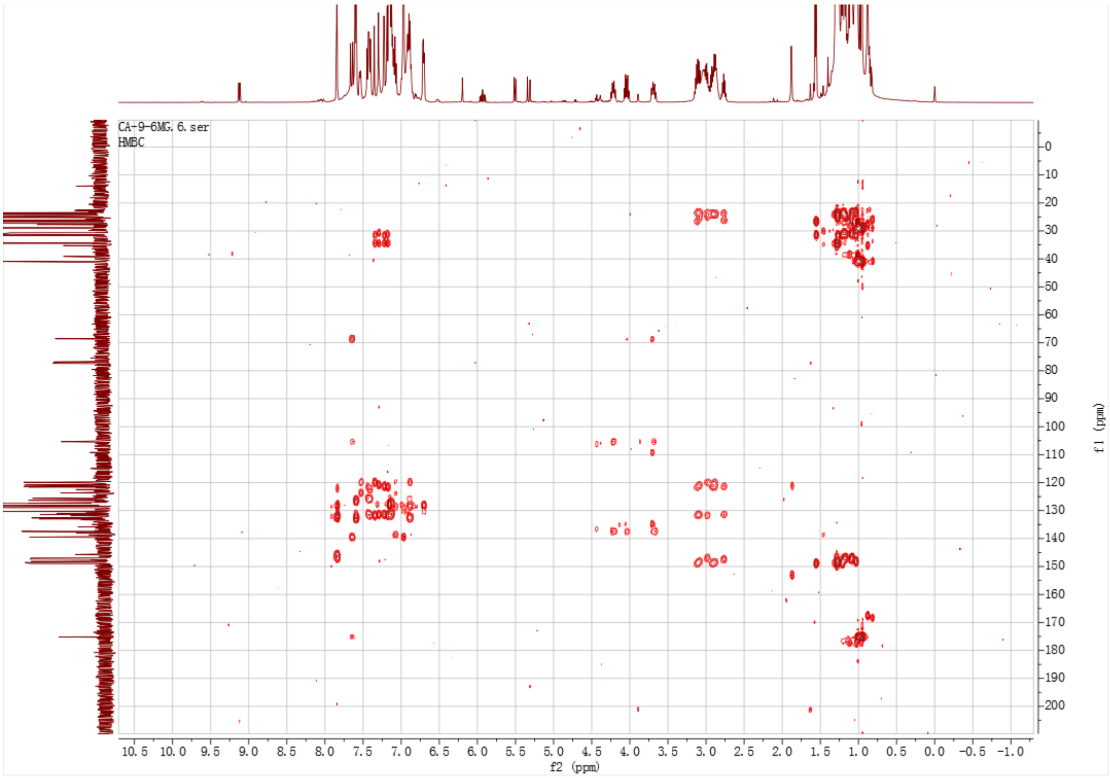

Supplementary Fig. 9 HMBC spectrum of the mixture of INT A.

HRMS (ESI) experiment

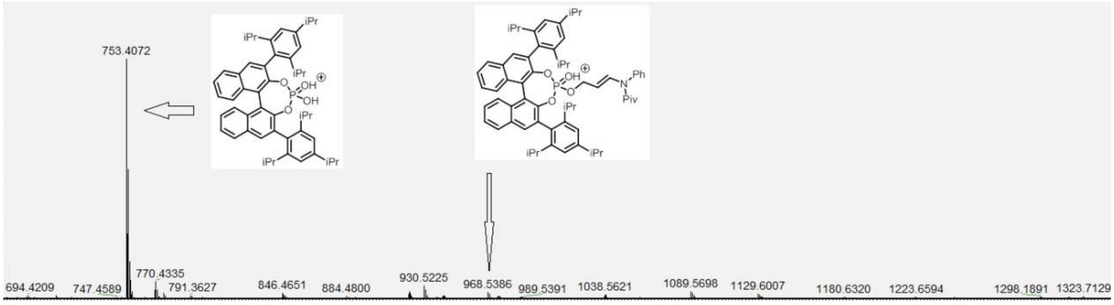

Supplementary Fig. 10 HRMS (ESI) spectrum of the mixture of INT A.

Characterization of INT-A$_{2w}$

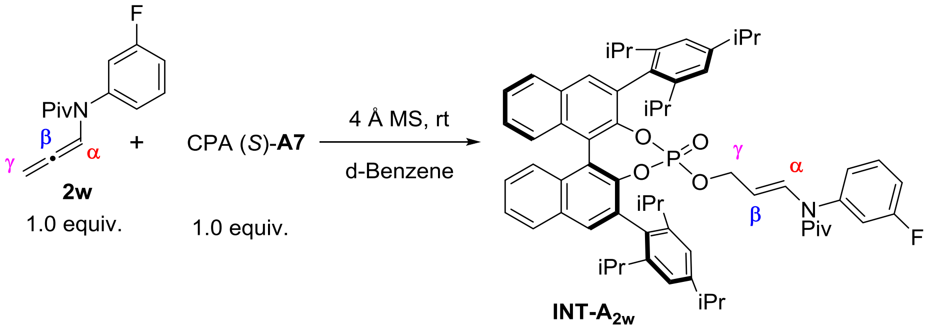

Characteristic NMR signals of **INT-A$_{2w}$**: ^{1}H NMR (500 MHz, Benzene-d_6) δ 4.30 – 4.19 (m, 1H), 4.00 (dt, J = 14.5, 7.4 Hz, 1H), 3.72 – 3.61 (m, 1H). ^{31}P NMR (202 MHz, Benzene-d_6) δ 1.83 (t, J = 8.7 Hz, with P-H coupling). ^{19}F NMR (471 MHz, Benzene-d_6) δ -110.90 (q, J = 8.5 Hz, with F-H coupling).

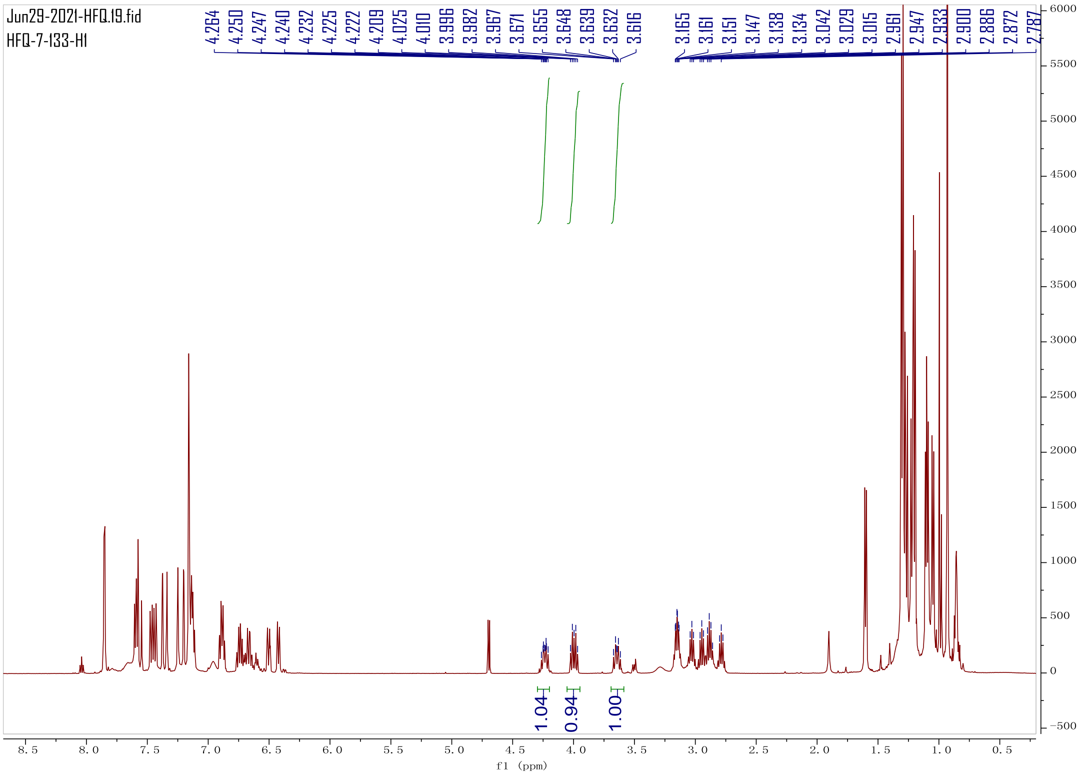

Supplementary Fig. 11 ^{1}H NMR of the mixture of **INT-A$_{2w}$**.

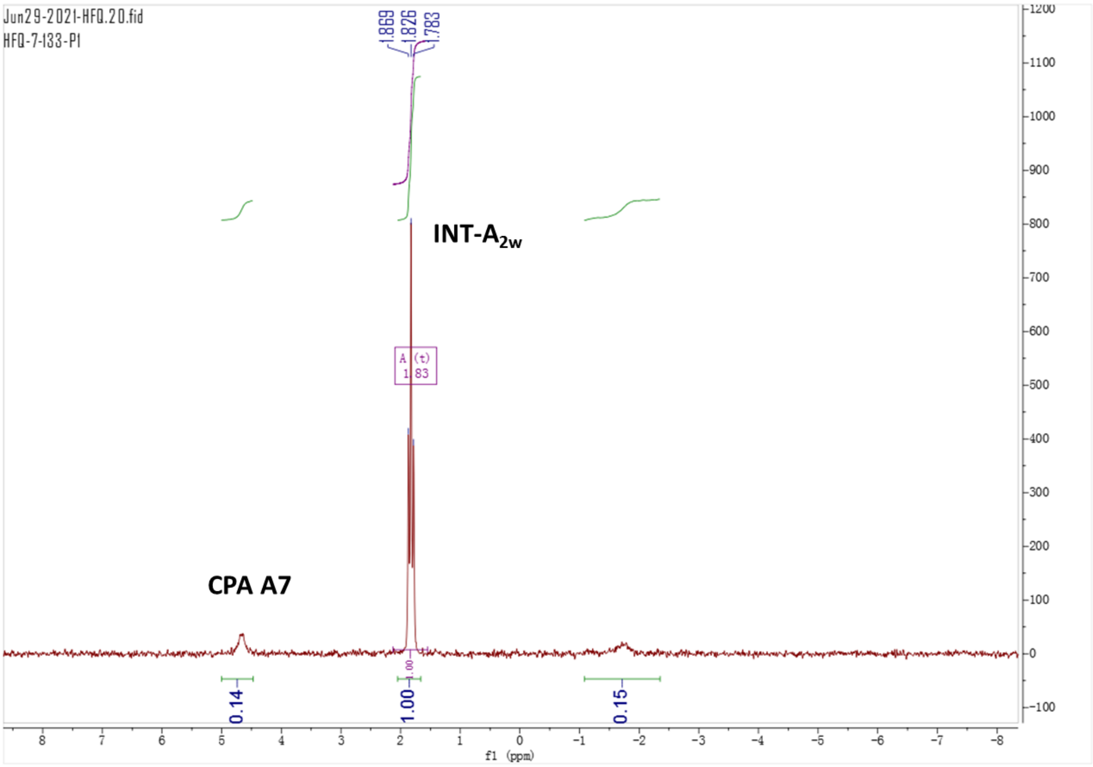

Supplementary Fig. 12 ^{31}P NMR of the mixture of **INT-A$_{2w}$**.

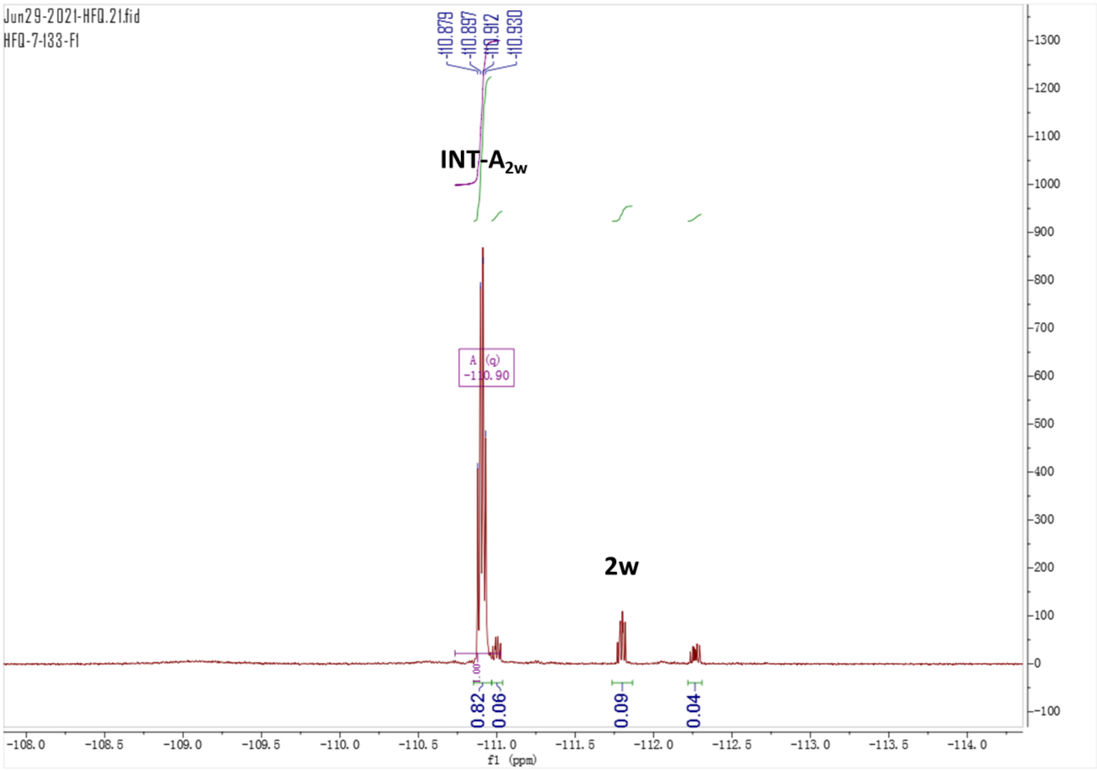

Supplementary Fig. 13 ^{19}F NMR of the mixture of **INT-A$_{2w}$**.

Monitoring the asymmetric reaction between ketone 1a and allenamide 2w by NMR:

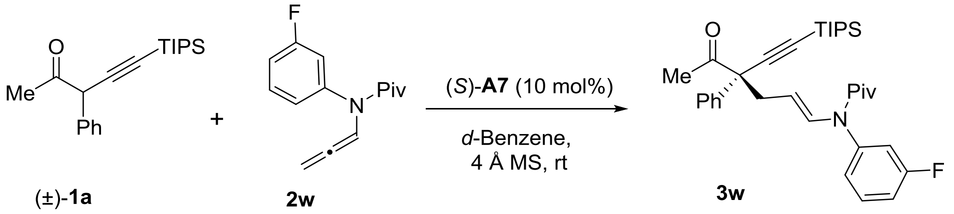

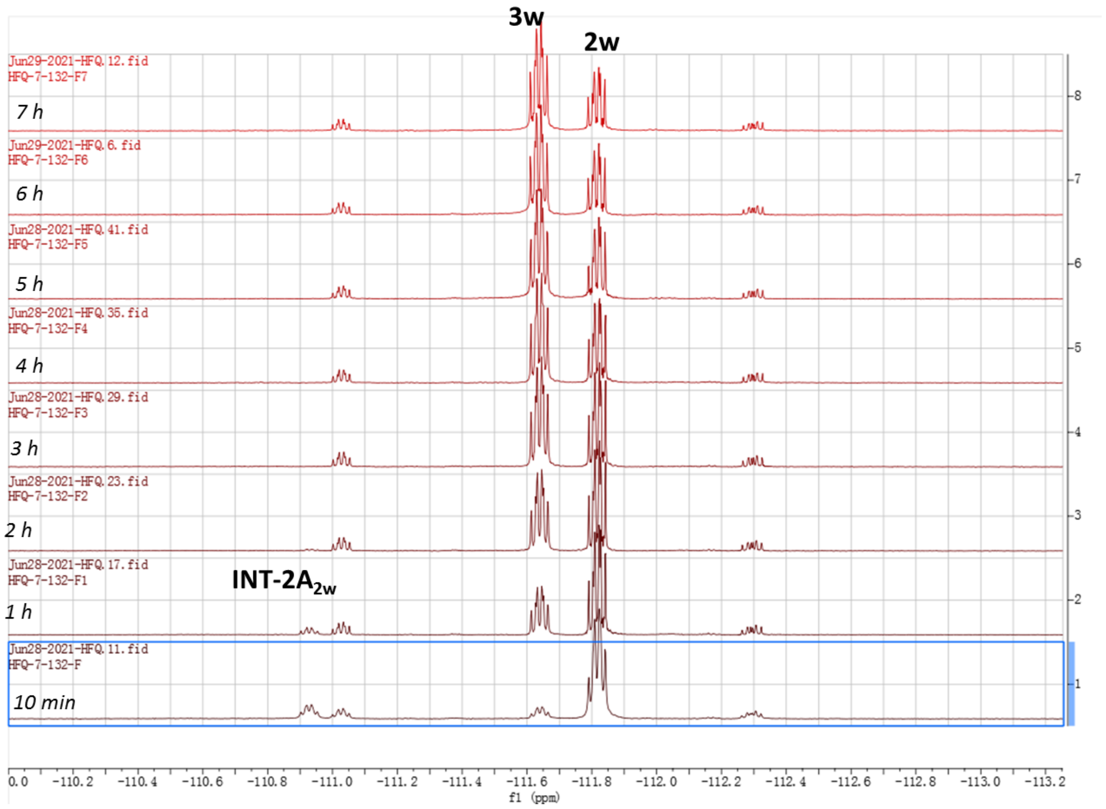

Supplementary Fig. 14 Monitoring the reaction by ^{19}F NMR

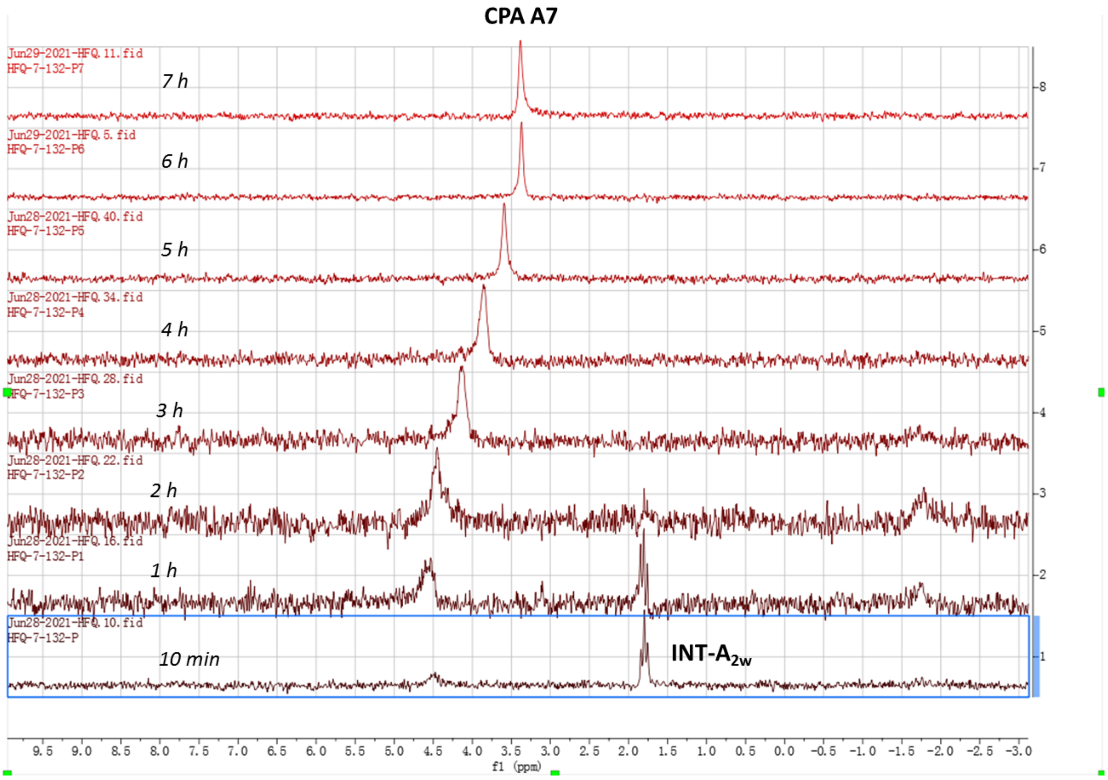

Supplementary Fig. 15 Monitoring the reaction by ^{31}P NMR

Reaction of allyl phosphate A7-allyl under the standard conditions

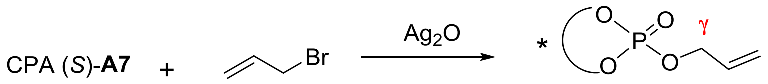

A7-allyl

^{13}C NMR, Cγ = 70.1 (d, J = 4.8 Hz, Cγ-P coupling)

^{31}P NMR (with ^{31}P-H coupling), 2.41 (t, $^{2}J(^{31}$P-H) = 13.7 Hz, P-Hγ coupling)

The allyl phosphate **A7-allyl** was prepared from CPA (*S*)-**A7** and allyl bromide according to a reported method[7].

^{1}H NMR (500 MHz, Benzene-d_6) δ 7.88 (s, 1H), 7.82 (s, 1H), 7.67 – 7.56 (m, 2H), 7.54 – 7.47 (m, 2H), 7.40 (s, 1H), 7.25 (s, 1H), 7.21 (s, 1H), 7.19 (s, 1H), 7.15 – 7.09 (m, 3H), 6.90 (q, J = 7.3 Hz, 2H), 5.16 (ddt, J = 16.7, 11.5, 6.0 Hz, 1H), 4.82 – 4.63 (m, 2H), 4.16 (td, J = 12.5, 5.2 Hz, 1H), 3.80 (td, J = 14.0, 6.7 Hz, 1H), 3.15 (mz, 2H), 3.00 (p, J = 6.8 Hz, 1H), 2.86 (h, J = 7.1 Hz, 2H), 2.73 (p, J = 6.8 Hz, 1H), 1.35 – 1.02 (m, 36H). ^{13}C NMR (126 MHz, Benzene-d_6) δ 149.4, 149.2, 149.0, 148.5, 147.9, 147.4, 146.2, 133.5, 133.3, 133.1, 132.9, 132.2, 132.1, 132.0, 131.9, 131.5,

128.8, 128.6, 126.8, 126.2, 126.1, 122.9, 122.0, 121.5, 121.1, 120.2, 119.2, **70.1 (d, *J* = 4.8 Hz)**, 34.9, 31.9, 31.8, 31.5, 31.2, 30.5, 27.0, 26.6, 25.4, 25.3, 24.5, 24.4, 24.3, 24.3, 23.8, 23.8, 23.7. ^{31}P NMR (202 MHz, Benzene-d_6) δ **2.41 (t, *J* = 13.7 Hz)**. HRMS-ESI (m/z) calculated for $C_{53}H_{62}O_4P^+$ [M+H]$^+$: 793.4380; found: 793.4341.

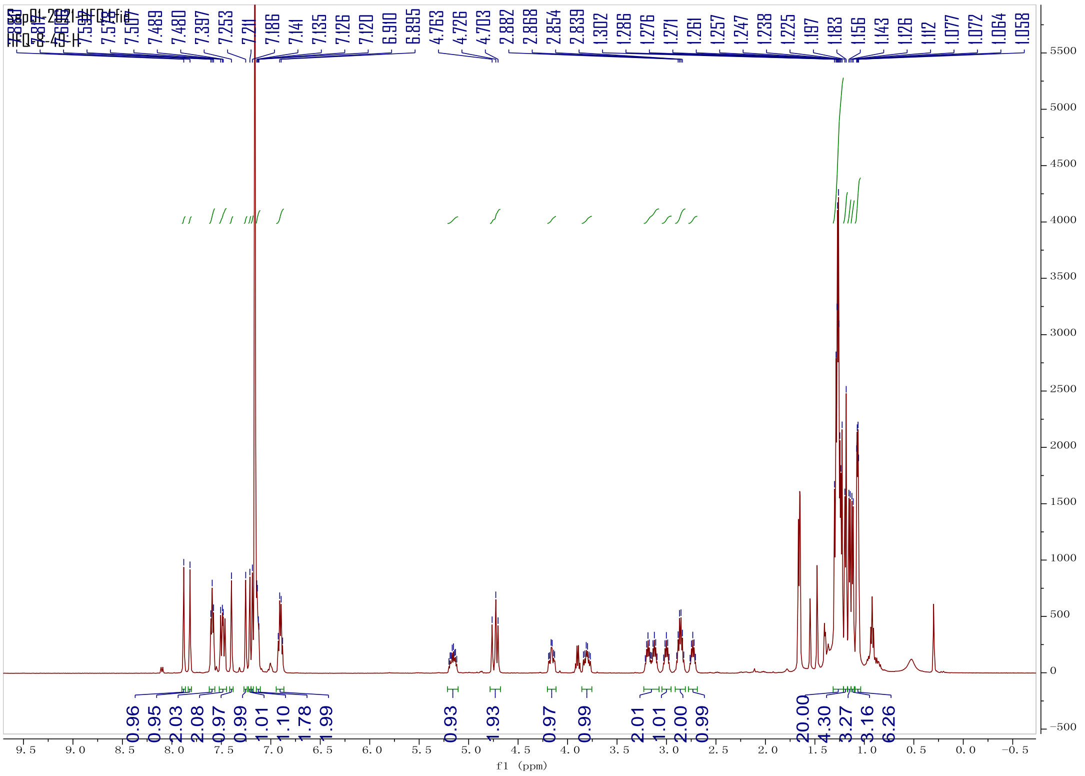

Supplementary Fig. 16 ^{1}H NMR of **A7-allyl**

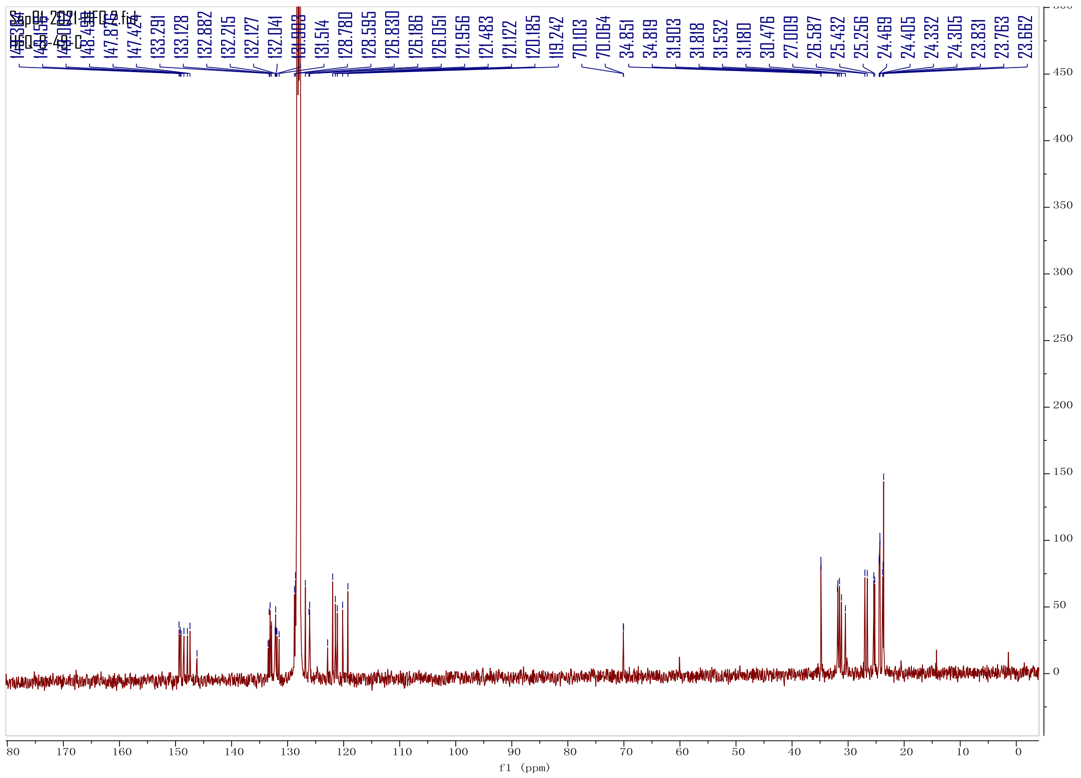

Supplementary Fig. 17 ^{13}C NMR of **A7-allyl**

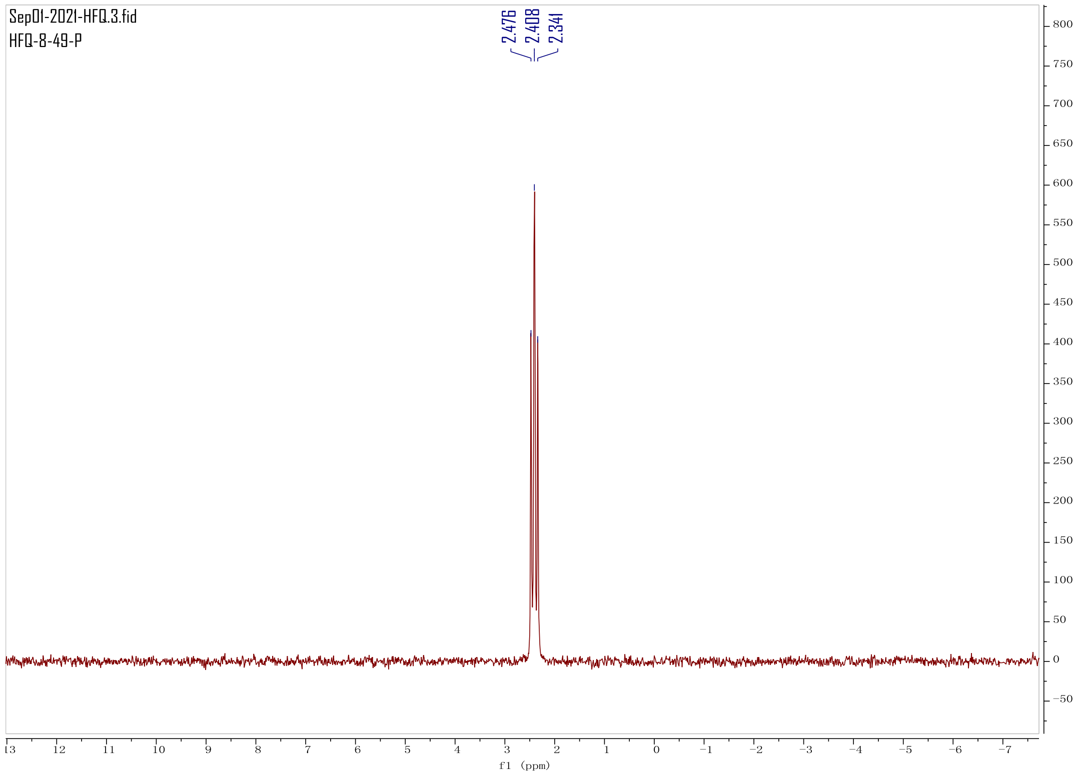

Supplementary Fig. 18 ^{31}P NMR spectrum of **A7-allyl**

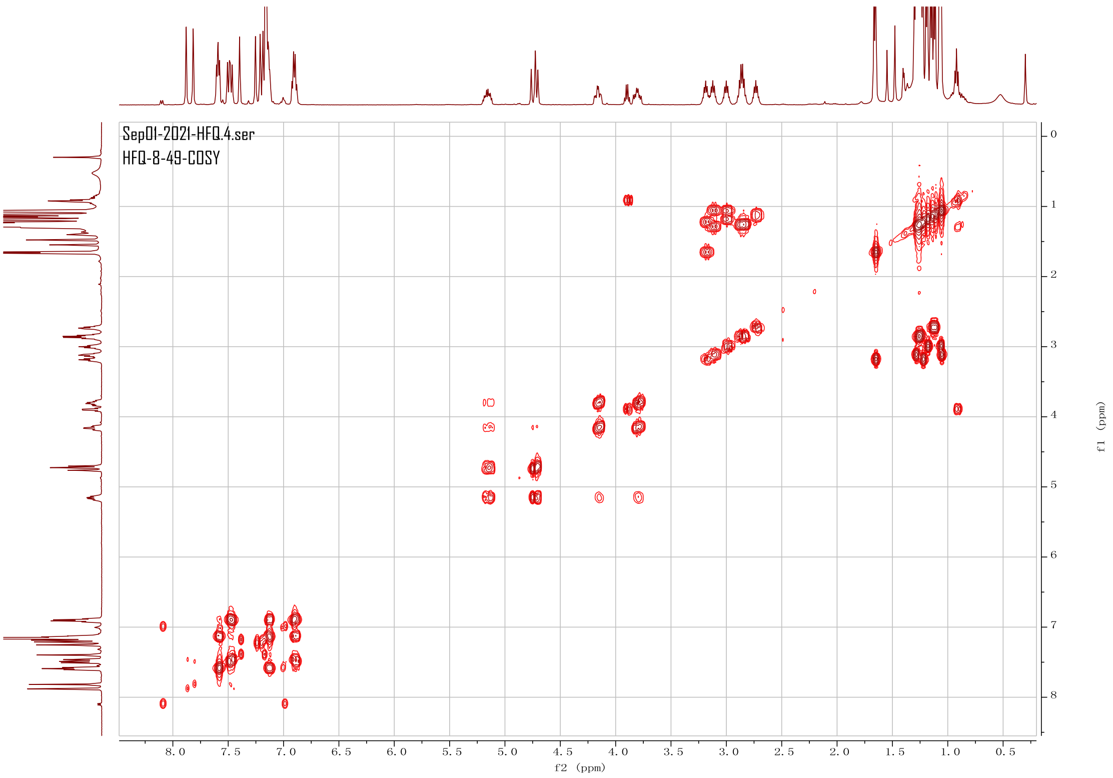

Supplementary Fig. 19 COSEY spectrum of **A7-allyl**

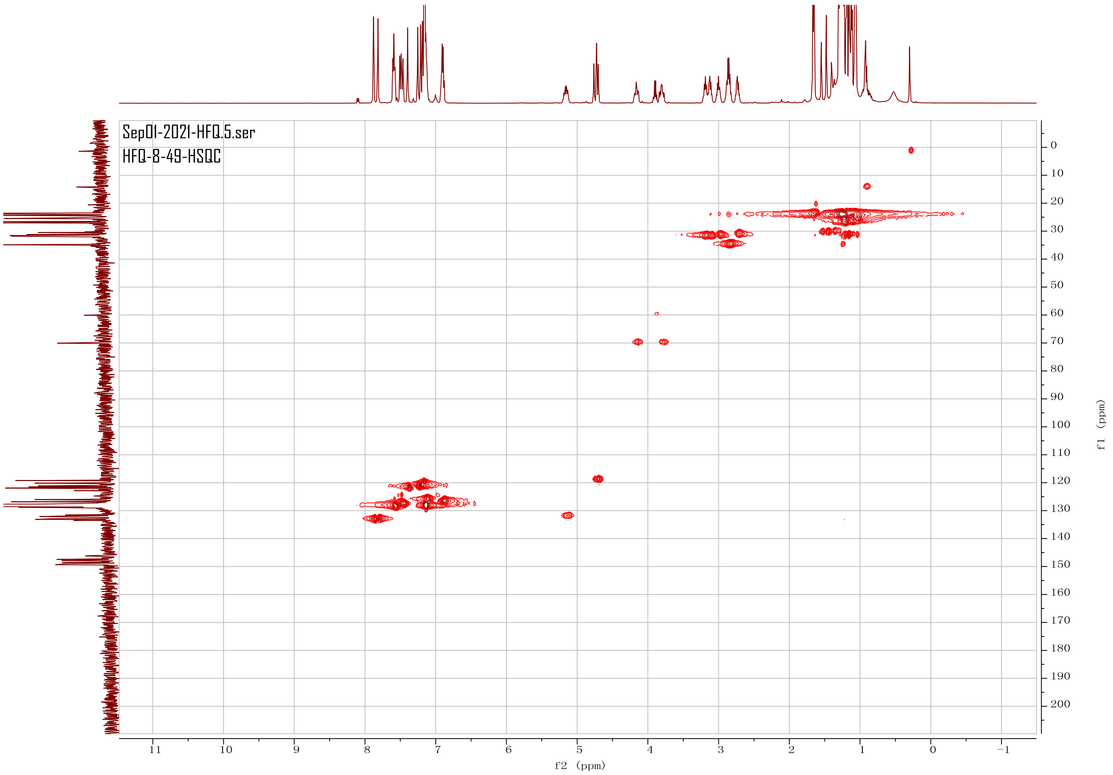

Supplementary Fig. 20 HSQC spectrum of **A7-allyl**

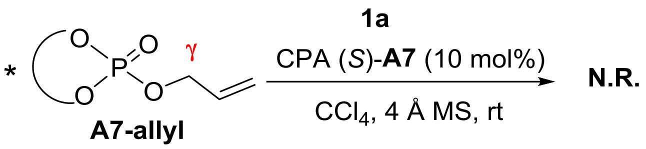

To a solution of **A7-allyl** (0.11 mmol) and **1a** (0.1 mmol) in CCl_4 (1 mL) was added 4 Å MS (30 mg) and CPA (*S*)-**A7** (0.01 mmol) at rt. After stirring overnight, the mixture was concentrated and analyzed by ^{1}H NMR, which suggested no reaction occurred.

Cross experiment

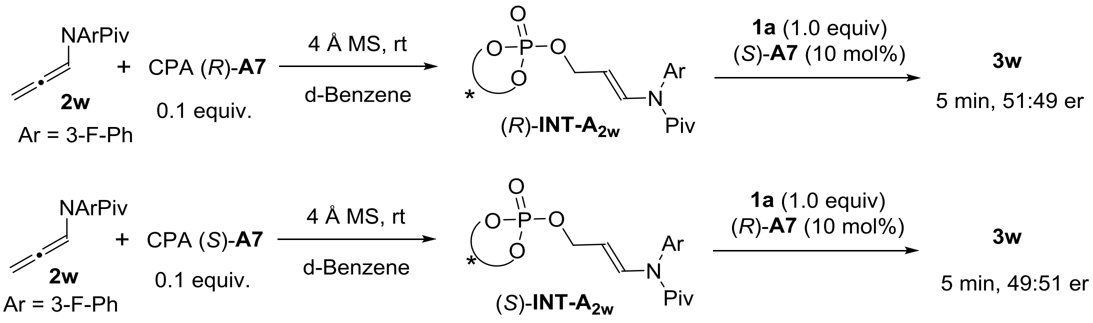

To a solution of **2w** (0.1 mmol) and 4 Å MS (20 mg) in D6-benzene (1 mL) was added CPA (*R*)-**A7** (0.01 mol) at rt. After stirring for 10 min, a solution of ketone **1a** (0.1 mmol) and (*S*)-**A7** (0.1 mmol) was added. The er value of the product **3w** was measured after 5 min (51:49 er).

On the other hand, to a solution of **2w** (0.1 mmol) and 4 Å MS (20 mg) in D6-benzene (1 mL) was added CPA (*S*)-**A7** (0.01 mol) at rt. After stirring for 10 min, a solution of ketone **1a** (0.1 mmol) and (*R*)-**A7** (0.1 mmol) was added. The er value of the product **3w** was measured after 5 min (49:51 er).

Reaction of allyl benzyl ether 6a and ketone 1a under the standard conditions

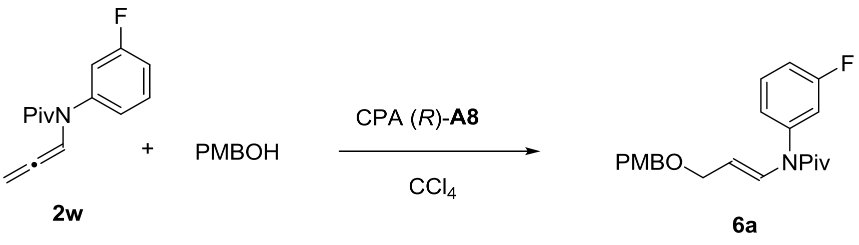

Allyl benzyl ether **6a** was prepared from the addition of allenamide **2w** and PMBOH under the catalysis of phosphoric acid (*R*)-**A8** (10 mol%). **6a**: ^{1}H NMR (500 MHz, Benzene-d_6) δ 7.99 (d, *J*

= 14.3 Hz, 1H), 7.20 (d, J = 8.2 Hz, 2H), 6.77 (d, J = 8.2 Hz, 2H), 6.74 – 6.68 (m, 1H), 6.67 – 6.61 (m, 2H), 6.56 (dd, J = 7.9, 1.8 Hz, 1H), 4.54 – 4.39 (m, 1H), 4.29 (s, 2H), 3.82 (d, J = 6.7 Hz, 2H), 3.30 (s, 3H), 0.98 (s, 9H). ^{13}C NMR (126 MHz, Benzene-d_6) δ 175.2, 164.1, 162.1, 159.6, 142.0 (d, J = 9.4 Hz), 134.9, 131.2, 130.4 (d, J = 8.9 Hz), 129.5, 126.7 (d, J = 3.1 Hz), 118.2 (d, J = 21.8 Hz), 115.6 (d, J = 20.8 Hz), 114.1, 109.6, 71.7, 69.0, 54.8, 41.3, 29.3. ^{19}F NMR (471 MHz, Benzene-d_6) δ -111.0 (q, J = 8.4 Hz).

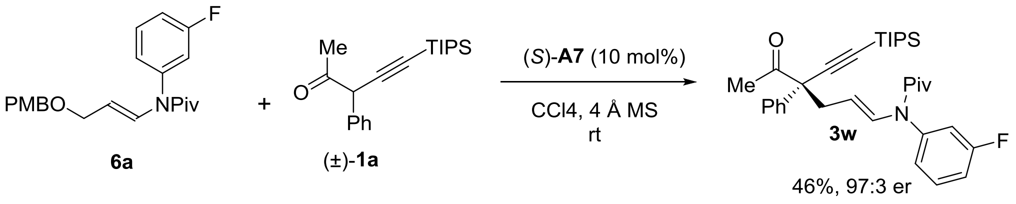

No **INT-A$_{2w}$** intermediate was observed by mixing **6a** with CPA **A7**

To a solution of **6a** (0.1 mmol), **1a** (0.1 mmol) and 4 Å MS (30 mg) was added CPA (S)-**A7** (0.01 mmol) at rt. After stirring overnight, the reaction mixture was directly subjected for column chromatography to give the product **3w** in 46% yield with 97:3 er.

Mixing **6a** and CPA **A7** in D6-benzene did not afforded the covalent adduct **INT-A$_{2w}$** as monitored by ^{31}P NMR.

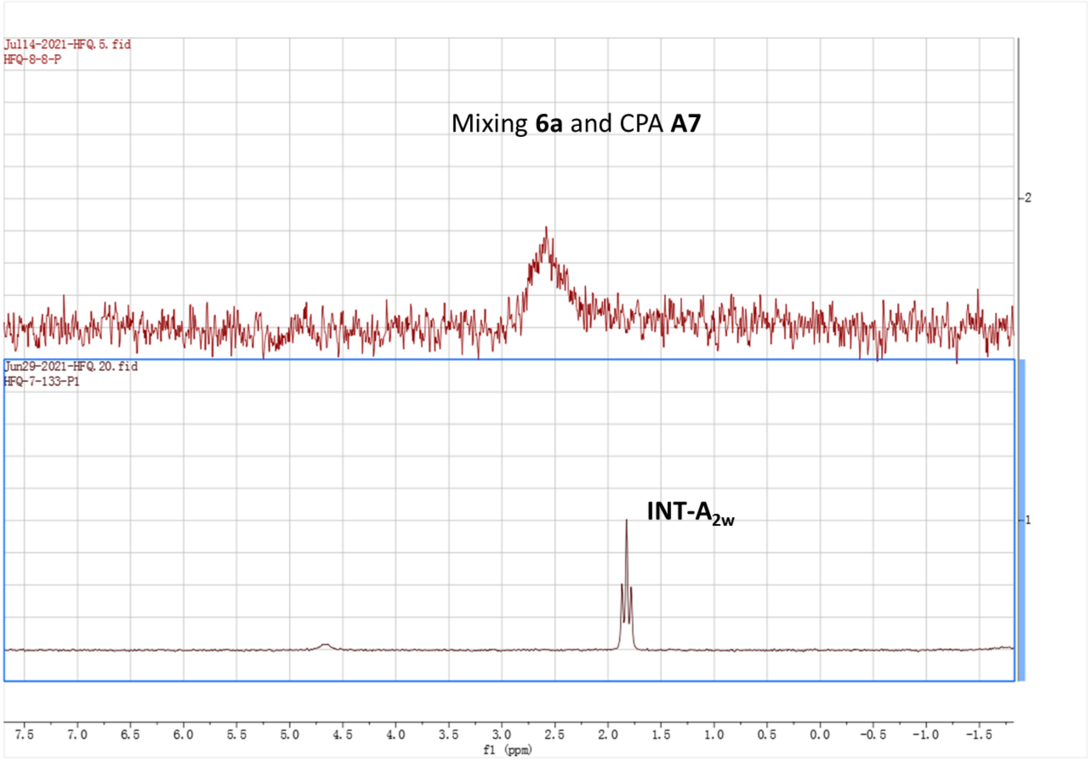

Supplementary Fig. 21 ^{31}P NMR of mixing **6a** and CPA **A7**.

6. Large scale asymmetric synthesis of 3a

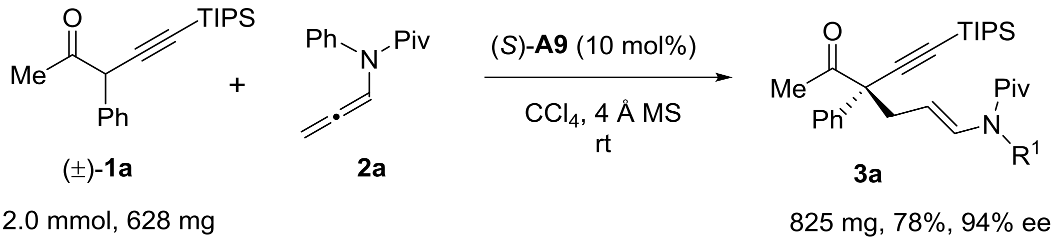

To a dried flask charged was added 4Å molecular sieves (2.0 g), allenamide **2a** (473 mg, 2.2 mmol) and (*S*)-**A9** (152 mg 0.2 mmol) sequentially, which was followed by adding a solution of ketone **1a** (628 mg, 2 mmol) in CCl_4 (20 mL). After stirring at the room temperature for 20 h, the mixture was filtered through Celite and the filtrate was concentrated under vacuum to give a residue, which was purified by column chromatography on silica gel (petroleum ether:EtOAc, 30:1 ~10:1) to give the products **3a** (825 mg, 78% yield, 94% ee).

7. Transformations of chiral products

(*S*)-N-(4-acetyl-4-phenyl-6-(triisopropylsilyl)hex-5-yn-1-yl)-N-phenylpivalamide (**7a**)

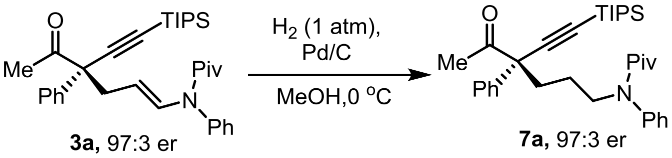

To a solution of **3a** (14 mg) in MeOH (1 mL) was added Pd/C (10% wt, 50% water, 6mg). The reaction mixture was then stirred under H_2 atmosphere (1 bar) for 3 h at 0 ^{o}C. The reaction mixture was then filtered through Celite and concentrated under vacuum to give a residue, which was purified by column chromatography on silica gel (petroleum ether:EtOAc = 16:1) to give **7a** (11 mg, 78% yield) as colorless oil. ^{1}H NMR (500 MHz, Chloroform-*d*) δ 7.5 (d, *J* = 7.4 Hz, 2H), 7.3 (m, 6H), 7.1 (d, *J* = 7.0 Hz, 2H), 3.7 (m, 1H), 3.5 (m, 1H), 2.1 (m, 4H), 1.9 (m, 1H), 1.1 (s, 21H), 1.0 (s, 9H). ^{13}C NMR (126 MHz, $CDCl_3$) δ 203.9, 177.3, 143.6, 138.3, 129.8, 128.9, 128.8, 127.6, 127.6, 126.8, 106.3, 90.2, 59.4, 52.4, 40.9, 35.5, 29.5, 26.5, 23.6, 18.7, 11.3. $[\alpha]_D^{23}$ = -8.0 (c 1.0, $CHCl_3$). IR: ν = 3022, 2960, 2927, 2895, 2839, 2162, 1719, 1628, 1593 cm^{-1}. HRMS-APCI (m/z)

calculated for $C_{34}H_{50}NO_2Si^+$ $[M+H]^+$: 532.3605; found: 532.3612. HPLC: Chiralpak ID column, 90:10 hexanes/isopropanol, 1 mL/min; t_R = 5.6 min (minor), 6.8 min (major); 97:3 er.

(*R*)-N-(4-acetyl-4-phenyl-6-(triisopropylsilyl)hexyl)-N-phenylpivalamide (**8a**)

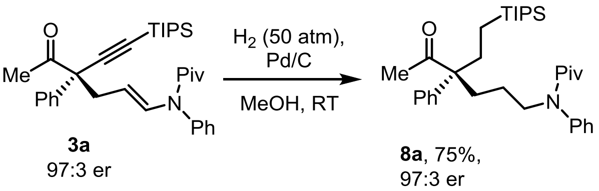

To a solution of **3a** (24 mg) in MeOH (1 mL) was added Pd/C (10% wt, 50% water, 10mg). After stirring under H_2 atmosphere (45 Bar) at rt for 12 h, the mixture was filtered through Celite and concentrated under vacuum to give a residue, which was purified by column chromatography on silica gel (petroleum ether:EtOAc = 15:1) to give **8a** (18 mg 75% yield) as colorless oil. ^{1}H NMR (500 MHz, Chloroform-*d*) δ 7.3 (m, 5H), 7.2 (t, *J* = 7.3 Hz, 1H), 7.1 (d, *J* = 7.2 Hz, 2H), 7.1 (d, *J* = 6.2 Hz, 2H), 3.6 (m, 2H), 2.0 (m, 4H), 1.9 (s, 3H), 1.2 (m, 3H), 0.9 (m, 31H). ^{13}C NMR (126 MHz, $CDCl_3$) δ 208.7, 175.2, 141.4, 140.0, 127.5, 126.9, 126.5, 125.6, 124.7, 124.7, 58.4, 51.0, 38.8, 27.4, 26.7, 25.0, 24.1, 20.0, 16.7, 16.6, 8.7. $[\alpha]_D^{23}$ = -5.8 (c 1.0, $CHCl_3$). IR: ν = 3018, 2960, 2943, 2893, 2866, 1705, 1628, 1595, 1534 cm^{-1}. HRMS-APCI (m/z) calculated for $C_{34}H_{54}NO_2Si^+$ $[M+H]^+$: 536.3918; found: 536.3926. HPLC: Chiralpak IC column, 90:10 hexanes/isopropanol, 1 mL/min; t_R =7.9min (minor), 8.3 min (major); 97:3 er.

(*S*,*E*)-N-(4-(1-benzyl-1H-1,2,3-triazol-4-yl)-5-oxo-4-phenylhex-1-en-1-yl)-N-phenylpivalamide (**9m**)

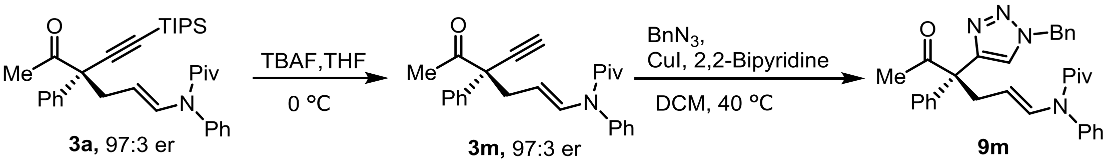

To a solution of **3a** (38 mg 0.07 mmol) in THF (1 mL) was added TBAF (1 M in THF, 88 uL) at 0 °C. After stirring at this temperature for 4 h, the mixture was quenched by NH_4Cl (aq) and extracted with EtOAc. The combined organic layer was dried over Na_2SO4, filtered, and

concentrated to give a residue, which was purified by column chromatography on silica gel (petroleum ether:EtOAc = 15:1) to give **3m** (25 mg, 96% yield).

To a 8 mL flask charged with **3m** (25 mg), BnN_3(11 mg, 1.2 eq), 2,2'-Bipyridine (1 mg, 0.1 eq) and CuI (1.3 mg, 0.1 eq) was added DCM (1 mL) at rt. After stirring at 40 ℃ for 16 h, the mixture was cooled to rt and concentrated to give a residue, which was purified by column chromatography on silica gel (petroleum ether:EtOAc = 10:1) to give **9m** as sticky solid (23 mg, 68% yield). ^{1}H NMR (500 MHz, Chloroform-*d*) δ 7.5 (s, 1H), 7.4 (m, 3H), 7.2 (m, 9H), 7.0 (d, *J* = 7.3 Hz, 2H), 6.9 (m, 2H), 5.5 (m, 2H), 3.8 (dt, *J* = 14.5, 7.5 Hz, 1H), 3.1 (dd, *J* = 14.1, 6.5 Hz, 1H), 3.0 (dd, *J* = 14.2, 8.4 Hz, 1H), 2.0 (s, 3H), 1.0 (s, 9H). ^{13}C NMR (126 MHz, $CDCl_3$) δ 206.4, 175.9, 148.1, 140.3, 140.0, 134.8, 134.0, 130.2, 129.1, 128.9, 128.7, 128.6, 128.2, 127.9, 127.5, 127.2, 123.3, 109.1, 61.3, 54.0, 40.9, 37.6, 29.1, 27.0. $[\alpha]_D^{23}$ = 6.6 (c 1.0, $CHCl_3$). IR: ν = 3068, 3020, 2968, 2918, 2871, 1711, 1658, 1637, 1597, 1522 cm^{-1}. HRMS-APCI (m/z) calculated for $C_{32}H_{35}N_4O_2^+$ $[M+H]^+$: 507.2755; found: 507.2762. HPLC: Chiralpak IA column, 60:40 hexanes/isopropanol, 1 mL/min; t_R =7.1min (minor), 10.7 min (major); 96% ee.

(*S*)-4-acetyl-4-phenyl-6-(triisopropylsilyl)hex-5-ynal (**10a**)

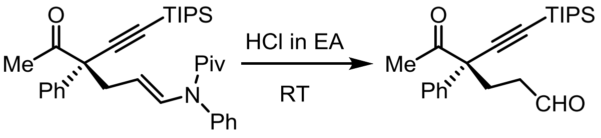

3a, 97:3 er **10a,** 97:3 er

To a solution of **3a** (160 mg) in EtOAc (2 mL) was added (2 M in EtOAc, 2 mL HCl), the reaction mixture was stirred at room temperature for 5 min then quenched by adding $NaHCO_3$ (aq) and extracted with EtOAc for 3 times. The combined organic layer was dried over Na_2SO4, filtered, and concentrated to give a residue, which was purified by column chromatography on silica gel (petroleum ether:EtOAc = 20:1) to give **10a** (99 mg, 88% yield) as colorless oil. ^{1}H NMR (500 MHz, Chloroform-*d*) δ 9.7 (s, 1H), 7.5 (d, *J* = 7.6 Hz, 2H), 7.4 (t, *J* = 7.5 Hz, 2H), 7.3 (t, *J* = 7.3 Hz, 1H), 2.6 (m, 1H), 2.5 (m, 1H), 2.3 (m, 2H), 2.2 (s, 3H), 1.1 (s, *J* = 4.3 Hz, 21H). ^{13}C NMR (126 MHz, $CDCl_3$) δ 203.4, 201.5, 137.6, 129.1, 128.0, 126.7, 105.4, 91.3, 58.8, 40.4, 30.7, 26.4, 18.7, 11.3. $[\alpha]_D^{23}$ = -10.6 (c 1.0, $CHCl_3$). IR: ν = 3053, 2960, 2945, 2891, 2866, 2725, 2168, 1721, 1595 cm^{-1}. HRMS-ESI (m/z) calculated for $C_{23}H_{35}O_2Si^+$ $[M+H]^+$: 371.2401; found: 371.2405.

HPLC: Chiralpak IC column, 97:03 hexanes / isopropanol, 1 mL/min; t_R =7.9min (major), 9.1 min (minor); 97:3 er.

(*S*)-1-((triisopropylsilyl)ethynyl)-5,6-dihydro-[1,1'-biphenyl]-2(1H)-one (**11a**)

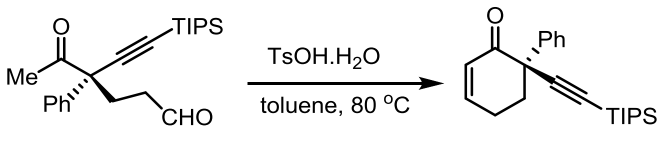

10a, 97:3 er **11a,** 97:3 er

To a solution of **10a** (25 mg) in toluene (1 mL) was added TsOH H_2O (6 mg, 0.5 eq) at rt. After stirring at 80 ℃ for 20 h, the mixture was cooled to rt and concentrated to give a residue, which was purified by column chromatography on silica gel to give **11a** (22 mg, 88% yield) as clear oil. ^{1}H NMR (500 MHz, Chloroform-*d*) δ 7.5 (d, *J* = 7.7 Hz, 2H), 7.4 (t, *J* = 7.6 Hz, 2H), 7.3 (s, 1H), 7.0 (m, 1H), 6.2 (d, *J* = 9.8 Hz, 1H), 2.9 (m, 1H), 2.5 (m, 2H), 2.2 (m, 1H), 1.6 (s, 3H), 1.1 (s, 21H). ^{13}C NMR (126 MHz, $CDCl_3$) δ 194.6, 149.9, 140.2, 128.6, 128.2, 127.6, 127.3, 104.4, 88.3, 53.5, 37.9, 24.8, 18.7, 11.3. $[\alpha]_D^{23}$ = 53.8(c 1.0, $CHCl_3$). IR: ν = 3057, 2939, 2927, 2891, 2864, 2160, 1688, 1636, 1620 cm^{-1}. HRMS-ESI (m/z) calculated for $C_{23}H_{33}OSi^+$ $[M+H]^+$: 353.2295; found: 353.2296. HPLC: Chiralpak IC column, 90:10 hexanes/isopropanol, 1 mL/min; t_R =7.9min (minor), 8.3 min (major); 97:3 er.

N-((S,E)-4-((R)-1-hydroxyethyl)-4-phenyl-6-(triisopropylsilyl)hex-1-en-5-yn-1-yl)-N-phenylpivalamide (**12a**)

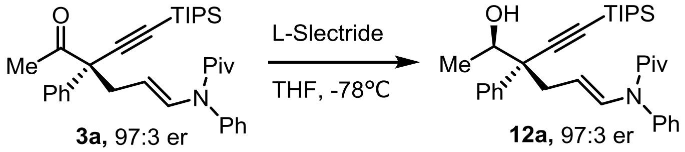

3a, 97:3 er **12a,** 97:3 er

To a solution of **3a** (39 mg) in THF (1 mL) was added L-Selectride (100 ul, 1 M in THF, 1.5 eq) at -78 ^{o}C. The reaction mixture was slowly warmed to 0 ^{o}C and stirred for another 3 h. The mixture was quenched by adding NH_4Cl (aq) and extracted with EtOAc for 3 times. The combined organic layer was dried over Na_2SO4, filtered, and concentrated to give a residue, which was purified by column chromatography on silica gel (petroleum ether:EtOAc = 4:1) to give **12a** (38

mg, 97% yield) as sticky solid. ^{1}H NMR (500 MHz, Chloroform-*d*) δ 7.5 (d, *J* = 7.7 Hz, 2H), 7.3 (d, *J* = 14.2 Hz, 1H), 7.3 (m, *J* = 13.6, 6.6 Hz, 5H), 7.2 (t, *J* = 7.3 Hz, 1H), 6.9 (m, 2H), 4.2 (dt, *J* = 14.5, 7.4 Hz, 1H), 4.0 (t, *J* = 6.1 Hz, 1H), 2.6 (dd, *J* = 13.7, 7.0 Hz, 1H), 2.5 (dd, *J* = 13.7, 7.8 Hz, 1H), 1.5 (d, *J* = 6.8 Hz, 1H), 1.2 (d, *J* = 6.2 Hz, 3H), 1.0 (m, 30H). ^{13}C NMR (126 MHz, $CDCl_3$) δ 175.9, 140.1, 139.6, 133.8, 130.1, 128.9, 128.1, 128.0, 127.9, 126.8, 110.1, 109.0, 87.3, 73.1, 53.3, 40.9, 39.3, 29.2, 18.9, 18.7, 11.2. $[\alpha]_D^{23}$ = 17.4(c 1.0, $CHCl_3$). IR: ν = 3063,2960, 2941, 2891, 2864, 2247, 2166, 1655, 1636, 1593 cm^{-1}. HRMS-ESI (m/z) calculated for $C_{34}H_{50}NO_2Si^+$ $[M+H]^+$: 532.3605; found: 532.3613. HPLC: Chiralpak ID column, 90:10 hexanes/isopropanol, 1 mL/min; t_R =6.8min (major), 7.6 min (minor); 97:3 er.

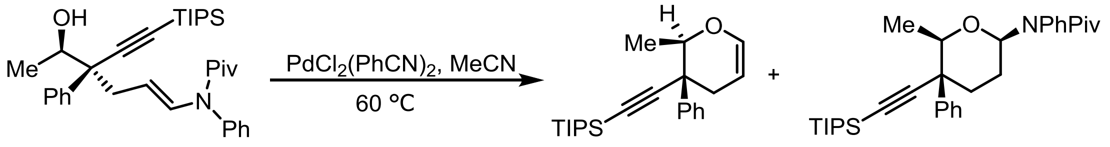

12a, 95.5:4.5 er **13a** 43%, 95:5 er **13b** 21%, 95:5 er

To a 8 mL flask charged with **12a** (57 mg) and $PdCl_2(PhCN)_2$ (4 mg, 0.1 eq) was added MeCN (1 mL) at rt. After stirring at 60 ℃ for 36 h, the mixture was cooled to rt and concentrated under vacuum to give a residue, which was purified by column chromatography on silica gel (petroleum ether:EtOAc = 30:1) to give **13a** as sticky solid (16 mg, 43% yield) and **13b** as clear oil (12 mg 21% yield).

Triisopropyl(((*2R*,*3S*)-2-methyl-3-phenyl-3,4-dihydro-2H-pyran-3-yl)ethynyl)silane (**13a**)

^{1}H NMR (500 MHz, Chloroform-*d*) δ 7.7 (d, *J* = 7.6 Hz, 2H), 7.3 (t, *J* = 7.5 Hz, 2H), 7.3 (t, 1H), 6.5 (d, *J* = 6.1 Hz, 1H), 5.0 (t, *J* = 6.6 Hz, 1H), 4.2 (q, *J* = 6.4 Hz, 1H), 2.8 (d, *J* = 18.0 Hz, 1H), 2.6 (dd, *J* = 17.9, 3.7 Hz, 1H), 1.2 (d, *J* = 6.4 Hz, 3H), 1.1 (s, 21H). ^{13}C NMR (126 MHz, $CDCl_3$) δ 144.5, 140.1, 128.1, 127.8, 127.0, 112.3, 101.2, 84.7, 77.2, 43.0, 35.6, 18.7, 16.3, 11.3. $[\alpha]_D^{23}$ = -20.0 (c 1.0, $CHCl_3$). IR: ν = 3066, 2962, 2941, 2921, 2868, 2163, 1736, 1660, 1599 cm^{-1}. HRMS-ESI (m/z) calculated for $C_{23}H_{35}OSi^+$ $[M+H]^+$: 355.2452; found: 355.2462. SFC: Chiralpak OD-H column, 99:01 CO_2 /isopropanol, 3 mL/min; t_R =5.2min (major), 5.9 min (minor); 95:5 er.

N-((2R,5S,6R)-6-methyl-5-phenyl-5-((triisopropylsilyl)ethynyl)tetrahydro-2H-pyran-2-yl)-N-phenylpivalamide **(13b)**

^{1}H NMR (500 MHz, Chloroform-*d*) δ 7.5 (d, *J* = 7.9 Hz, 2H), 7.4 (m, 3H), 7.1 (t, *J* = 7.3 Hz, 1H), 7.0 (t, *J* = 7.6 Hz, 2H), 6.0 (d, *J* = 11.2 Hz, 1H), 4.2 (q, *J* = 6.4 Hz, 1H), 2.3 (td, *J* = 13.6, 3.9 Hz, 1H), 1.9 (d, *J* = 13.7 Hz, 1H), 1.3 (d, *J* = 13.5 Hz, 1H), 1.3 (d, *J* = 6.4 Hz, 3H), 1.2 (m, 1H), 1.1 (s, 30H). ^{13}C NMR (126 MHz, $CDCl_3$) δ 178.2, 140.3, 138.4, 129.2, 128.5, 128.1, 127.3, 126.1, 112.6, 83.9, 83.8, 80.4, 77.2, 43.3, 41.5, 40.3, 29.4, 24.0, 18.7, 17.5, 11.3. $[\alpha]_D^{23}$ = -31.2 (c 1.0, $CHCl_3$). IR: ν = 3056, 2984, 2965, 2941, 2924, 2866, 2159, 1652, 1596 cm^{-1}. HRMS-ESI (m/z) calculated for $C_{34}H_{50}NO_2Si^+$ $[M+H]^+$: 532.3605; found: 532.3611. HPLC: Chiralpak IA column, 95:05 hexanes/isopropanol, 1 mL/min; t_R =3.4min (major), 3.8 min (minor); 95:5 er.

8. X-ray structures

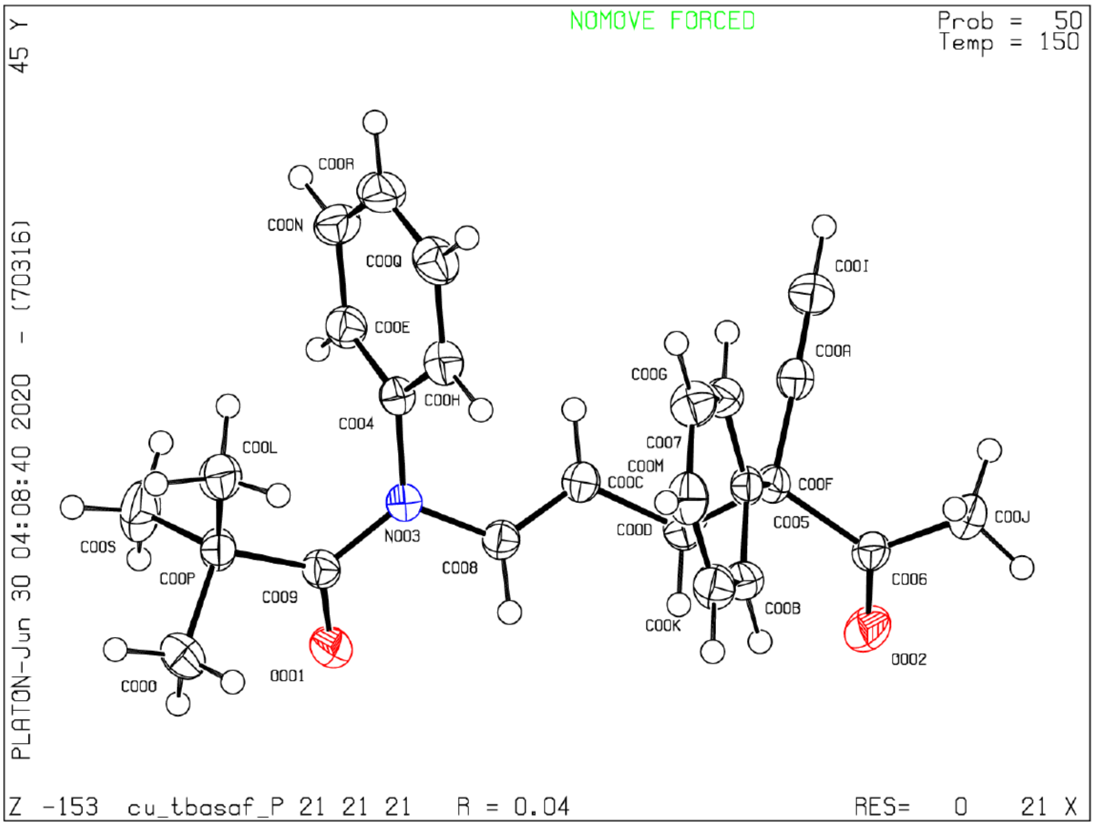

Supplementary Fig. 22 X-Ray structure of **3n** (with CCDC number 2047788)

Supplementary Table 22. Crystal data for **3n.**

Identification code	*cu_tbasaf_0m*
Empirical formula	$C_{25}H_{27}NO_2$
Formula weight	373.47
Temperature/K	150
Crystal system	orthorhombic
Space group	P212121
a/Å	8.7417(5)
b/Å	9.7806(5)
c/Å	24.5349(13)
α/°	90
β/°	90
γ/°	90
Volume/Å3	2097.7(2)
Z	4
ρcalcg/cm3	1.183
μ/mm-1	0.581
F(000)	800
Crystal size/mm^3	0.2 × 0.15 × 0.1
Radiation	CuKα (λ = 1.54178)
2Θ range for data collection/°	10.744 to 158.342
Index ranges	-11 ≤ h ≤ 10, -12 ≤ k ≤ 12, -31 ≤ l ≤ 31
Reflections collected	34092
Independent reflections	4466 [Rint = 0.0721, Rsigma = 0.0472]
Data/restraints/parameters	4466/0/257
Goodness-of-fit on F2	1.059
Final R indexes [I>=2σ (I)]	R1 = 0.0387, wR2 = 0.1021
Final R indexes [all data]	R1 = 0.0404, wR2 = 0.1039
Largest diff. peak/hole / e Å-3	0.18/-0.21
Flack parameter	0.18(11)

9. HPLC traces

(*S*,*E*)-N-(4-acetyl-4-phenyl-6-(triisopropylsilyl)hex-1-en-5-yn-1-yl)-N-phenylpivalamide (**3a**)

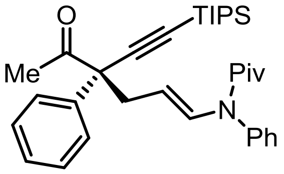

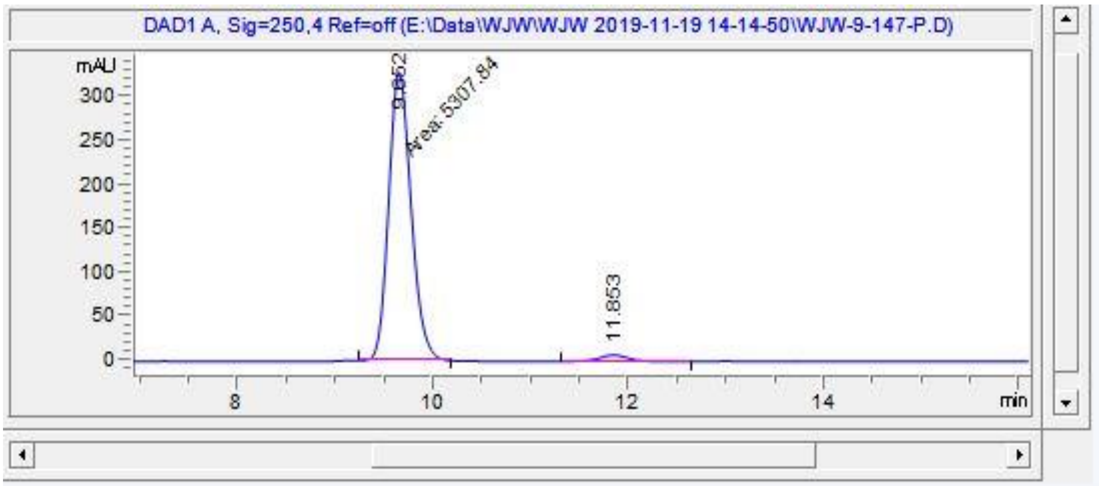

#	Time	Type	Area	Height	Width	Area%	Symmetry
1	9.652	MM	5307.8	331.6	0.2667	97.096	0.827
2	11.853	BB	158.8	7.5	0.3201	2.904	0.98

Supplementary Fig. 23 HPLC spectrum of racemic **3a.**

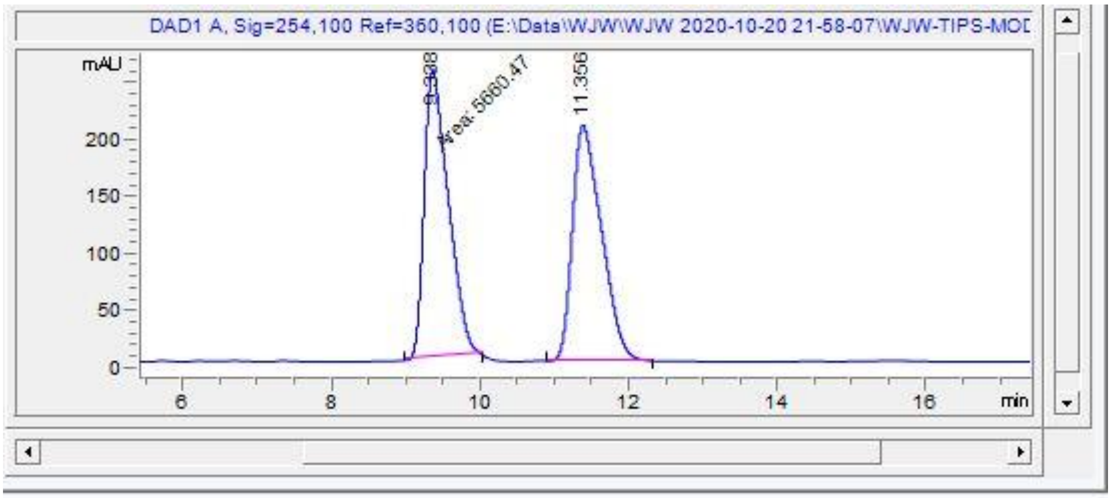

#	Time	Type	Area	Height	Width	Area%	Symmetry
1	9.338	MM	5660.5	253.2	0.3725	50.064	0.528
2	11.356	BV R	5645.9	207.9	0.3188	49.936	0.548

Supplementary Fig. 24 HPLC spectrum of chiral **3a.**

(*S*,*E*)-N-(4-acetyl-4-(p-tolyl)-6-(triisopropylsilyl)hex-1-en-5-yn-1-yl)-N-phenylpivalamide (**3b**)

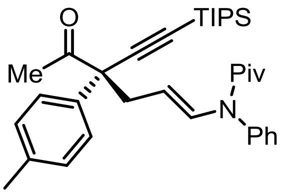

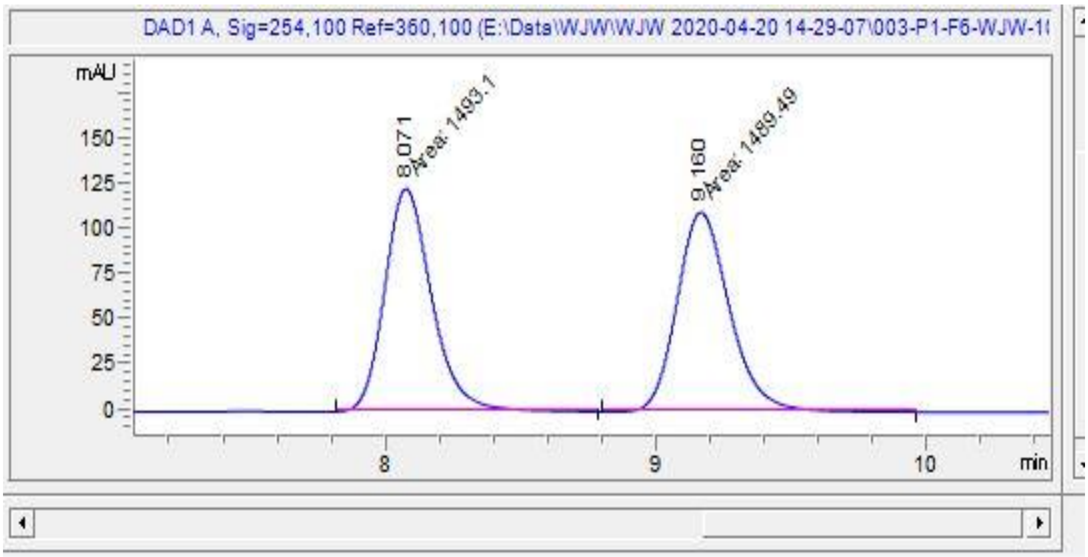

#	Time	Type	Area	Height	Width	Area%	Symmetry
1	8.071	MM	1493.1	123.1	0.2022	50.060	0.798
2	9.16	MM	1489.5	110.1	0.2255	49.940	0.797

Supplementary Fig. 25 HPLC spectrum of racemic **3b.**

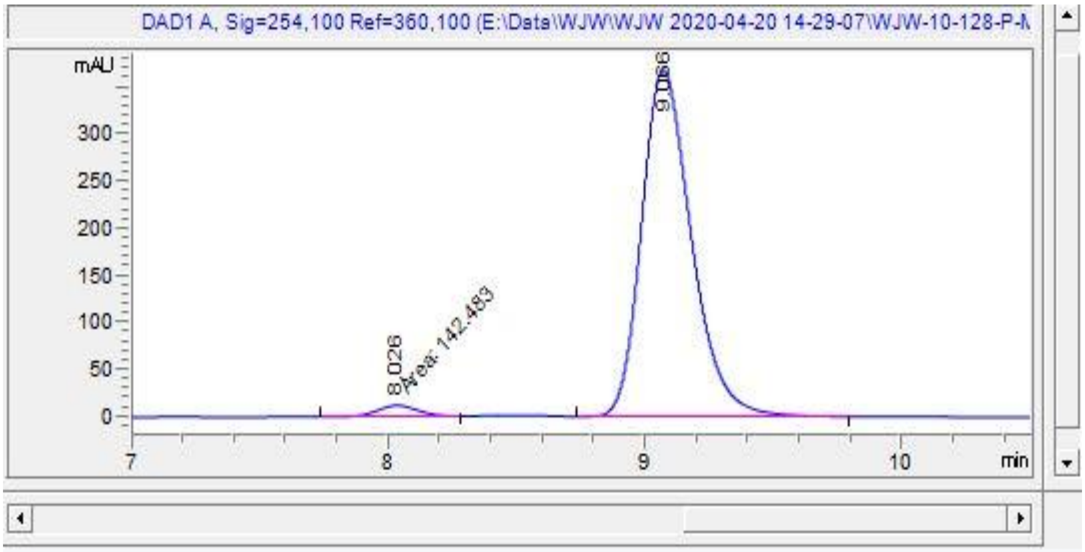

#	Time	Type	Area	Height	Width	Area%	Symmetry
1	8.026	MF	142.5	12.1	0.1957	2.791	0.88
2	9.066	VB	4962.6	367.6	0.2085	97.209	0.719

Supplementary Fig. 26 HPLC spectrum of chiral **3b.**

(*S*,*E*)-N-(4-acetyl-4-(4-chlorophenyl)-6-(triisopropylsilyl)hex-1-en-5-yn-1-yl)-N-phenylpivalamide (**3c**)

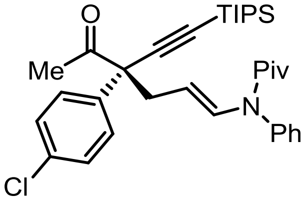

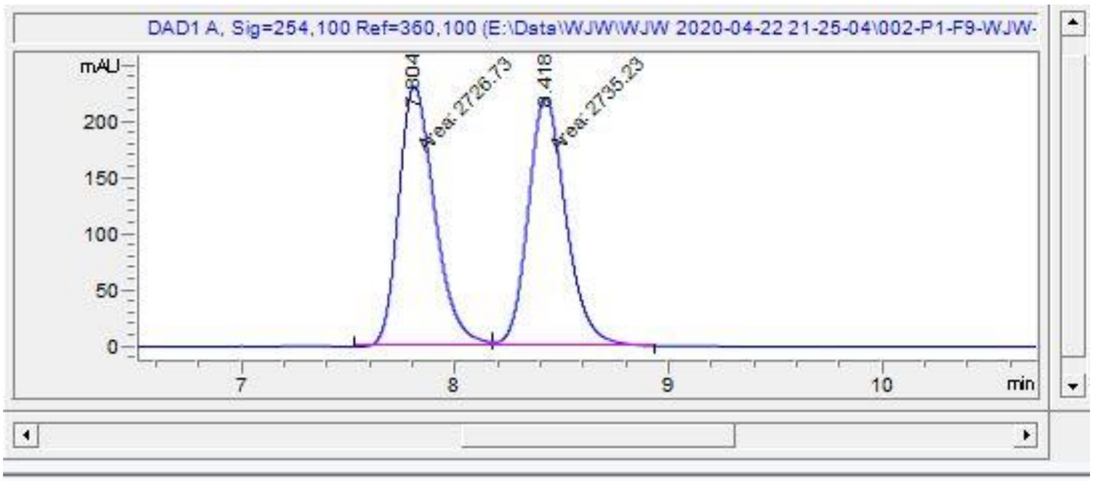

#	Time	Type	Area	Height	Width	Area%	Symmetry
1	7.804	MF	2726.7	231.6	0.1962	49.922	0
2	8.418	FM	2735.2	221.5	0.2058	50.078	0.804

Supplementary Fig. 27 HPLC spectrum of racemic **3c.**

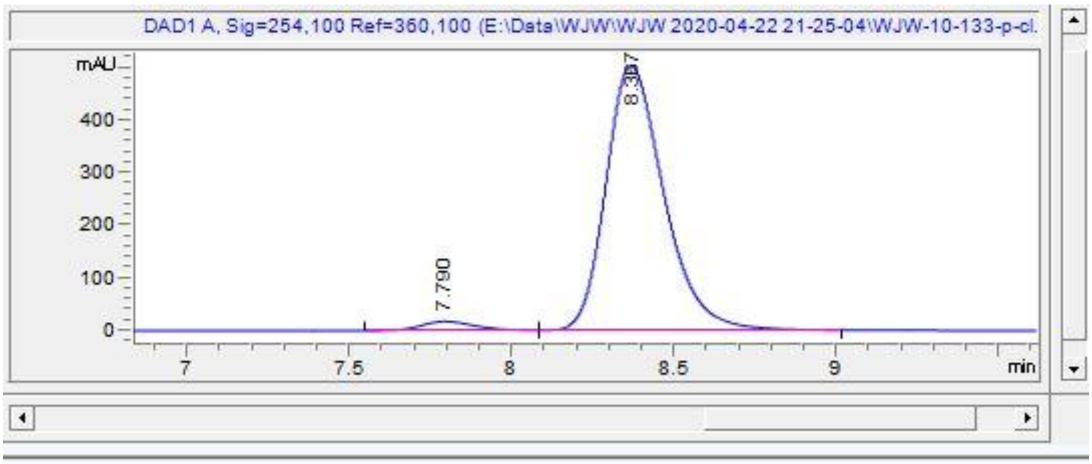

#	Time	Type	Area	Height	Width	Area%	Symmetry
1	7.79	BV	197.1	17.2	0.1731	3.067	0.754
2	8.367	VB	6229.1	501.3	0.1905	96.933	0.718

Supplementary Fig. 28 HPLC spectrum of chiral **3c.**

(*S*,*E*)-N-(4-acetyl-4-(4-(trifluoromethyl)phenyl)-6-(triisopropylsilyl)hex-1-en-5-yn-1-yl)-N-phenyl pivalamide (**3d**)

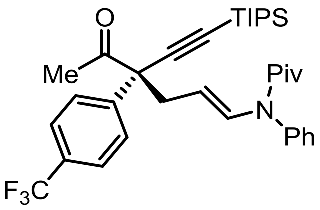

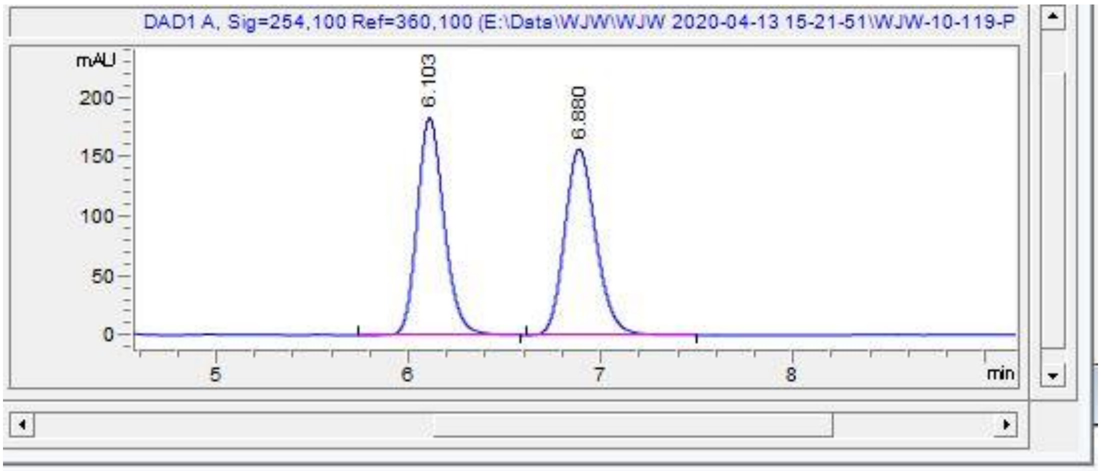

#	Time	Type	Area	Height	Width	Area%	Symmetry
1	6.103	BB	1770.2	183.1	0.1493	50.095	0.804
2	6.88	BB	1763.5	156.9	0.1721	49.905	0.819

Supplementary Fig. 29 HPLC spectrum of racemic **3d.**

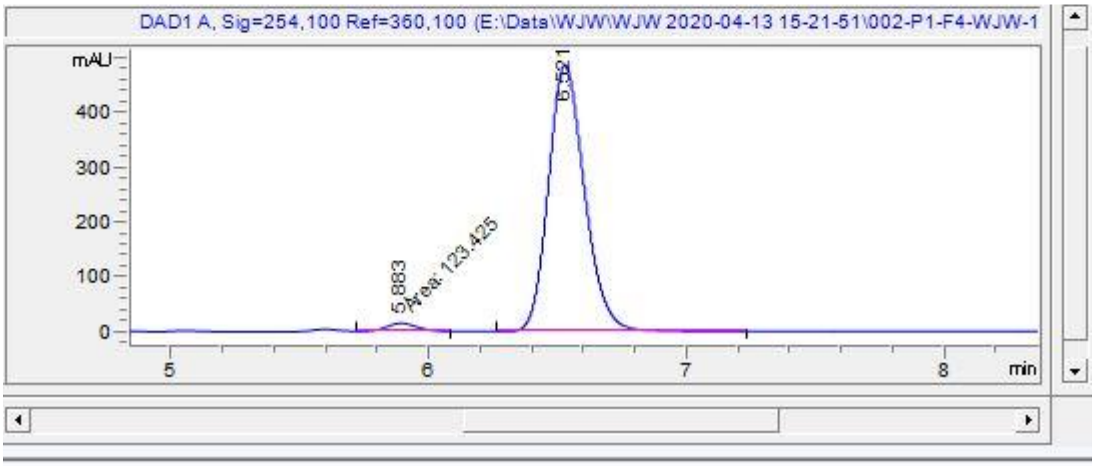

#	Time	Type	Area	Height	Width	Area%	Symmetry
1	5.883	MM	123.4	14.8	0.1389	2.517	0.868
2	6.521	BB	4781	489.4	0.1505	97.483	0.792

Supplementary Fig. 30 HPLC spectrum of chiral **3d.**

(*S*,*E*)-N-(4-acetyl-4-(m-tolyl)-6-(triisopropylsilyl)hex-1-en-5-yn-1-yl)-N-phenylpivalamide (**3e**)

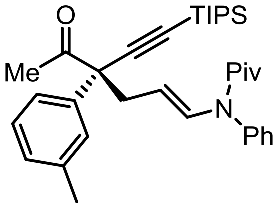

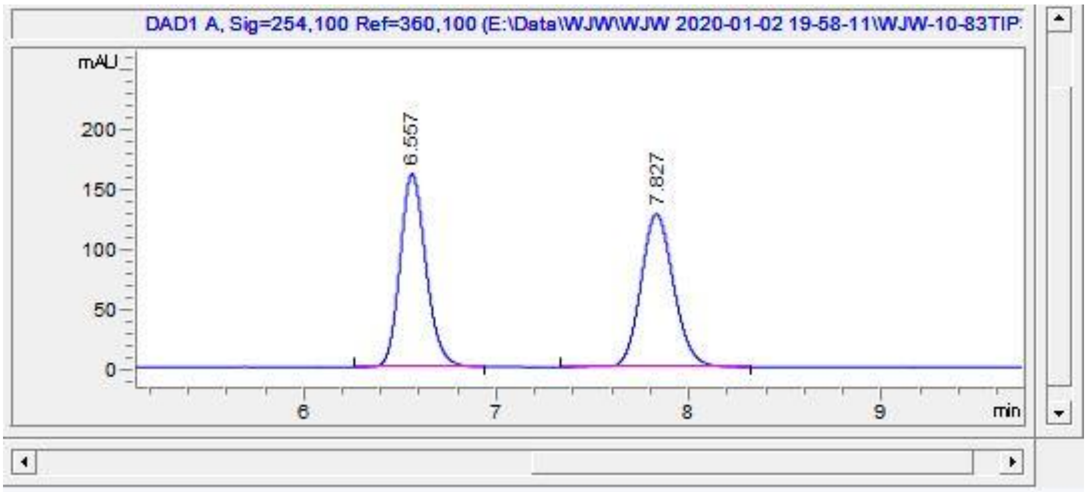

#	Time	Type	Area	Height	Width	Area%	Symmetry
1	6.557	BB	1513.7	161.1	0.1451	49.912	0.823
2	7.827	BB	1519.1	127.8	0.1832	50.088	0.867

Supplementary Fig. 31 HPLC spectrum of racemic **3e.**

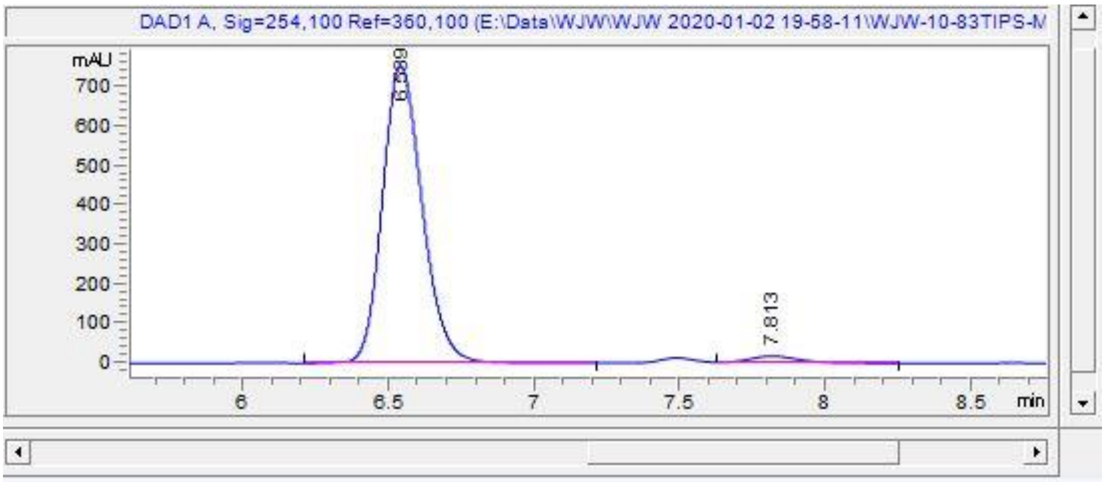

#	Time	Type	Area	Height	Width	Area%	Symmetry
1	6.539	BB	7207.4	756.8	0.1471	97.141	0.797
2	7.813	VB	212.1	17.7	0.172	2.859	0.864

Supplementary Fig. 32 HPLC spectrum of chiral **3e.**

(*S*,*E*)-N-(4-acetyl-4-(3-chlorophenyl)-6-(triisopropylsilyl)hex-1-en-5-yn-1-yl)-N-phenylpivalamide (**3f**)

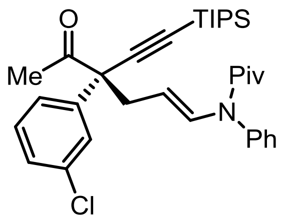

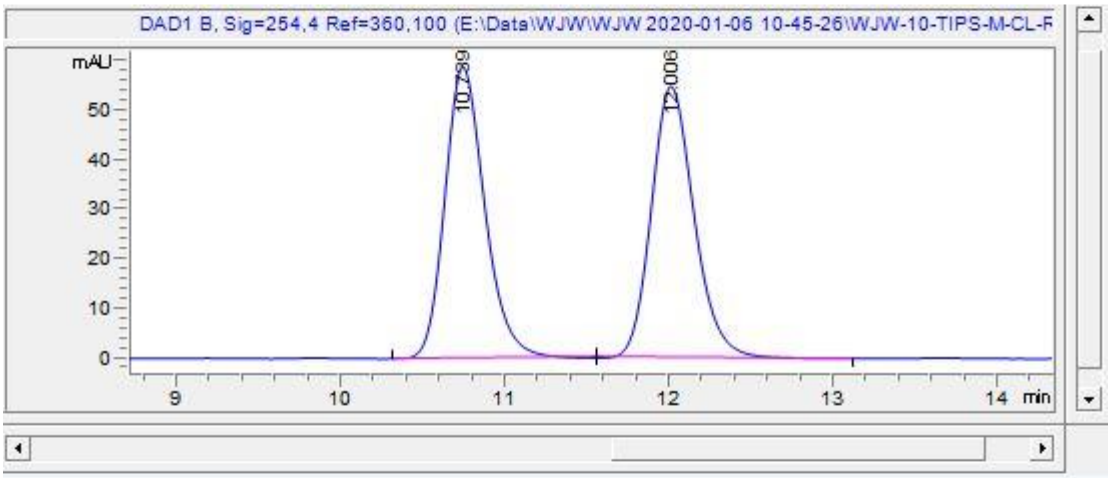

#	Time	Type	Area	Height	Width	Area%	Symmetry
1	10.739	BB	1001.3	59.1	0.2598	49.995	0.777
2	12.006	BB	1001.5	55.1	0.2781	50.005	0.804

Supplementary Fig. 33 HPLC spectrum of racemic **3f.**

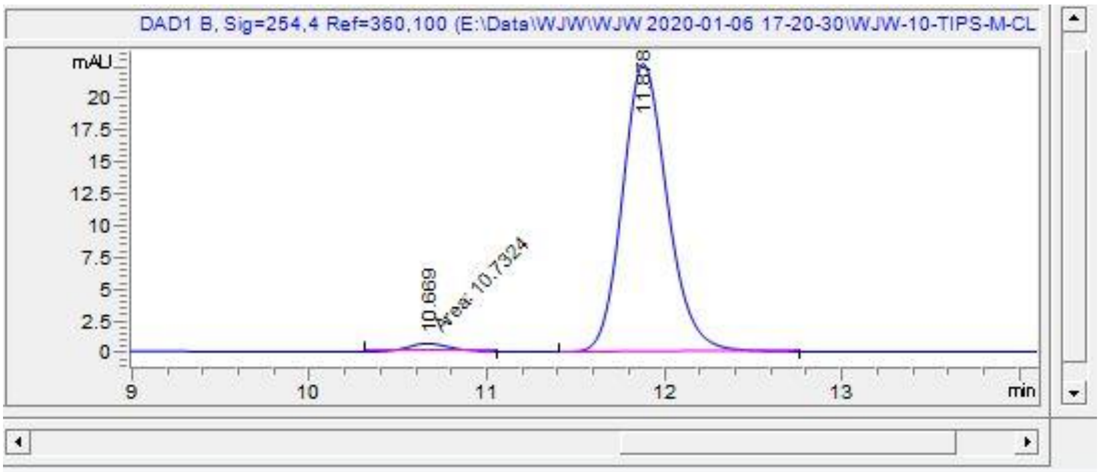

#	Time	Type	Area	Height	Width	Area%	Symmetry
1	10.669	MM	10.7	6.4E-1	0.2809	2.640	0.924
2	11.878	BB	395.7	22.5	0.2714	97.360	0.824

Supplementary Fig. 34 HPLC spectrum of chiral **3f.**

(*S*,*E*)-N-(4-acetyl-4-(3-fluorophenyl)-6-(triisopropylsilyl)hex-1-en-5-yn-1-yl)-N-phenylpivalamid e (**3g**)

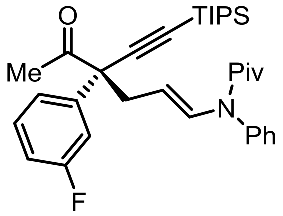

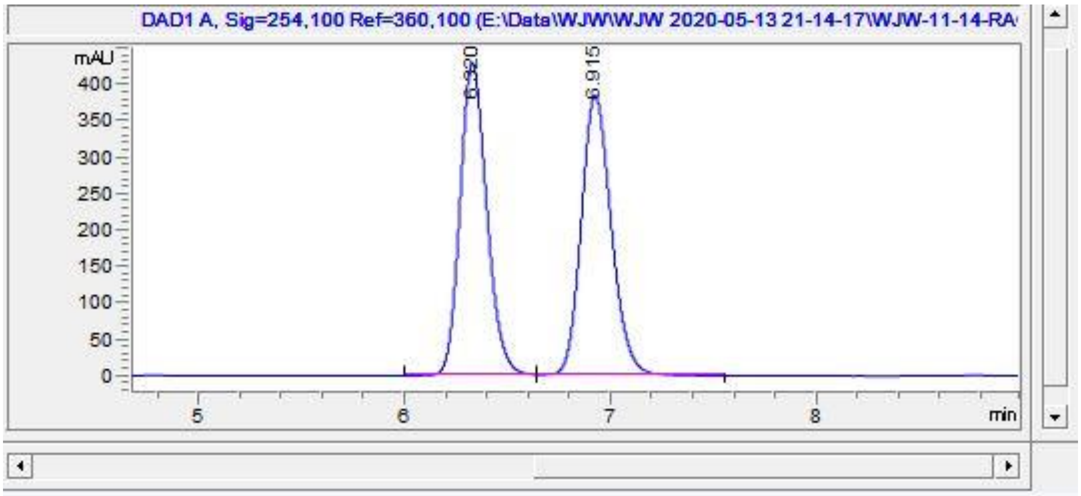

#	Time	Type	Area	Height	Width	Area%	Symmetry
1	6.32	BV	3888.2	429.3	0.1397	49.775	0.812
2	6.915	VB	3923.3	384.8	0.1554	50.225	0.816

Supplementary Fig. 35 HPLC spectrum of racemic **3g.**

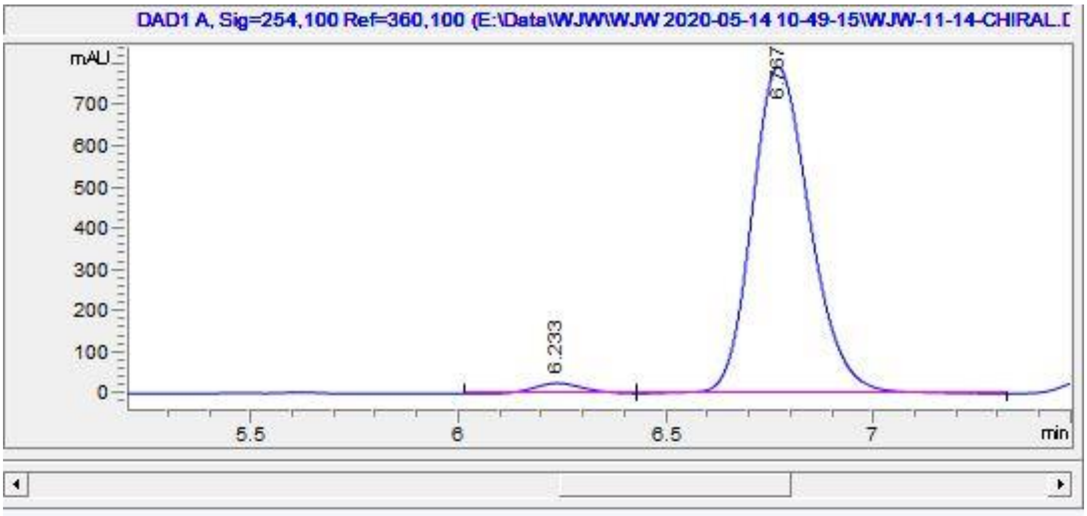

#	Time	Type	Area	Height	Width	Area%	Symmetry
1	6.233	BV	217.4	25.5	0.1326	2.713	0.85
2	6.767	VV	7795.7	800	0.1507	97.287	0.78

Supplementary Fig. 36 HPLC spectrum of chiral **3g.**

(*S*,*E*)-N-(4-acetyl-4-(3-methoxyphenyl)-6-(triisopropylsilyl)hex-1-en-5-yn-1-yl)-N-phenylpivalamide (**3h**)

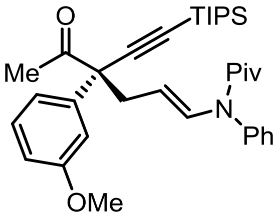

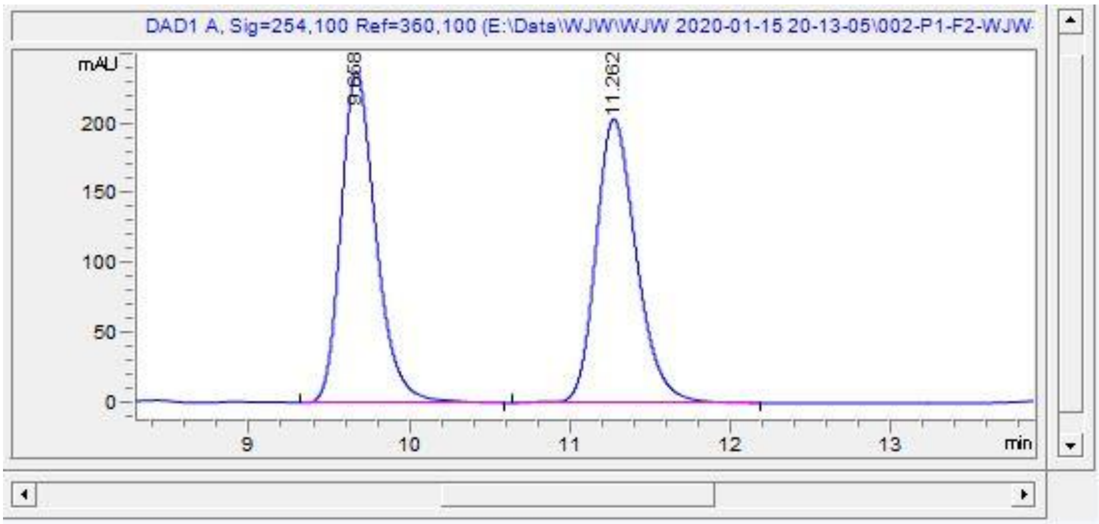

#	Time	Type	Area	Height	Width	Area%	Symmetry
1	9.658	BB	3604.1	237.7	0.2319	50.224	0.723
2	11.262	BB	3571.9	203.7	0.2661	49.776	0.763

Supplementary Fig. 37 HPLC spectrum of racemic **3h.**

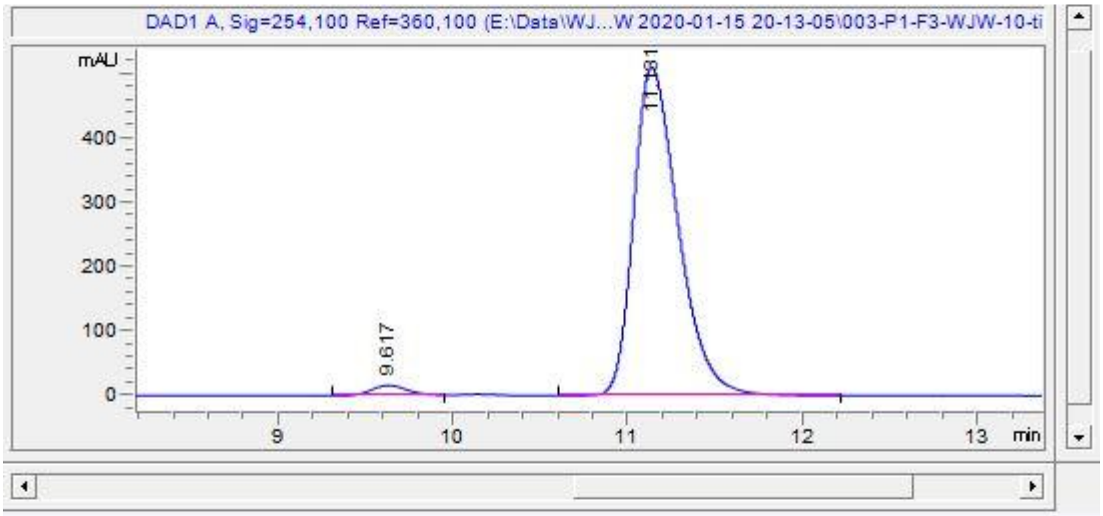

#	Time	Type	Area	Height	Width	Area%	Symmetry
1	9.617	BB	220.6	15.7	0.1804	2.365	0.832
2	11.131	BB	9107.7	512.4	0.2549	97.635	0.648

Supplementary Fig. 38 HPLC spectrum of chiral **3h.**

(*S*,*E*)-N-(4-acetyl-4-(naphthalen-2-yl)-6-(triisopropylsilyl)hex-1-en-5-yn-1-yl)-N-phenylpivalamid e (**3i**)

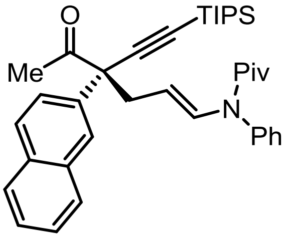

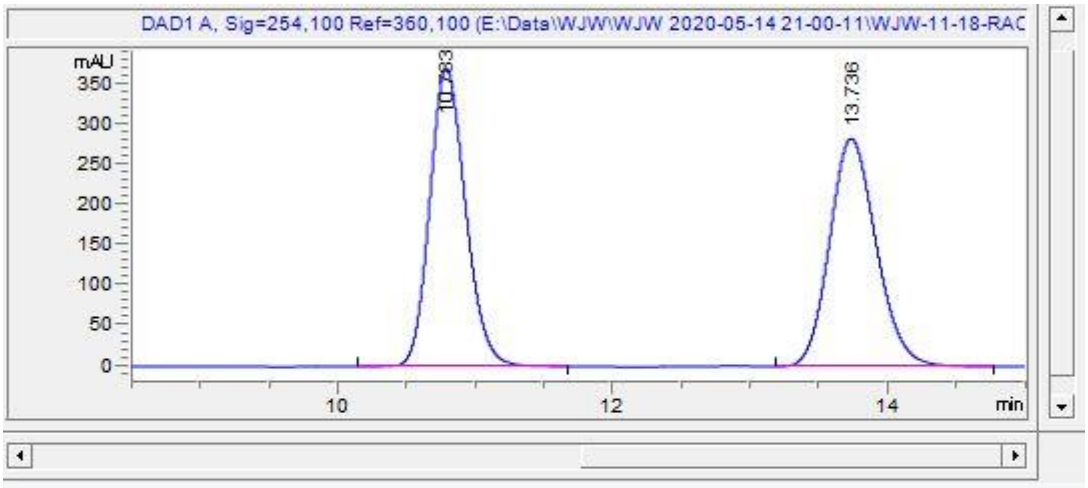

#	Time	Type	Area	Height	Width	Area%	Symmetry
1	10.783	BB	6796.3	372.7	0.2783	50.165	0.819
2	13.736	BB	6751.7	284.9	0.3479	49.835	0.832

Supplementary Fig. 39 HPLC spectrum of racemic **3i.**

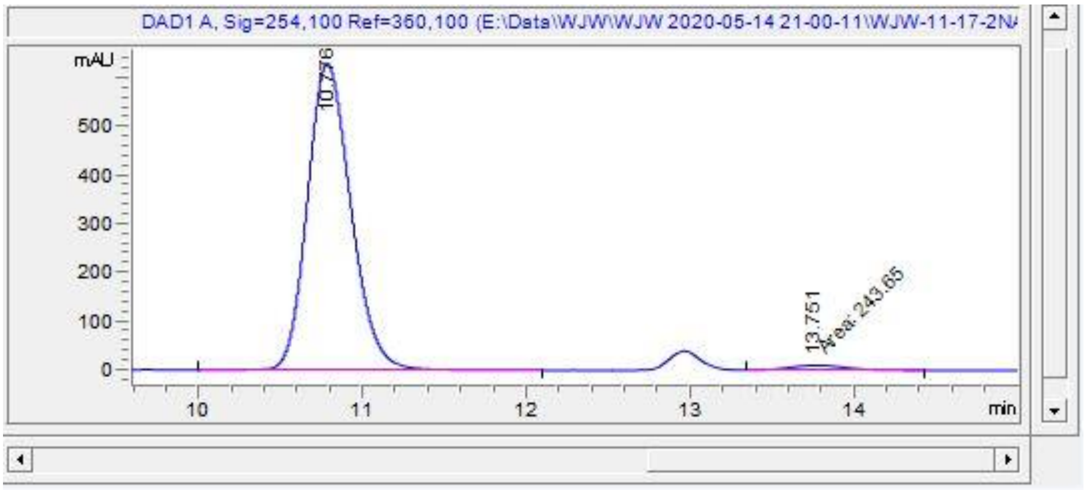

#	Time	Type	Area	Height	Width	Area%	Symmetry
1	10.776	BB	11591.9	628.2	0.2878	97.941	0.792
2	13.751	MM	243.7	10.3	0.3948	2.059	0.891

Supplementary Fig. 40 HPLC spectrum of chiral **3i.**

(*R*,*E*)-N-(4-acetyl-4-methyl-6-(triisopropylsilyl)hex-1-en-5-yn-1-yl)-N-phenylpivalamide (**3j**)

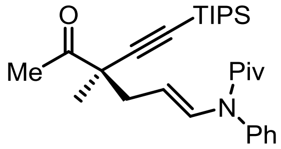

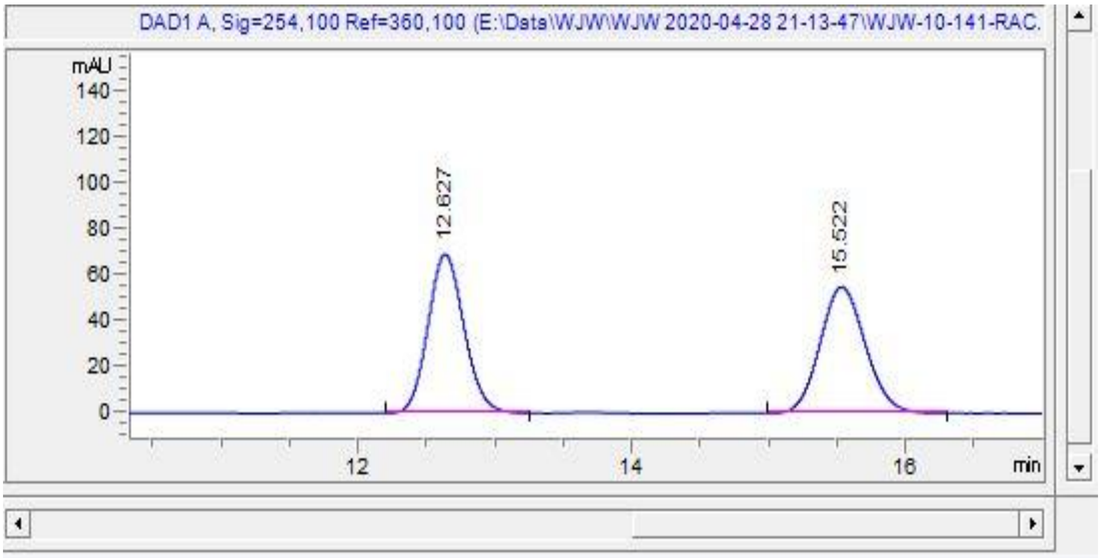

#	Time	Type	Area	Height	Width	Area%	Symmetry
1	12.627	BB	1281.1	69.9	0.2672	50.051	0.87
2	15.522	BB	1278.5	55.6	0.2868	49.949	0.892

Supplementary Fig. 41 HPLC spectrum of racemic **3j.**

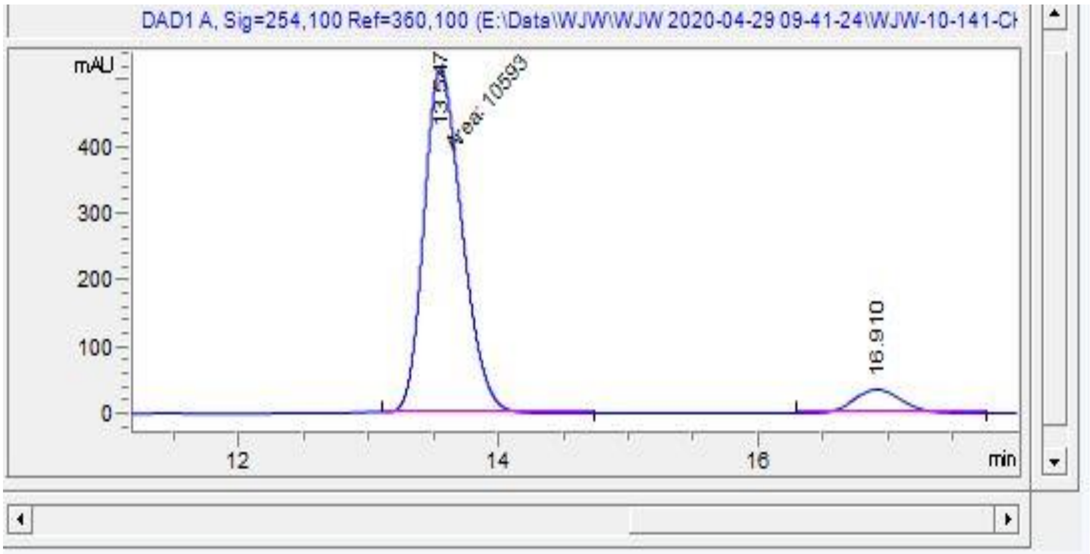

#	Time	Type	Area	Height	Width	Area%	Symmetry
1	13.547	FM	10593	513.8	0.3436	92.074	0.729
2	16.91	BB	911.9	35.5	0.3783	7.926	0.884

Supplementary Fig. 42 HPLC spectrum of chiral **3j.**

(*S*,*E*)-N-(4-acetyl-4-phenyl-6-(p-tolyl)hex-1-en-5-yn-1-yl)-N-phenylpivalamide (**3k**)

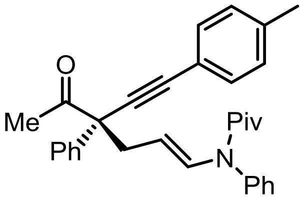

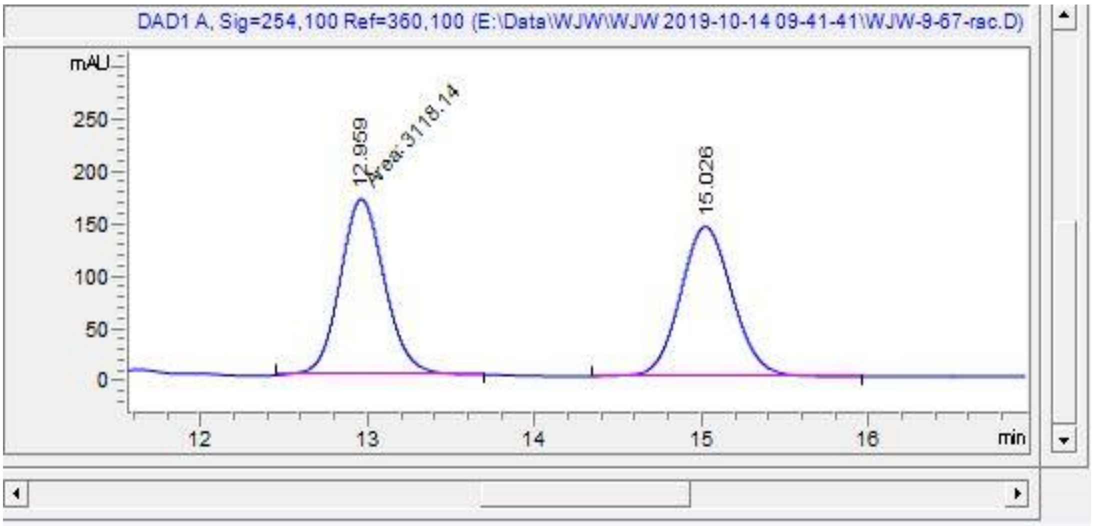

#	Time	Type	Area	Height	Width	Area%	Symmetry
1	12.959	MM	3118.1	167.4	0.3104	50.195	0.895
2	15.026	BB	3094	142.5	0.3333	49.805	0.899

Supplementary Fig. 43 HPLC spectrum of racemic **3k.**

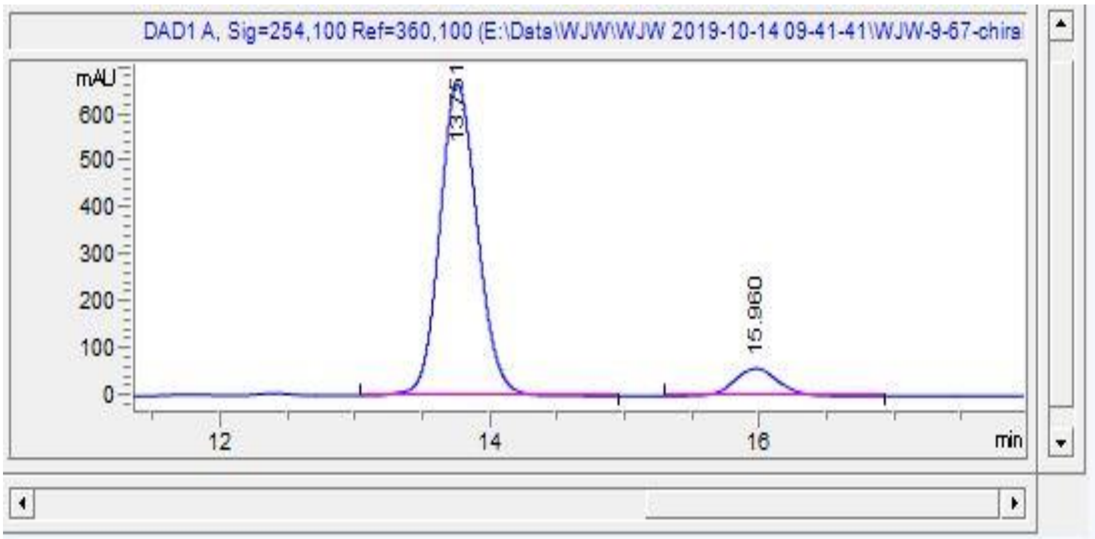

#	Time	Type	Area	Height	Width	Area%	Symmetry
1	13.751	BB	13023.9	671.2	0.2839	90.661	0.862
2	15.96	BB	1341.6	59.2	0.3179	9.339	0.903

Supplementary Fig. 44 HPLC spectrum of chiral **3k.**

(R,E)-N-(4-acetyl-4-ethyl-6-phenylhex-1-en-5-yn-1-yl)-N-phenylpivalamide (**3l**)

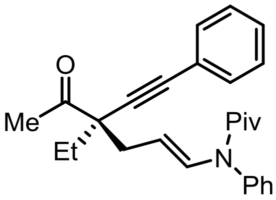

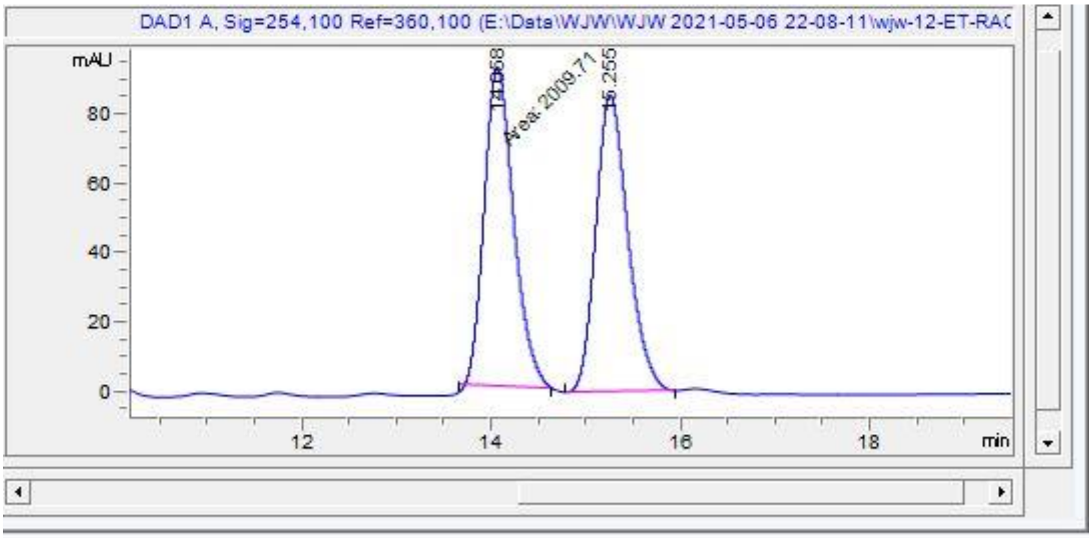

#	Time	Type	Area	Height	Width	Area%	Symmetry
1	14.058	MM	2009.7	92.4	0.3623	49.886	0.762
2	15.255	BB	2018.9	85.7	0.3323	50.114	0.775

Supplementary Fig. 45 HPLC spectrum of racemic **3l.**

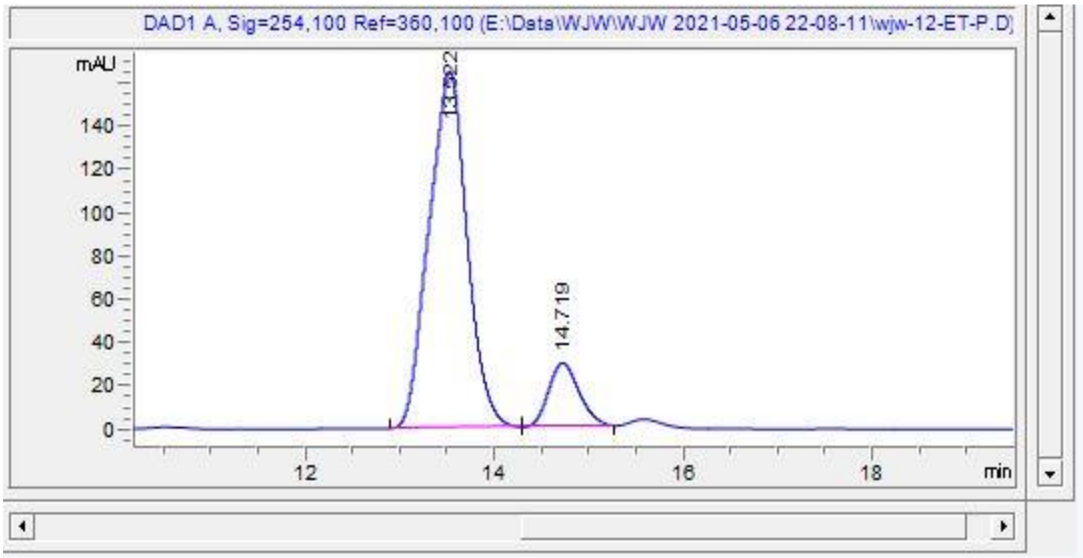

#	Time	Type	Area	Height	Width	Area%	Symmetry
1	13.522	BB	4639.5	165.1	0.3784	87.452	1.139
2	14.719	BB	665.7	29.5	0.2706	12.548	0.83

Supplementary Fig. 46 HPLC spectrum of chiral **3l.**

(*S*,*E*)-N-(4-acetyl-4-phenyldec-1-en-5-yn-1-yl)-N-phenylpivalamide (**3m**)

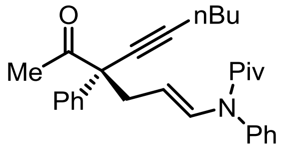

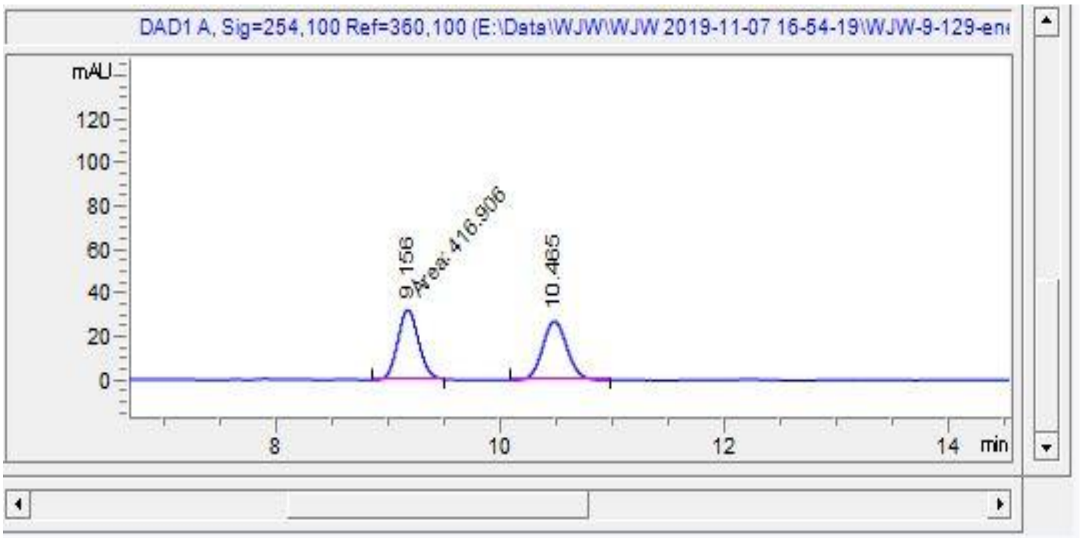

#	Time	Type	Area	Height	Width	Area%	Symmetry
1	9.156	MM	416.9	32.3	0.2152	50.104	0.911
2	10.465	BB	415.2	27.3	0.2242	49.896	0.887

Supplementary Fig. 47 HPLC spectrum of racemic **3m.**

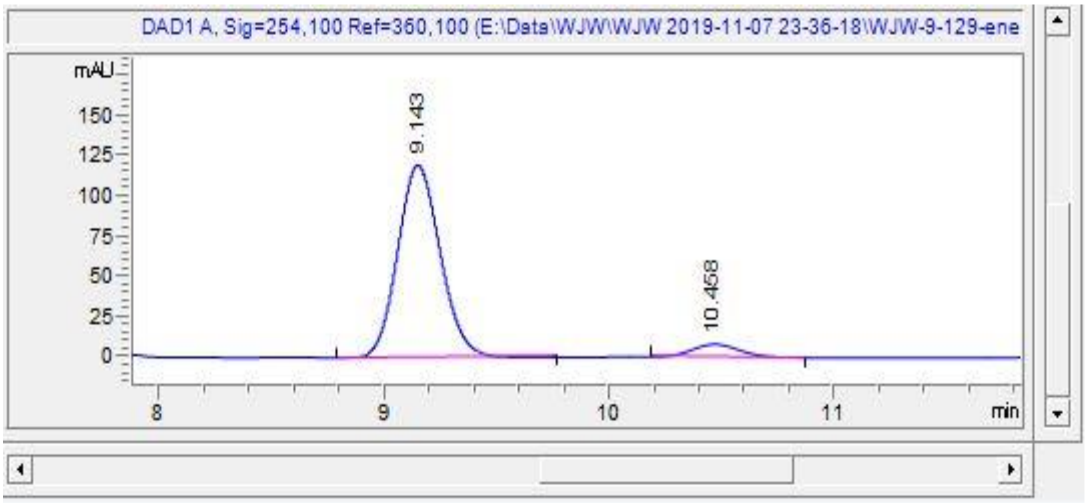

#	Time	Type	Area	Height	Width	Area%	Symmetry
1	9.143	BB	1578.7	119.8	0.2037	93.103	0.862
2	10.458	BB	117	8	0.1834	6.897	0.897

Supplementary Fig. 48 HPLC spectrum of chiral **3m.**

(*R*,*E*)-N-(4-acetyl-4-phenylhex-1-en-5-yn-1-yl)-N-phenylpivalamide (**3n**)

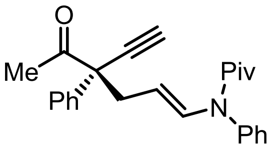

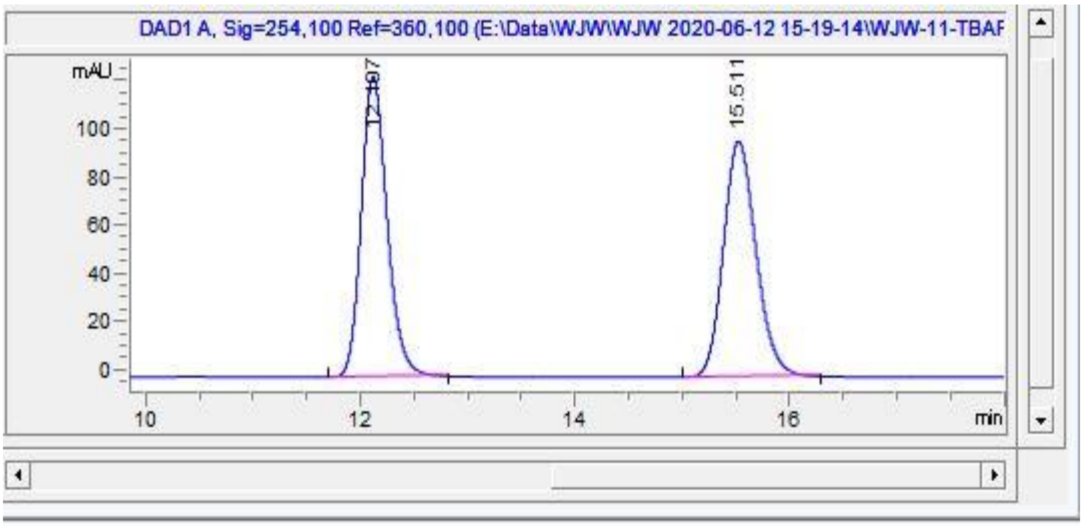

#	Time	Type	Area	Height	Width	Area%	Symmetry
1	12.107	BB	2080.7	125.3	0.2387	49.921	0.786
2	15.511	BB	2087.2	98.5	0.3023	50.079	0.813

Supplementary Fig. 49 HPLC spectrum of racemic **3n.**

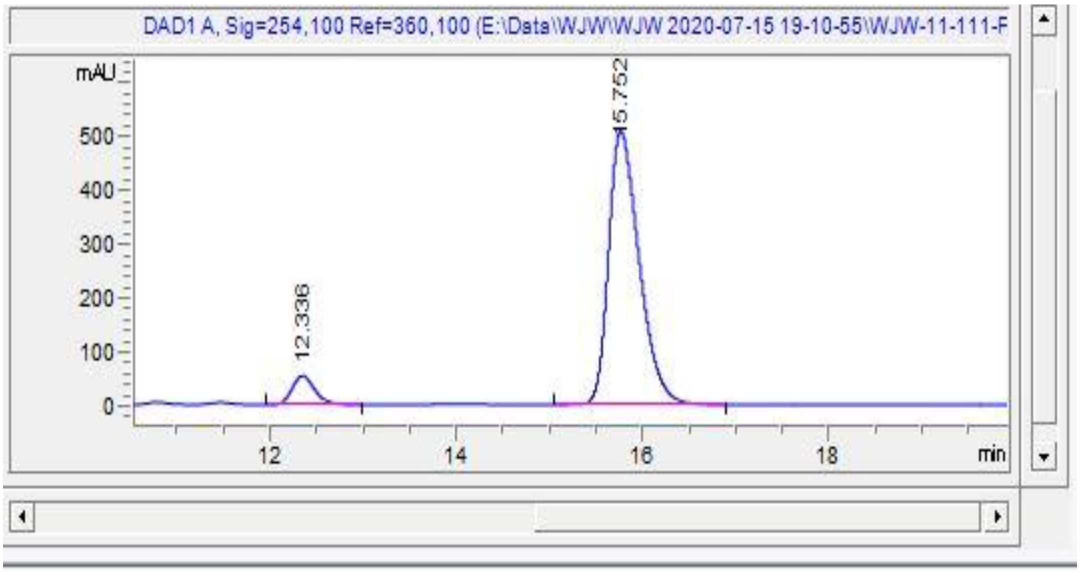

#	Time	Type	Area	Height	Width	Area%	Symmetry
1	12.336	BB	913.7	53.6	0.243	7.300	0.82
2	15.752	BB	11602.8	506.9	0.269	92.700	0.639

Supplementary Fig. 50 HPLC spectrum of chiral **3n.**

(*S*,*E*)-N-(5-oxo-4-phenyl-4-((triisopropylsilyl)ethynyl)hept-1-en-1-yl)-N-phenylpivalamide (**3o**)

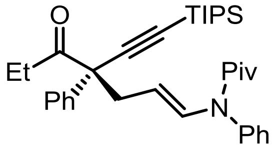

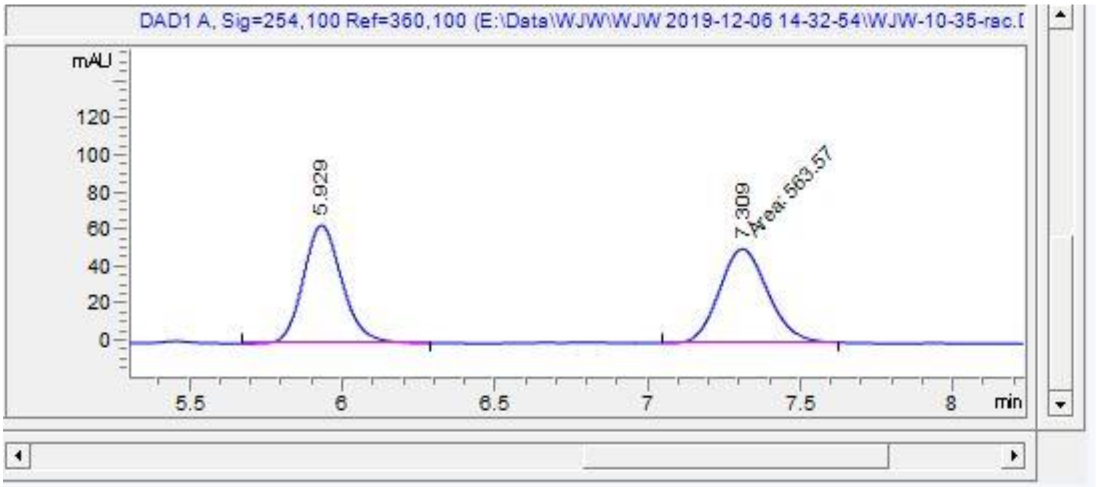

#	Time	Type	Area	Height	Width	Area%	Symmetry
1	5.929	BB	563.7	63.8	0.1361	50.005	0.844
2	7.309	MM	563.6	50.8	0.1849	49.995	0.878

Supplementary Fig. 51 HPLC spectrum of racemic **3o.**

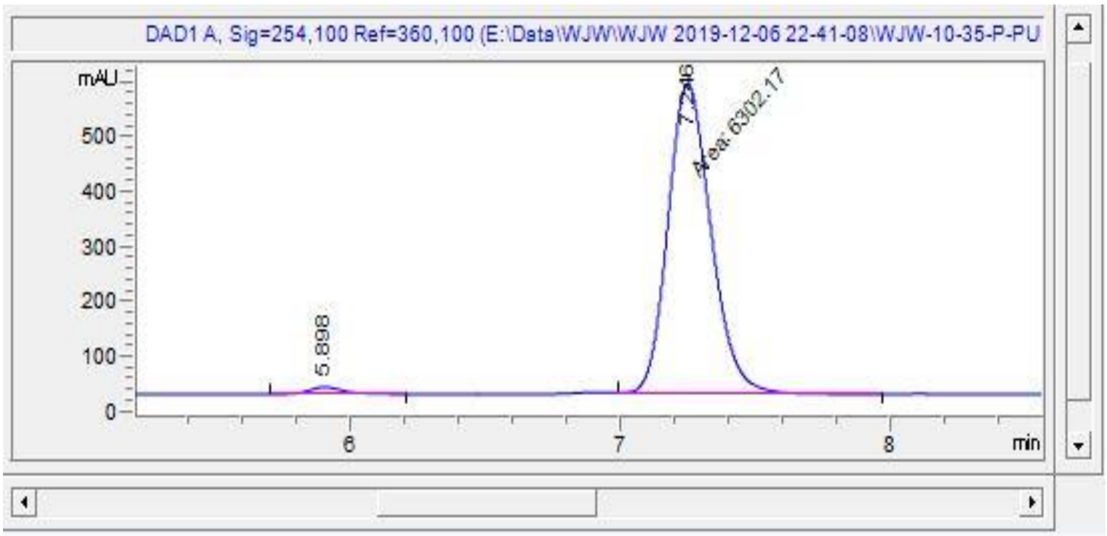

#	Time	Type	Area	Height	Width	Area%	Symmetry
1	5.898	BB	110.8	12.9	0.1314	1.728	0.757
2	7.246	FM	6302.2	566.5	0.1854	98.272	0.815

Supplementary Fig. 52 HPLC spectrum of chiral **3o.**

(*S*,*E*)-N-(5-oxo-4-phenyl-4-((triisopropylsilyl)ethynyl)non-1-en-1-yl)-N-phenylpivalamide (**3p**)

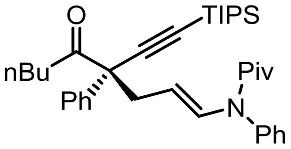

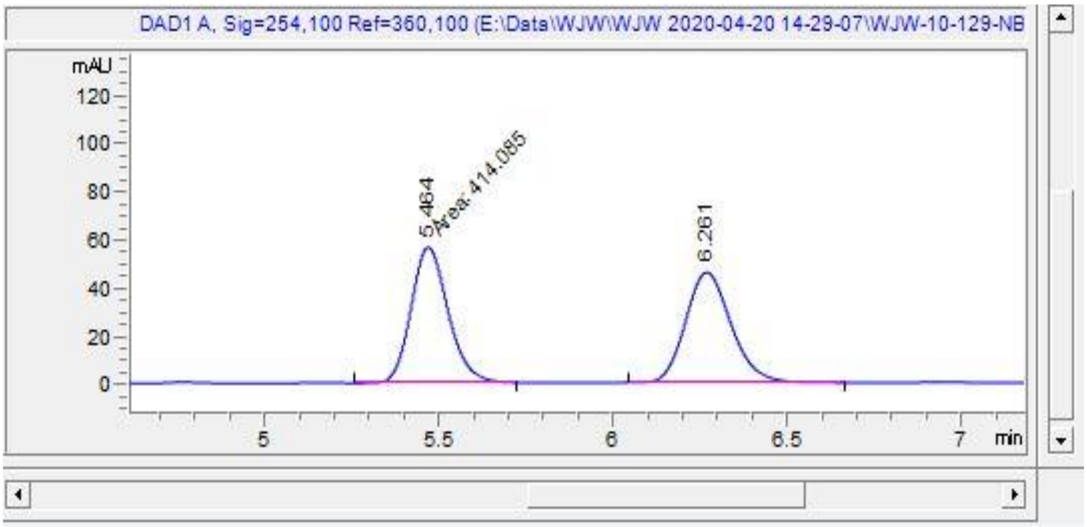

#	Time	Type	Area	Height	Width	Area%	Symmetry
1	5.464	MM	414.1	56.3	0.1226	50.025	0.825
2	6.261	BB	413.7	45.8	0.1399	49.975	0.826

Supplementary Fig. 53 HPLC spectrum of racemic **3p.**

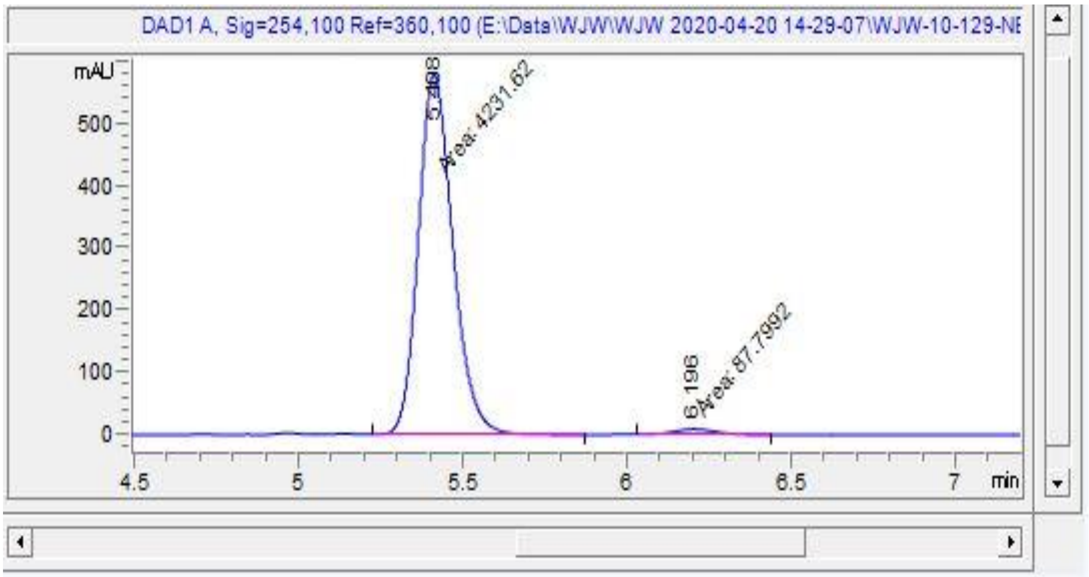

#	Time	Type	Area	Height	Width	Area%	Symmetry
1	5.408	FM	4231.6	576.4	0.1224	97.967	0.8
2	6.196	FM	87.8	9.4	0.1556	2.033	0.918

Supplementary Fig. 54 HPLC spectrum of chiral **3p.**

(*S*,*E*)-N-(6-methyl-5-oxo-4-phenyl-4-((triisopropylsilyl)ethynyl)hept-1-en-1-yl)-N-phenylpivalamide (**3q**)

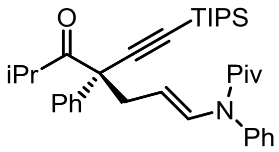

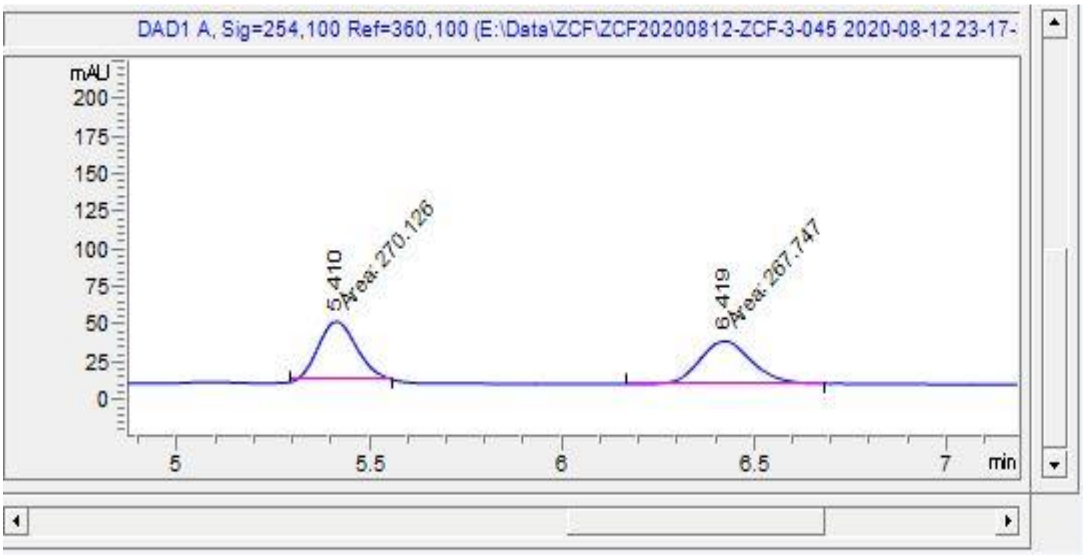

#	Time	Type	Area	Height	Width	Area%	Symmetry
1	5.41	MM	270.1	38.9	0.1157	50.221	0.822
2	6.419	MM	267.7	28.5	0.1568	49.779	0.864

Supplementary Fig. 55 HPLC spectrum of racemic **3q.**

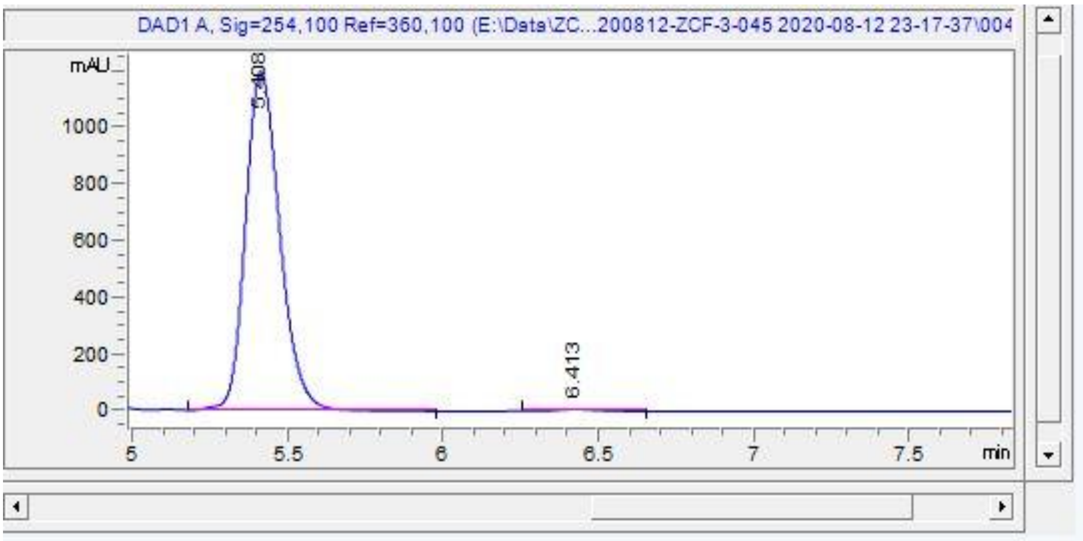

#	Time	Type	Area	Height	Width	Area%	Symmetry
1	5.408	BB	9166.2	1197.5	0.1133	99.428	0.769
2	6.413	BB	52.8	6.3	0.1003	0.572	0.799

Supplementary Fig. 56 HPLC spectrum of chiral **3q.**

(*S*,*E*)-N-(4-acetyl-4-phenyl-6-(triisopropylsilyl)hex-1-en-5-yn-1-yl)-N-(p-tolyl)pivalamide (**3r**)

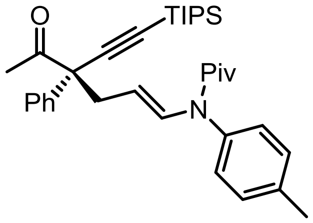

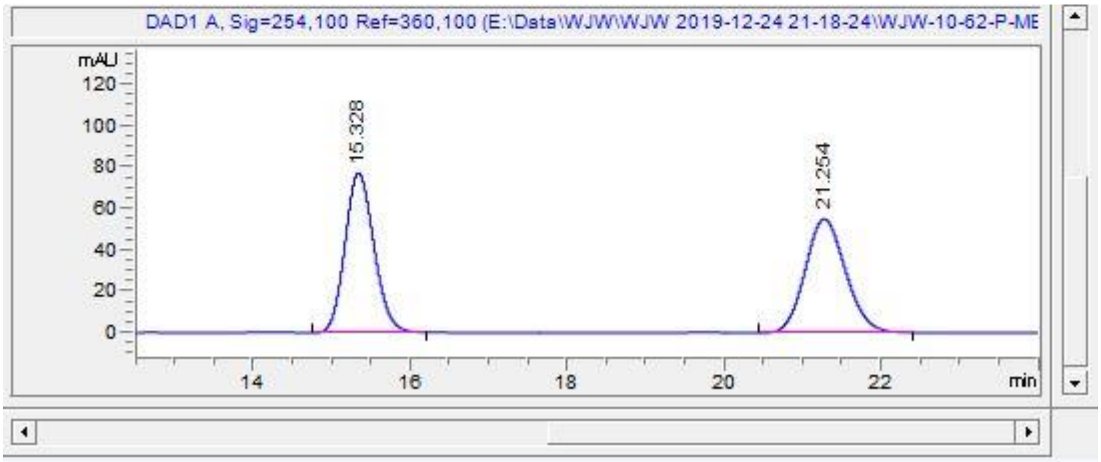

#	Time	Type	Area	Height	Width	Area%	Symmetry
1	15.328	BB	1958.4	76.8	0.3669	50.021	0.845
2	21.254	BB	1956.8	54.7	0.4245	49.979	0.856

Supplementary Fig. 57 HPLC spectrum of racemic **3r.**

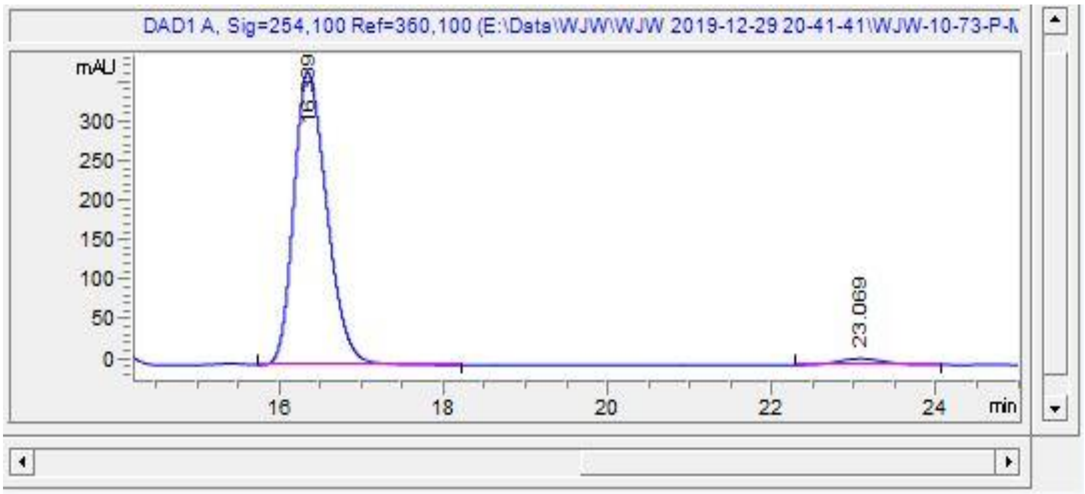

#	Time	Type	Area	Height	Width	Area%	Symmetry
1	16.339	BB	10468.3	371.9	0.4279	97.151	0.708
2	23.069	BB	307	7.9	0.4559	2.849	0.937

Supplementary Fig. 58 HPLC spectrum of chiral **3r.**

(*S*,*E*)-N-(4-acetyl-4-phenyl-6-(triisopropylsilyl)hex-1-en-5-yn-1-yl)-N-(4-methoxyphenyl)pivalamide (**3s**)

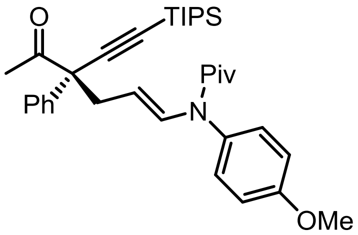

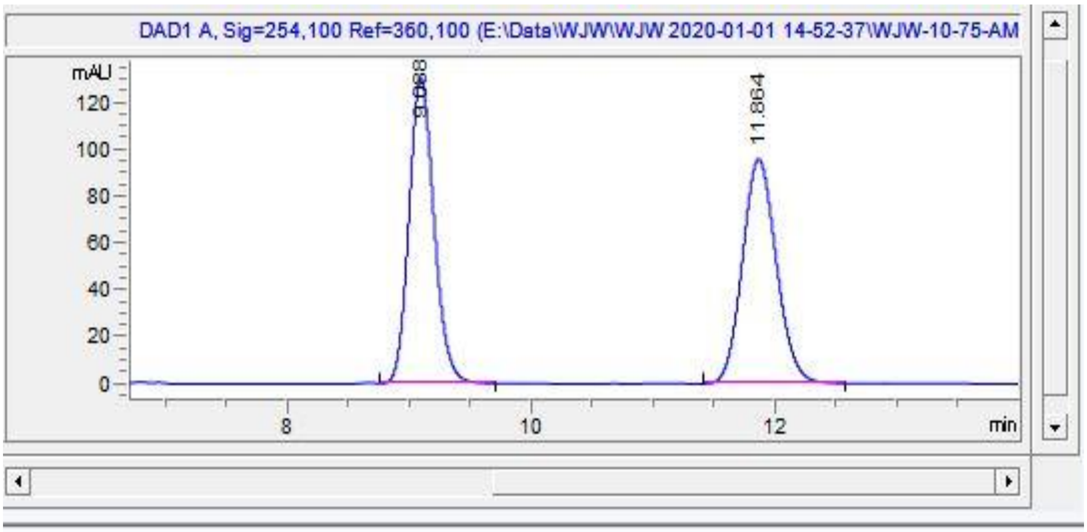

#	Time	Type	Area	Height	Width	Area%	Symmetry
1	9.088	BB	1865.9	130.9	0.2188	49.961	0.833
2	11.864	BB	1868.9	96.1	0.2938	50.039	0.877

Supplementary Fig. 59 HPLC spectrum of racemic **3s.**

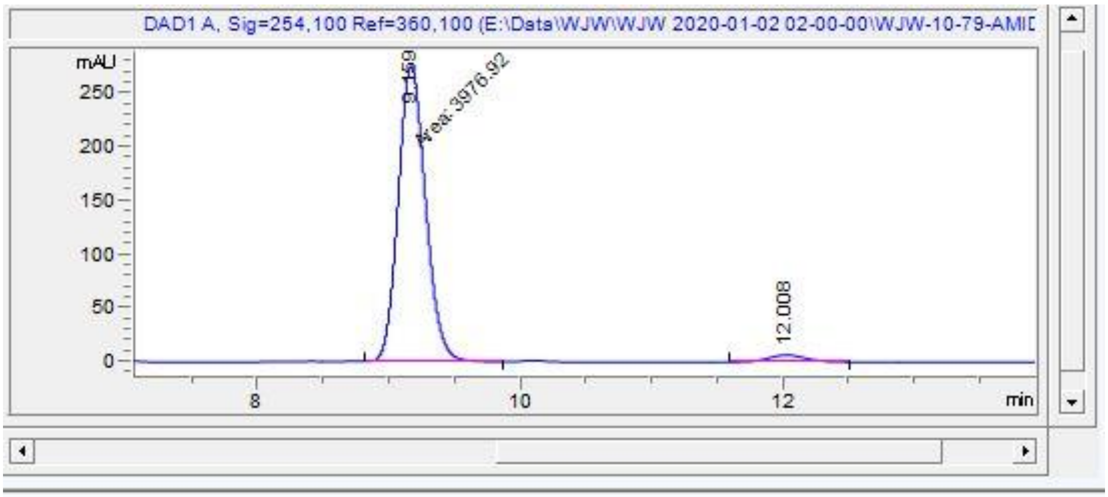

#	Time	Type	Area	Height	Width	Area%	Symmetry
1	9.159	FM	3976.9	274.8	0.2412	96.865	0.848
2	12.008	BB	128.7	6.5	0.2317	3.135	0.906

Supplementary Fig. 60 HPLC spectrum of chiral **3s.**

(*S*,*E*)-N-(4-acetyl-4-phenyl-6-(triisopropylsilyl)hex-1-en-5-yn-1-yl)-N-(4-chlorophenyl)pivalamide (**3t**)

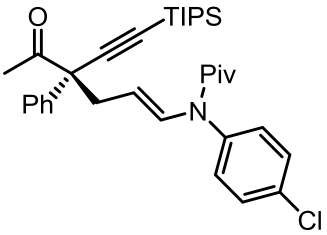

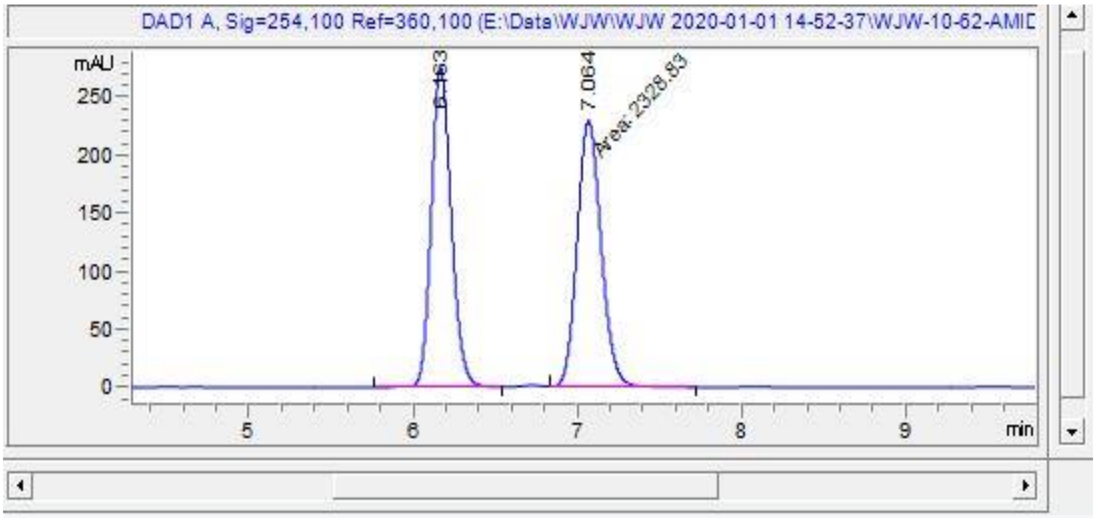

#	Time	Type	Area	Height	Width	Area%	Symmetry
1	6.163	BB	2335.3	275.2	0.1315	50.069	0.838
2	7.064	FM	2328.8	229.3	0.1693	49.931	0.855

Supplementary Fig. 61 HPLC spectrum of racemic **3t.**

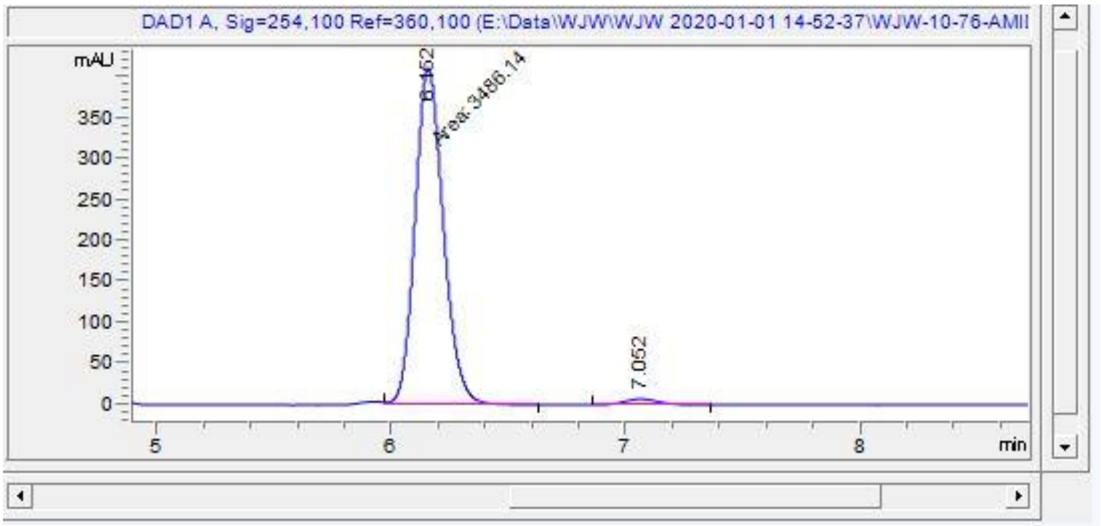

#	Time	Type	Area	Height	Width	Area%	Symmetry
1	6.152	FM	3486.1	412.1	0.141	97.960	0.839
2	7.052	BB	72.6	7.3	0.1265	2.040	0.839

Supplementary Fig. 62 HPLC spectrum of chiral **3t.**

(*S*,*E*)-N-(4-acetyl-4-phenyl-6-(triisopropylsilyl)hex-1-en-5-yn-1-yl)-N-(4-(trifluoromethyl)phenyl) pivalamide (**3u**)

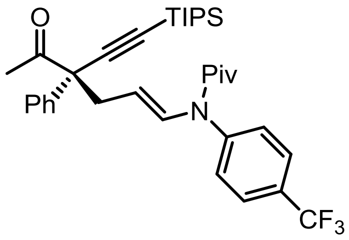

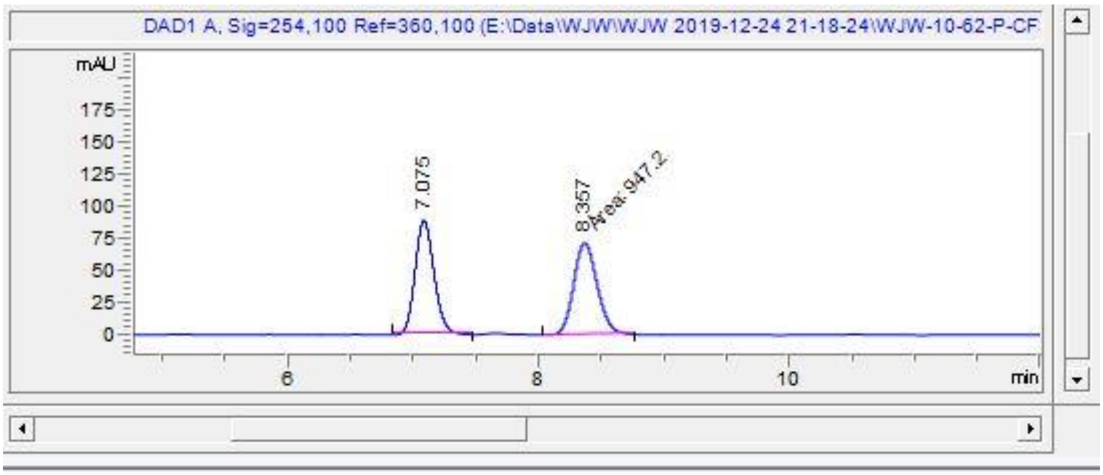

#	Time	Type	Area	Height	Width	Area%	Symmetry
1	7.075	BB	946.4	90.2	0.1619	49.979	0.831
2	8.357	MM	947.2	72.4	0.218	50.021	0.86

Supplementary Fig. 63 HPLC spectrum of racemic **3u.**

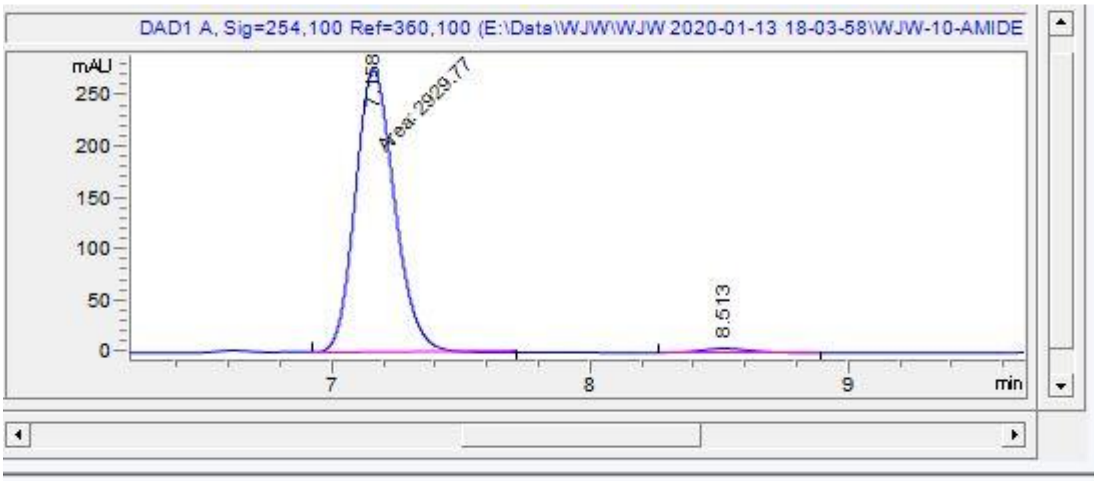

#	Time	Type	Area	Height	Width	Area%	Symmetry
1	7.158	FM	2929.8	275.4	0.1773	98.147	0.822
2	8.513	BB	55.3	4.2	0.1554	1.853	0.833

Supplementary Fig. 64 HPLC spectrum of chiral **3u.**

(*S*,*E*)-N-(4-acetyl-4-phenyl-6-(triisopropylsilyl)hex-1-en-5-yn-1-yl)-N-(3-methoxyphenyl)pivalamide (**3v**)

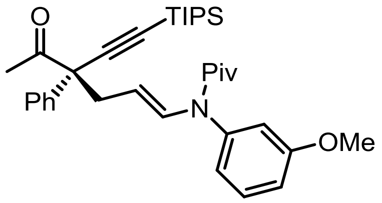

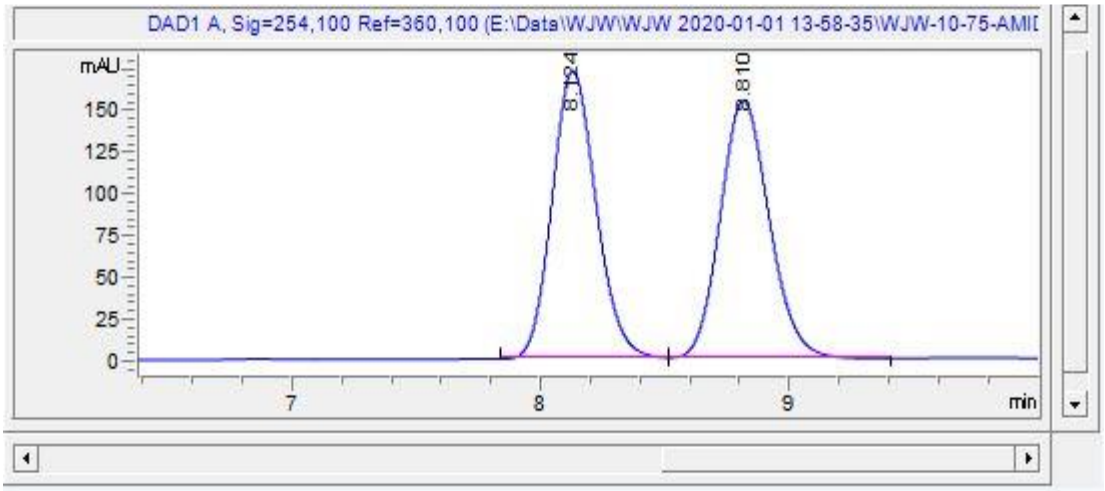

#	Time	Type	Area	Height	Width	Area%	Symmetry
1	8.124	BV	2142.9	173.5	0.1916	49.980	0.844
2	8.81	VB	2144.6	155.5	0.2129	50.020	0.85

Supplementary Fig. 65 HPLC spectrum of racemic **3v.**

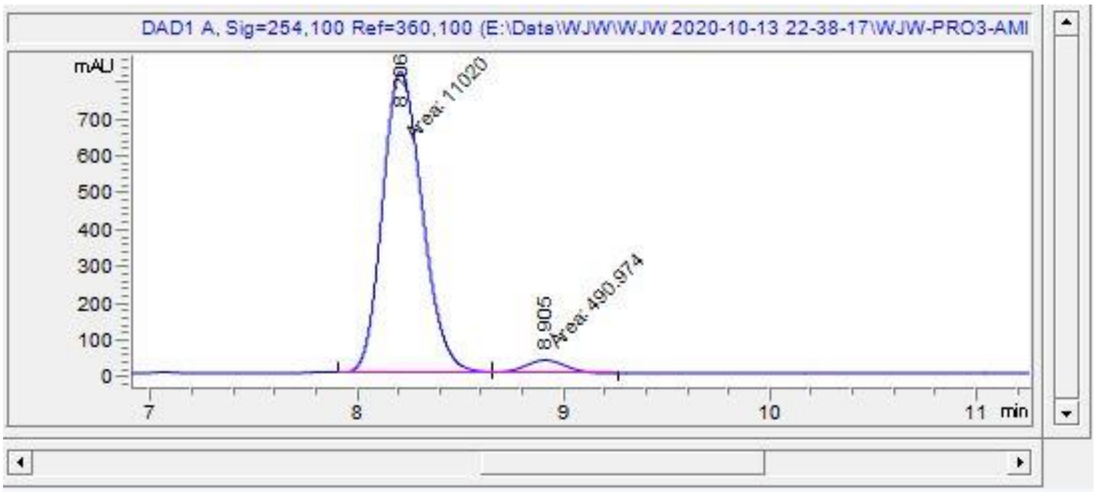

#	Time	Type	Area	Height	Width	Area%	Symmetry
1	8.206	FM	11020	819.6	0.2241	95.735	0.84
2	8.905	FM	491	35.3	0.2319	4.265	1.086

Supplementary Fig. 66 HPLC spectrum of chiral **3v.**

(*S*,*E*)-N-(4-acetyl-4-phenyl-6-(triisopropylsilyl)hex-1-en-5-yn-1-yl)-N-(3-fluorophenyl)pivalamide (**3w**)

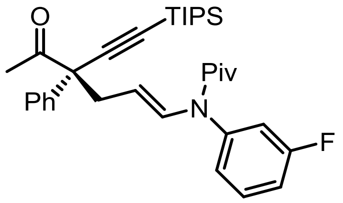

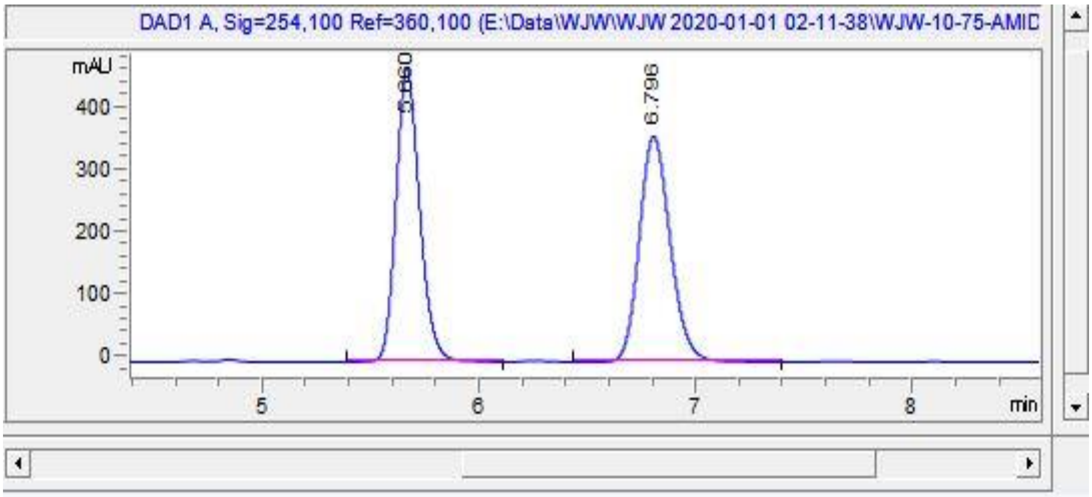

#	Time	Type	Area	Height	Width	Area%	Symmetry
1	5.66	BV	3568.6	469.4	0.1172	49.939	0.798
2	6.796	BB	3577.2	361	0.1526	50.061	0.82

Supplementary Fig. 67 HPLC spectrum of racemic **3w.**

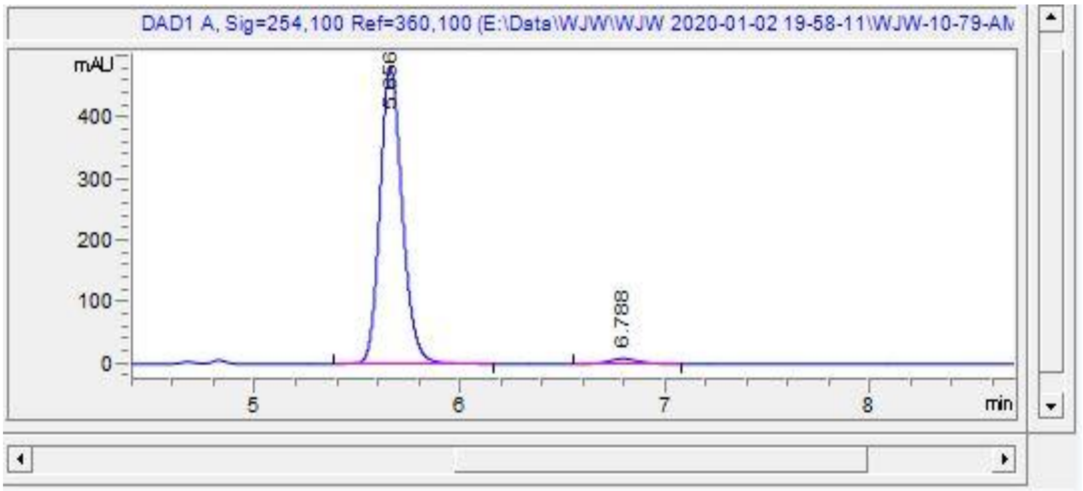

#	Time	Type	Area	Height	Width	Area%	Symmetry
1	5.656	BB	3642.9	480.5	0.1169	97.718	0.805
2	6.788	BB	85.1	8.6	0.1531	2.282	0.875

Supplementary Fig. 68 HPLC spectrum of chiral **3w.**

(*S*,*E*)-N-(4-acetyl-4-phenyl-6-(triisopropylsilyl)hex-1-en-5-yn-1-yl)-N-(naphthalen-2-yl)pivalamide (**3x**)

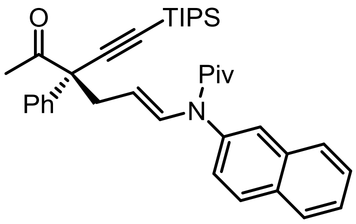

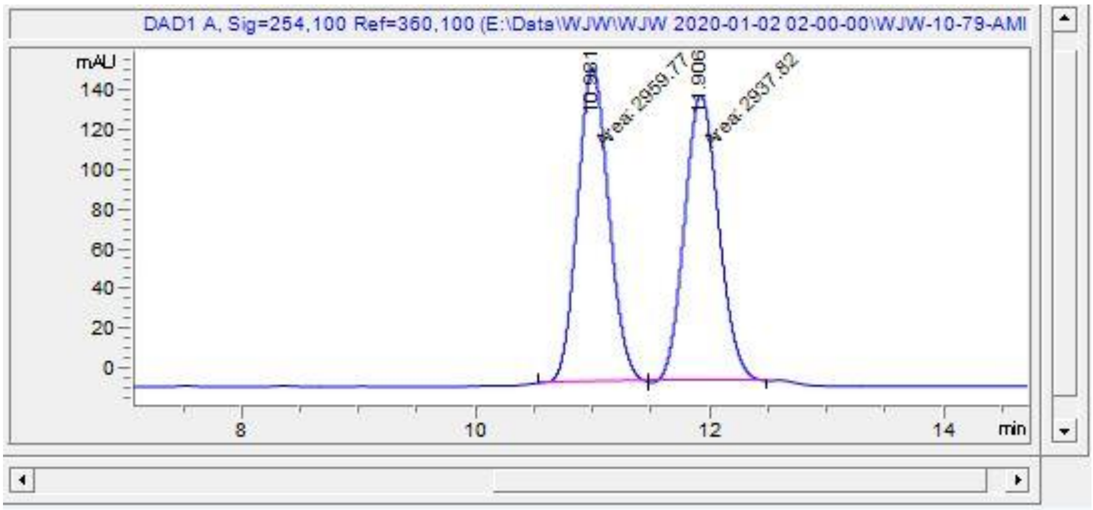

#	Time	Type	Area	Height	Width	Area%	Symmetry
1	10.981	MF	2959.8	158.4	0.3114	50.186	0.885
2	11.906	FM	2937.8	144.2	0.3396	49.814	0.881

Supplementary Fig. 69 HPLC spectrum of racemic **3x.**

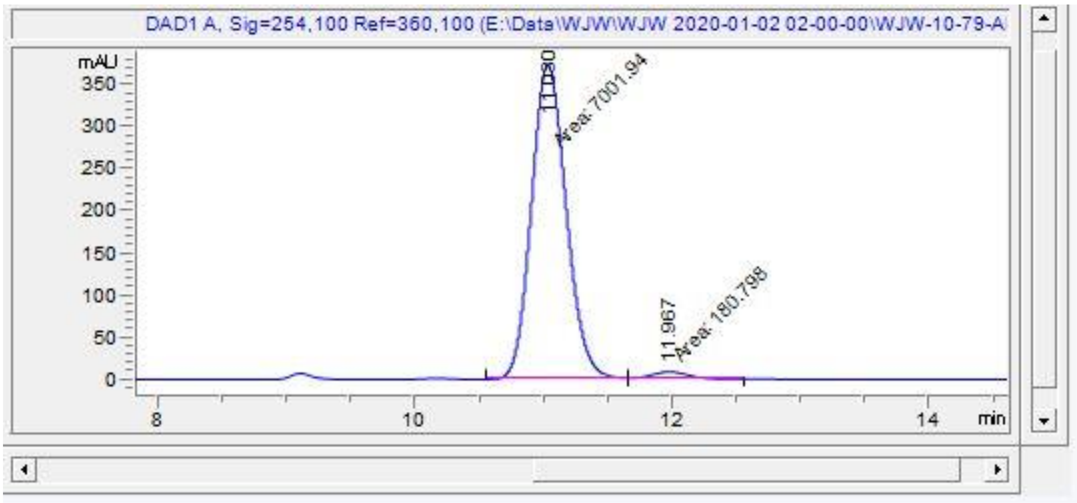

#	Time	Type	Area	Height	Width	Area%	Symmetry
1	11.03	MF	7001.9	370.5	0.315	97.483	0
2	11.967	FM	180.8	8.8	0.3444	2.517	0.92

Supplementary Fig. 70 HPLC spectrum of chiral **3x.**

(*S*,*E*)-N-(4-acetyl-4-phenyl-6-(triisopropylsilyl)hex-1-en-5-yn-1-yl)-N-phenylisobutyramide (**3y**)

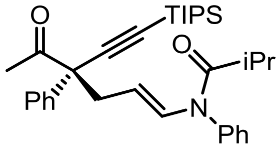

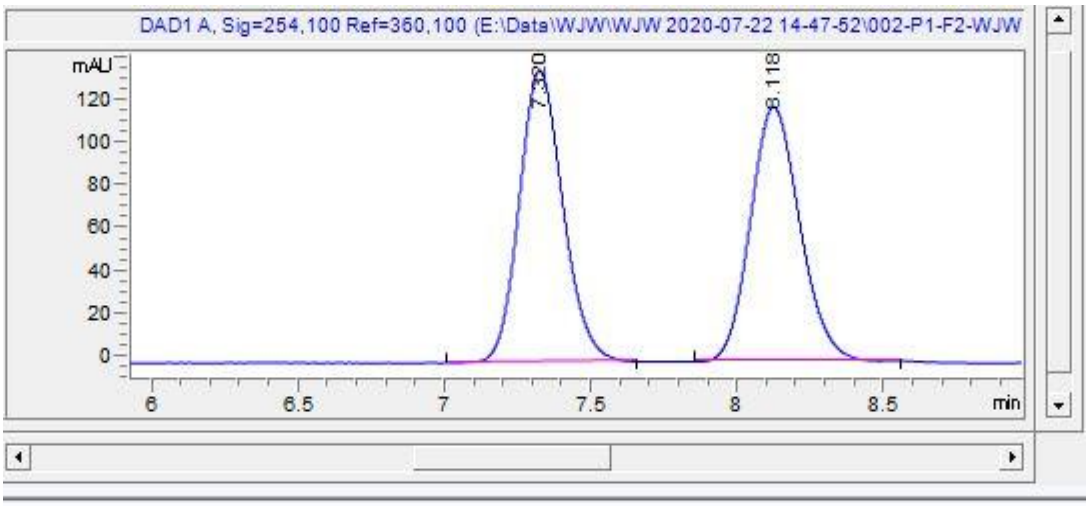

#	Time	Type	Area	Height	Width	Area%	Symmetry
1	7.32	BB	1420	136.6	0.1593	50.344	0.843
2	8.118	BV R	1400.6	119.3	0.177	49.656	0.852

Supplementary Fig. 71 HPLC spectrum of racemic **3y.**

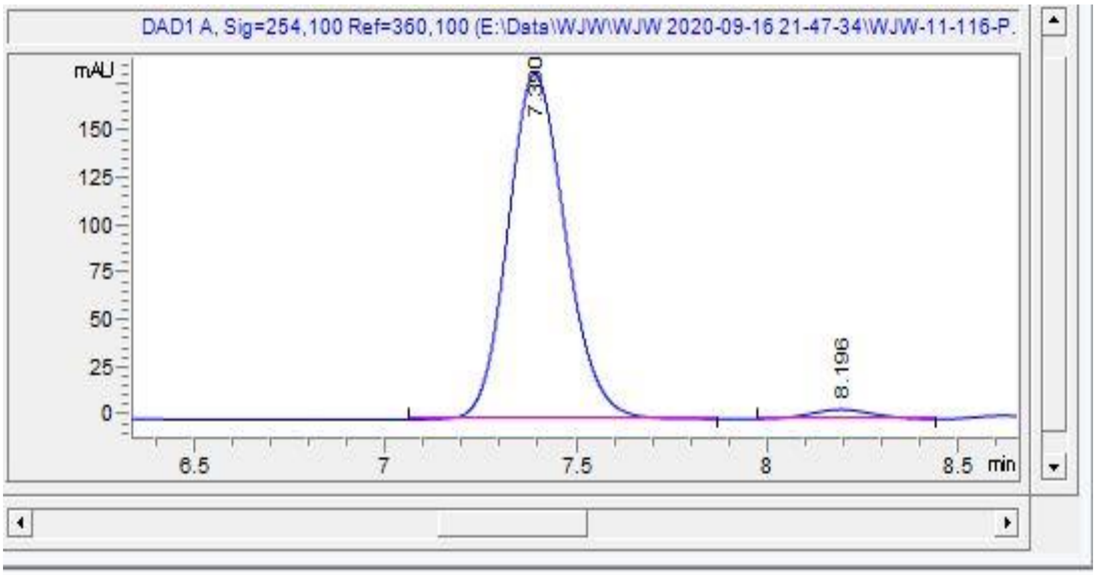

#	Time	Type	Area	Height	Width	Area%	Symmetry
1	7.39	BV R	1916.4	181.7	0.1615	97.106	0.832
2	8.196	BB	57.1	5	0.1383	2.894	0.998

Supplementary Fig. 72 HPLC spectrum of chiral **3y.**

(*S*,*E*)-N-(4-acetyl-4-phenyl-6-(triisopropylsilyl)hex-1-en-5-yn-1-yl)-N-phenylacetamide (**3z**)

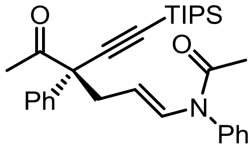

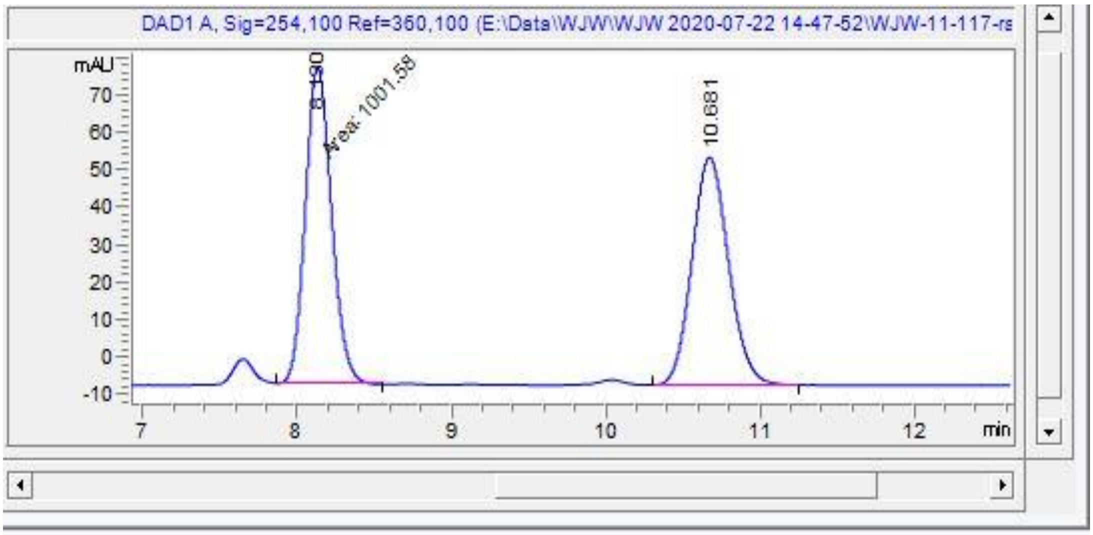

#	Time	Type	Area	Height	Width	Area%	Symmetry
1	8.13	FM	1001.6	84.2	0.1983	50.043	0.858
2	10.681	BV R	999.9	60.5	0.2035	49.957	0.931

Supplementary Fig. 73 HPLC spectrum of racemic **3z.**

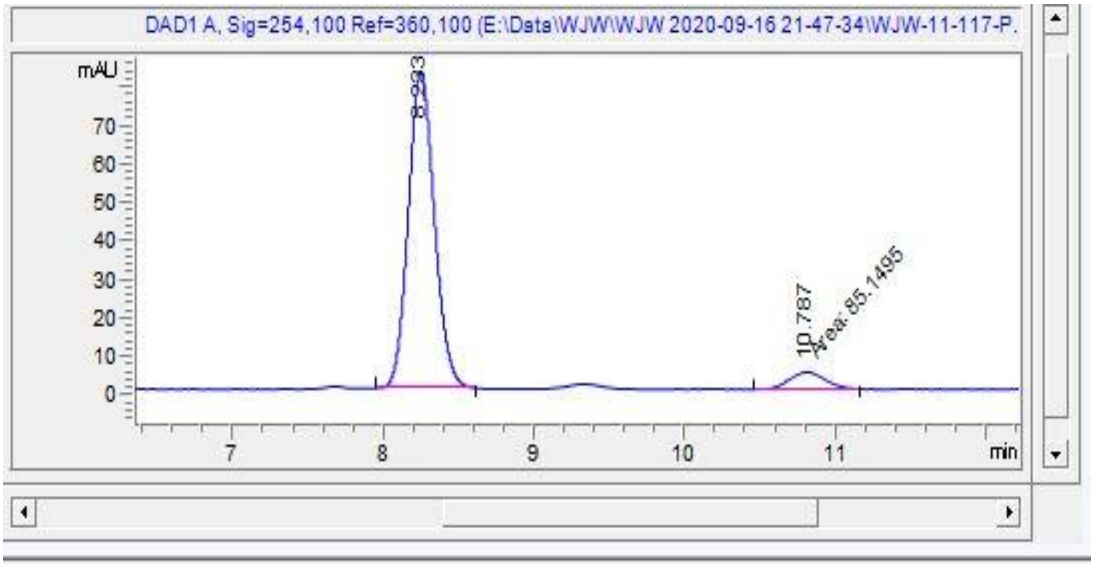

#	Time	Type	Area	Height	Width	Area%	Symmetry
1	8.233	VV R	983.4	81.6	0.1611	92.031	0.85
2	10.787	MM	85.1	4.8	0.2987	7.969	0.876

Supplementary Fig. 74 HPLC spectrum of chiral **3z.**

N-((R,1E,5E)-4-acetyl-4,6-diphenylhexa-1,5-dien-1-yl)-N-phenylpivalamide (**5a**)

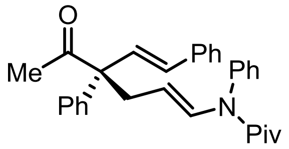

with (*S*)-**A9** catalyst (10 mol%)

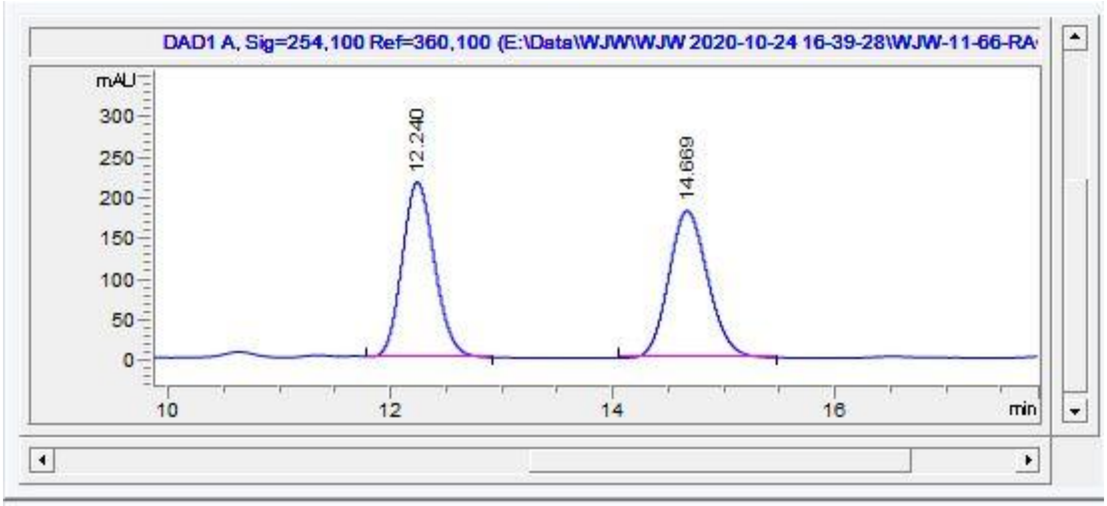

#	Time	Type	Area	Height	Width	Area%	Symmetry
1	12.24	BV R	4336.1	214.5	0.2398	49.482	0.87
2	14.669	VV R	4426.9	180.3	0.2899	50.518	0.832

Supplementary Fig. 75 HPLC spectrum of racemic **5a.**

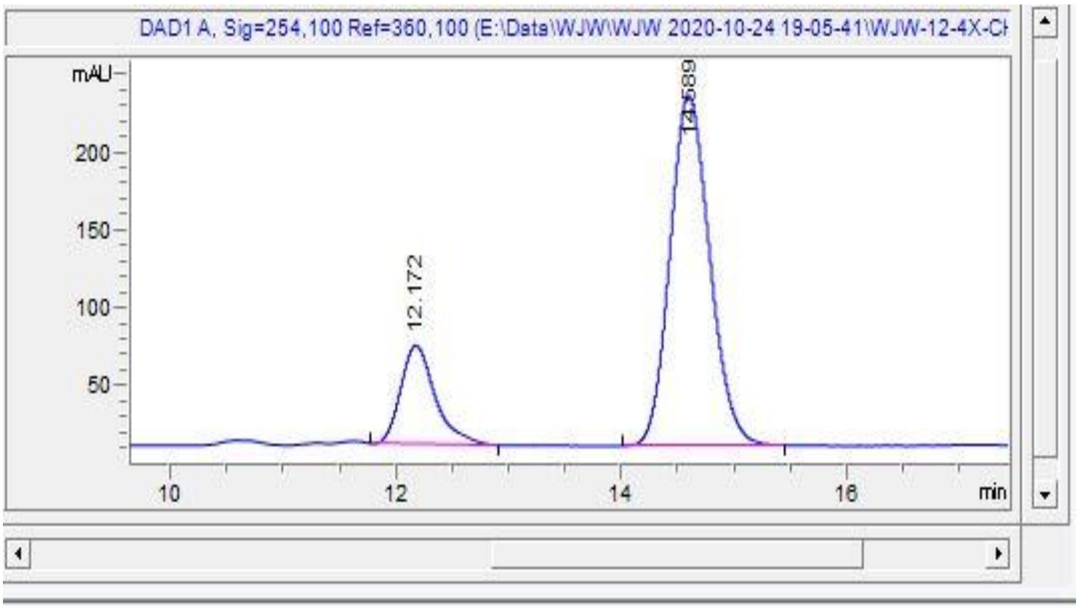

#	Time	Type	Area	Height	Width	Area%	Symmetry
1	12.172	BV R	1353.5	63.3	0.2521	19.695	0.784
2	14.589	VV R	5519	223.8	0.2917	80.305	0.834

Supplementary Fig. 76 HPLC spectrum of chiral **5a.**

N-((S,1E,5E)-4-acetyl-4,6-diphenylhexa-1,5-dien-1-yl)-N-phenylpivalamide (**5a**)

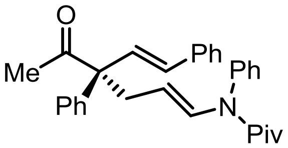

with (*R*)-**A6** catalyst (10 mol%)

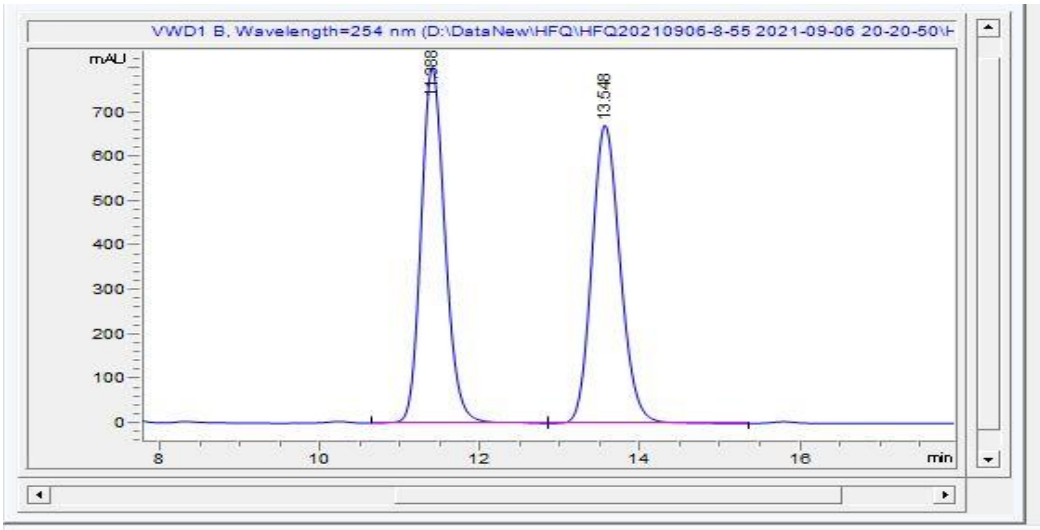

#	Time	Type	Area	Height	Width	Area%	Symmetry
1	11.388	VV R	16580.3	797.5	0.3201	50.267	0.821
2	13.548	BB	16403.8	667.5	0.3807	49.733	0.827

Supplementary Fig. 77 HPLC spectrum of racemic **5a.**

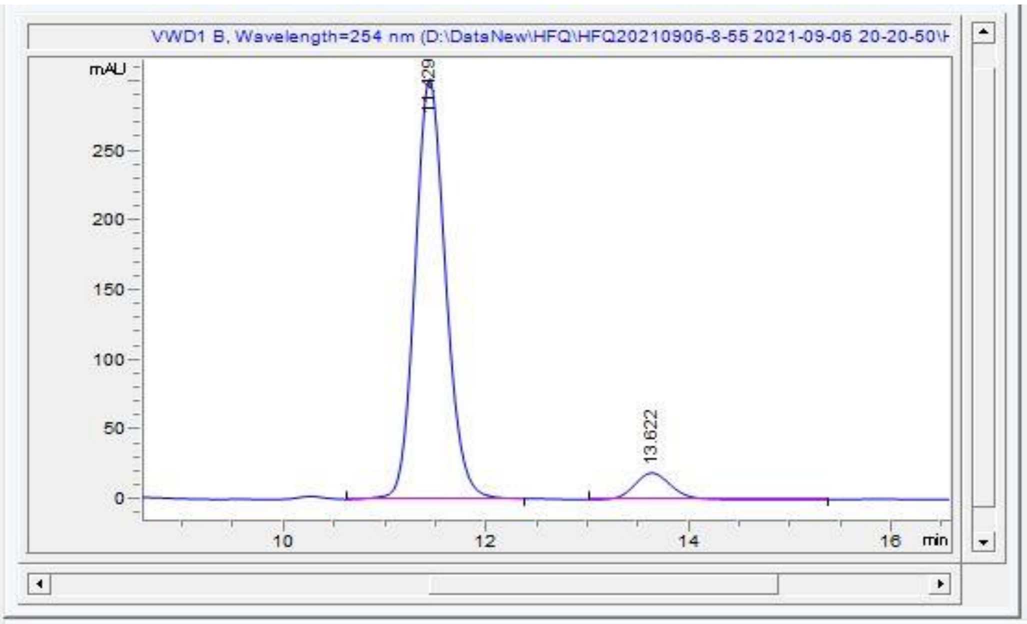

#	Time	Type	Area	Height	Width	Area%	Symmetry
1	11.429	BB	6281.8	300	0.3243	92.997	0.856
2	13.622	BB	473.1	19	0.3851	7.003	0.854

Supplementary Fig. 78 HPLC spectrum of chiral **5a.**

ethyl (*E*)-2-acetyl-2-phenyl-5-(N-phenylpivalamido)pent-4-enoate (**5d**)

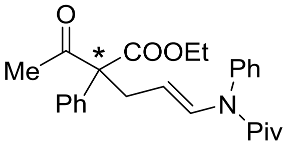

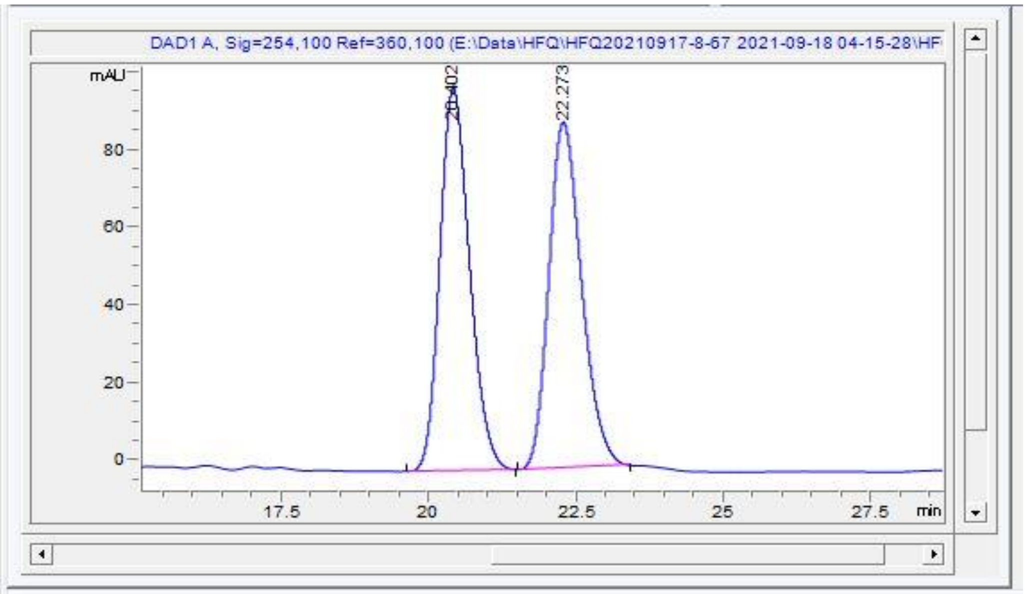

#	Time	Type	Area	Height	Width	Area%	Symmetry
1	20.402	BB	3467.3	99.1	0.4153	49.989	0.78
2	22.273	BB	3468.8	89.7	0.4534	50.011	0.788

Supplementary Fig. 79 HPLC spectrum of racemic **5d.**

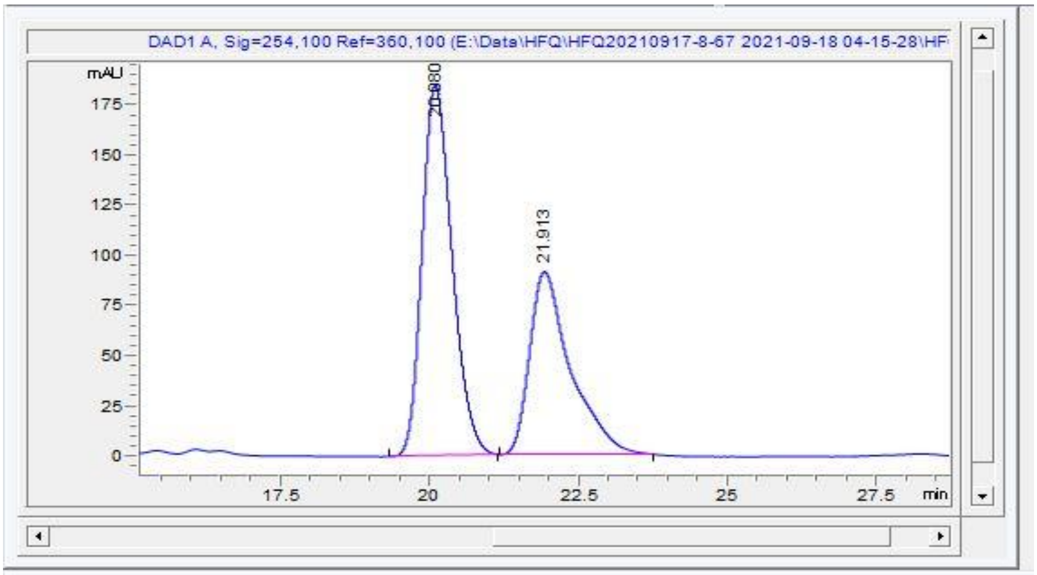

#	Time	Type	Area	Height	Width	Area%	Symmetry
1	20.08	BB	6493.4	185.6	0.4111	59.648	0.751
2	21.913	BB	4392.8	91.6	0.5641	40.352	0.553

Supplementary Fig. 80 HPLC spectrum of chiral **5d.**

(*S*)-N-(4-acetyl-4-phenyl-6-(triisopropylsilyl)hex-5-yn-1-yl)-N-phenylpivalamide (**7a**)

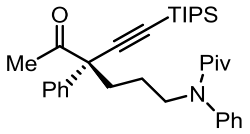

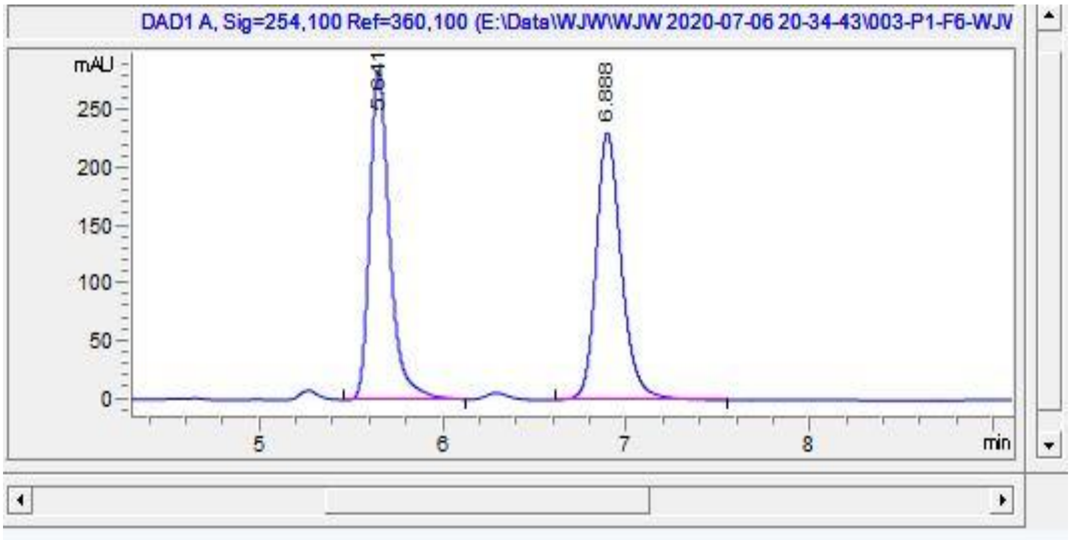

#	Time	Type	Area	Height	Width	Area%	Symmetry
1	5.641	BB	2188.5	285.9	0.1148	49.619	0.696
2	6.888	BB	2222.1	232.1	0.1442	50.381	0.75

Supplementary Fig. 81 HPLC spectrum of racemic **7a.**

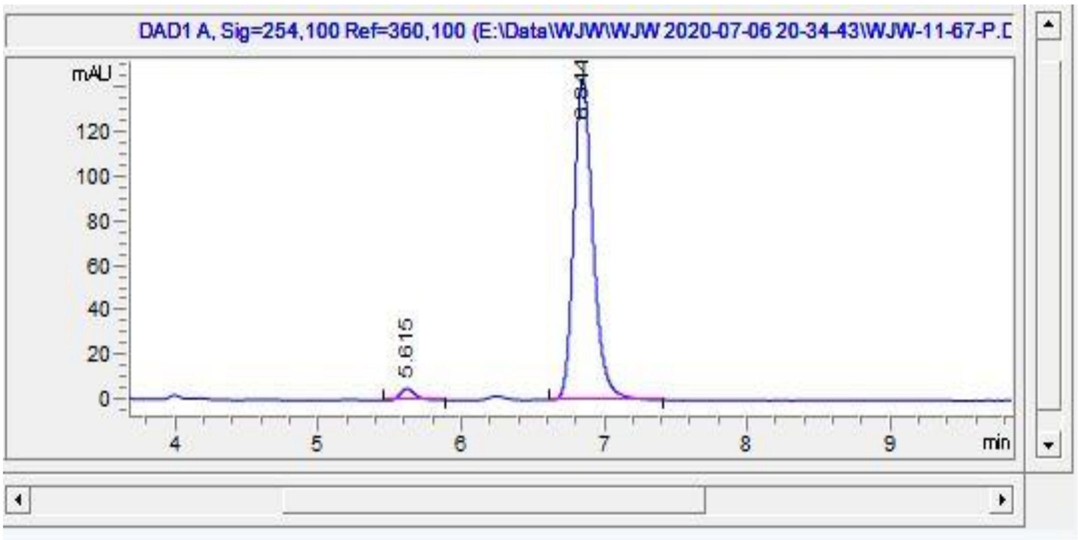

#	Time	Type	Area	Height	Width	Area%	Symmetry
1	5.615	BB	38.9	5.3	0.0973	2.810	0.777
2	6.844	BB	1346.6	144.1	0.14	97.190	0.767

Supplementary Fig. 82 HPLC spectrum of chiral **7a.**

(*R*)-N-(4-acetyl-4-phenyl-6-(triisopropylsilyl)hexyl)-N-phenylpivalamide (**8a**)

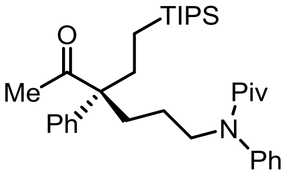

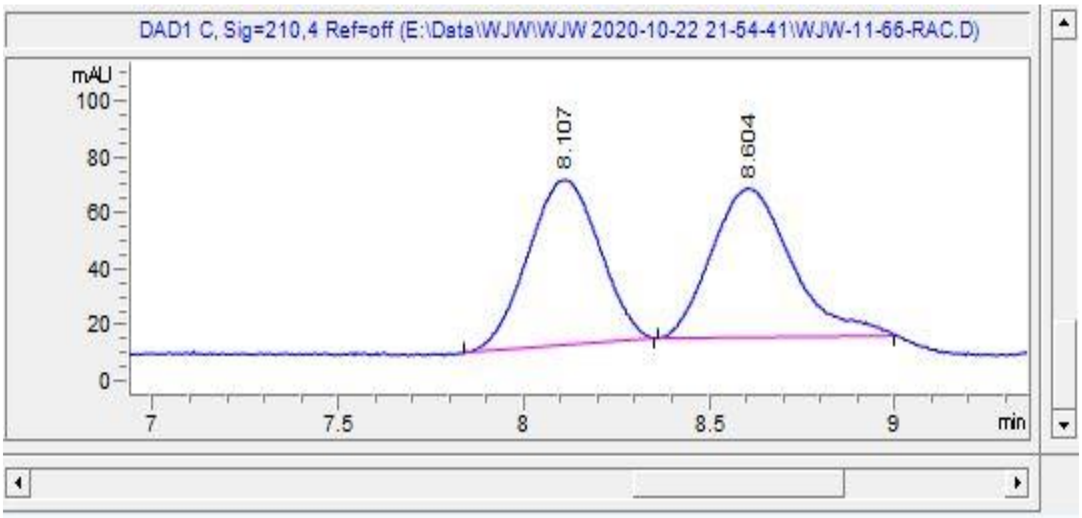

#	Time	Type	Area	Height	Width	Area%	Symmetry
1	8.107	VB R	794.8	59.1	0.1599	49.809	1.017
2	8.604	BV R	800.9	53.1	0.1757	50.191	0.824

Supplementary Fig. 83 HPLC spectrum of racemic **8a.**

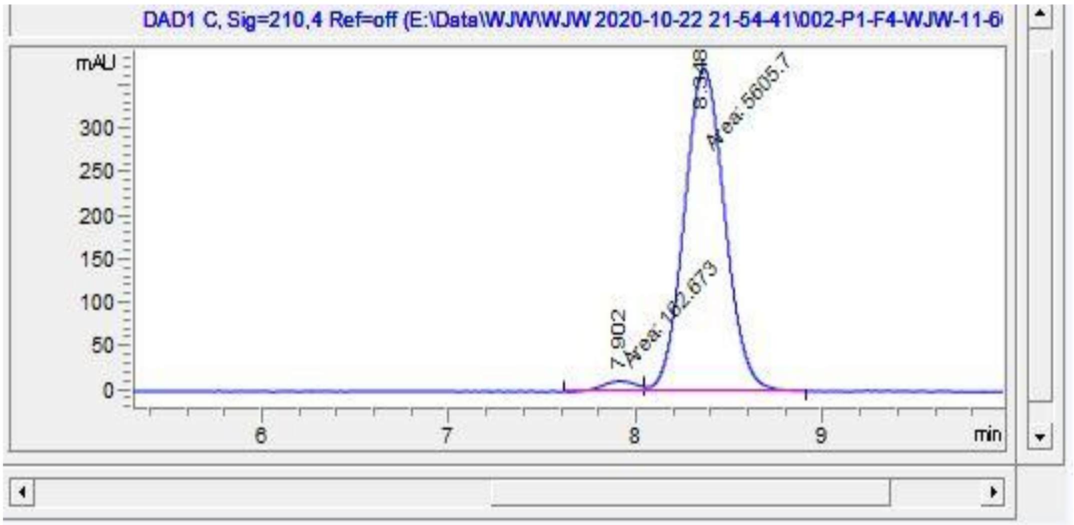

#	Time	Type	Area	Height	Width	Area%	Symmetry
1	7.902	MF	162.7	12.4	0.2185	2.820	1.143
2	8.348	FM	5605.7	372.4	0.2509	97.180	0.88

Supplementary Fig. 84 HPLC spectrum of chiral **8a.**

(*S*,*E*)-N-(4-(1-benzyl-1H-1,2,3-triazol-4-yl)-5-oxo-4-phenylhex-1-en-1-yl)-N-phenylpivalamide (**9m**)

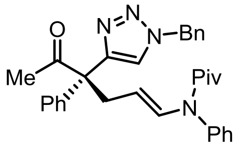

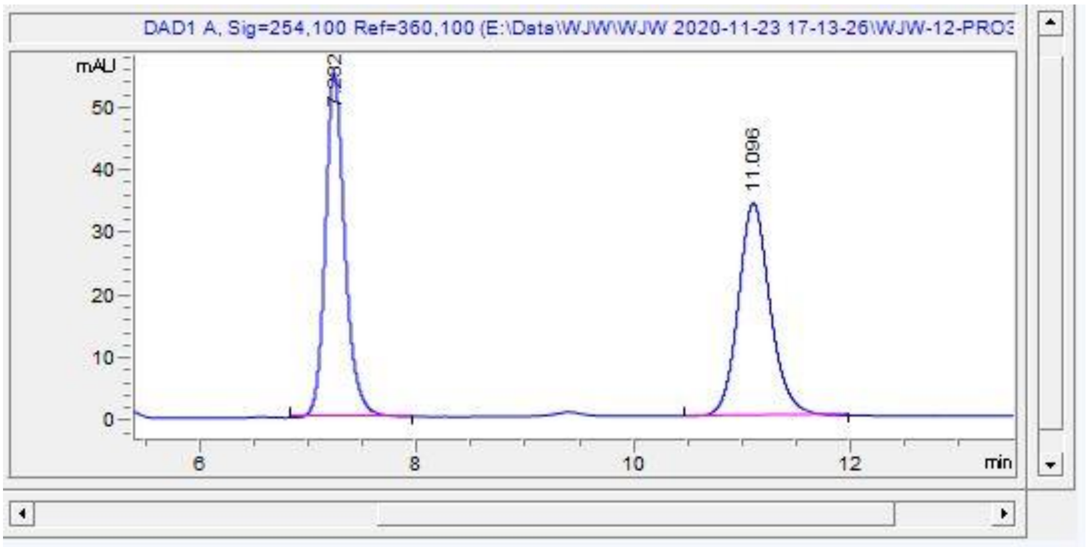

#	Time	Type	Area	Height	Width	Area%	Symmetry
1	7.232	BB	693.5	55	0.1901	49.975	0.796
2	11.096	BB	694.2	34	0.3033	50.025	0.847

Supplementary Fig. 85 HPLC spectrum of racemic **9m.**

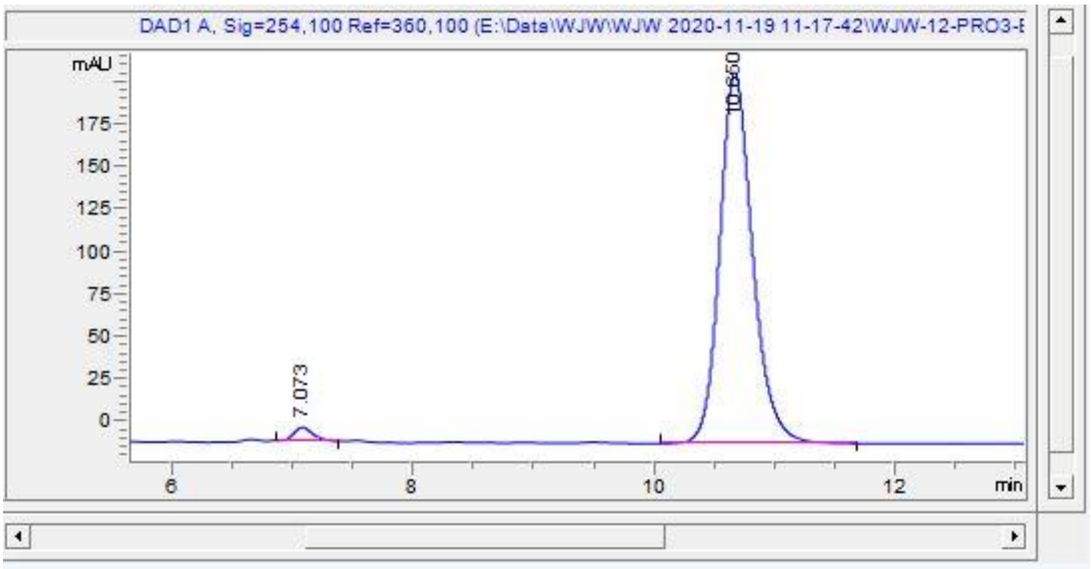

#	Time	Type	Area	Height	Width	Area%	Symmetry
1	7.073	BB	84.9	7.8	0.1531	2.014	0.792
2	10.65	BB	4133.4	218.6	0.2851	97.986	0.799

Supplementary Fig. 86 HPLC spectrum of chiral **9m.**

(*S*)-4-acetyl-4-phenyl-6-(triisopropylsilyl)hex-5-ynal (**10a**)

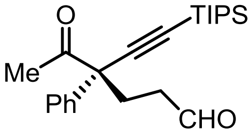

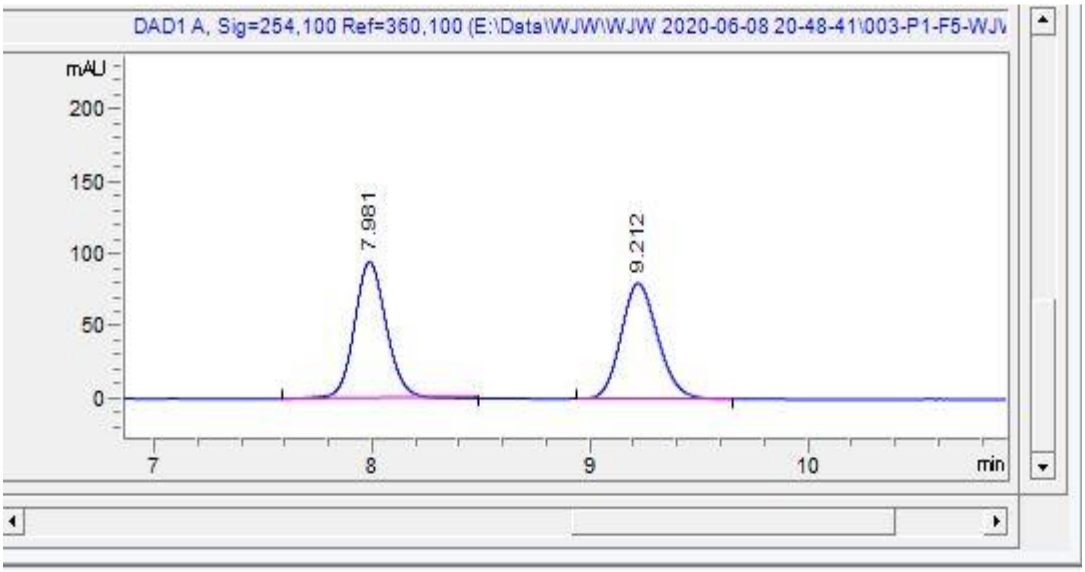

#	Time	Type	Area	Height	Width	Area%	Symmetry
1	7.981	BB	974.9	94.6	0.1568	50.946	0.867
2	9.212	BB	938.7	79.8	0.1742	49.054	0.849

Supplementary Fig. 87 HPLC spectrum of racemic **10a.**

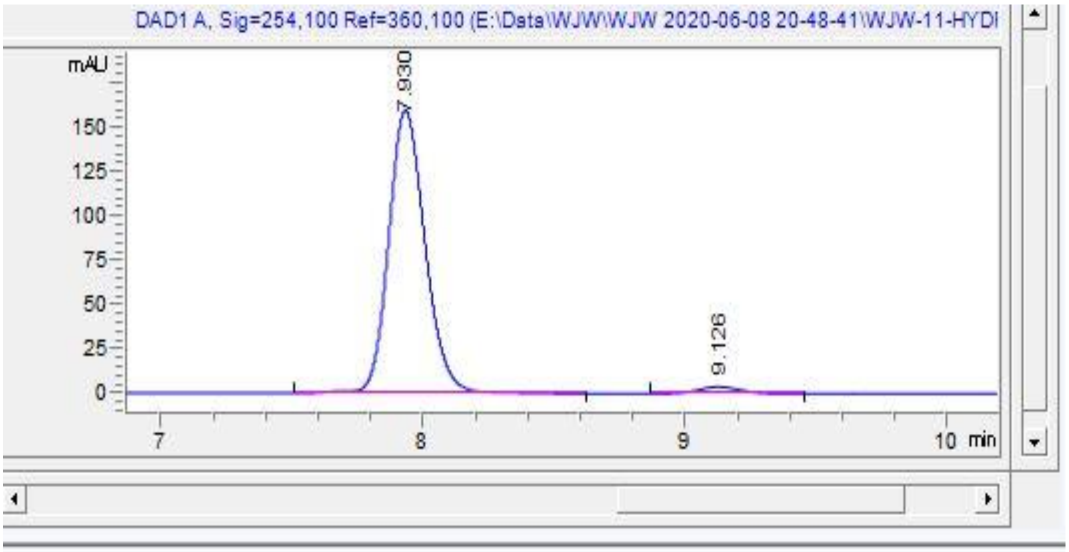

#	Time	Type	Area	Height	Width	Area%	Symmetry
1	7.93	BB	1537.4	158.4	0.1497	97.285	0.82
2	9.126	BB	42.9	3.8	0.1367	2.715	0.931

Supplementary Fig. 88 HPLC spectrum of chiral **10a.**

(*S*)-1-((triisopropylsilyl)ethynyl)-5,6-dihydro-[1,1'-biphenyl]-2(1H)-one (**11a**)

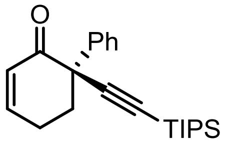

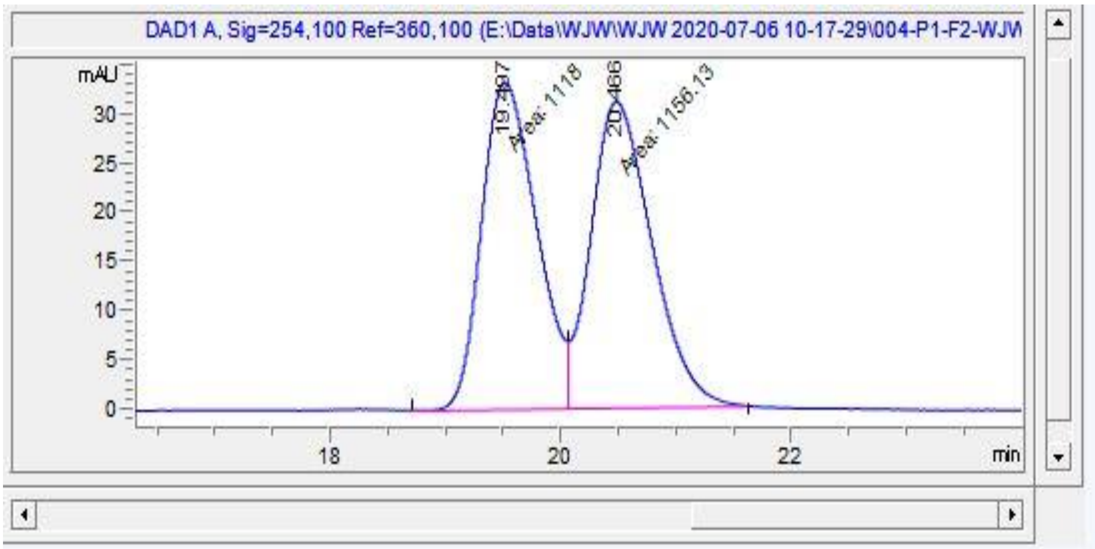

#	Time	Type	Area	Height	Width	Area%	Symmetry
1	19.497	MF	1118	33.5	0.5564	49.162	0
2	20.466	FM	1156.1	31.4	0.614	50.838	0.694

Supplementary Fig. 89 HPLC spectrum of racemic **11a.**

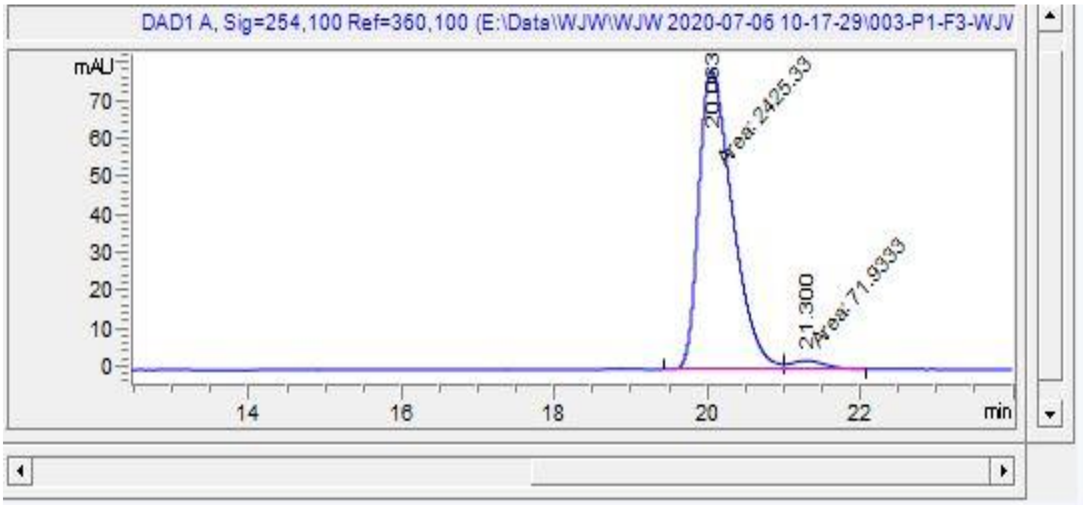

#	Time	Type	Area	Height	Width	Area%	Symmetry
1	20.063	MF	2425.3	78.9	0.5126	97.120	0.582
2	21.3	FM	71.9	2.3	0.5209	2.880	0.785

Supplementary Fig. 90 HPLC spectrum of chiral **11a.**

N-((4*S*,*E*)-4-(1-hydroxyethyl)-4-phenyl-6-(triisopropylsilyl)hex-1-en-5-yn-1-yl)-N-phenylpivalamide (**12a**)

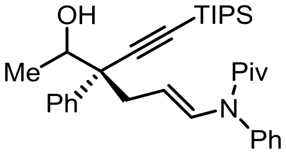

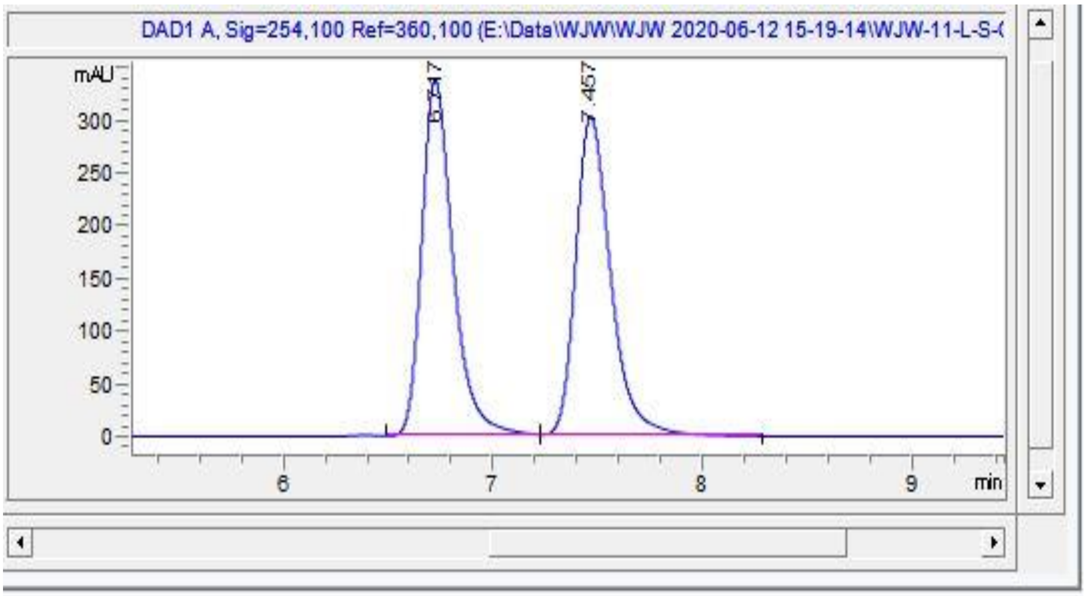

#	Time	Type	Area	Height	Width	Area%	Symmetry
1	6.717	BV	3505	337.7	0.1571	49.842	0.696
2	7.457	VB	3527.2	303.2	0.1747	50.158	0.694

Supplementary Fig. 91 HPLC spectrum of racemic **12a.**

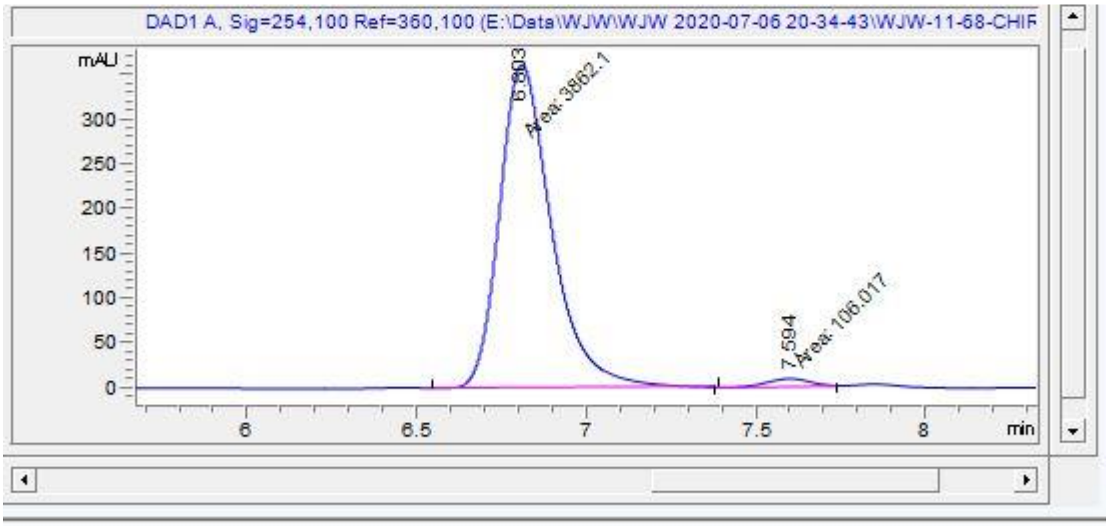

#	Time	Type	Area	Height	Width	Area%	Symmetry
1	6.803	MM	3862.1	360.3	0.1787	97.328	0.71
2	7.594	MM	106	10.3	0.1723	2.672	1.158

Supplementary Fig. 92 HPLC spectrum of chiral **12a.**

Triisopropyl(((2R,3S)-2-methyl-3-phenyl-3,4-dihydro-2H-pyran-3-yl)ethynyl)silane (**13a**)

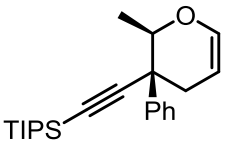

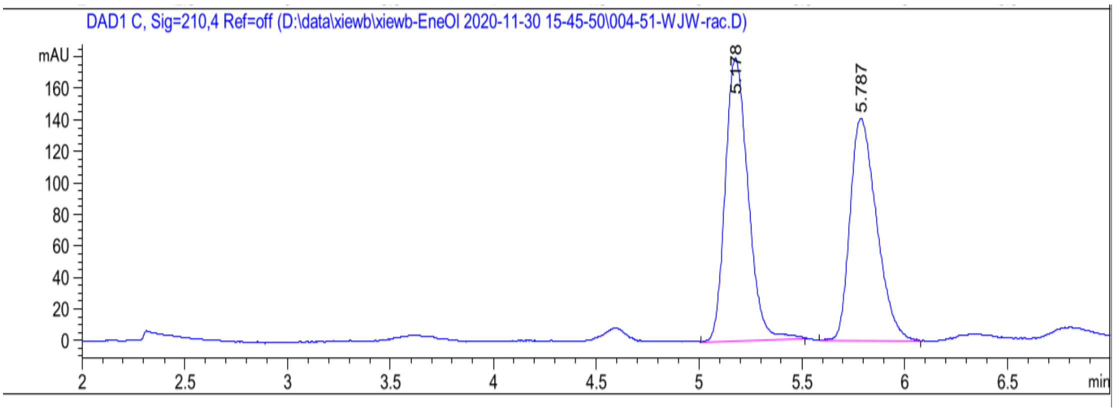

峰 #	保留时间 [min]	类型	峰宽 [min]	峰面积 [mAU*s]	峰高 [mAU]	峰面积 %
1	5.178	BV R	0.1194	1390.88672	179.46378	52.2499
2	5.787	VV R	0.1338	1271.10107	140.77255	47.7501

Supplementary Fig. 93 HPLC spectrum of racemic **13a.**

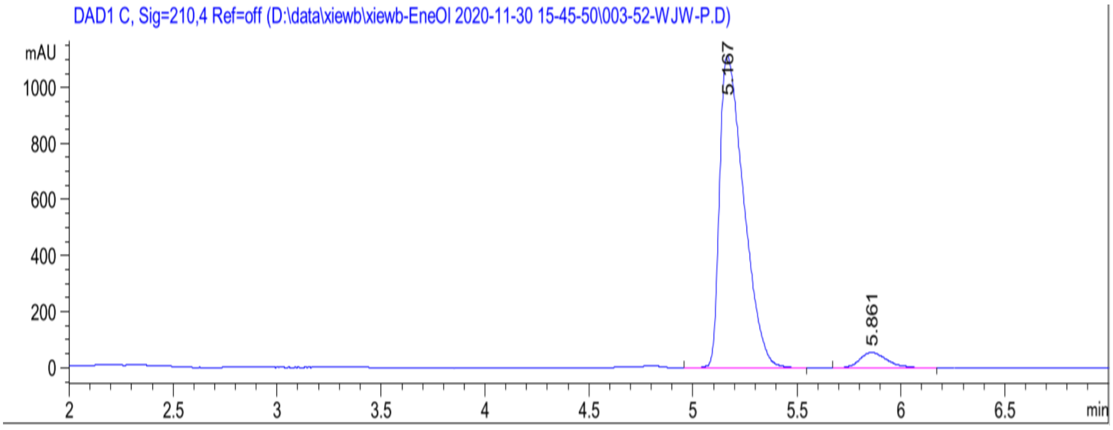

峰 #	保留时间 [min]	类型	峰宽 [min]	峰面积 [mAU*s]	峰高 [mAU]	峰面积 %
1	5.167	VV R	0.1263	8999.91406	1113.28345	94.8836
2	5.861	VV R	0.1278	485.30075	54.28787	5.1164

Supplementary Fig. 94 HPLC spectrum of chiral **13a.**

N-((2*R*,5*S*,6*R*)-6-methyl-5-phenyl-5-((triisopropylsilyl)ethynyl)tetrahydro-2H-pyran-2-yl)-N-phenylpivalamide (**13b**)

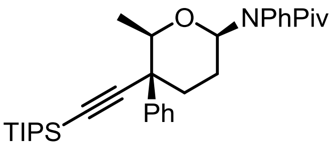

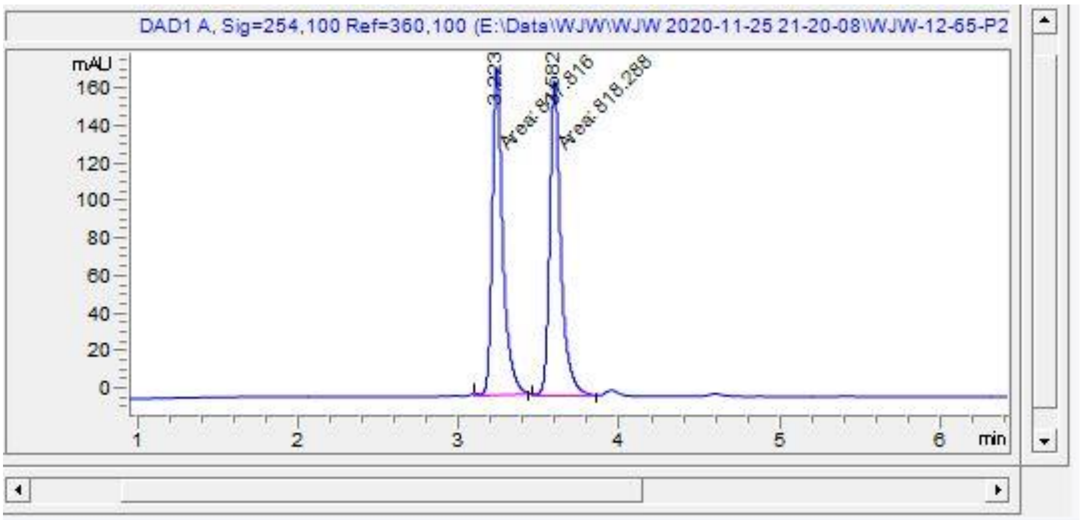

#	Time	Type	Area	Height	Width	Area%	Symmetry
1	3.223	MM	817.8	174.2	0.0783	49.986	0.706
2	3.582	MF	818.3	166.6	0.0818	50.014	0.702

Supplementary Fig. 95 HPLC spectrum of racemic **13b.**

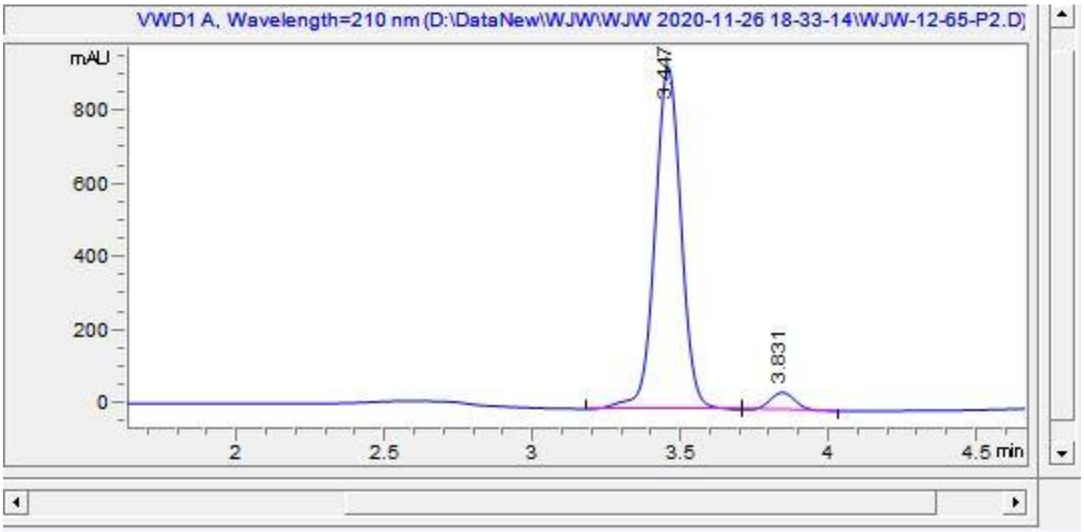

#	Time	Type	Area	Height	Width	Area%	Symmetry
1	3.447	BV	5767.3	942	0.0934	95.030	0.992
2	3.831	VB	301.6	49.5	0.095	4.970	0.956

Supplementary Fig. 96 HPLC spectrum of chiral **13b.**

10. NMR spectrums

3-phenyl-5-(triisopropylsilyl)pent-4-yn-2-one (**1a**)

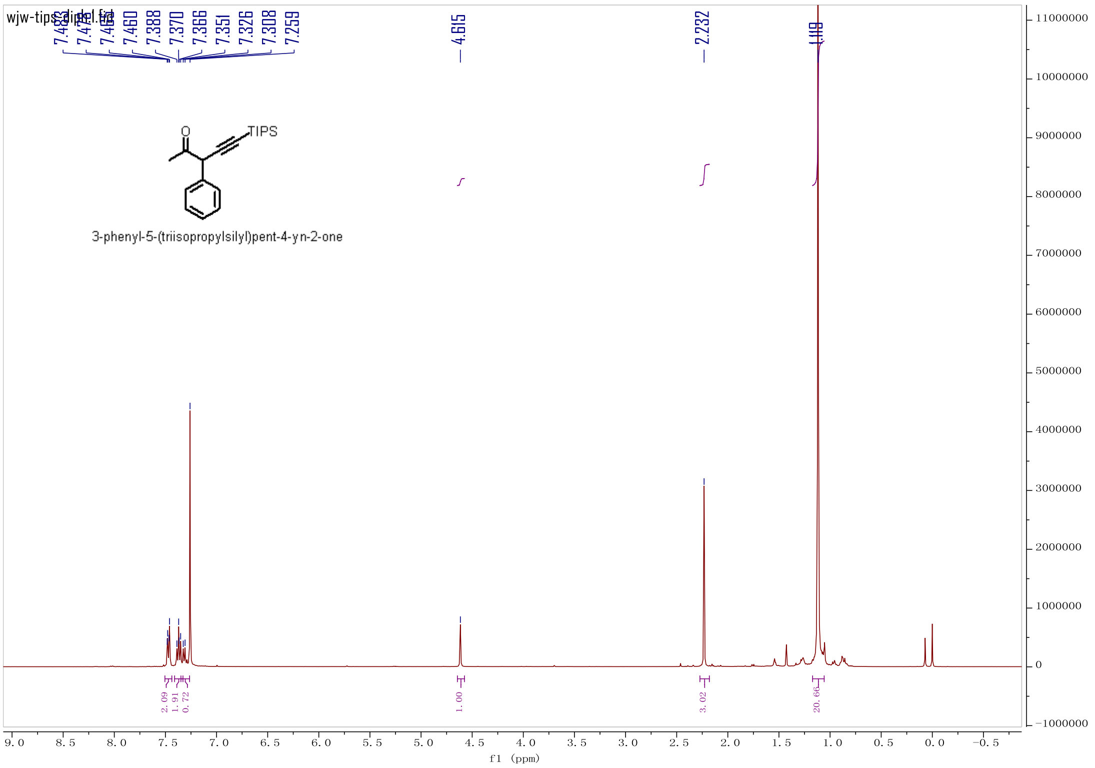

Supplementary Fig. 97 ^{1}H NMR spectrum of **1a**

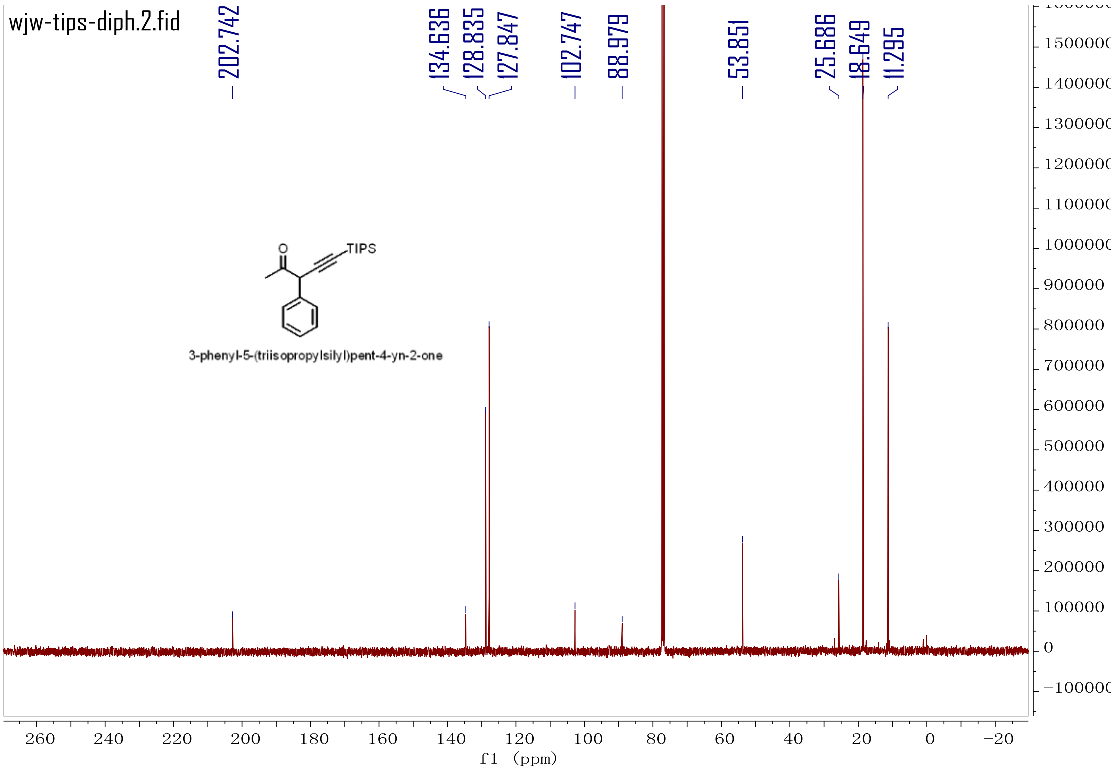

Supplementary Fig. 98 ^{13}C NMR spectrum of **1a**

3-(p-tolyl)-5-(triisopropylsilyl)pent-4-yn-2-one (**1b**)

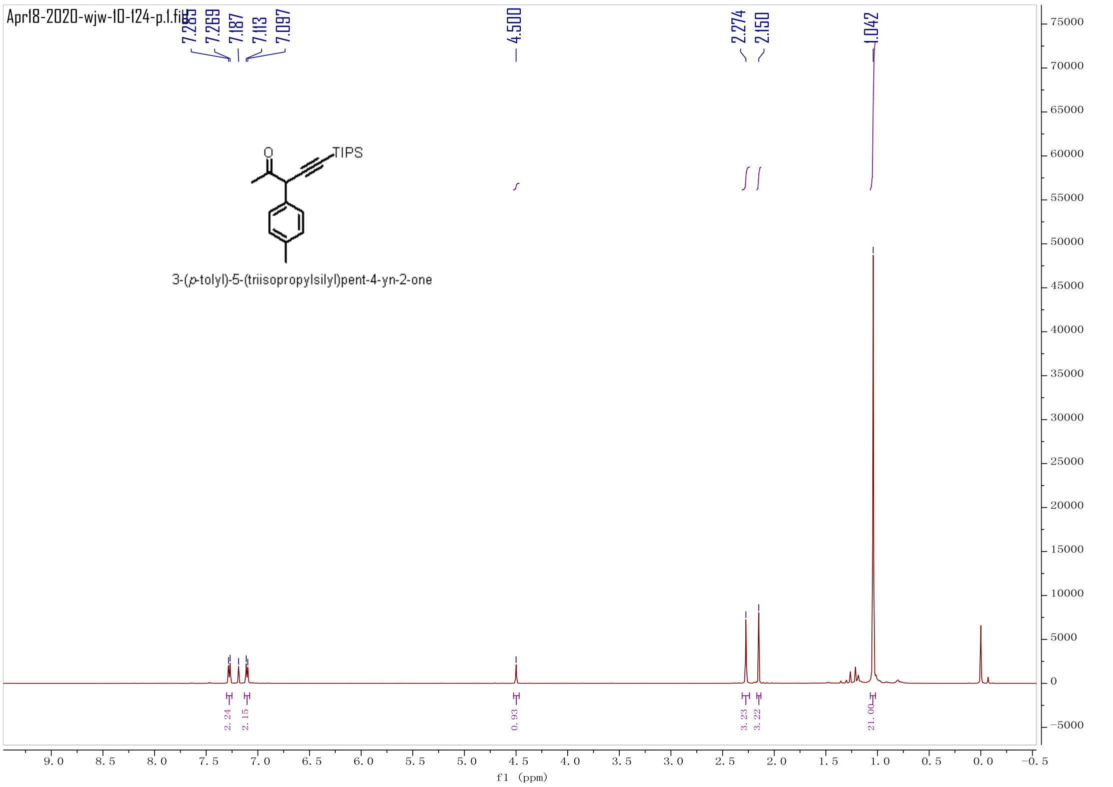

Supplementary Fig. 99 ^{1}H NMR spectrum of **1b**

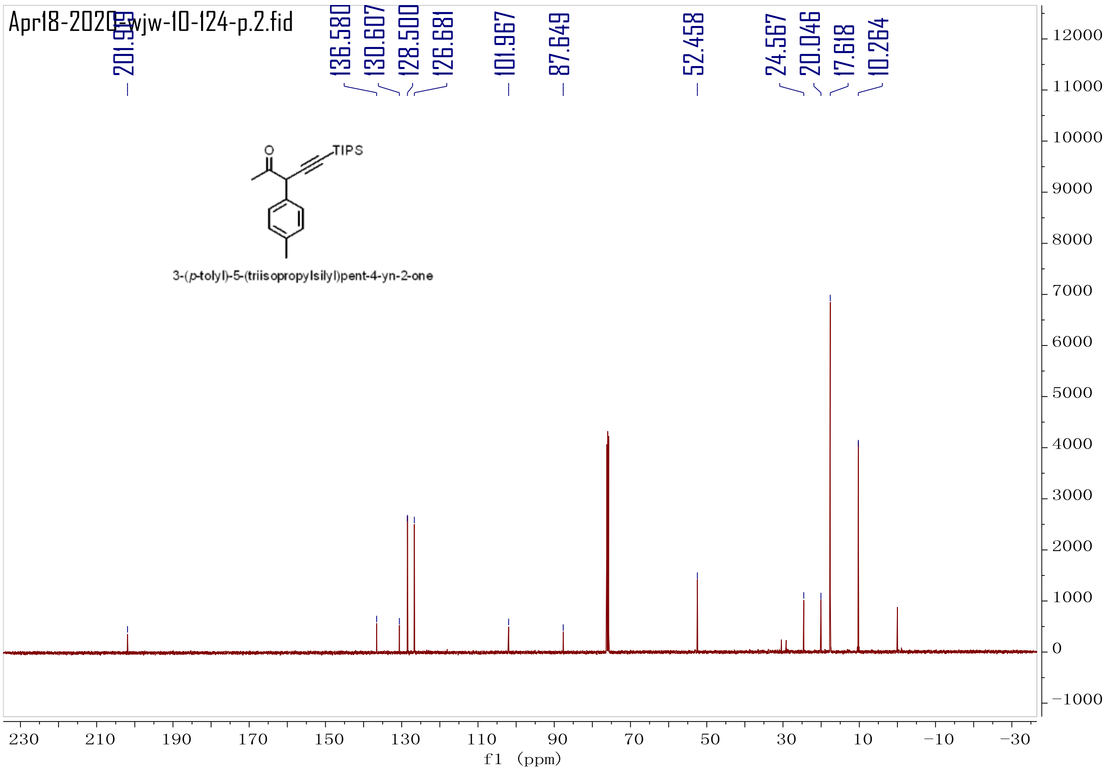

Supplementary Fig. 100 ^{13}C NMR spectrum of **1b**

3-(4-chlorophenyl)-5-(triisopropylsilyl)pent-4-yn-2-one (**1c**)

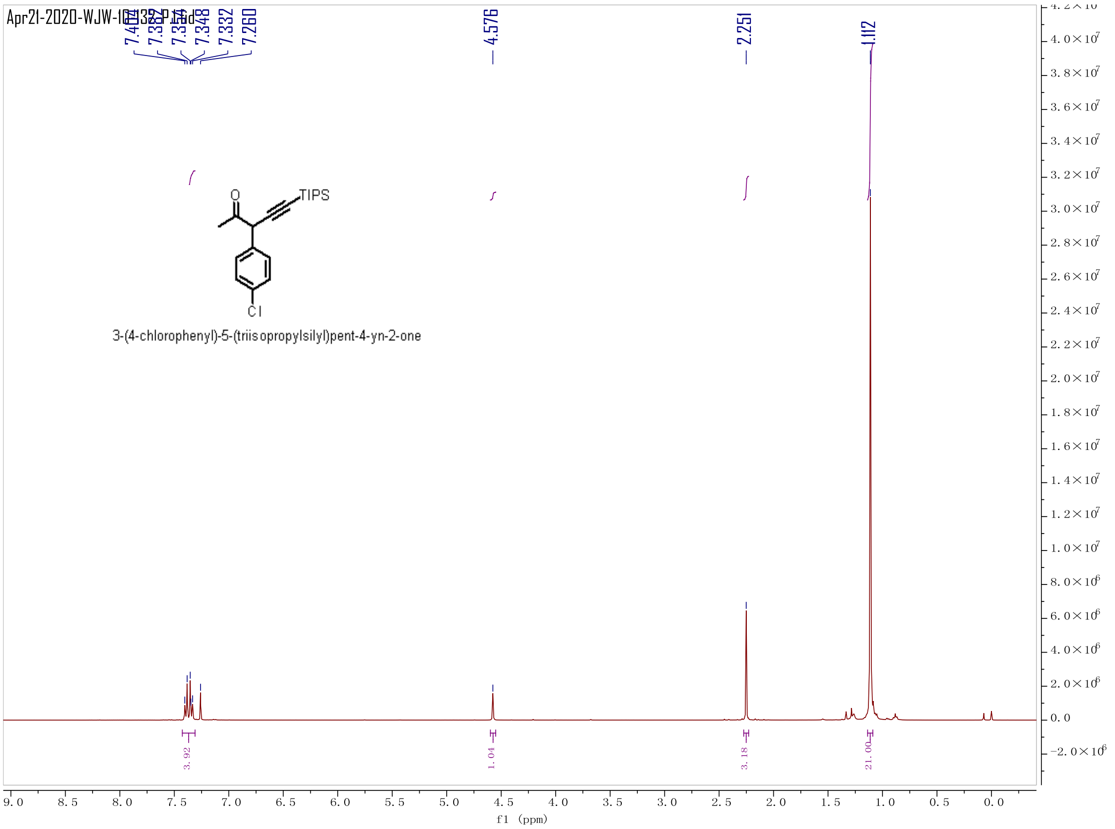

Supplementary Fig. 101 ^{1}H NMR spectrum of **1c**

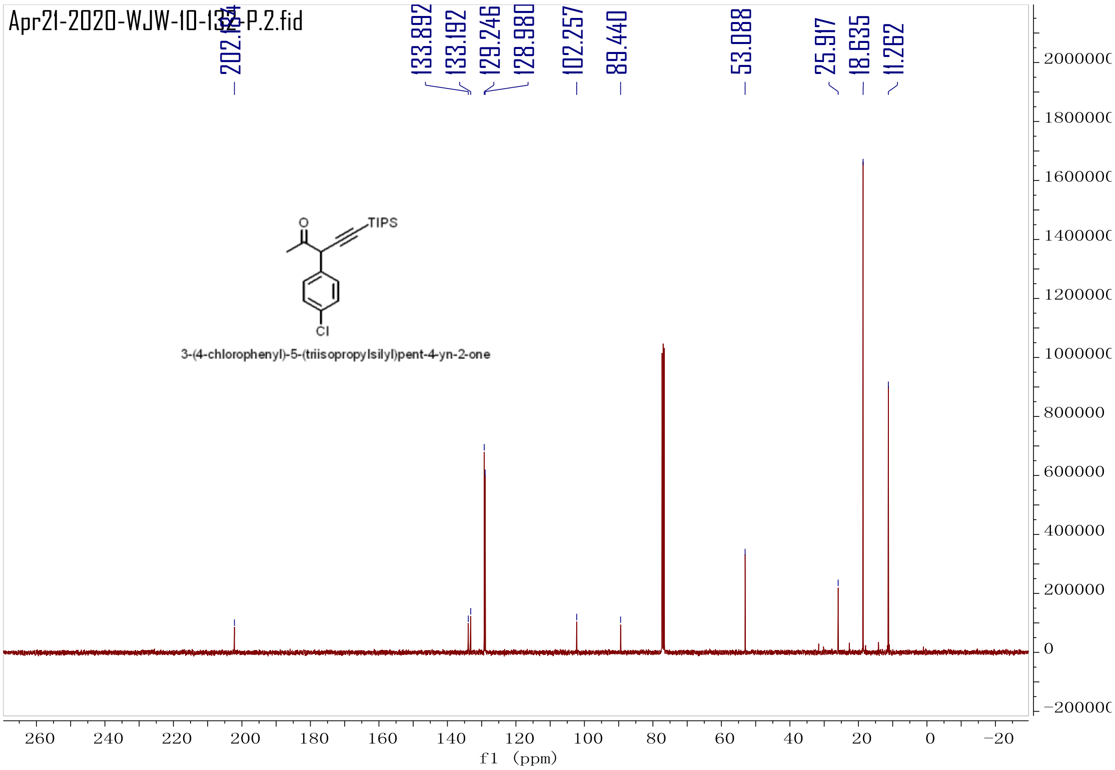

Supplementary Fig. 102 ^{13}C NMR spectrum of **1c**

3-(4-(trifluoromethyl)phenyl)-5-(triisopropylsilyl)pent-4-yn-2-one (**1d**)

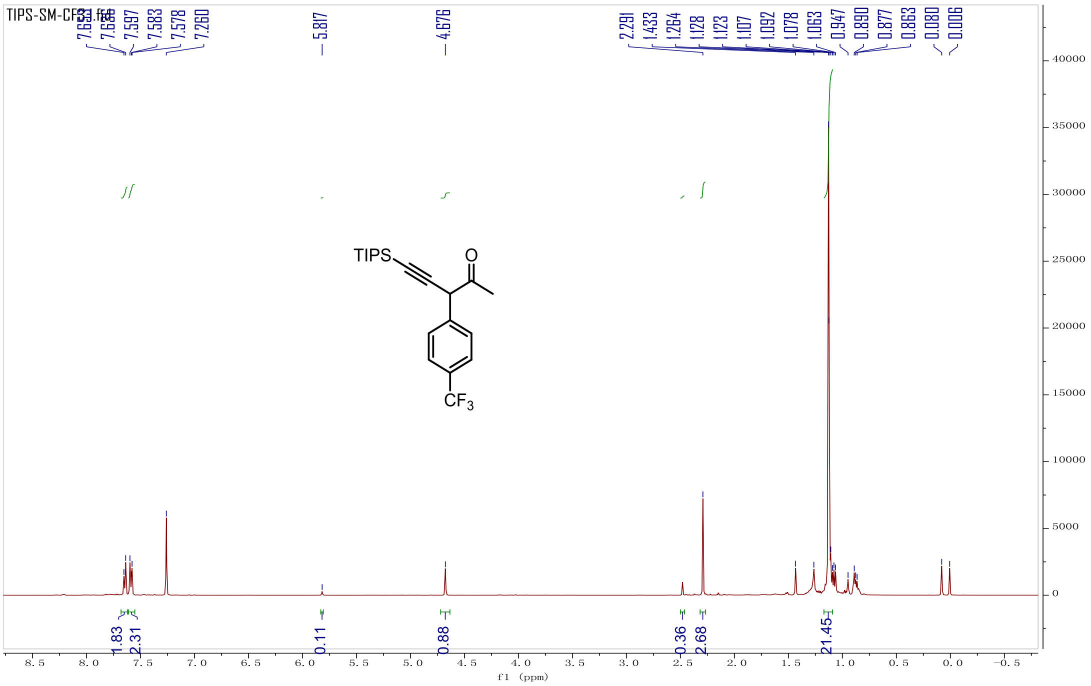

Supplementary Fig. 103 ^{1}H NMR spectrum of **1d**

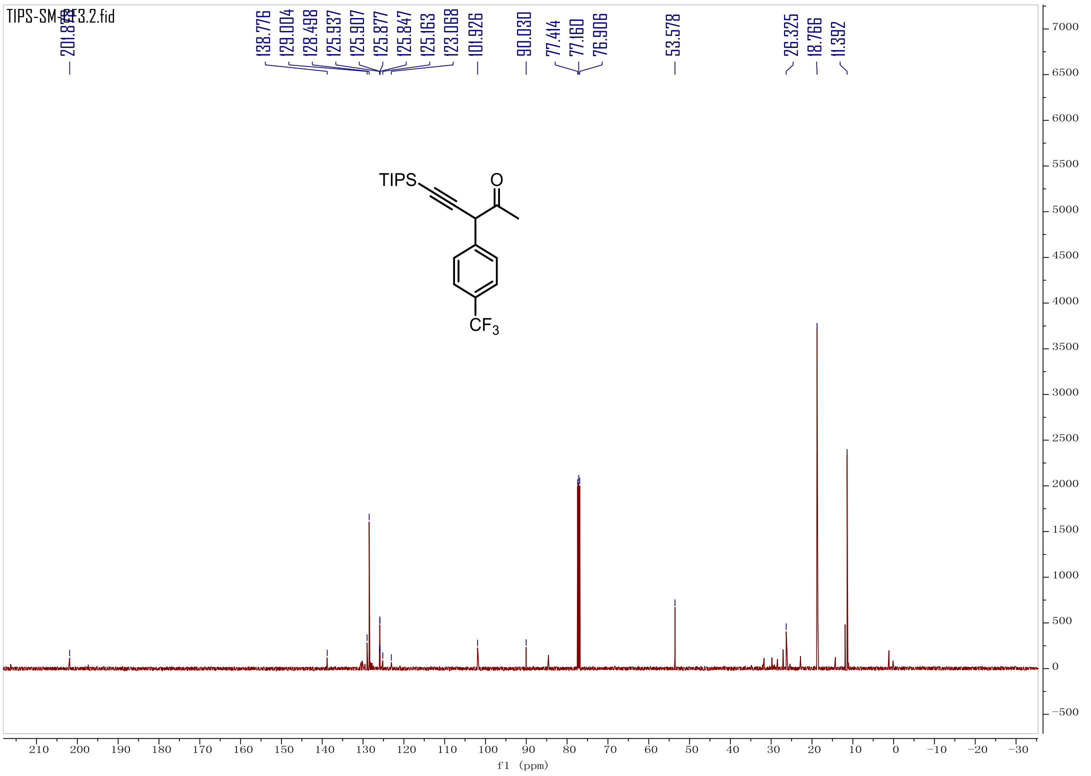

Supplementary Fig. 104 ^{13}C NMR spectrum of **1d**

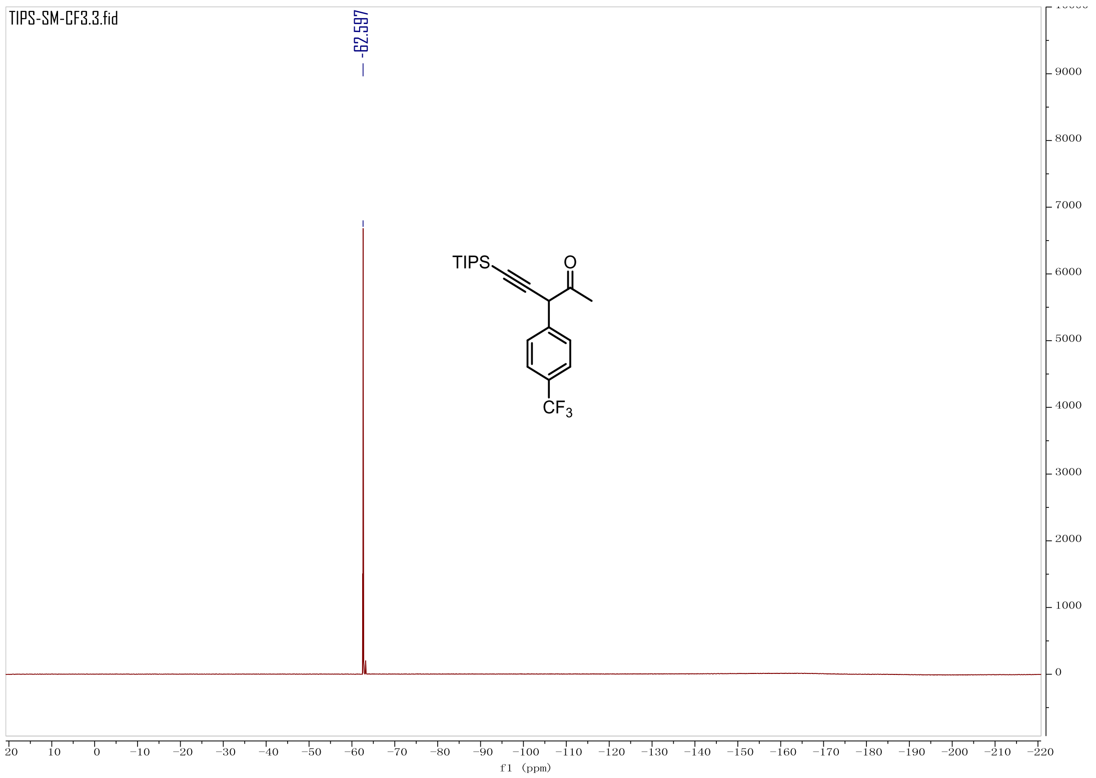

Supplementary Fig. 105 ^{19}F NMR spectrum of **1d**

3-(m-tolyl)-5-(triisopropylsilyl)pent-4-yn-2-one (**1e**)

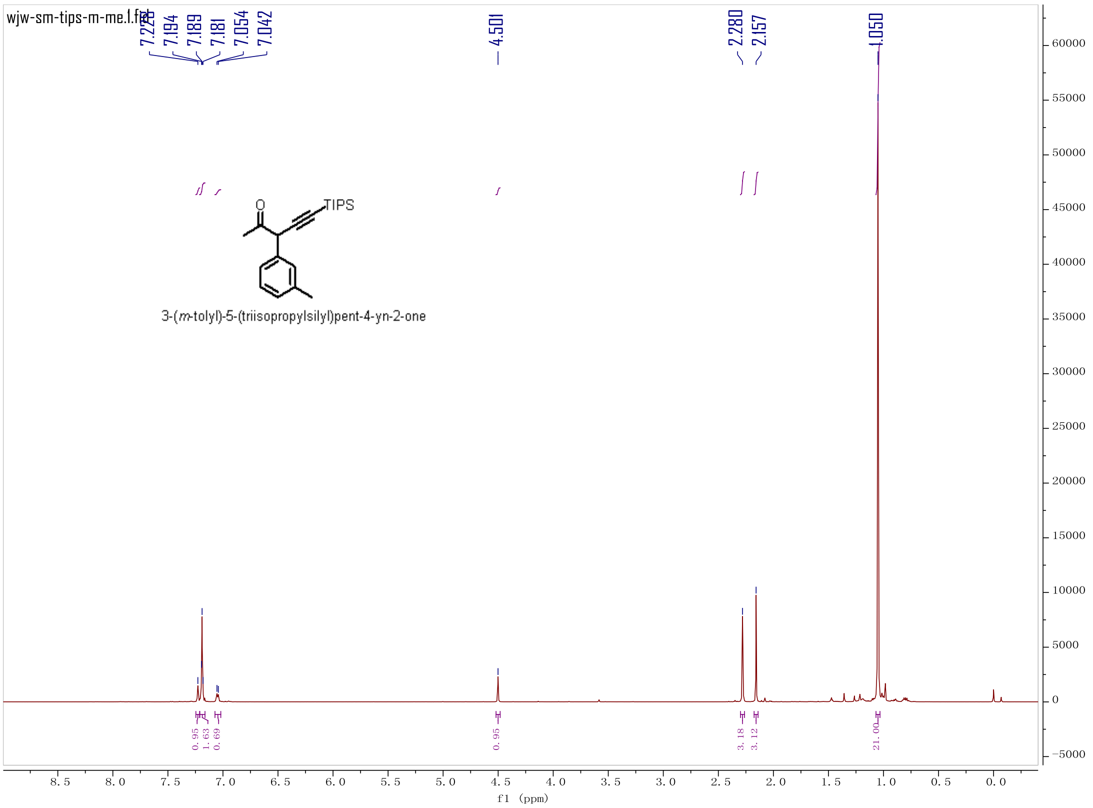

Supplementary Fig. 106 ^{1}H NMR spectrum of **1e**

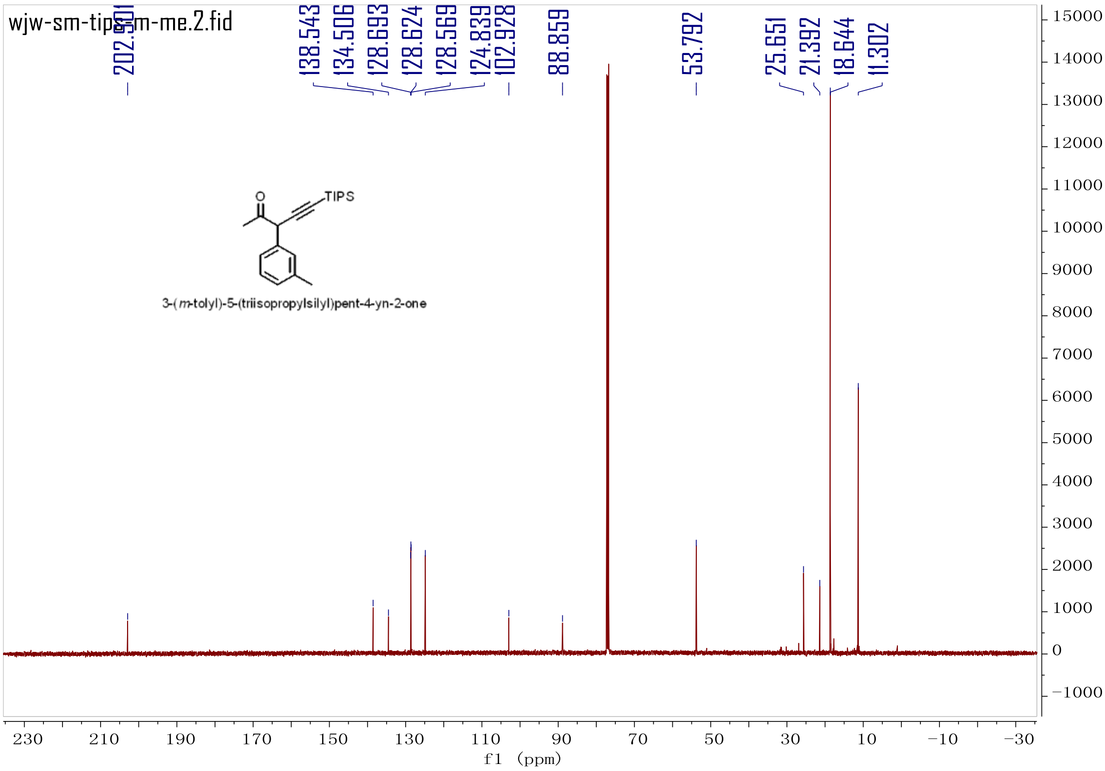

Supplementary Fig. 107 ^{13}C NMR spectrum of **1e**

3-(3-chlorophenyl)-5-(triisopropylsilyl)pent-4-yn-2-one (**1f**)

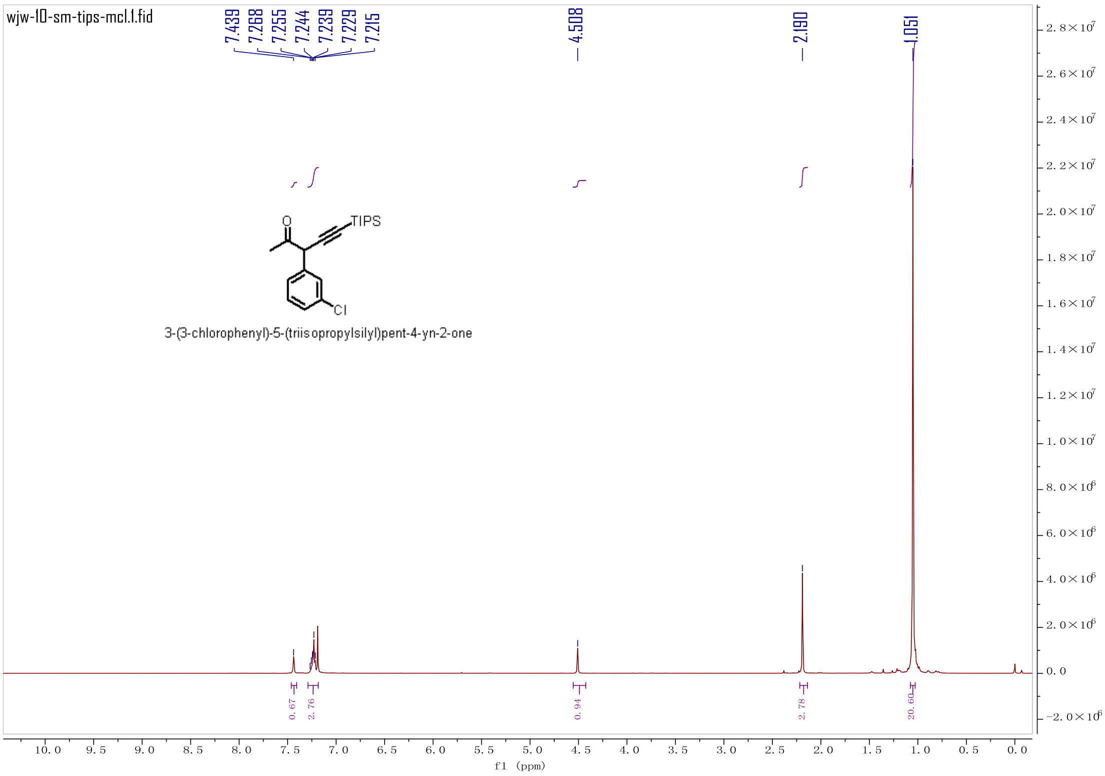

Supplementary Fig. 108 ^{1}H NMR spectrum of **1f**

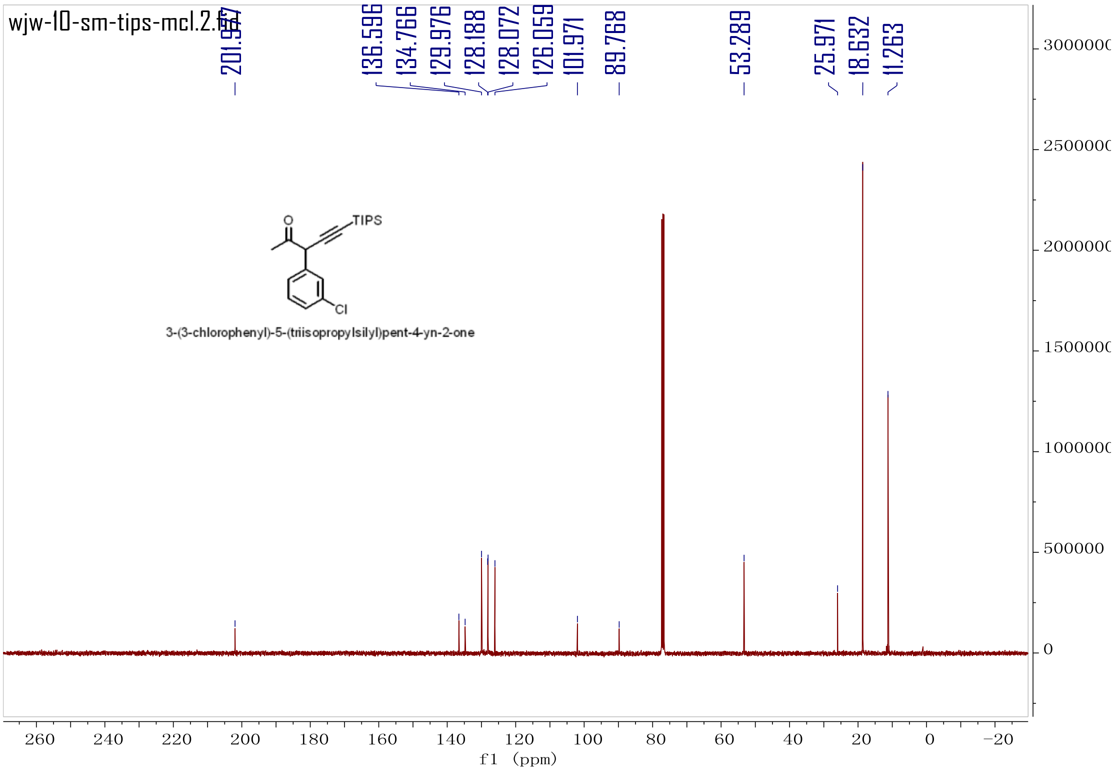

Supplementary Fig. 109 ^{13}C NMR spectrum of **1f**

3-(3-fluorophenyl)-5-(triisopropylsilyl)pent-4-yn-2-one (**1g**)

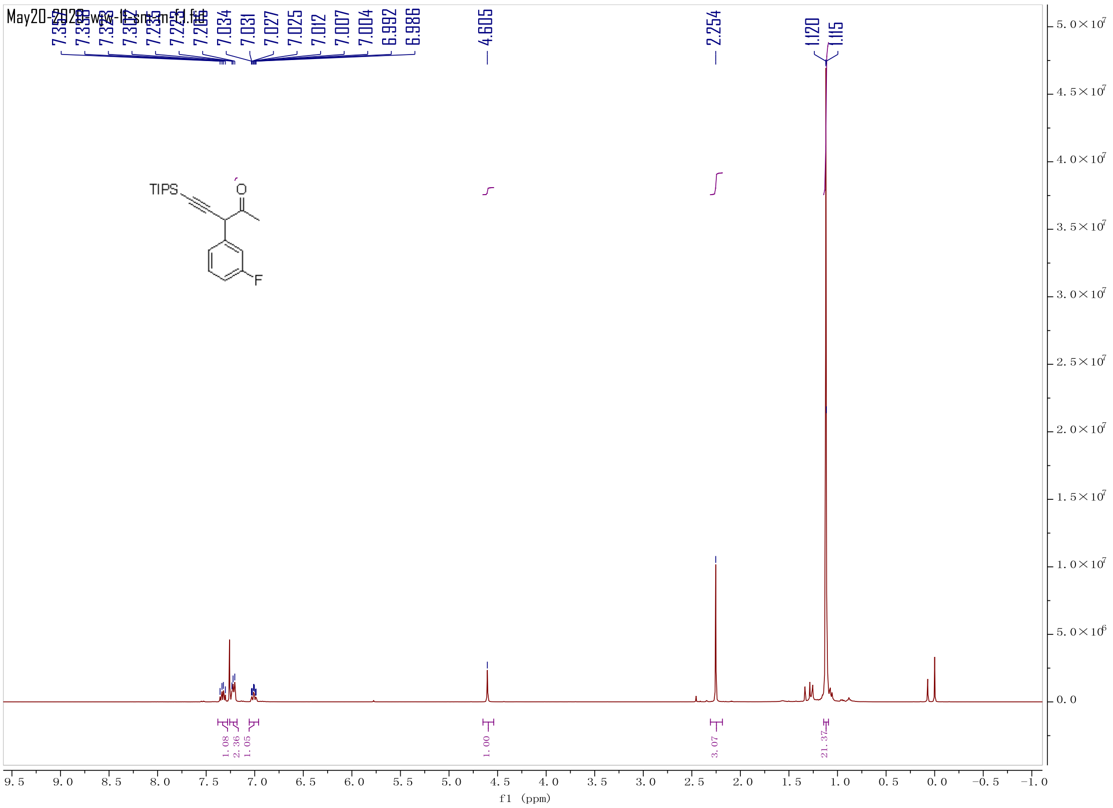

Supplementary Fig. 110 ^{1}H NMR spectrum of **1g**

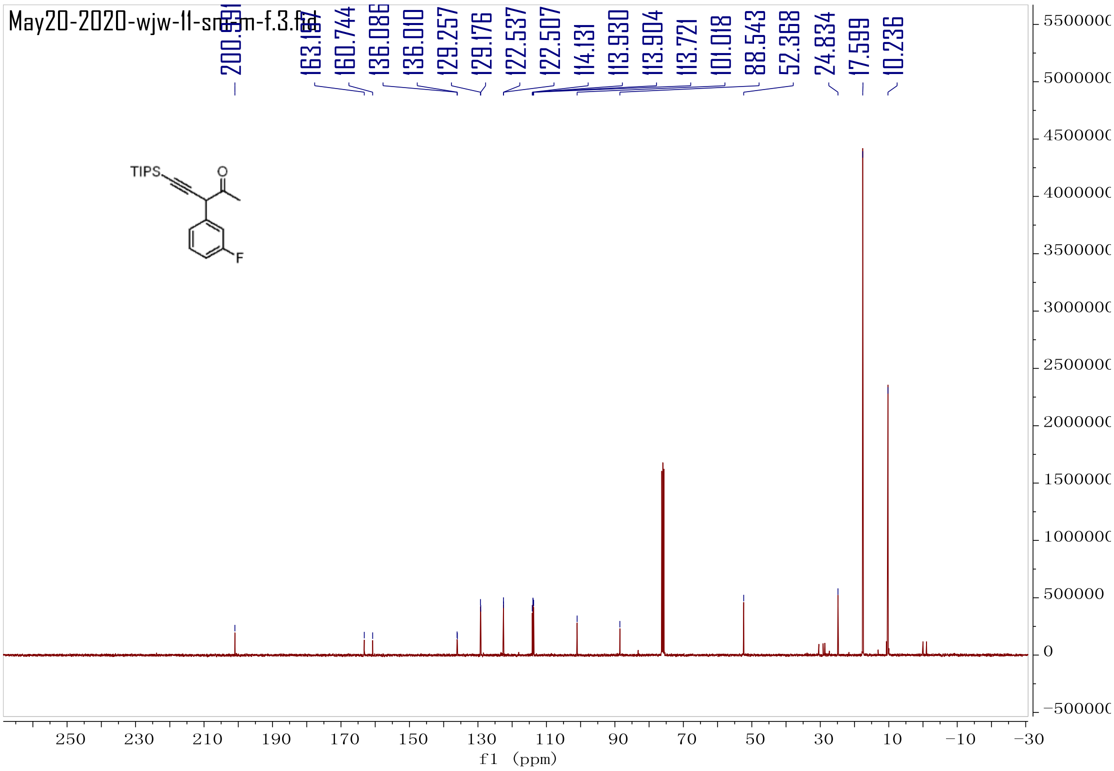

Supplementary Fig. 111 ^{13}C NMR spectrum of **1g**

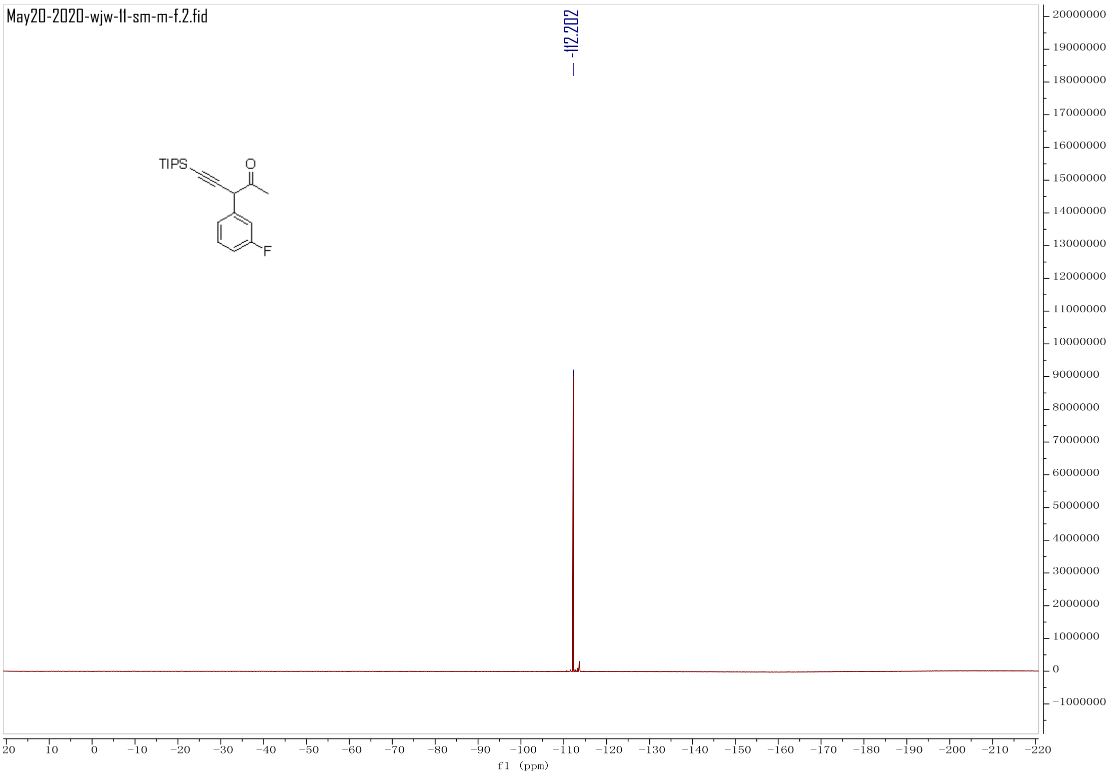

Supplementary Fig. 112 ^{19}F NMR spectrum of **1g**

3-(3-methoxyphenyl)-5-(triisopropylsilyl)pent-4-yn-2-one (**1h**)

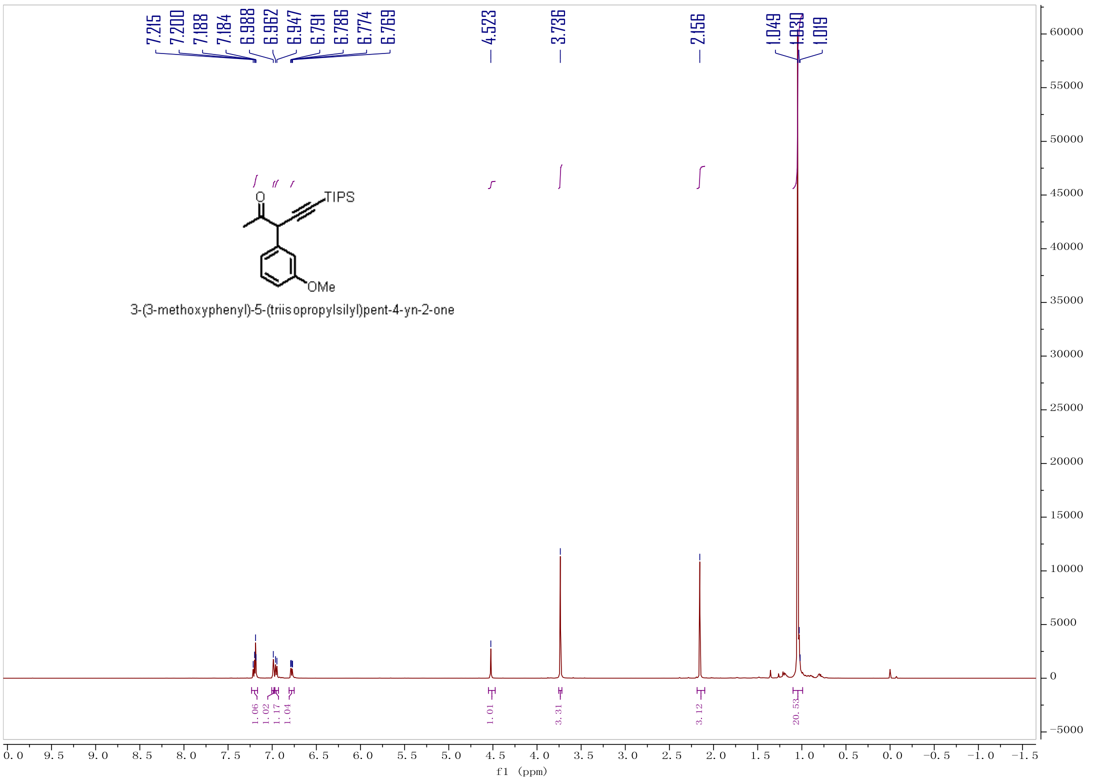

Supplementary Fig. 113 ^{1}H NMR spectrum of **1h**

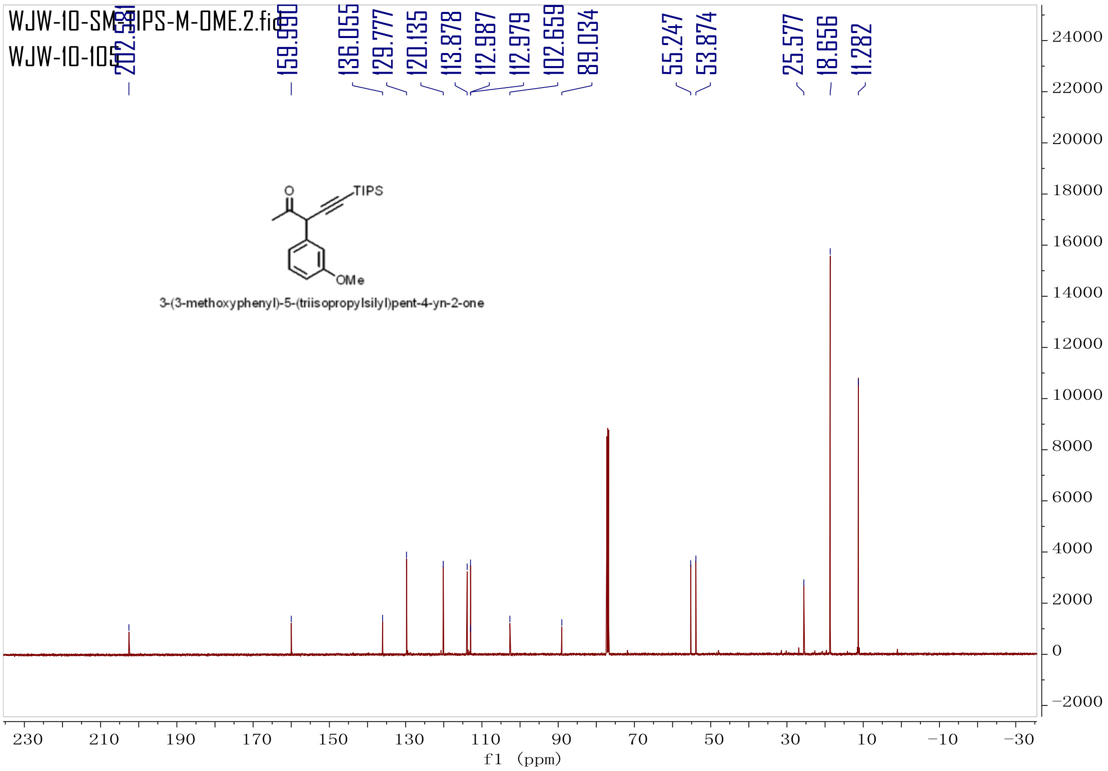

Supplementary Fig. 114 ^{13}C NMR spectrum of **1h**

3-(naphthalen-2-yl)-5-(triisopropylsilyl)pent-4-yn-2-one (**1i**)

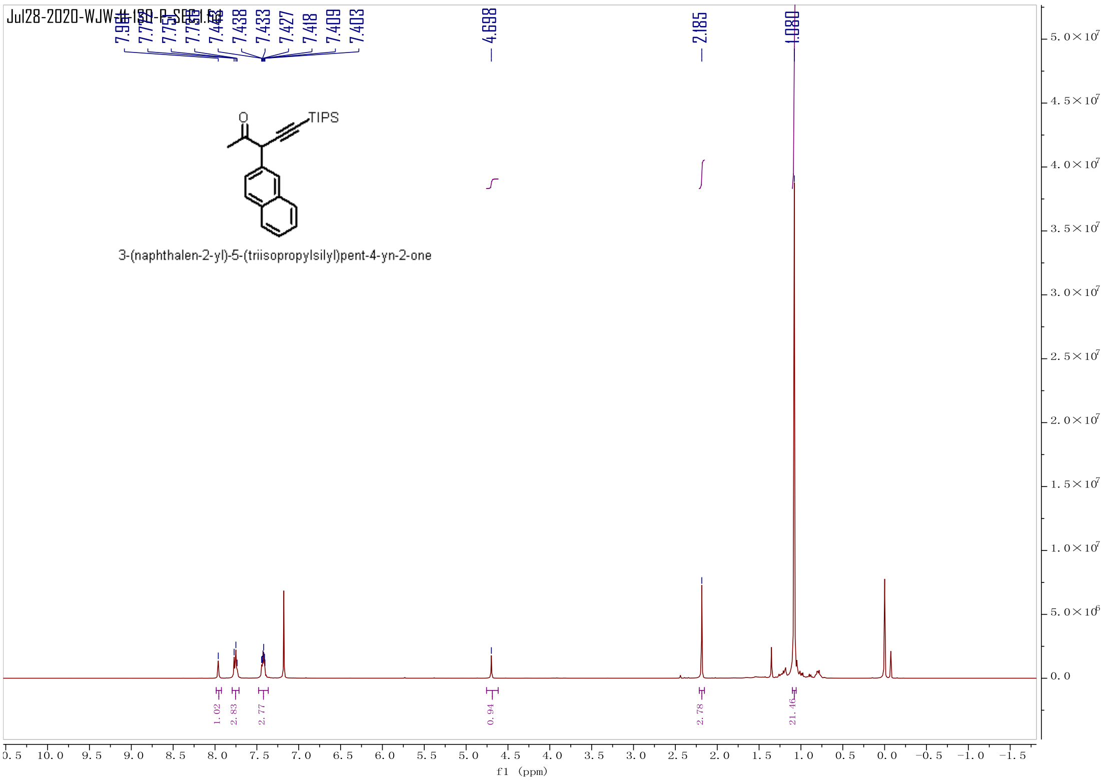

Supplementary Fig. 115 ^{1}H NMR spectrum of **1i**

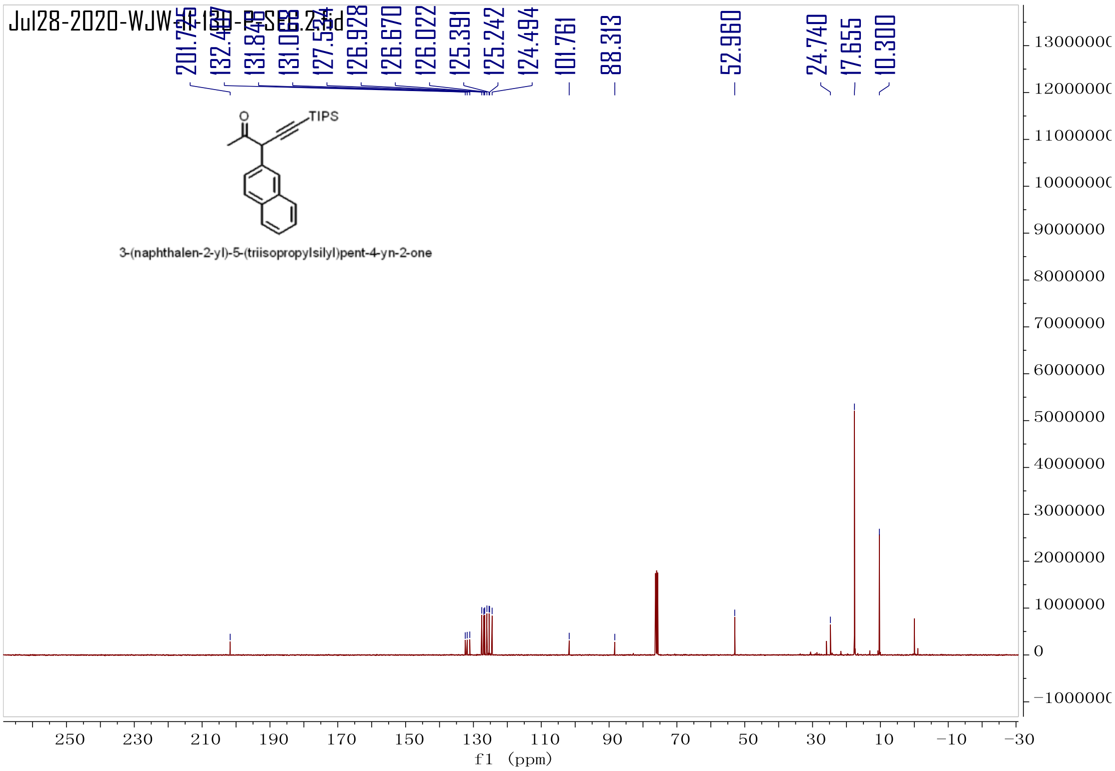

Supplementary Fig. 116 ^{13}C NMR spectrum of **1i**

3-methyl-5-(triisopropylsilyl)pent-4-yn-2-one (**1j**)

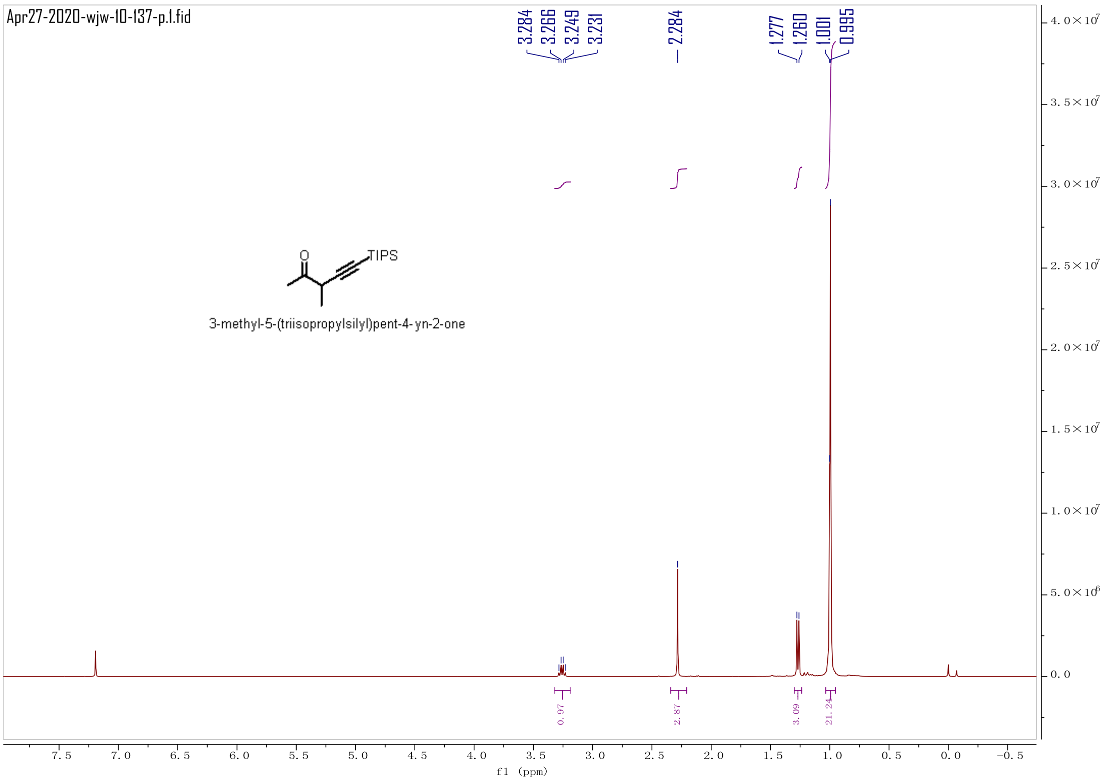

Supplementary Fig. 117 ^{1}H NMR spectrum of **1j**

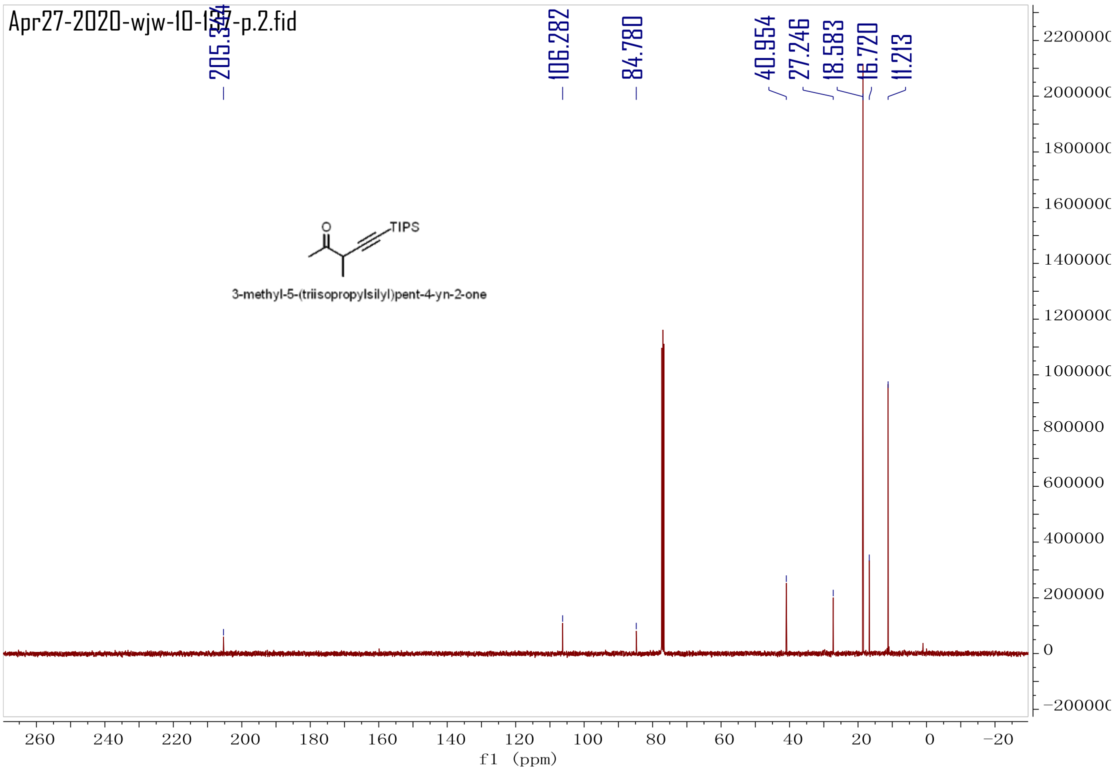

Supplementary Fig. 118 ^{13}C NMR spectrum of **1j**

3-phenyl-5-(p-tolyl)pent-4-yn-2-one (**1k**)

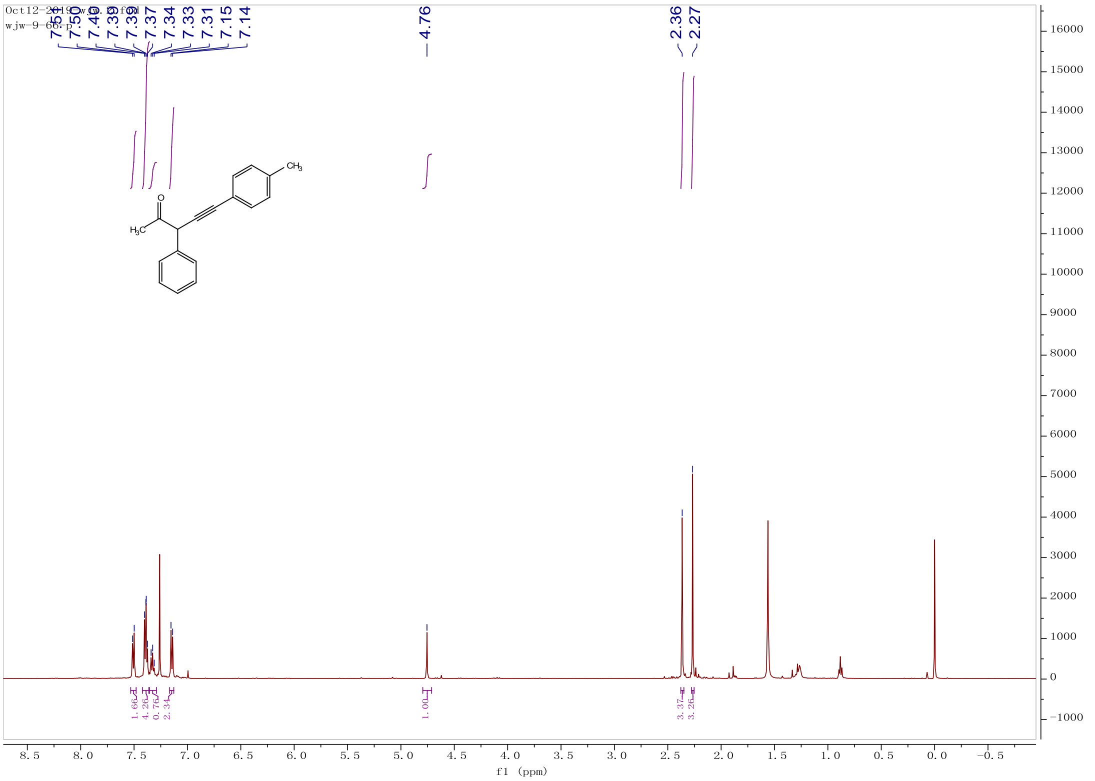

Supplementary Fig. 119 ^{1}H NMR spectrum of **1k**

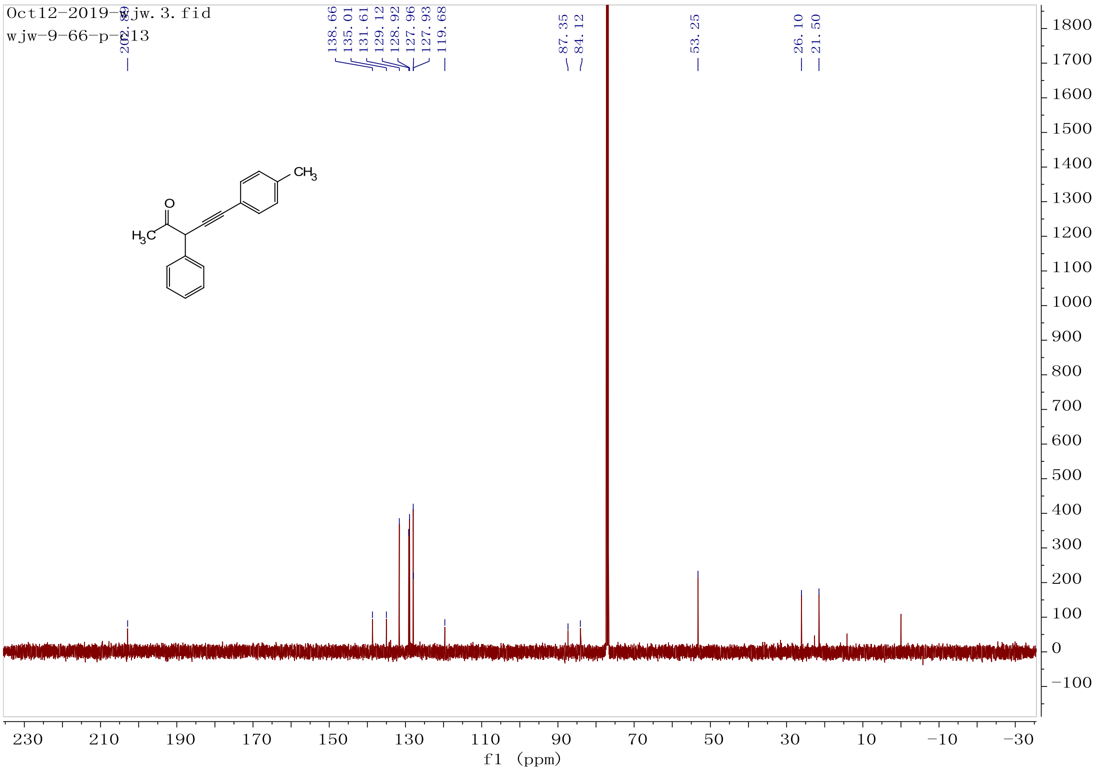

Supplementary Fig. 120 ^{13}C NMR spectrum of **1k**

3-ethyl-5-phenylpent-4-yn-2-one **(1l)**

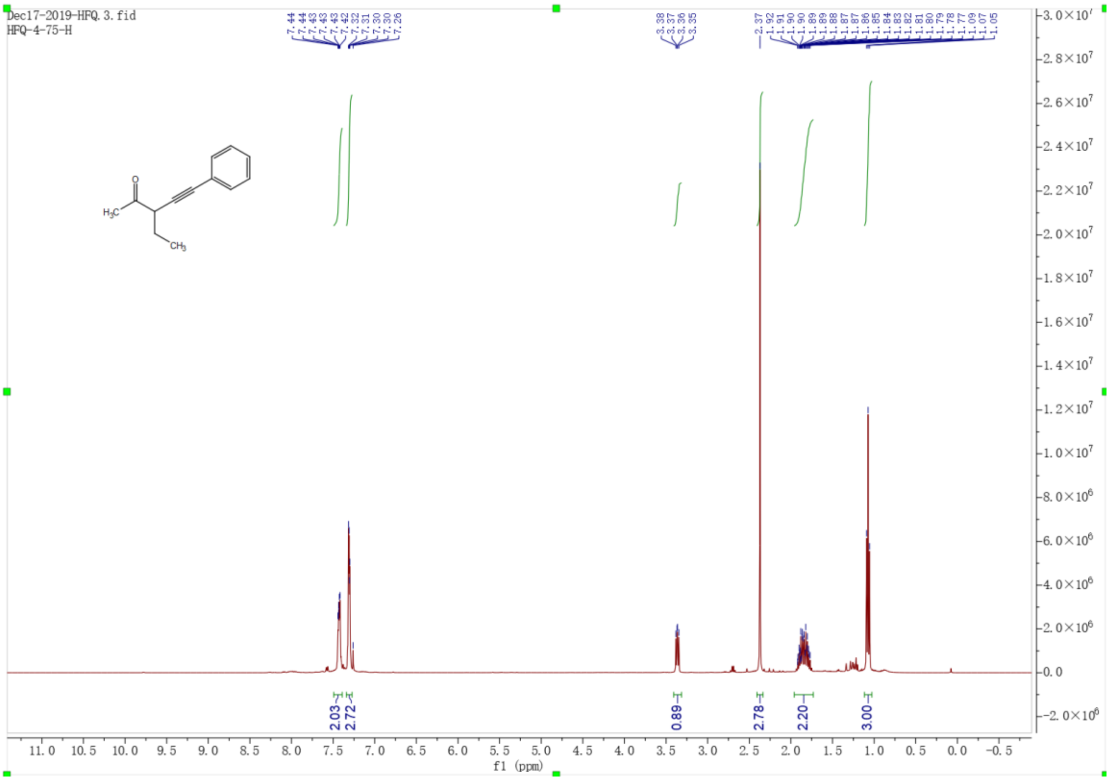

Supplementary Fig. 121 ^{1}H NMR spectrum of **1l**

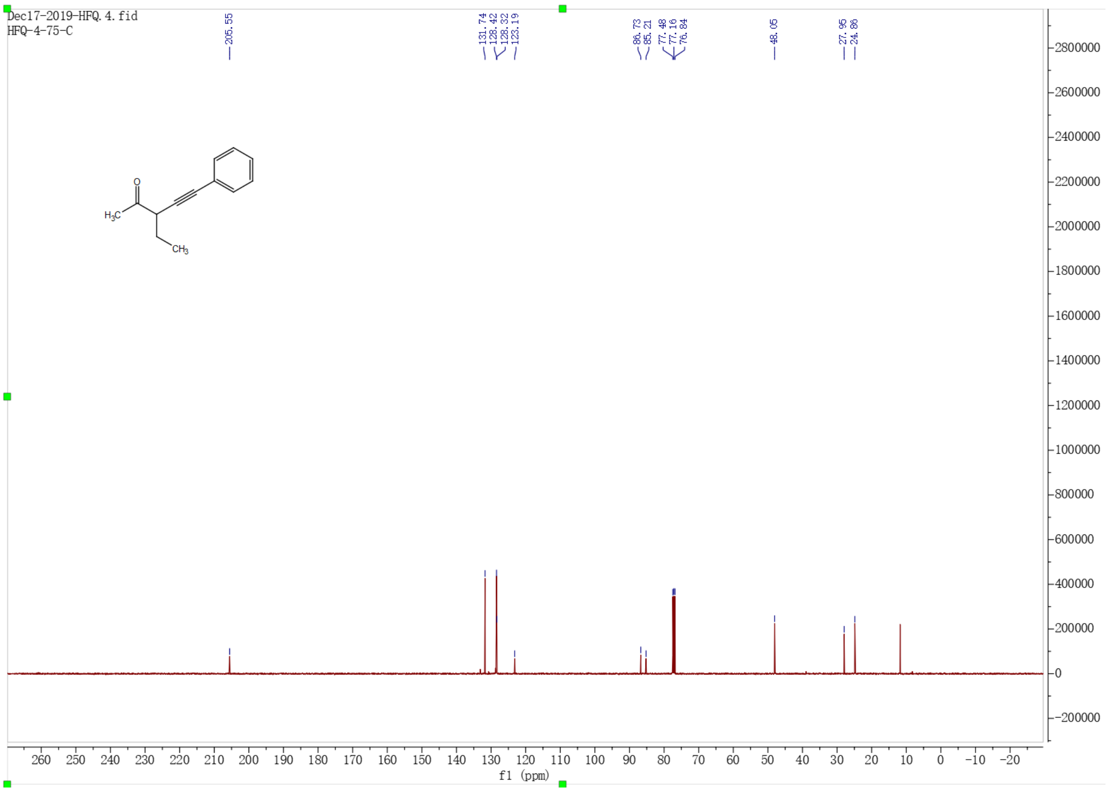

Supplementary Fig. 122 ^{13}C NMR spectrum of **1l**

4-phenyl-6-(triisopropylsilyl)hex-5-yn-3-one (**1o**)

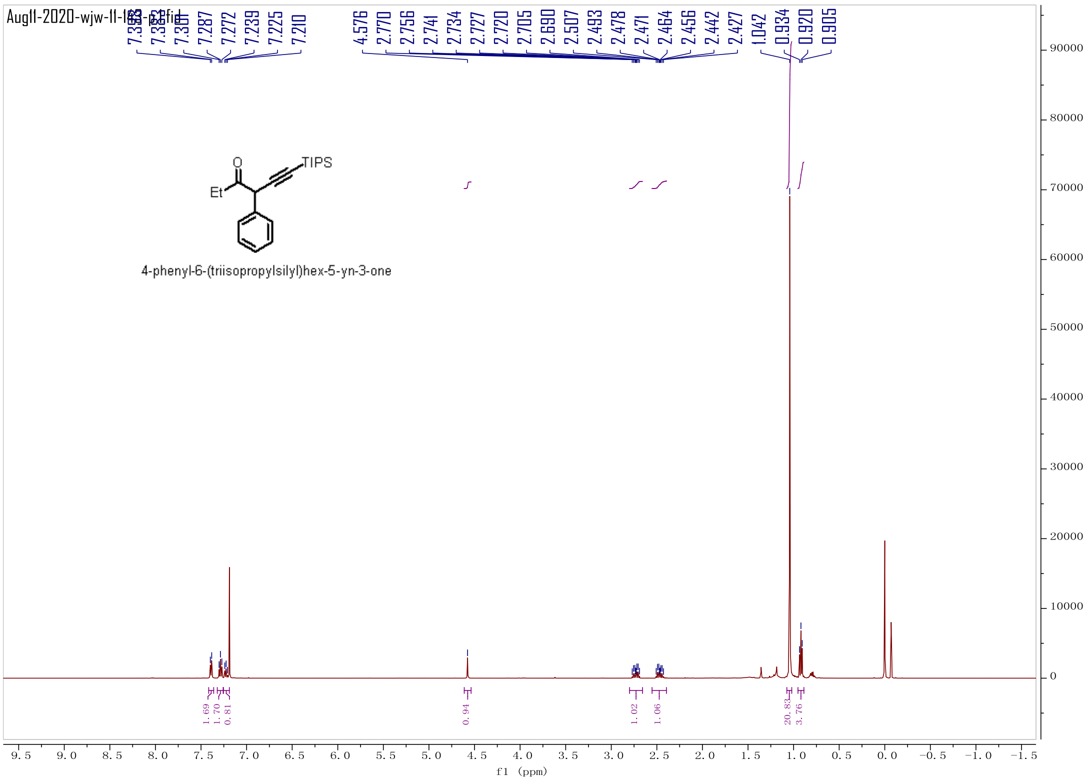

Supplementary Fig. 123 ^{1}H NMR spectrum of **1o**

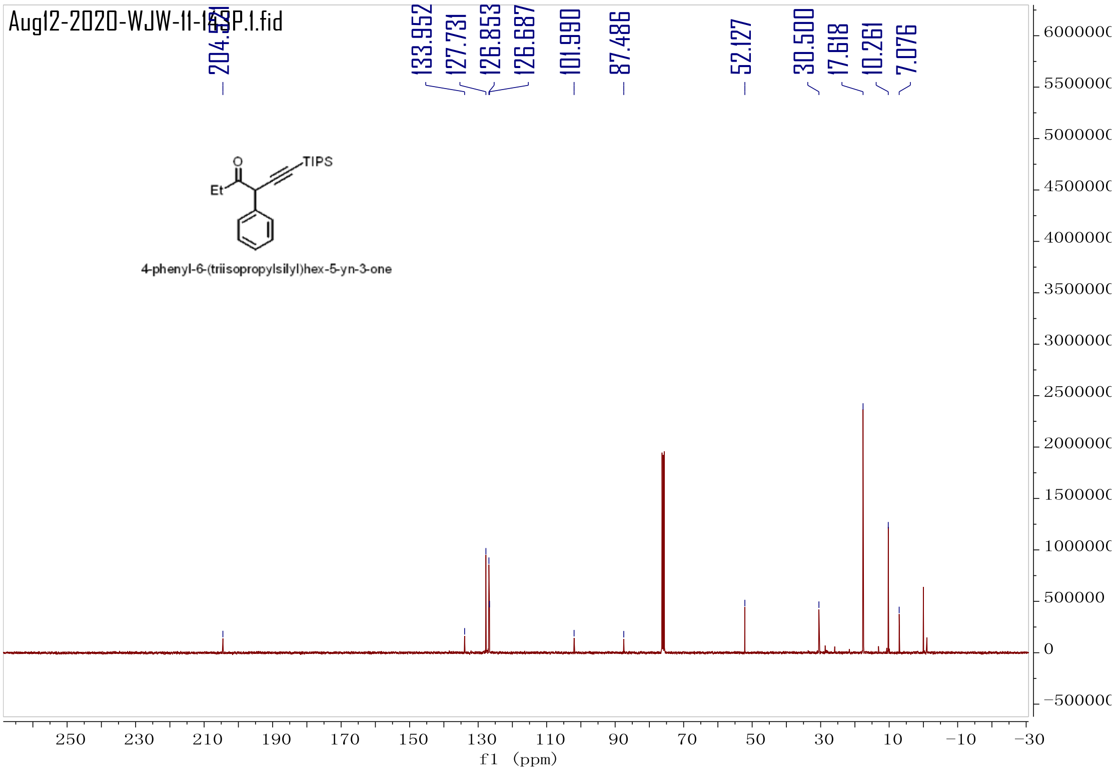

Supplementary Fig. 124 ^{13}C NMR spectrum of **1o**

3-phenyl-1-(triisopropylsilyl)oct-1-yn-4-one (**1p**)

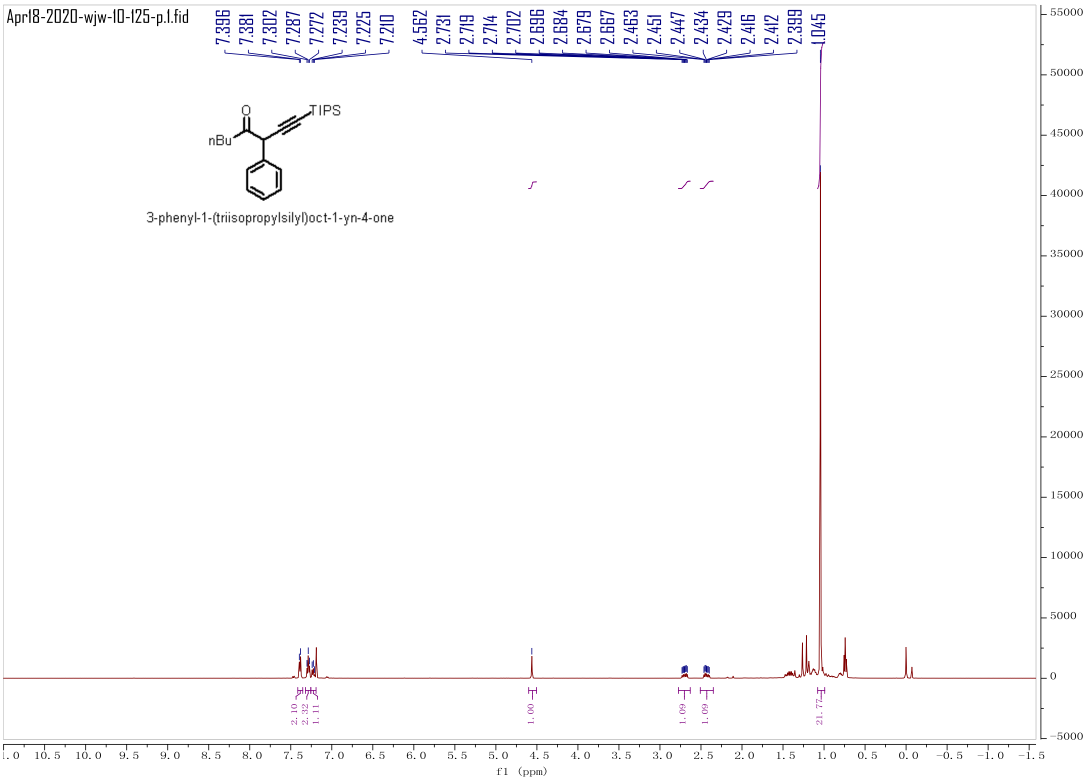

Supplementary Fig. 125 ^{1}H NMR spectrum of **1p**

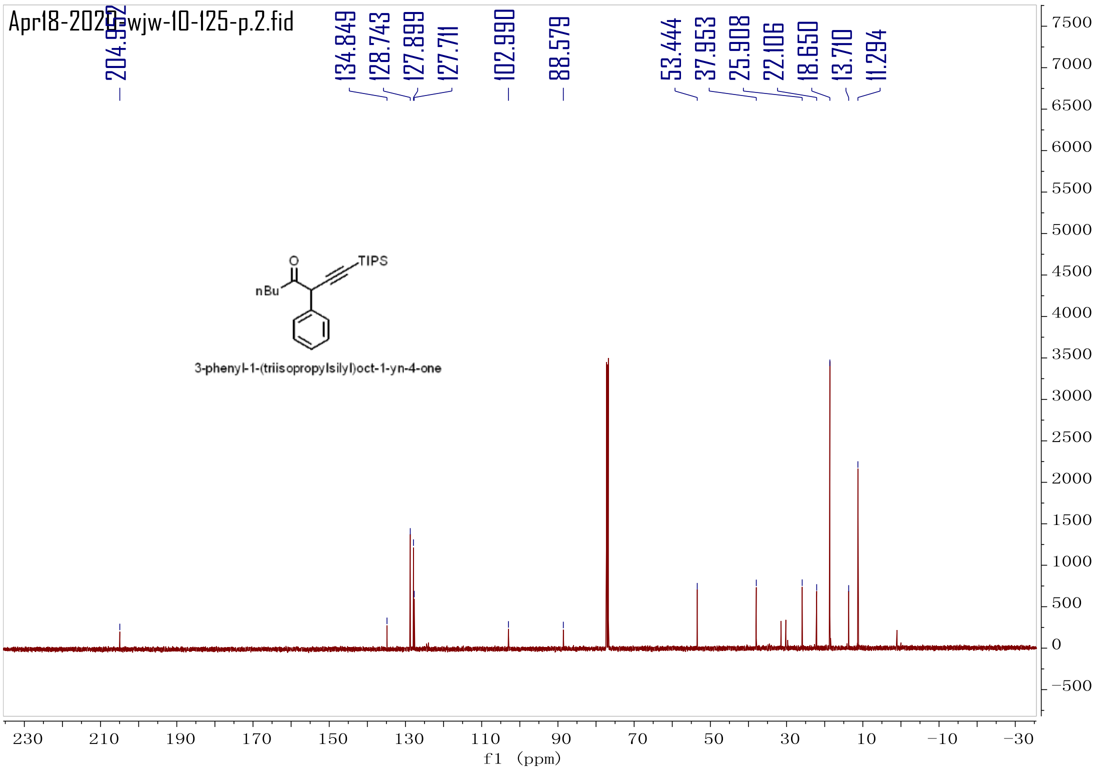

Supplementary Fig. 126 ^{13}C NMR spectrum of **1p**

2-methyl-4-phenyl-6-(triisopropylsilyl)hex-5-yn-3-one (**1q**)

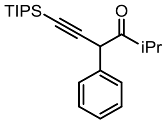

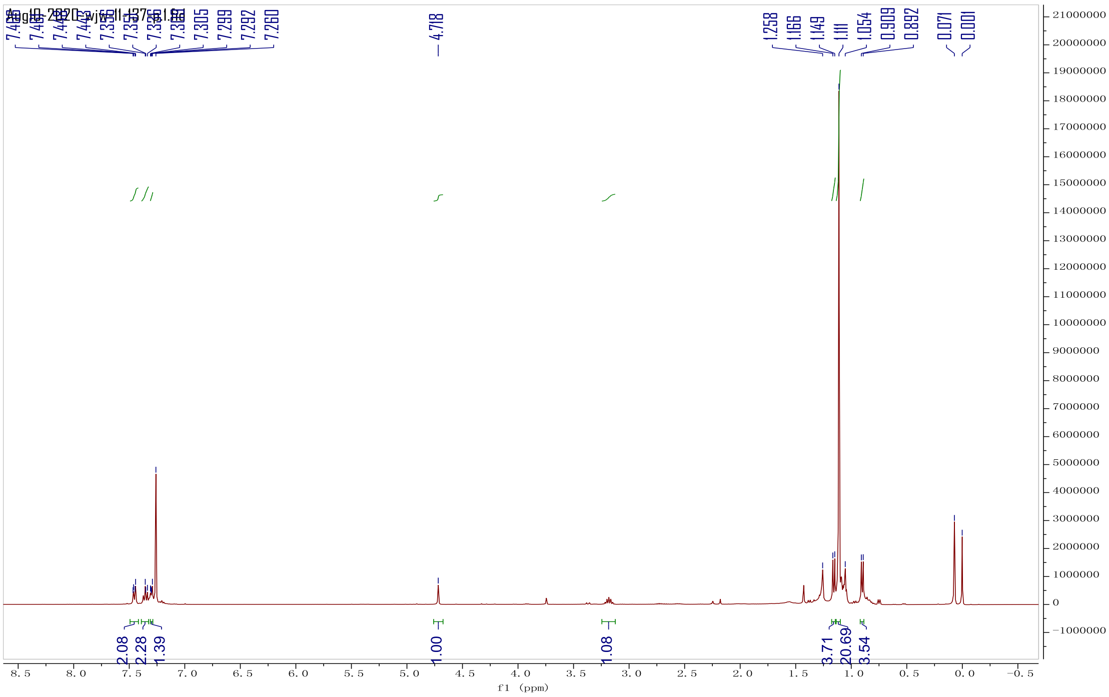

Supplementary Fig. 127 ^{1}H NMR spectrum of **1q**

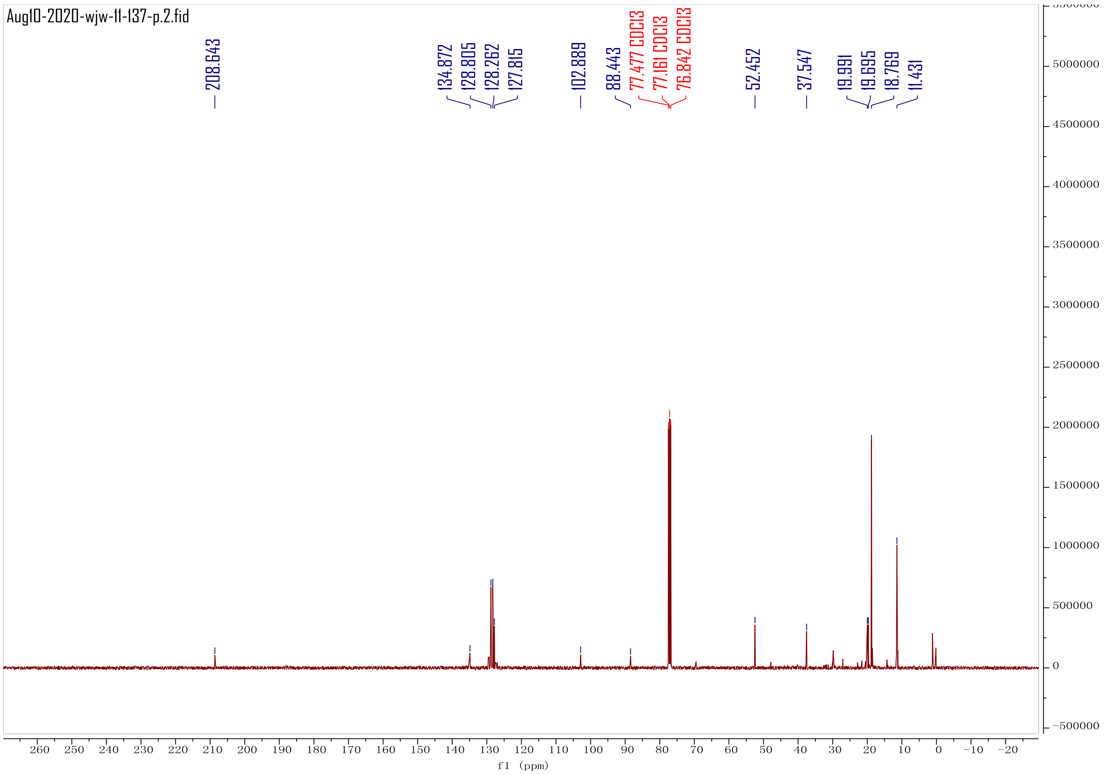

Supplementary Fig. 128 ^{13}C NMR spectrum of **1q**

N-(propa-1,2-dien-1-yl)-N-(m-tolyl)pivalamide (**2a**)

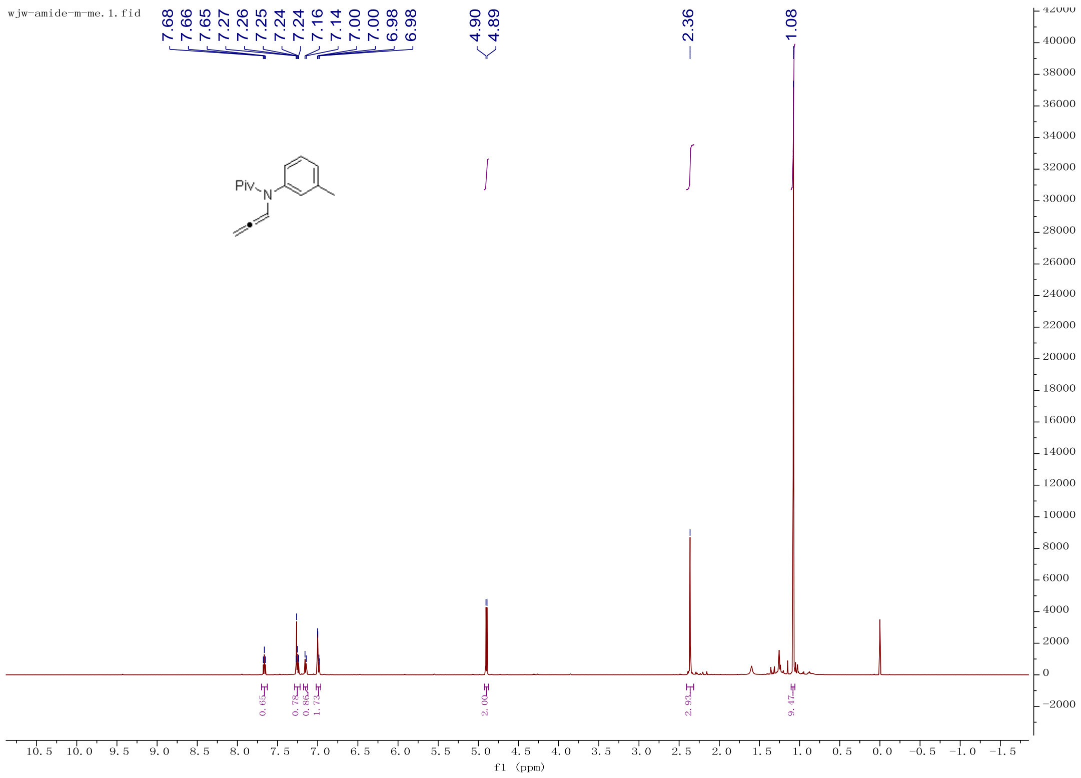

Supplementary Fig. 129 ^{1}H NMR spectrum of **2a**

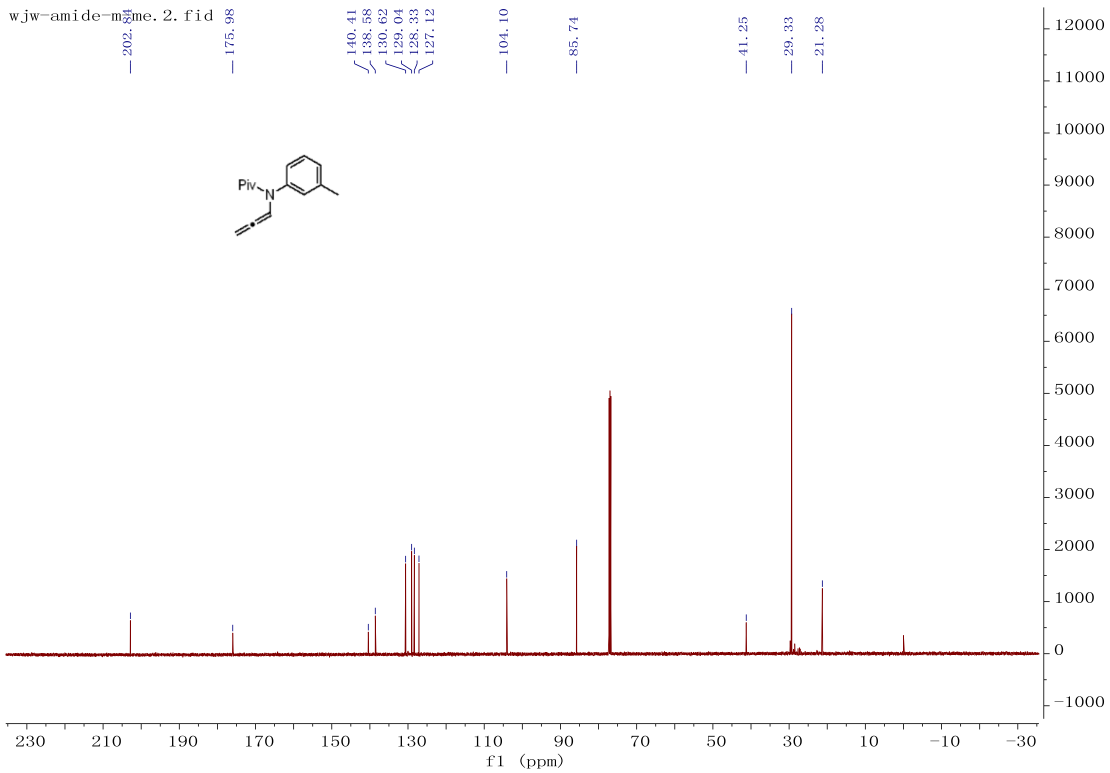

Supplementary Fig. 130 ^{13}C NMR spectrum of **2a**

N-(propa-1,2-dien-1-yl)-N-(p-tolyl)pivalamide (**2r**)

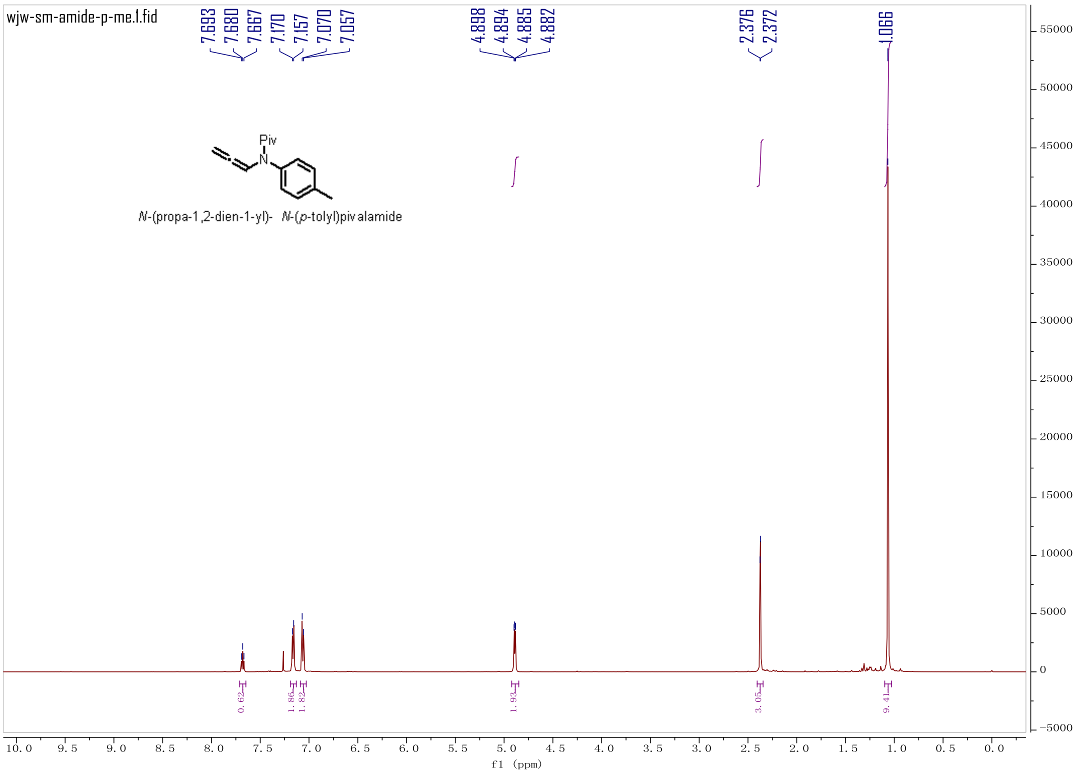

Supplementary Fig. 131 ^{1}H NMR spectrum of **2r**

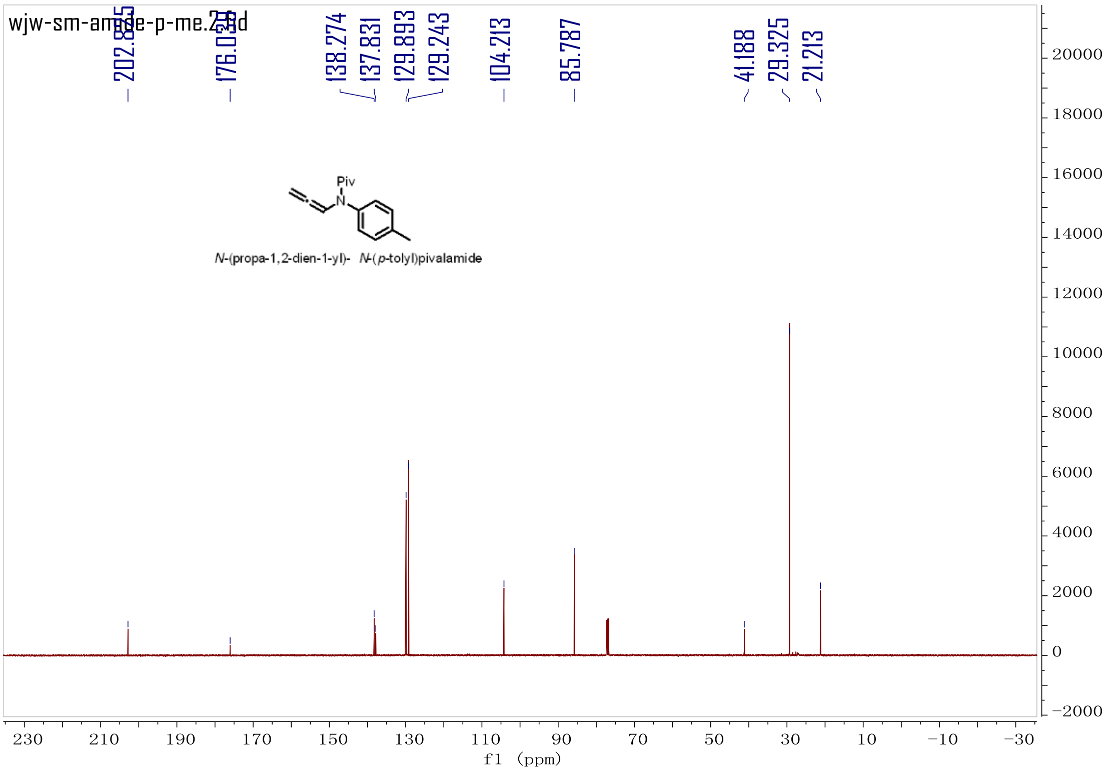

Supplementary Fig. 132 ^{13}C NMR spectrum of **2r**

N-(4-chlorophenyl)-N-(propa-1,2-dien-1-yl)pivalamide (**2t**)

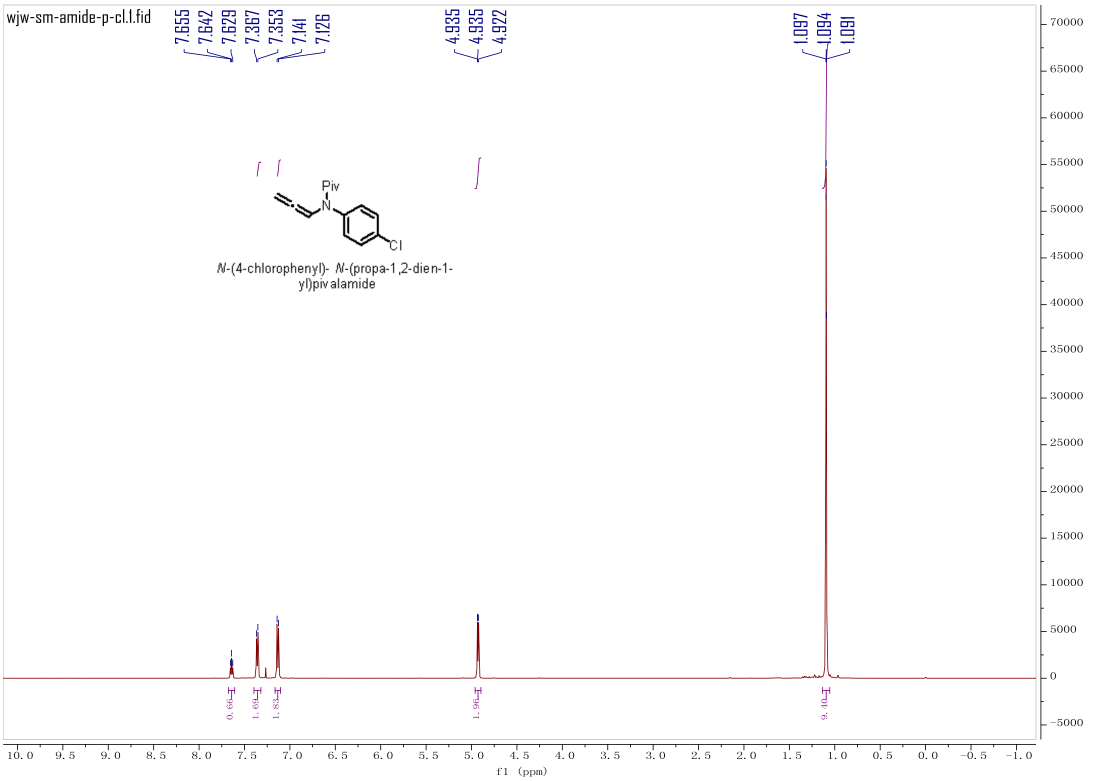

Supplementary Fig. 133 ^{1}H NMR spectrum of **2t**

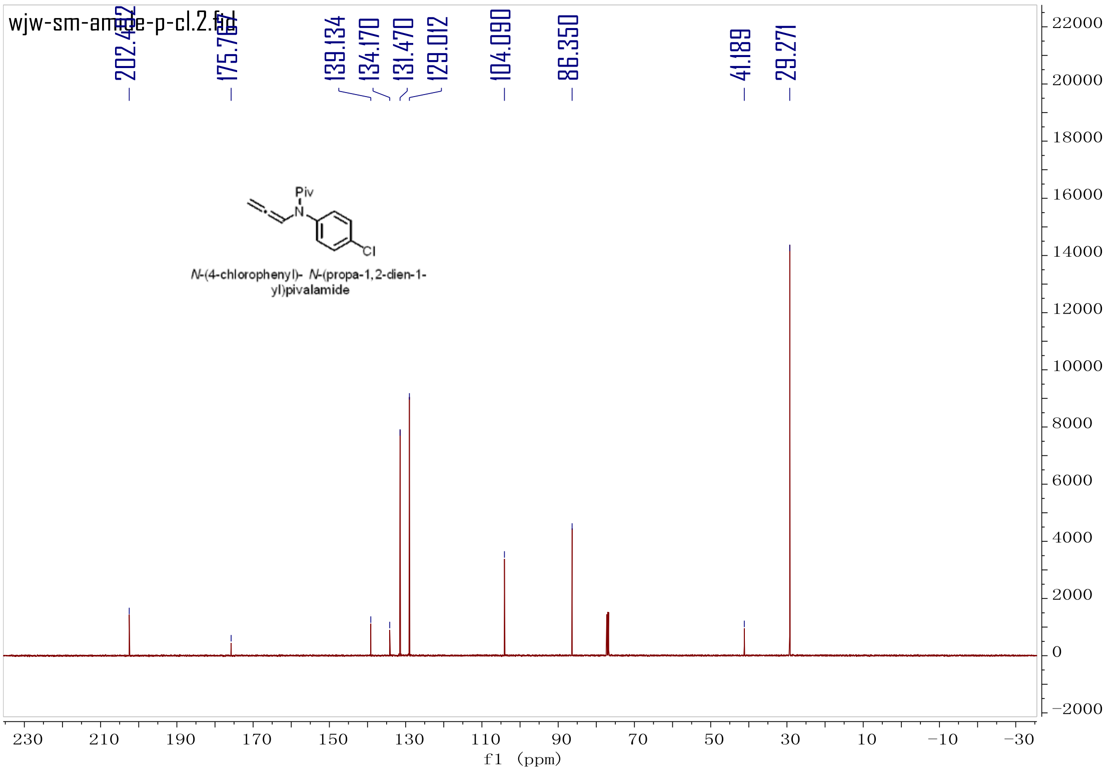

Supplementary Fig. 134 ^{13}C NMR spectrum of **2t**

N-(propa-1,2-dien-1-yl)-N-(4-(trifluoromethyl)phenyl)pivalamide (**2u**)

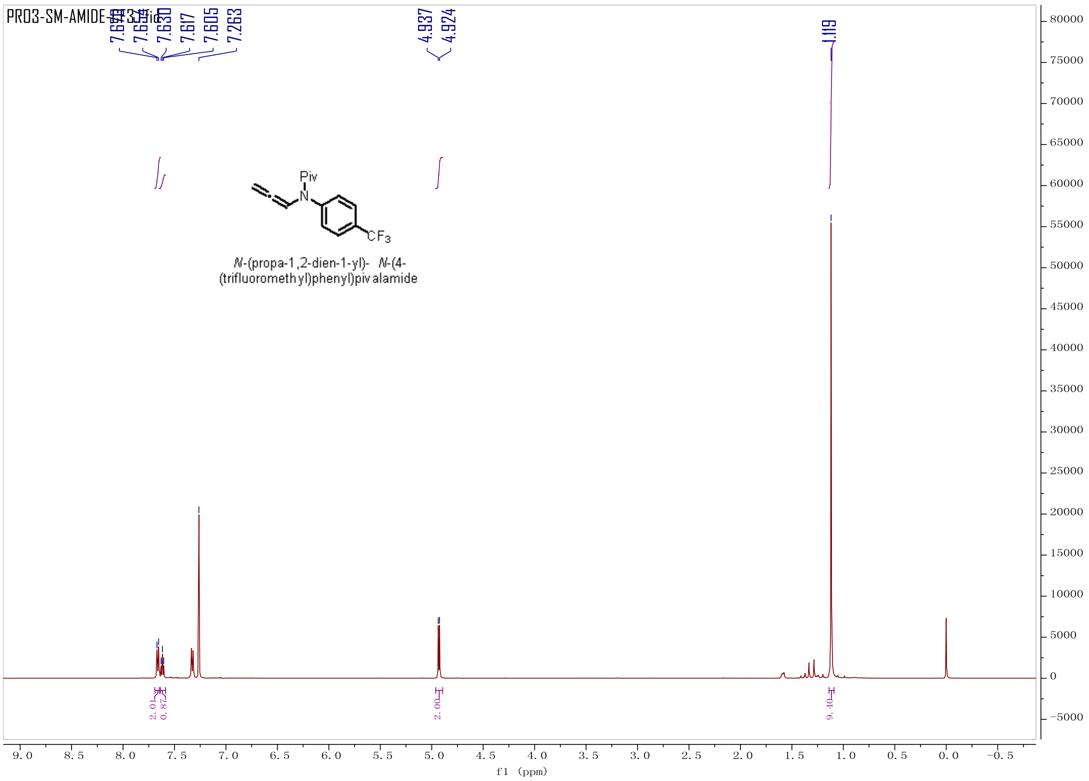

Supplementary Fig. 135 ^{1}H NMR spectrum of **2u**

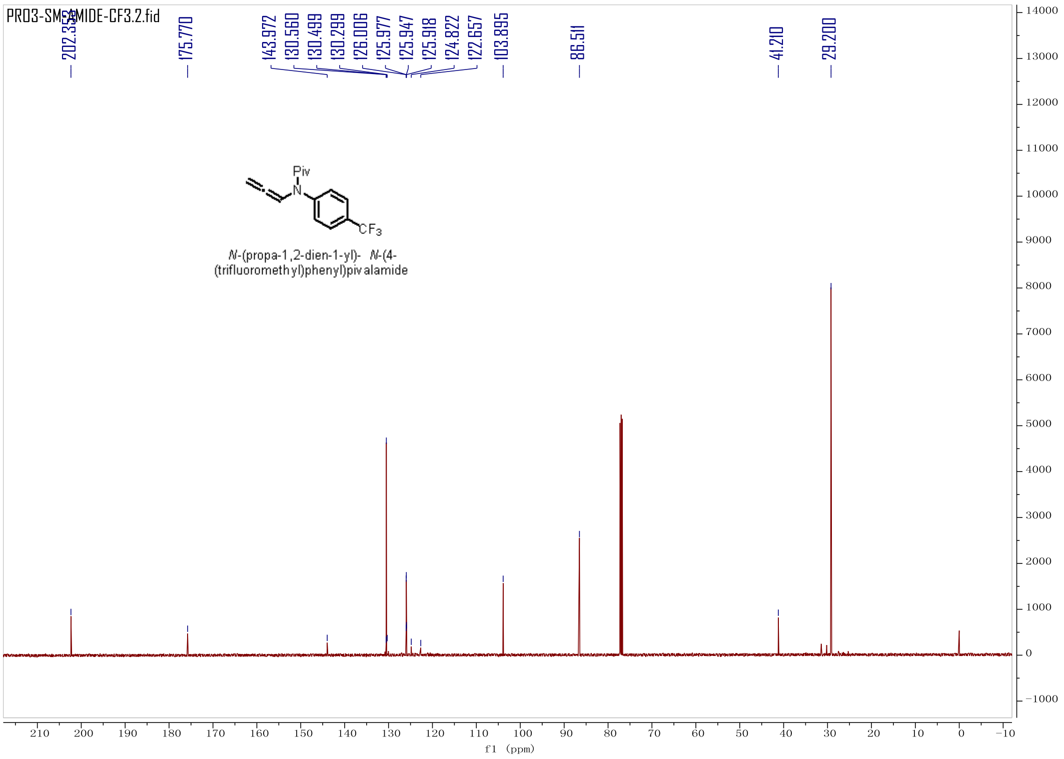

Supplementary Fig. 136 ^{13}C NMR spectrum of **2u**

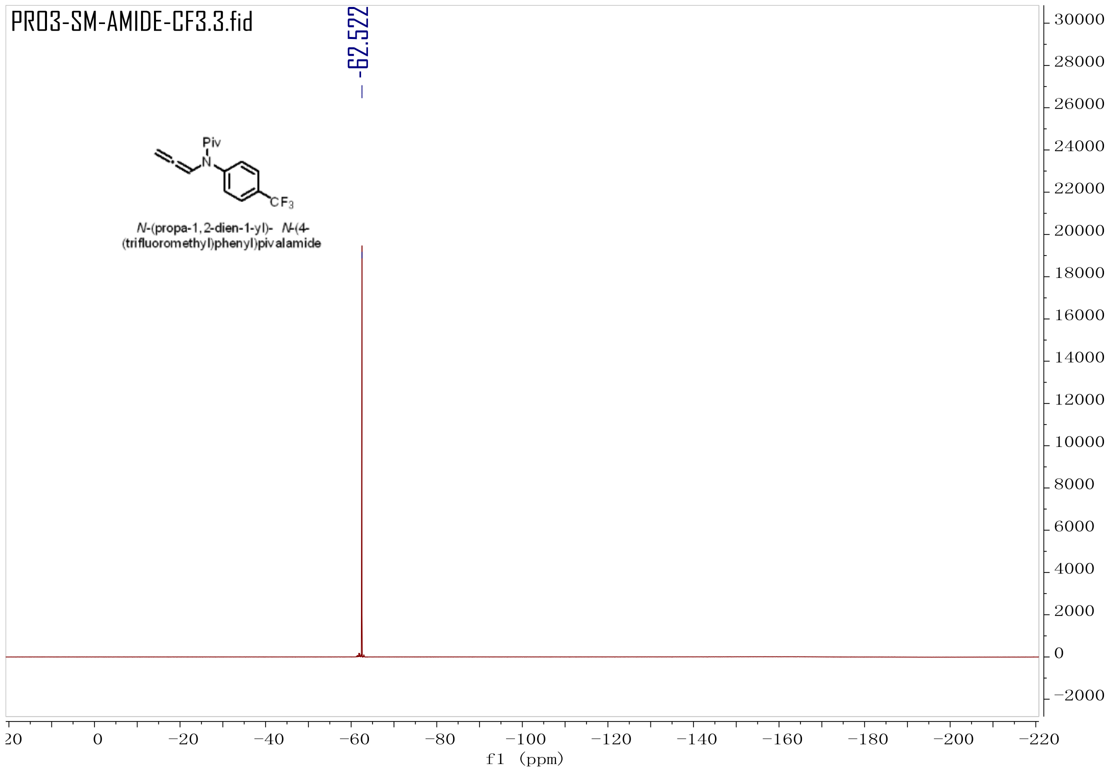

Supplementary Fig. 137 ^{19}F NMR spectrum of **2u**

N-(3-methoxyphenyl)-N-(propa-1,2-dien-1-yl)pivalamide (**2v**)

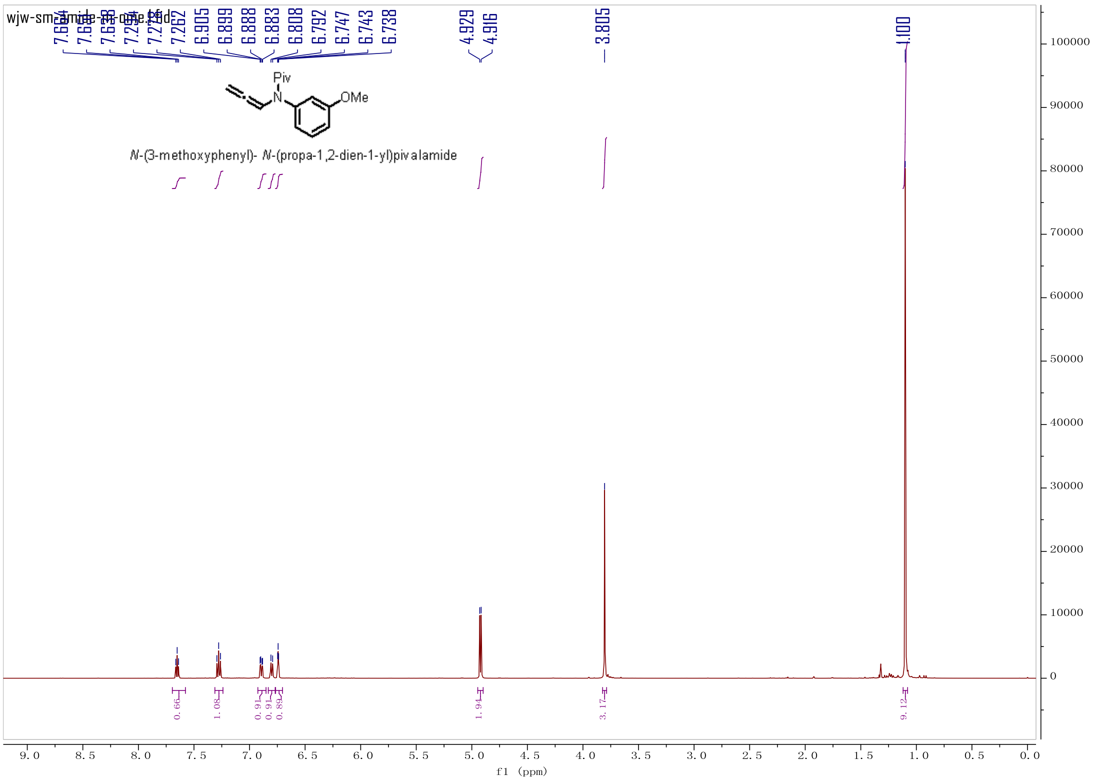

Supplementary Fig. 138 ^{1}H NMR spectrum of **2v**

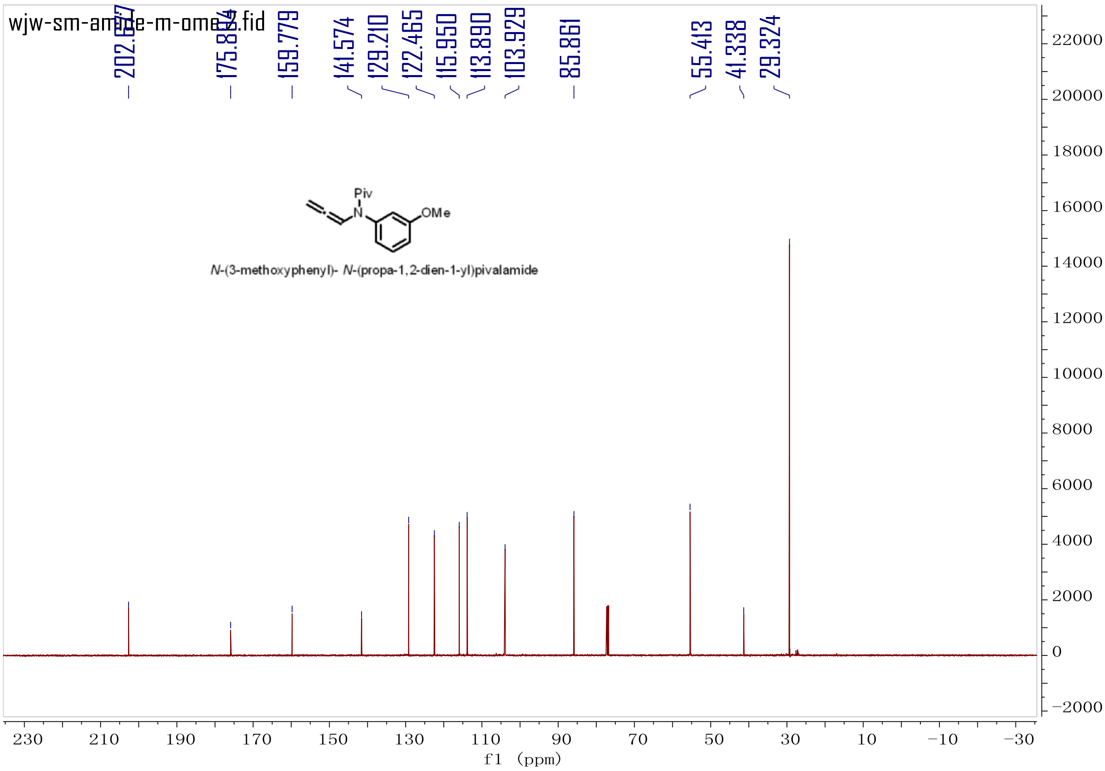

Supplementary Fig. 139 ^{13}C NMR spectrum of **2v**

N-(3-fluorophenyl)-N-(propa-1,2-dien-1-yl)pivalamide (**2w**)

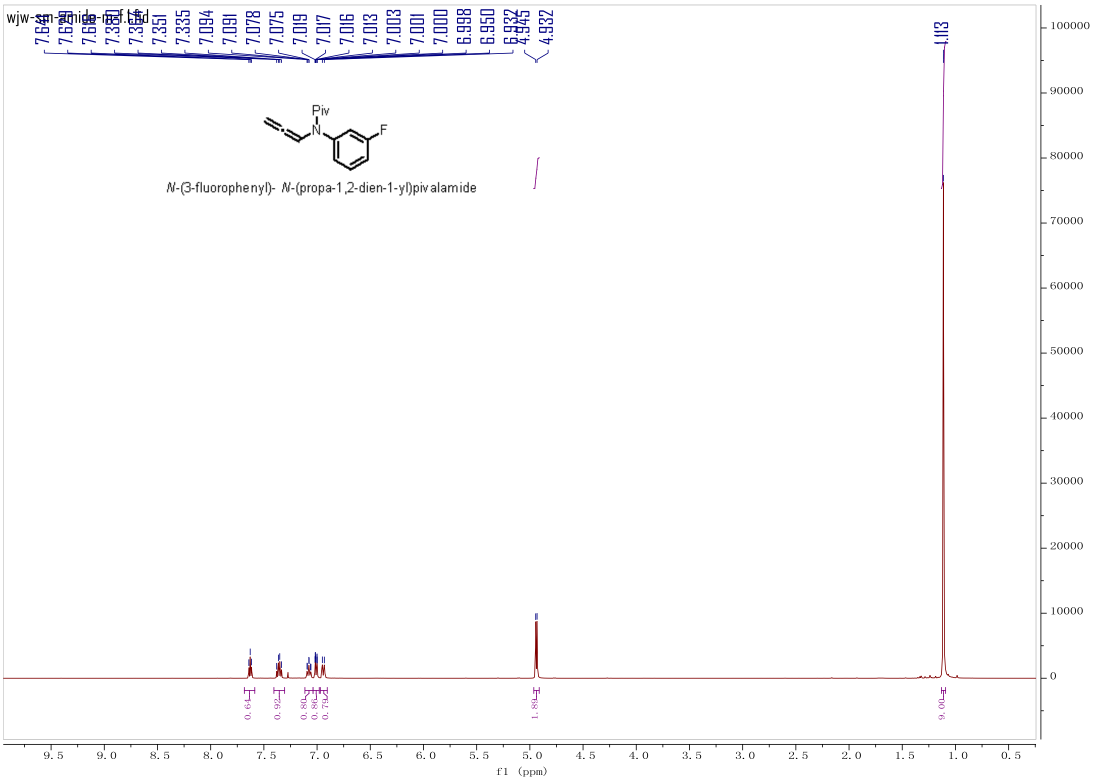

Supplementary Fig. 140 ^{1}H NMR spectrum of **2w**

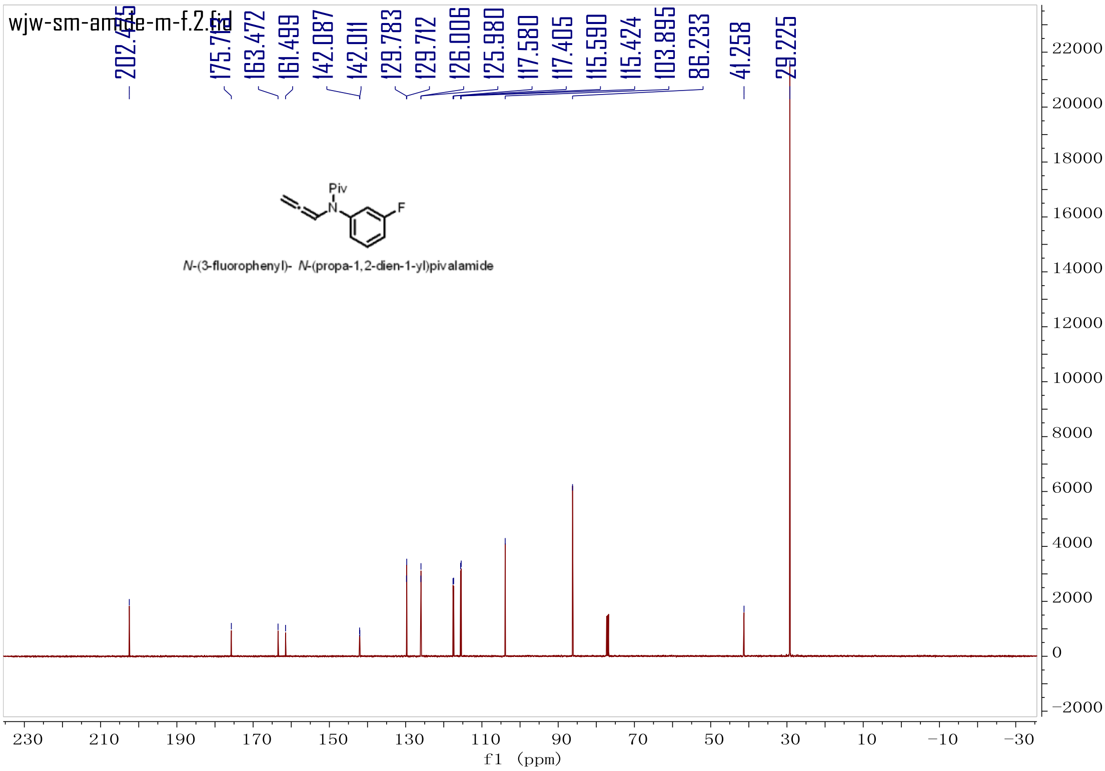

Supplementary Fig. 141 ^{13}C NMR spectrum of **2w**

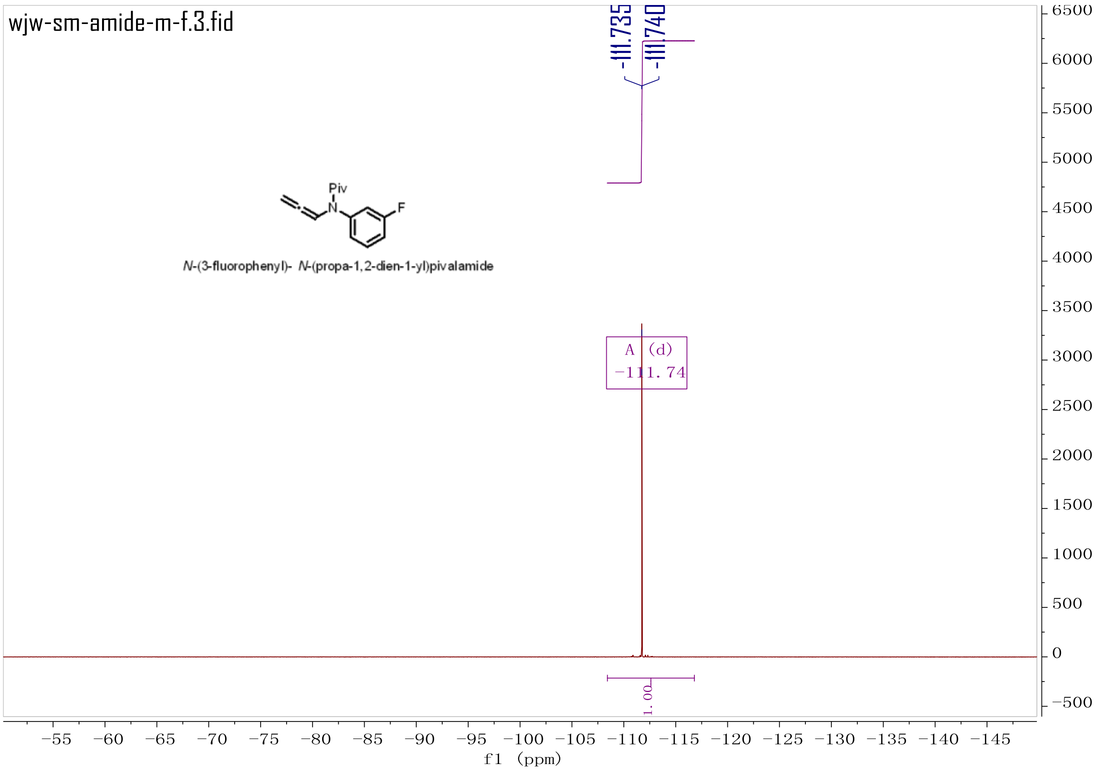

Supplementary Fig. 142 ^{19}F NMR spectrum of **2w**

N-(naphthalen-2-yl)-N-(propa-1,2-dien-1-yl)pivalamide (**2x**)

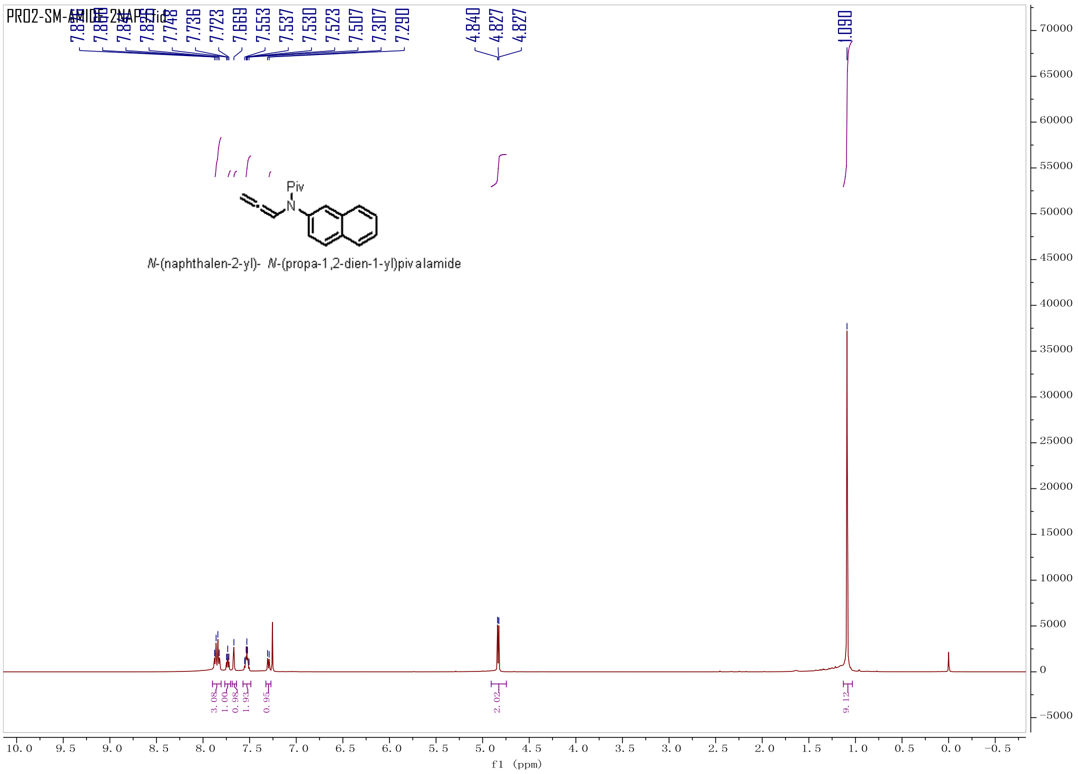

Supplementary Fig. 143 ^{1}H NMR spectrum of **2x**

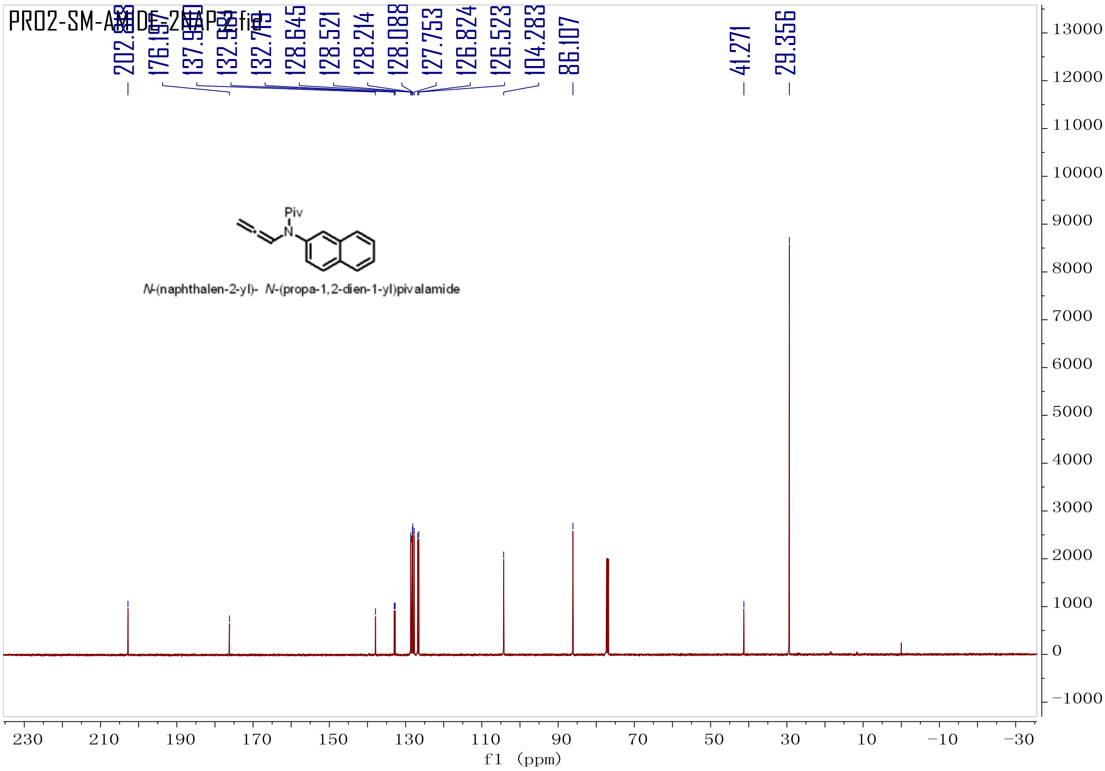

Supplementary Fig. 144 ^{13}C NMR spectrum of **2x**

(*S*,*E*)-N-(4-acetyl-4-phenyl-6-(triisopropylsilyl)hex-1-en-5-yn-1-yl)-N-phenylpivalamide (**3a**)

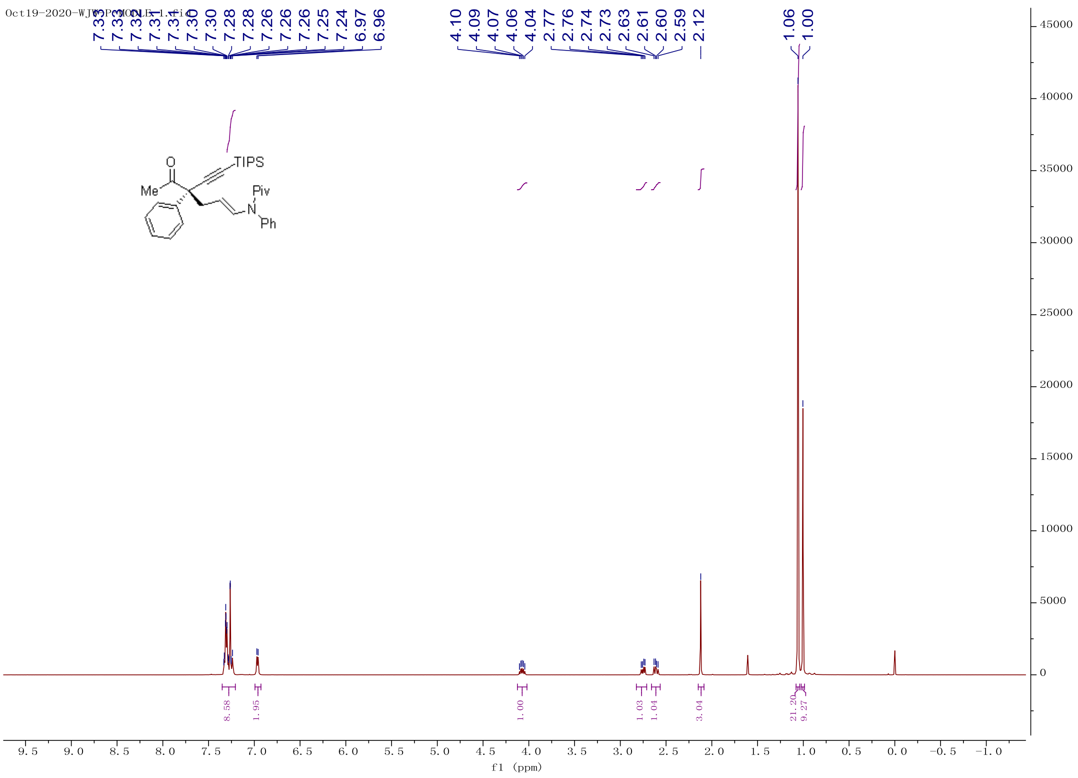

Supplementary Fig. 145 ^{1}H NMR spectrum of **3a**

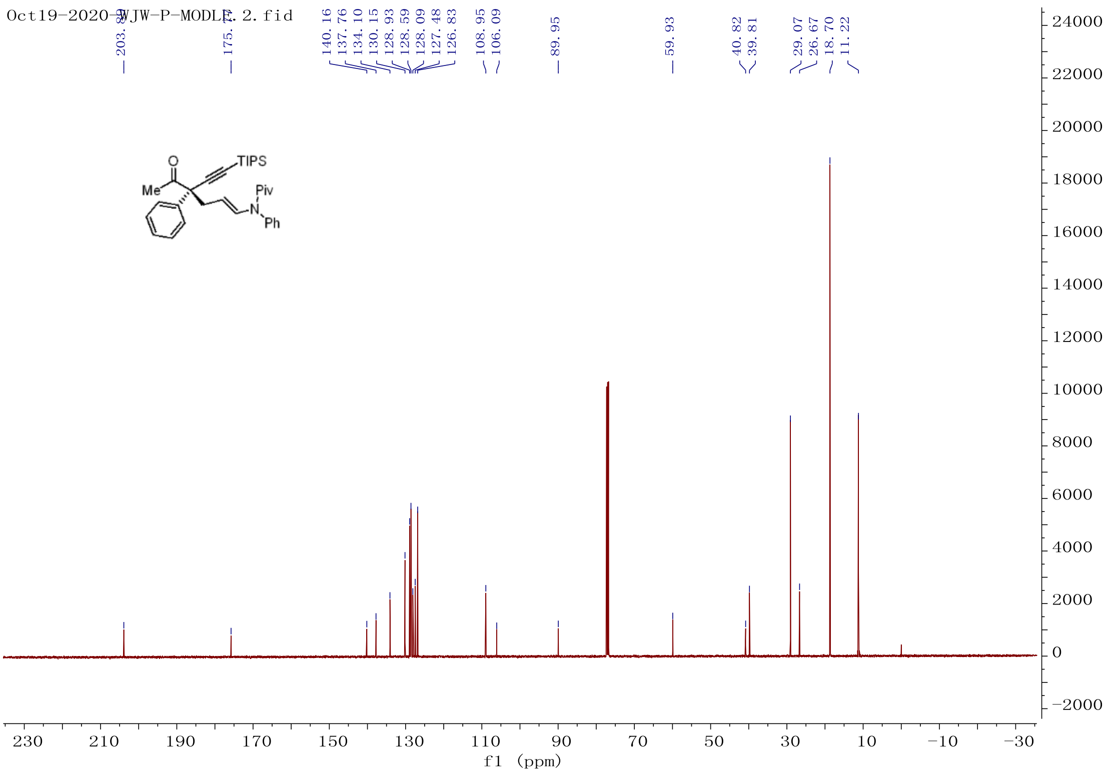

Supplementary Fig. 146 ^{13}C NMR spectrum of **3a**

(*S*,*E*)-N-(4-acetyl-4-(p-tolyl)-6-(triisopropylsilyl)hex-1-en-5-yn-1-yl)-N-phenylpivalamide (**3b**)

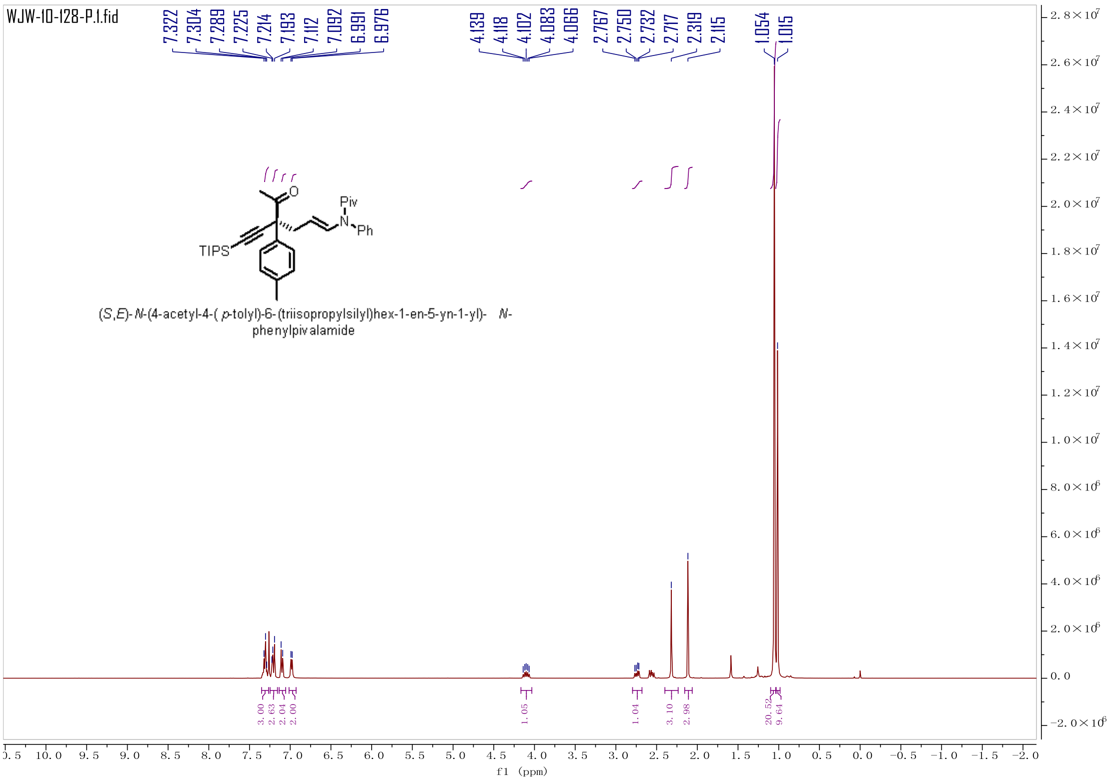

Supplementary Fig. 147 ^{1}H NMR spectrum of **3b**

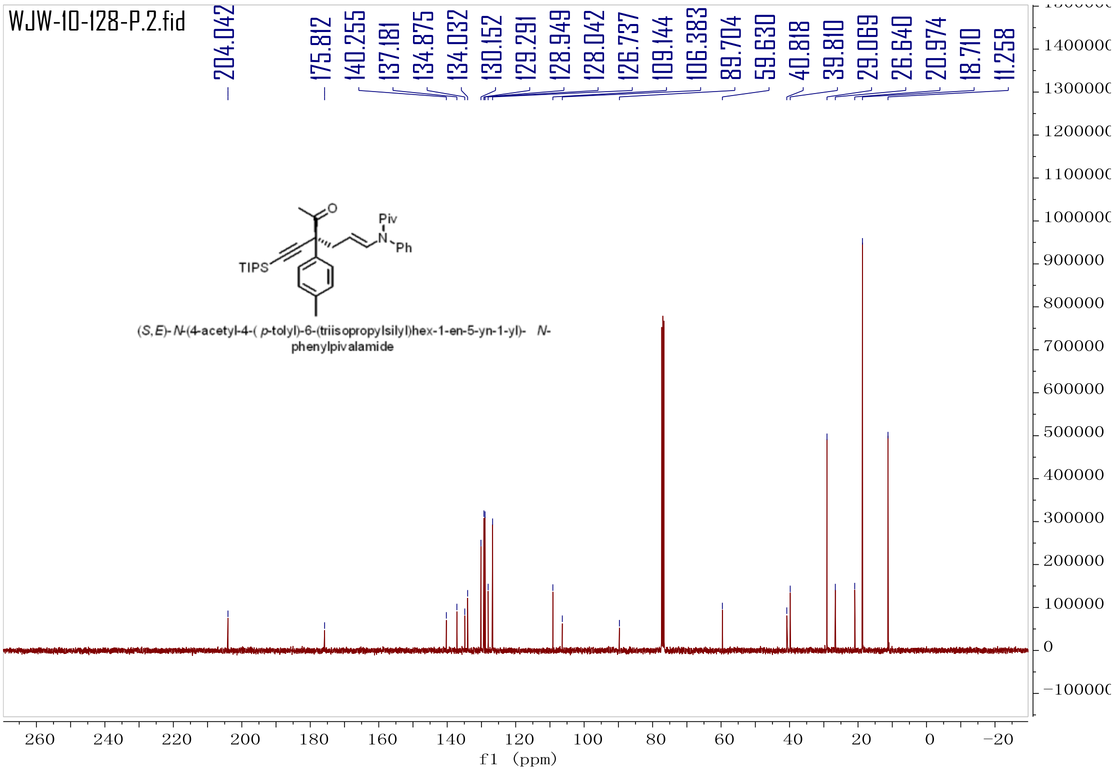

Supplementary Fig. 148 ^{13}C NMR spectrum of **3b**

(*S*,*E*)-N-(4-acetyl-4-(4-chlorophenyl)-6-(triisopropylsilyl)hex-1-en-5-yn-1-yl)-N-phenylpivalamide (**3c**)

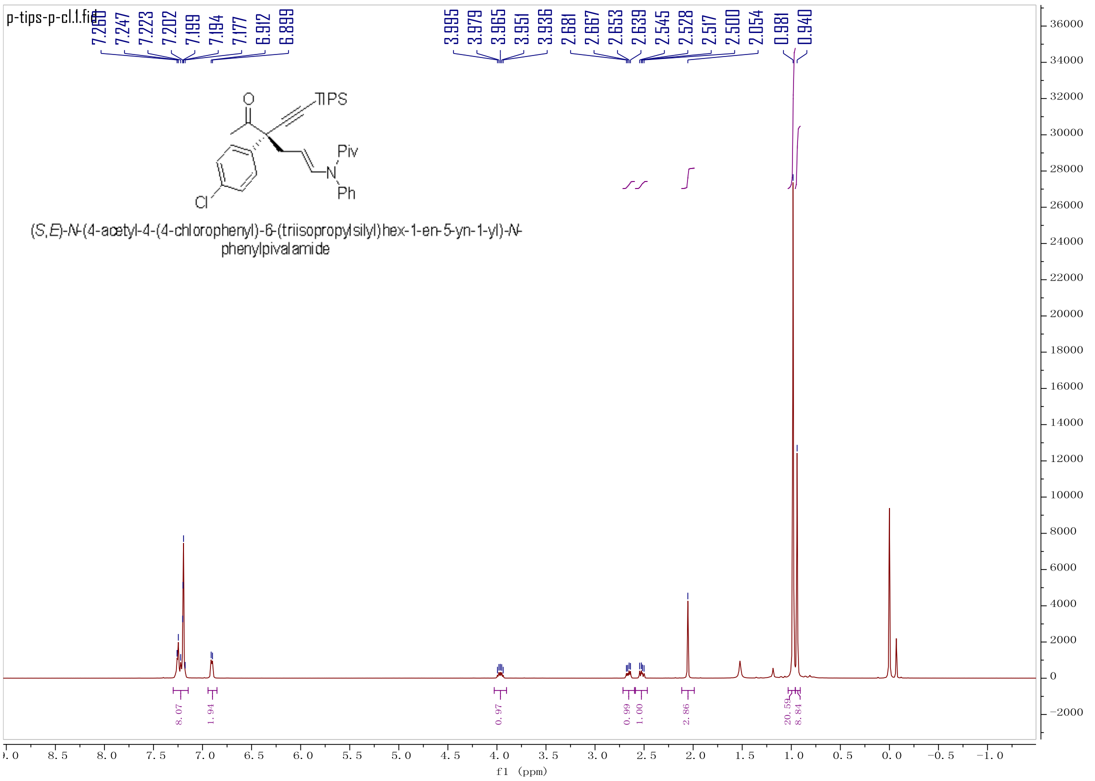

Supplementary Fig. 149 ^{1}H NMR spectrum of **3c**

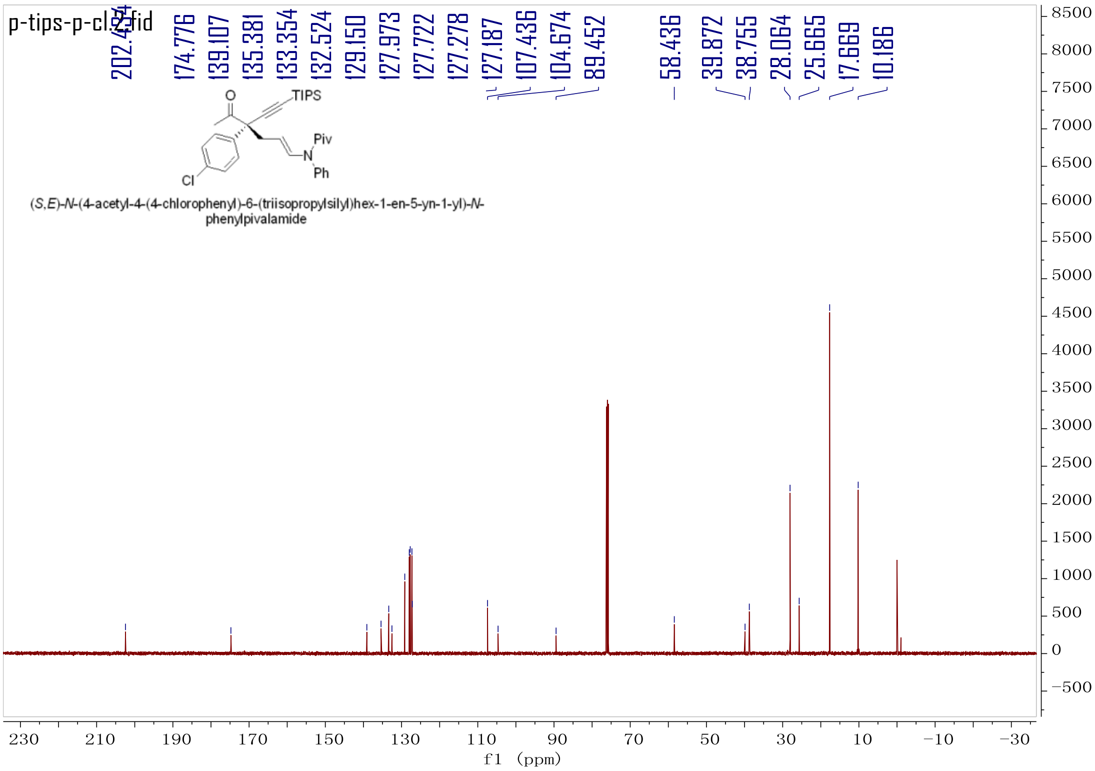

Supplementary Fig. 150 ^{13}C NMR spectrum of **3c**

(*S*,*E*)-N-(4-acetyl-4-(4-(trifluoromethyl)phenyl)-6-(triisopropylsilyl)hex-1-en-5-yn-1-yl)-N-phenyl pivalamide (**3d**)

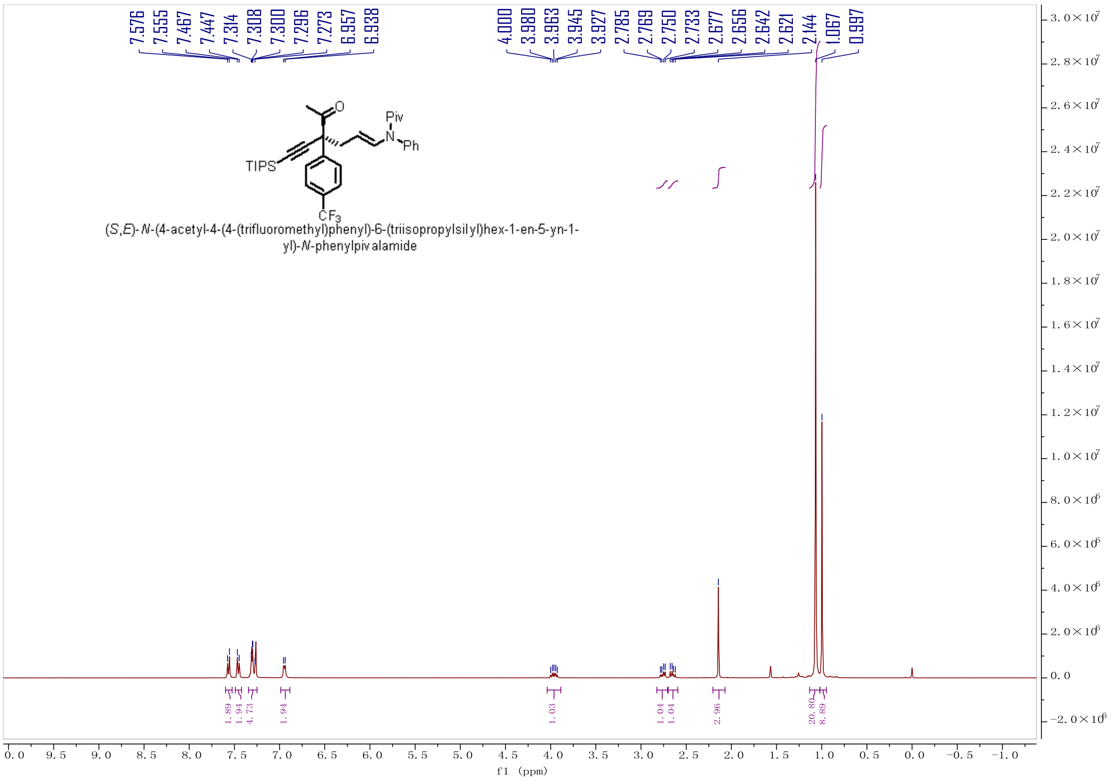

Supplementary Fig. 151 ^{1}H NMR spectrum of **3d**

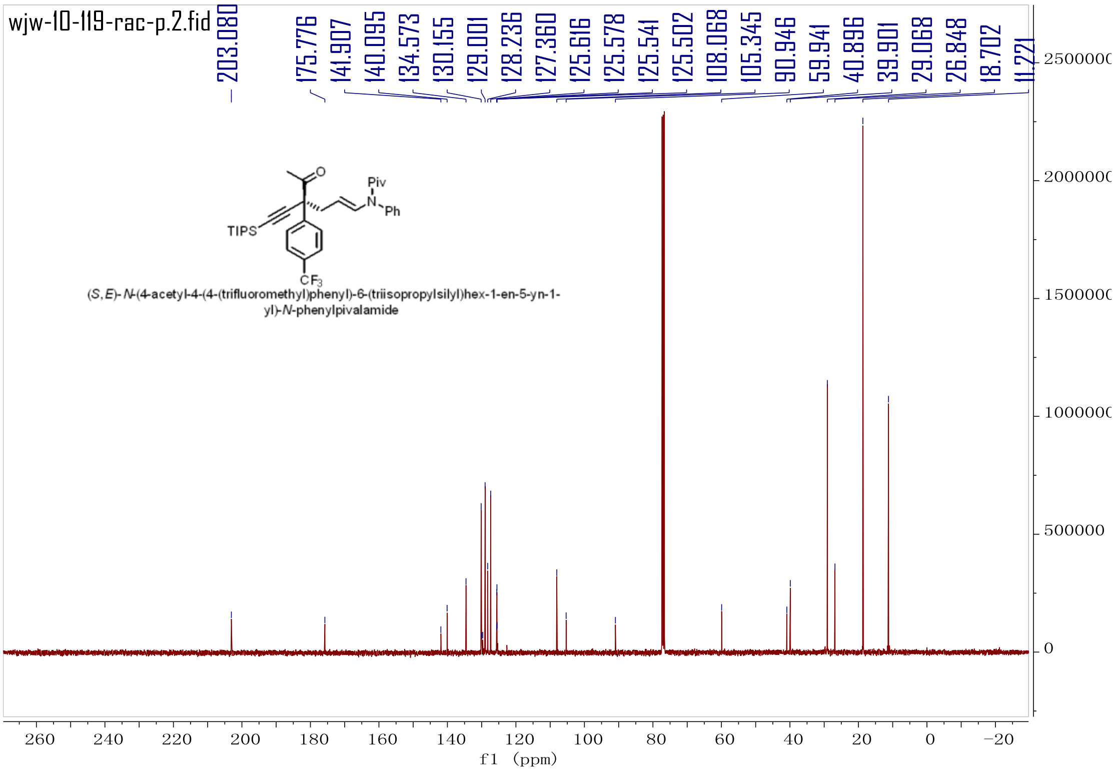

Supplementary Fig. 152 ^{13}C NMR spectrum of **3d**

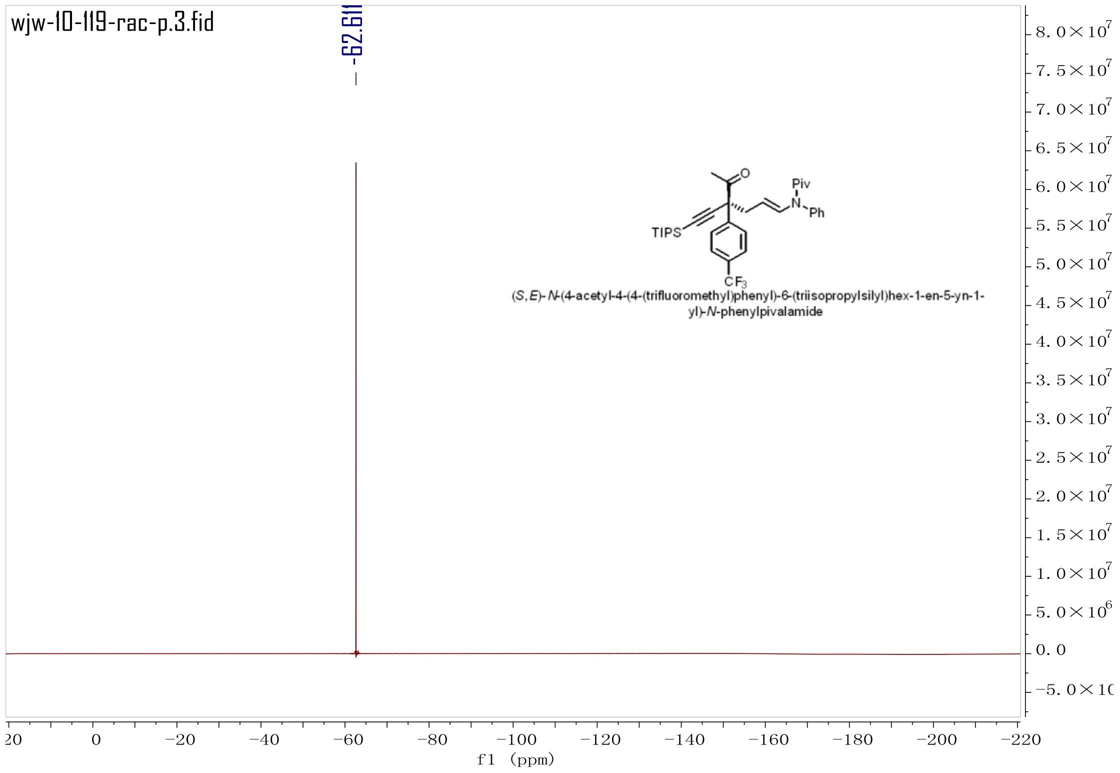

Supplementary Fig. 153 ^{19}F NMR spectrum of **3d**

(*S*,*E*)-N-(4-acetyl-4-(m-tolyl)-6-(triisopropylsilyl)hex-1-en-5-yn-1-yl)-N-phenylpivalamide (**3e**)

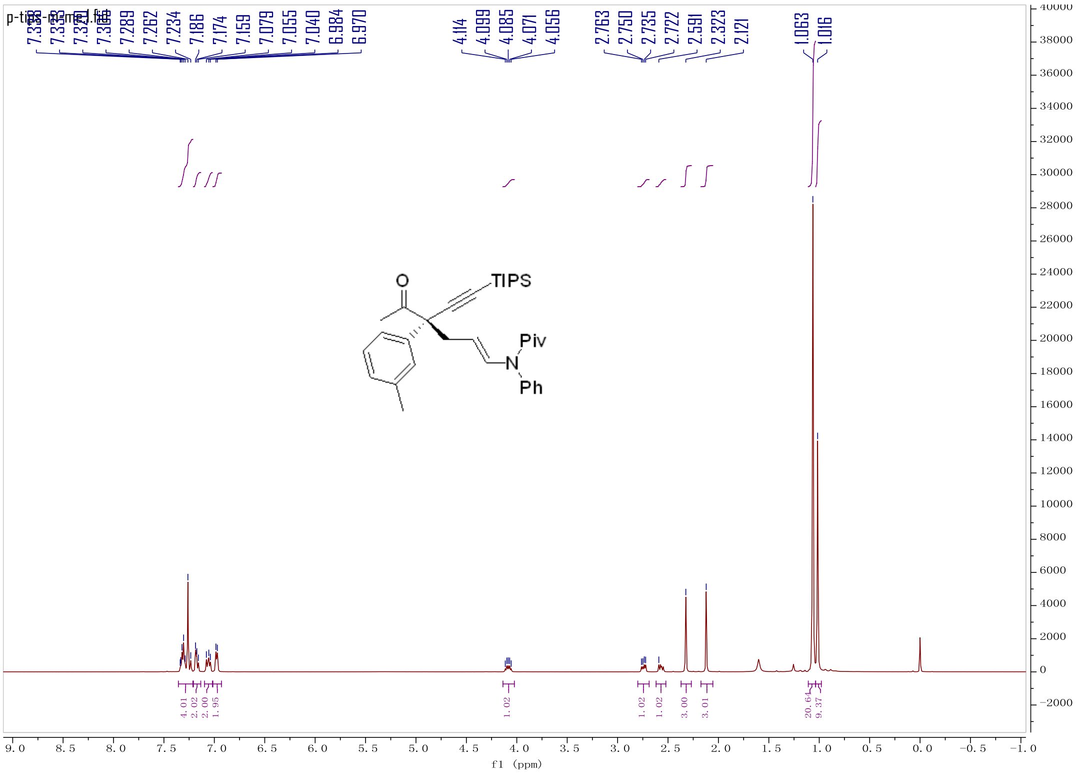

Supplementary Fig. 154 ^{1}H NMR spectrum of **3e**

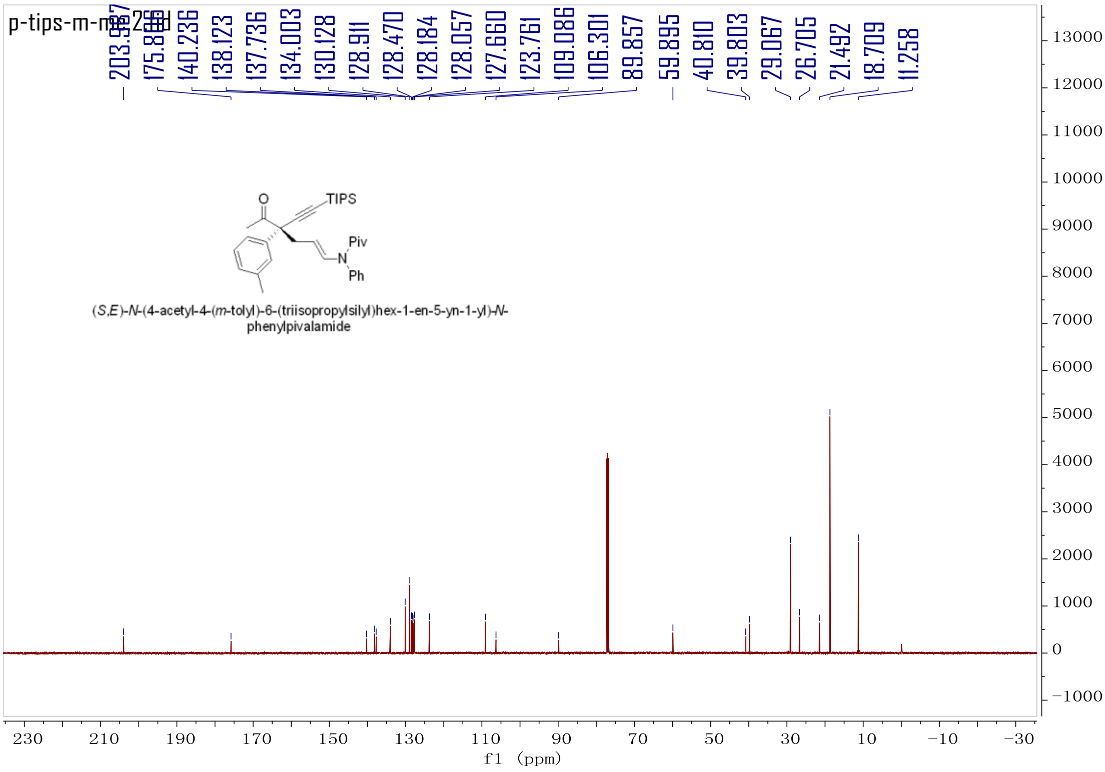

Supplementary Fig. 155 ^{13}C NMR spectrum of **3e**

(*S*,*E*)-N-(4-acetyl-4-(3-chlorophenyl)-6-(triisopropylsilyl)hex-1-en-5-yn-1-yl)-N-phenylpivalamide (**3f**)

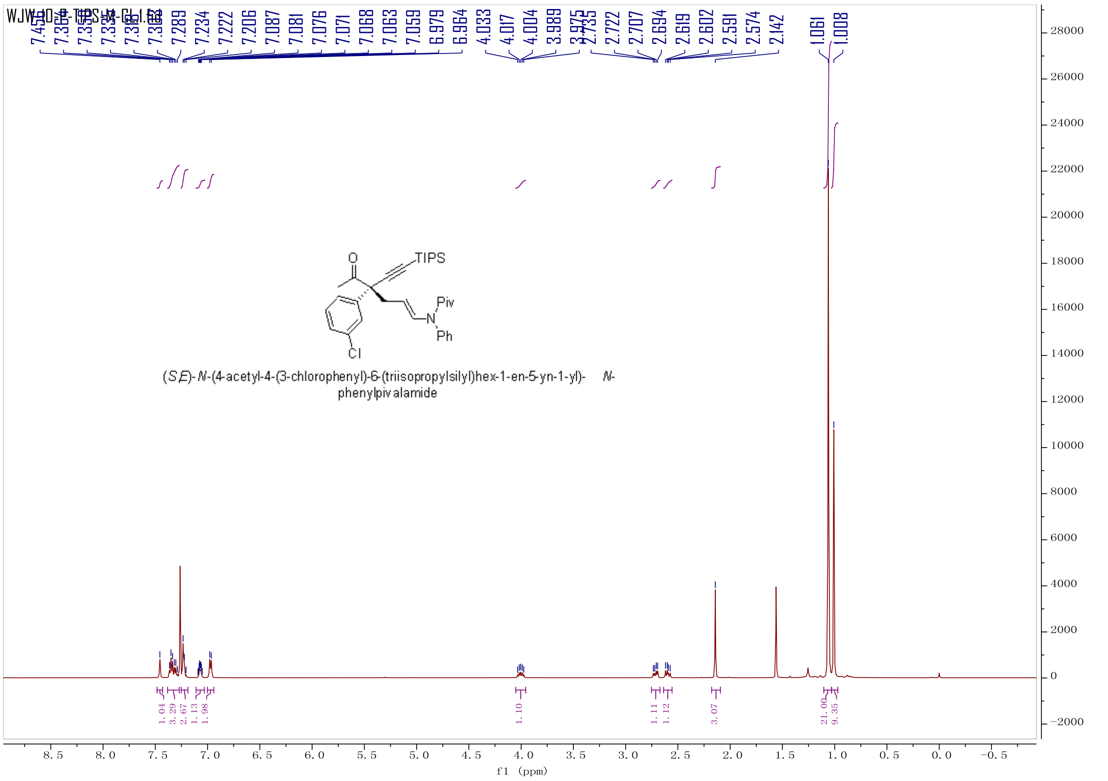

Supplementary Fig. 156 ^{1}H NMR spectrum of **3f**

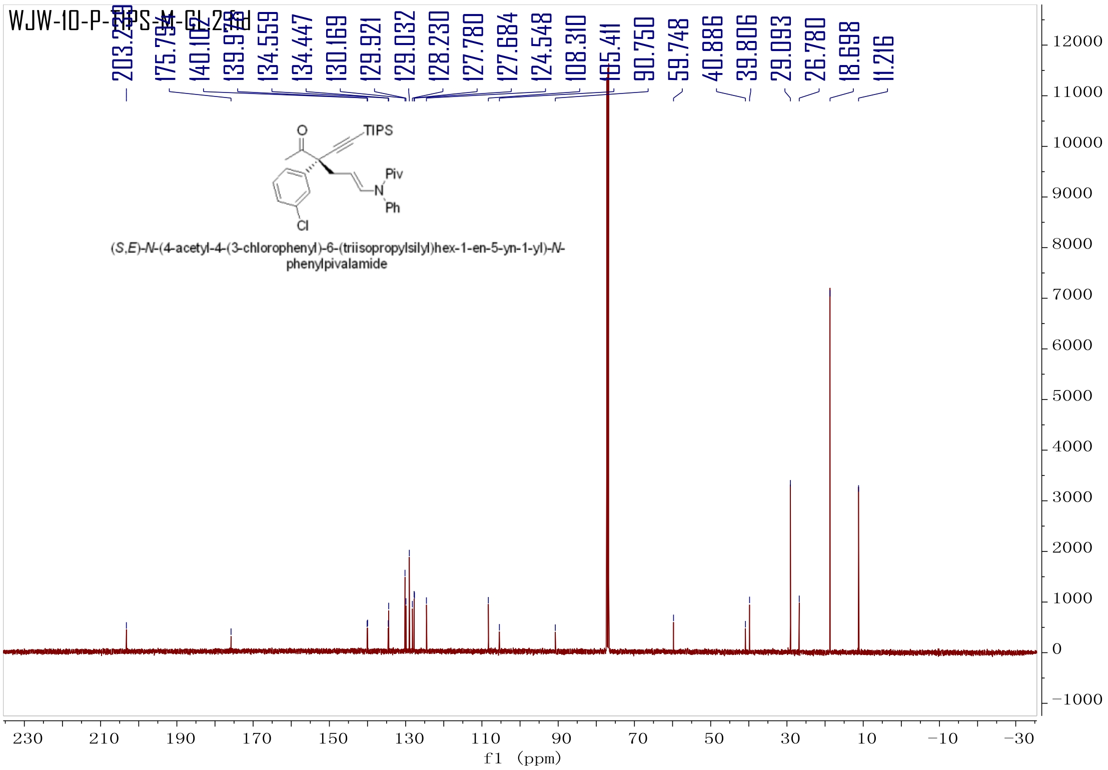

Supplementary Fig. 157 ^{13}C NMR spectrum of **3f**

(*S*,*E*)-N-(4-acetyl-4-(3-fluorophenyl)-6-(triisopropylsilyl)hex-1-en-5-yn-1-yl)-N-phenylpivalamide (**3g**)

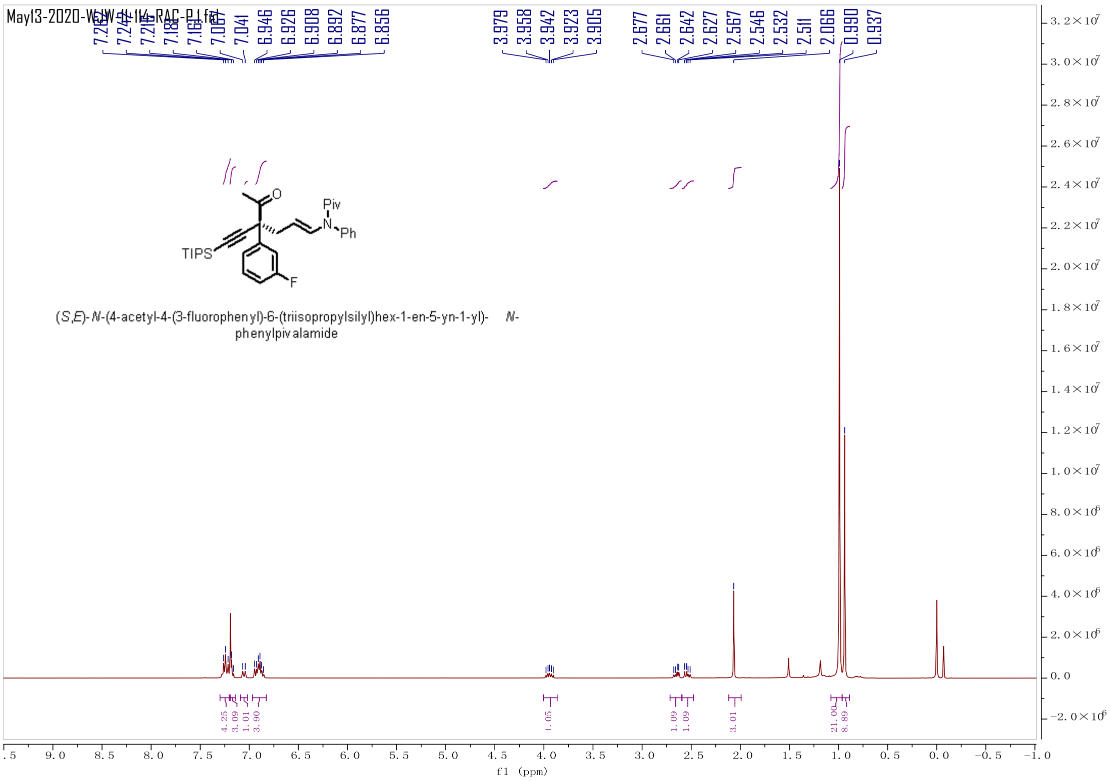

Supplementary Fig. 158 ^{1}H NMR spectrum of **3g**

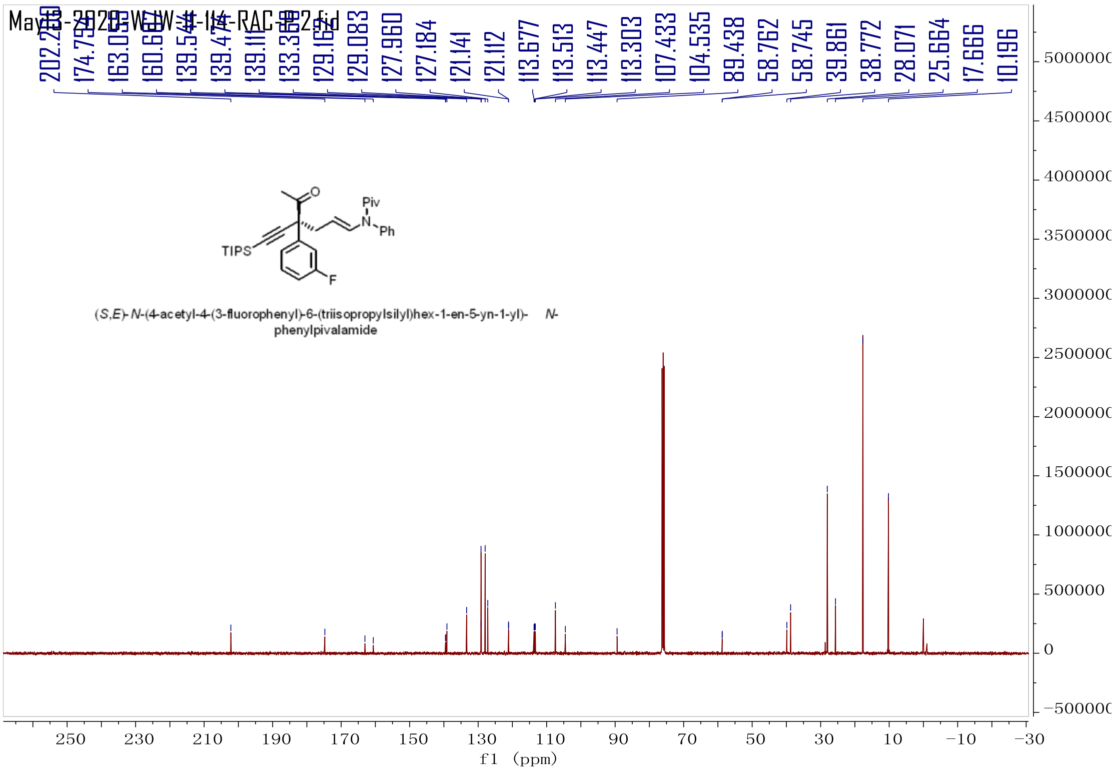

Supplementary Fig. 159 ^{13}C NMR spectrum of **3g**

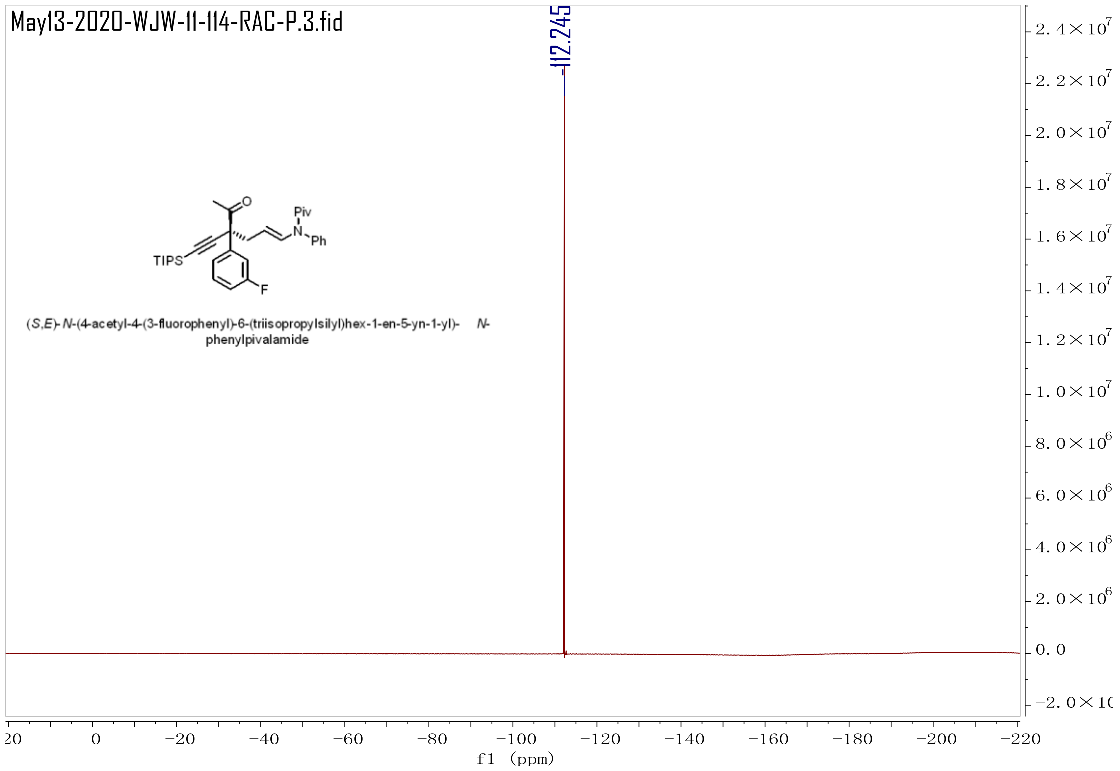

Supplementary Fig. 160 ^{19}F NMR spectrum of **3g**

(*S*,*E*)-N-(4-acetyl-4-(3-methoxyphenyl)-6-(triisopropylsilyl)hex-1-en-5-yn-1-yl)-N-phenylpivalamide (**3h**)

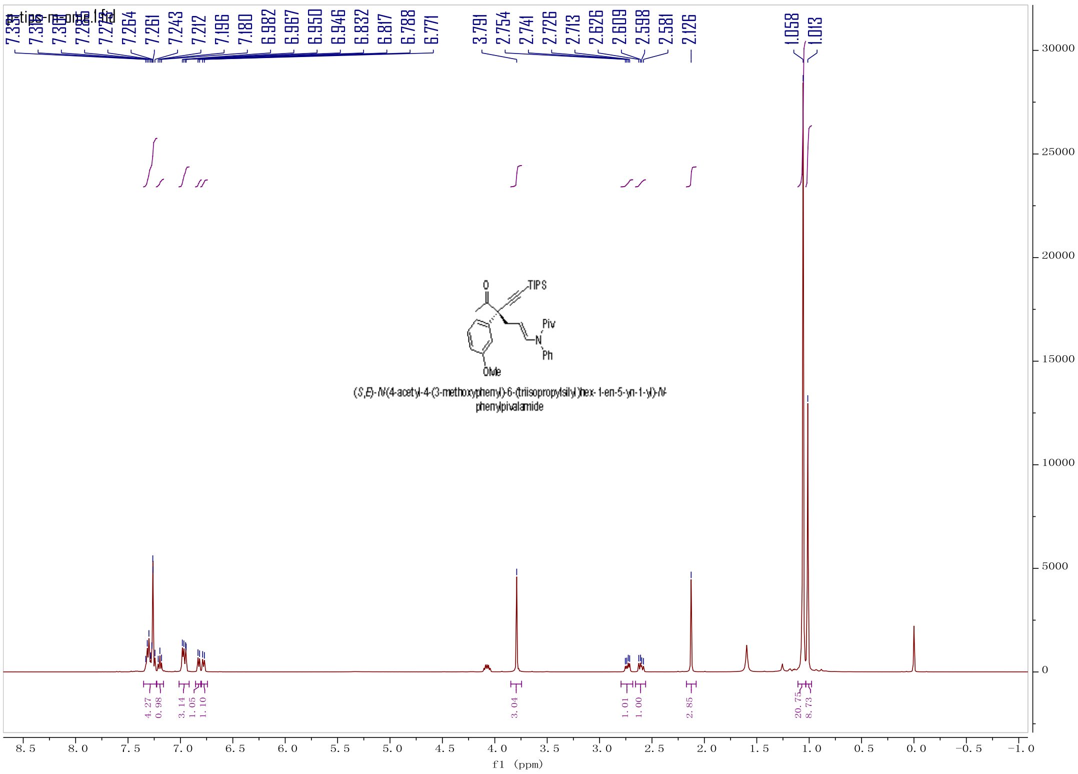

Supplementary Fig. 161 ^{1}H NMR spectrum of **3h**

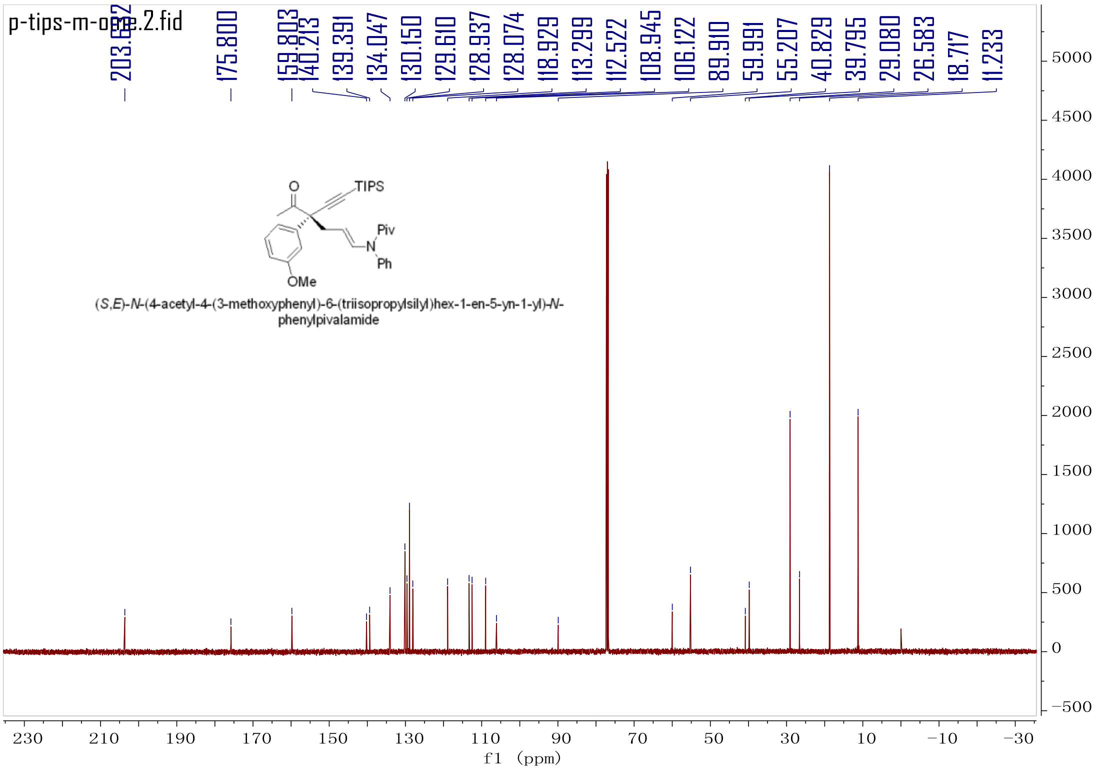

Supplementary Fig. 162 ^{13}C NMR spectrum of **3h**

(*S*,*E*)-N-(4-acetyl-4-(naphthalen-2-yl)-6-(triisopropylsilyl)hex-1-en-5-yn-1-yl)-N-phenylpivalamide (**3i**)

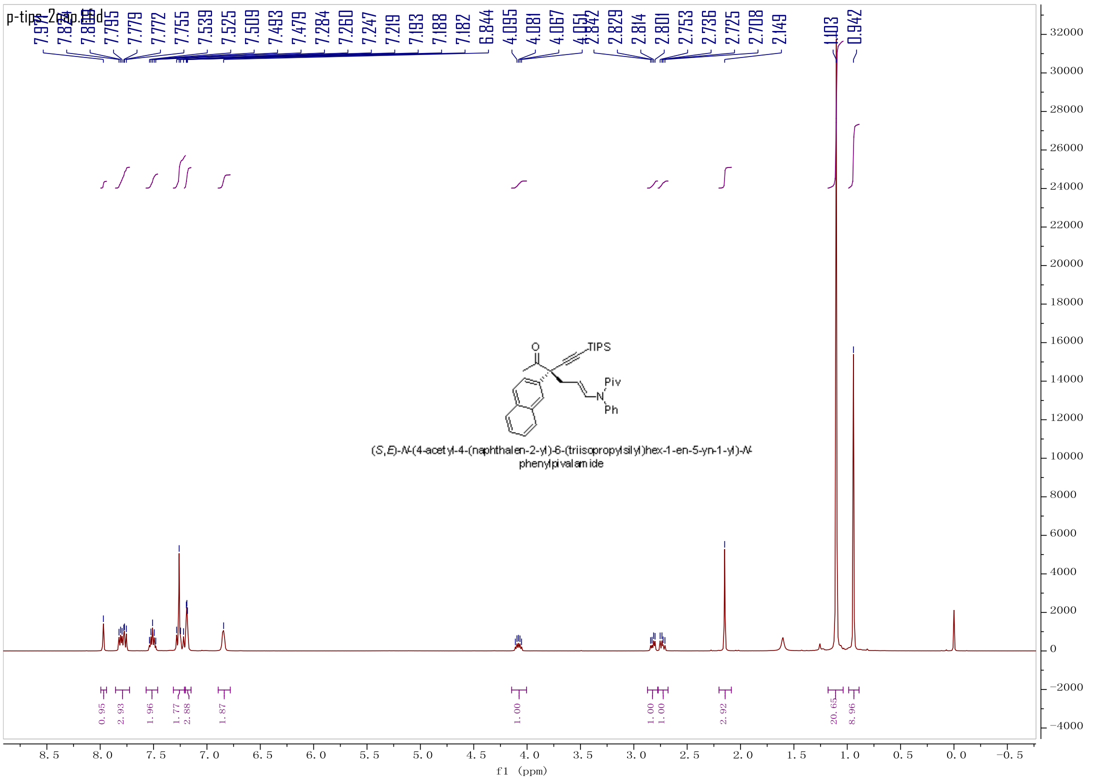

Supplementary Fig. 163 ^{1}H NMR spectrum of **3i**

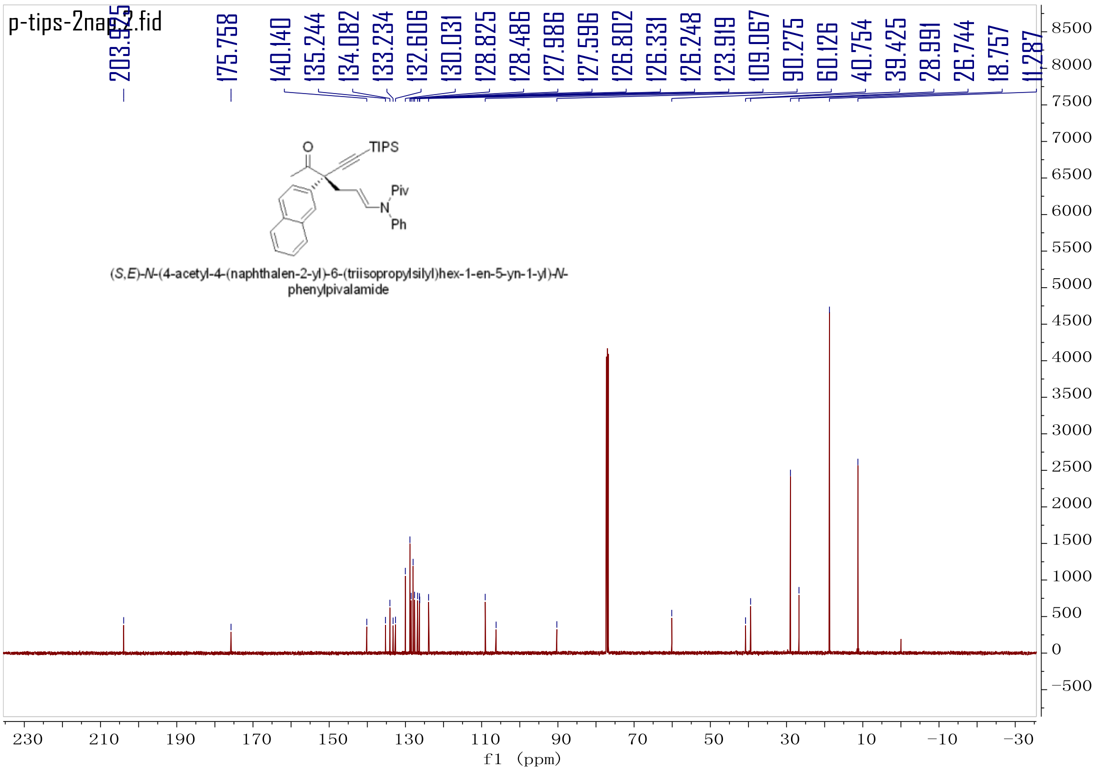

Supplementary Fig. 164 ^{13}C NMR spectrum of **3i**

(*R*,*E*)-N-(4-acetyl-4-methyl-6-(triisopropylsilyl)hex-1-en-5-yn-1-yl)-N-phenylpivalamide (**3j**)

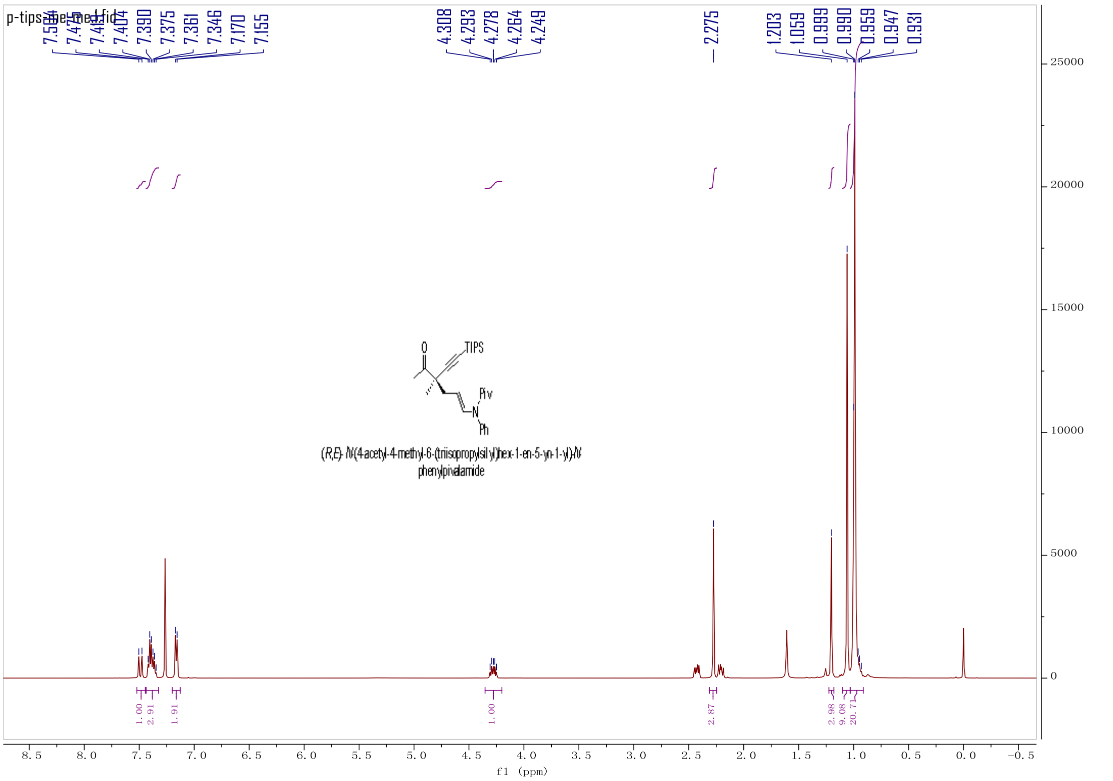

Supplementary Fig. 165 ^{1}H NMR spectrum of **3j**

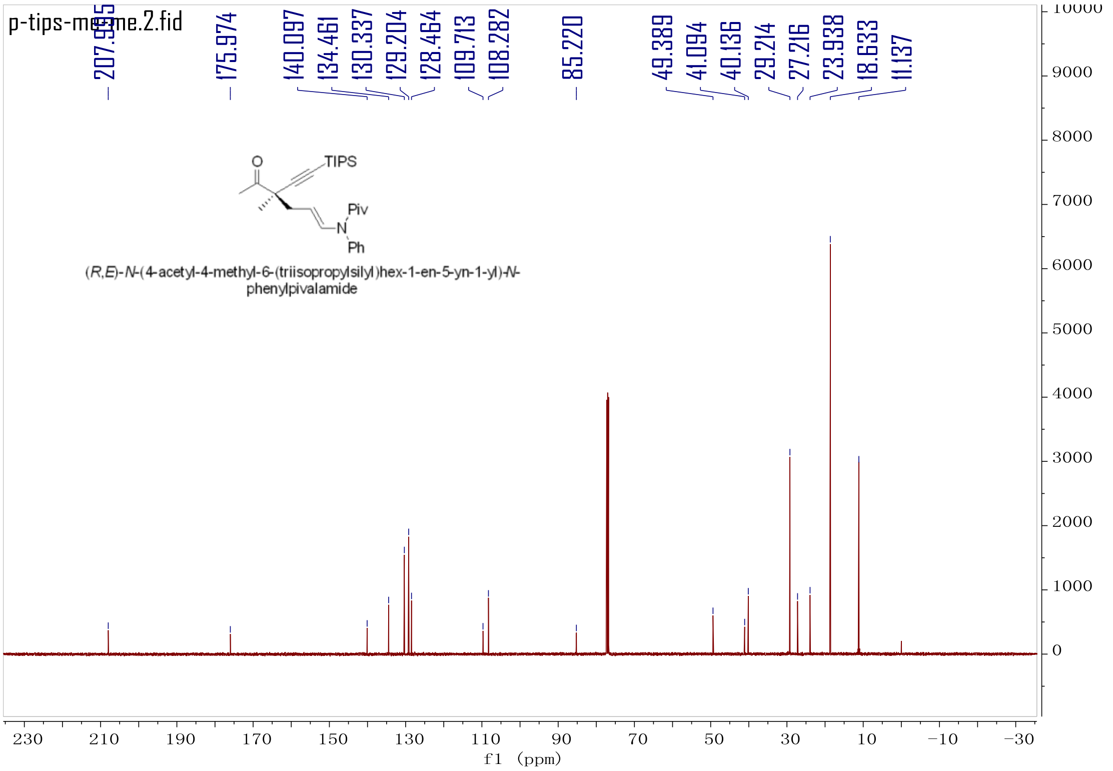

Supplementary Fig. 166 ^{13}C NMR spectrum of **3j**

(*S*,*E*)-N-(4-acetyl-4-phenyl-6-(p-tolyl)hex-1-en-5-yn-1-yl)-N-phenylpivalamide (**3k**)

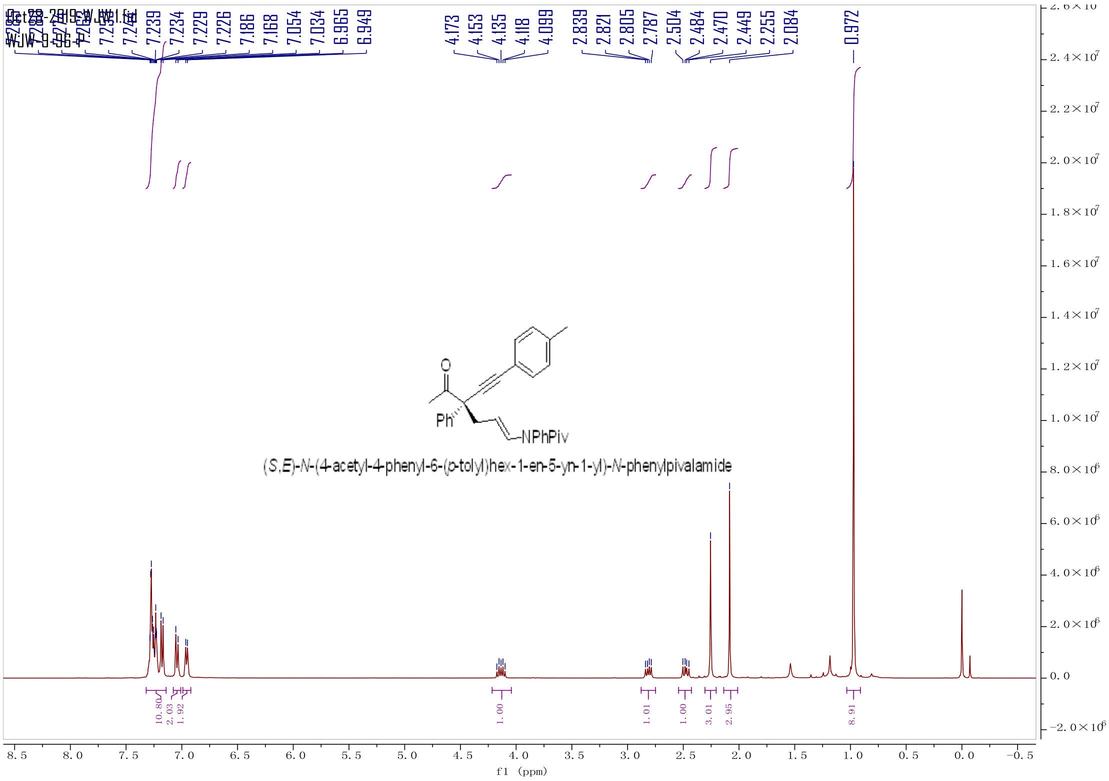

Supplementary Fig. 167 ^{1}H NMR spectrum of **3k**

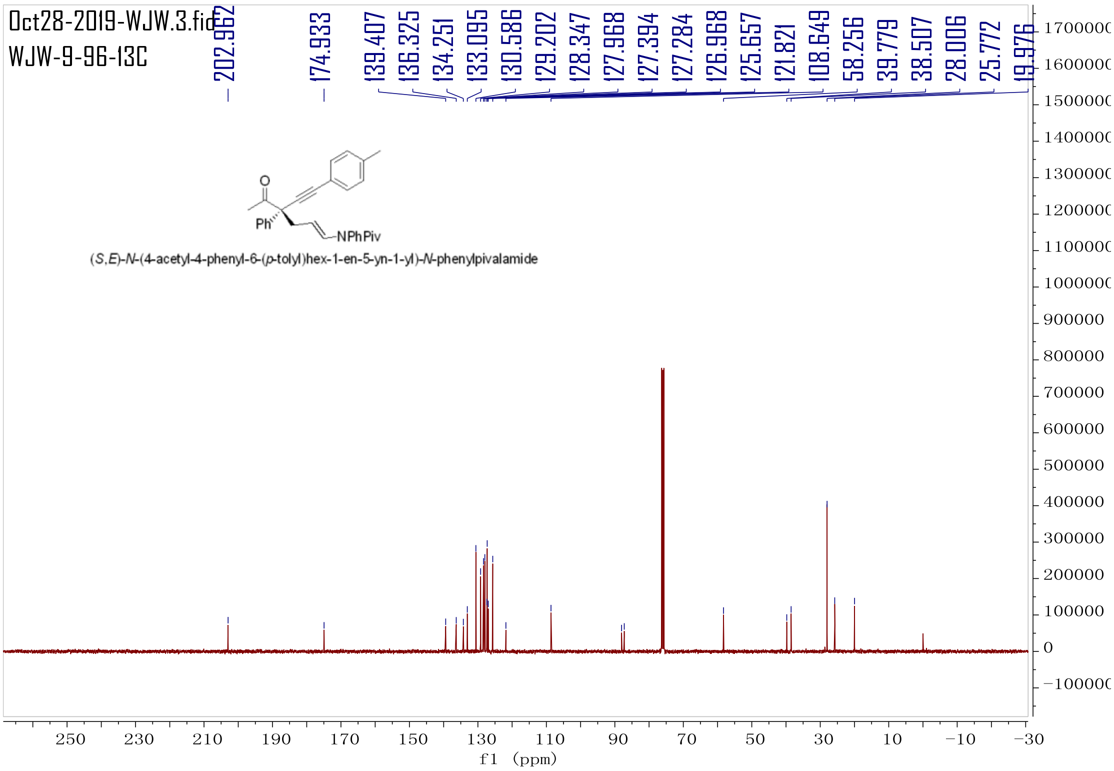

Supplementary Fig. 168 ^{13}C NMR spectrum of **3k**

(*R*,*E*)-N-(4-acetyl-4-ethyl-6-phenylhex-1-en-5-yn-1-yl)-N-phenylpivalamide (**3l**)

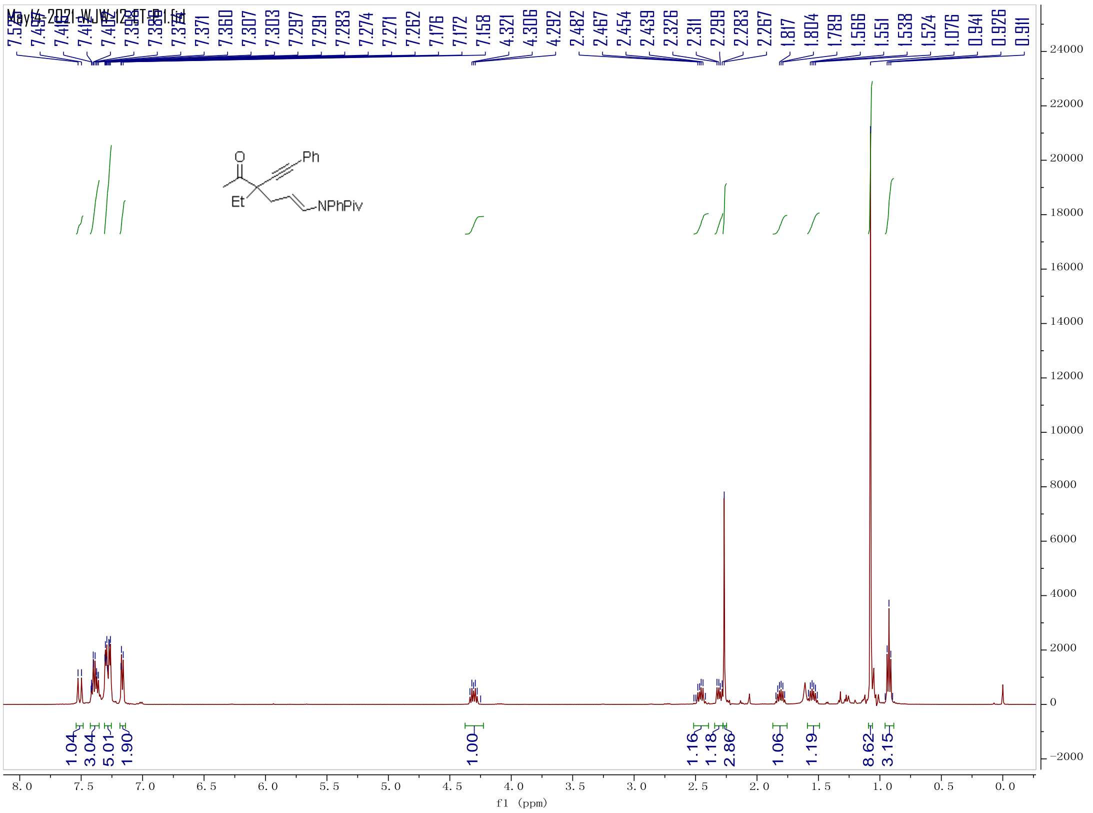

Supplementary Fig. 169 ^{1}H NMR spectrum of **3l**

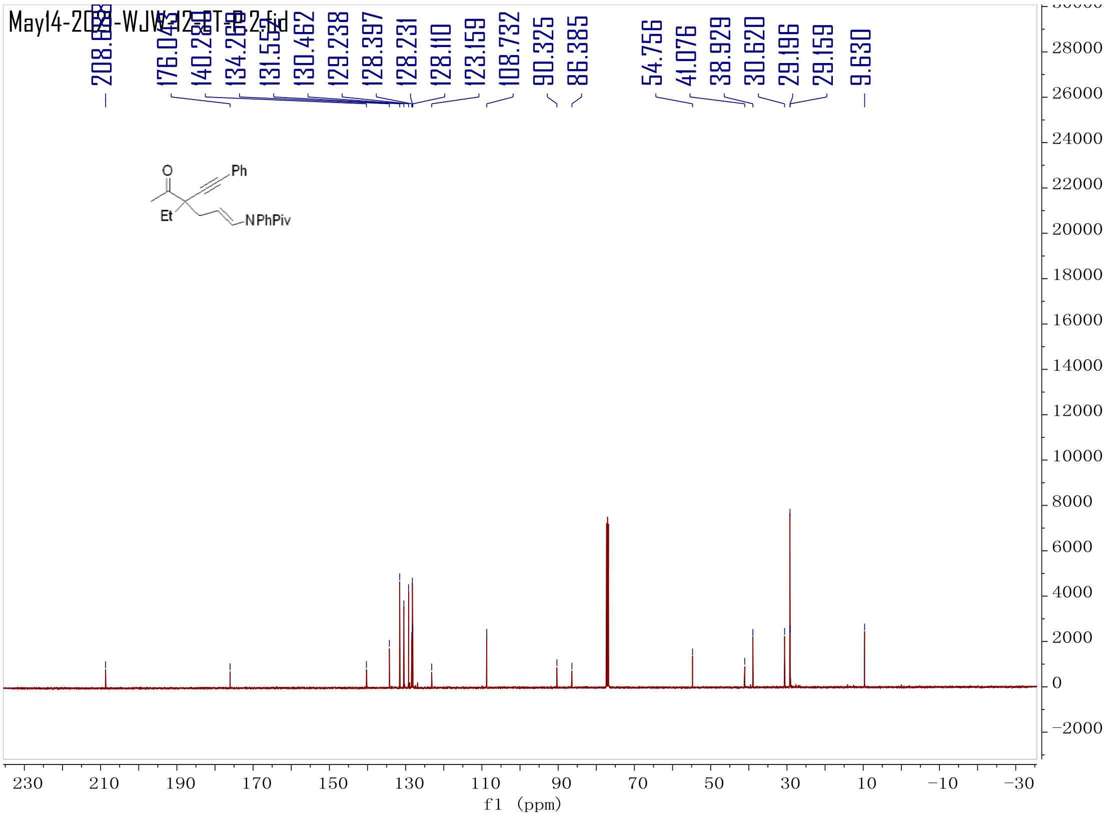

Supplementary Fig. 170 ^{13}C NMR spectrum of **3l**

(*S*,*E*)-N-(4-acetyl-4-phenyldec-1-en-5-yn-1-yl)-N-phenylpivalamide (**3m**)

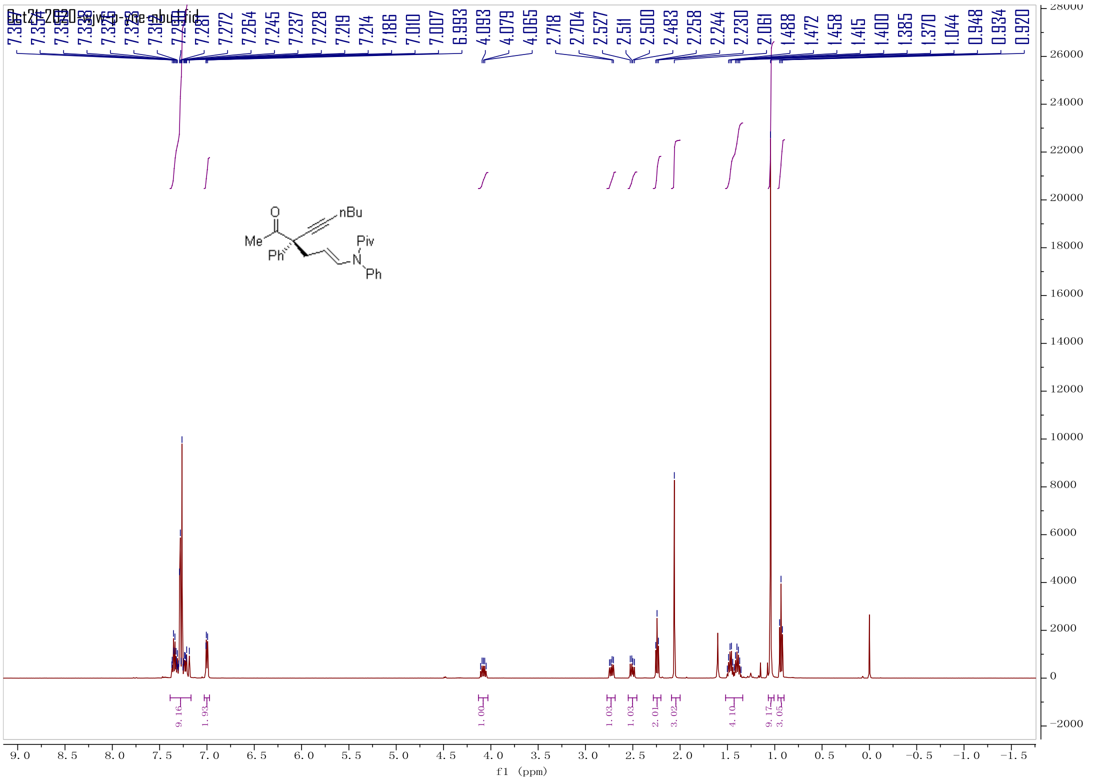

Supplementary Fig. 171 ^{1}H NMR spectrum of **3m**

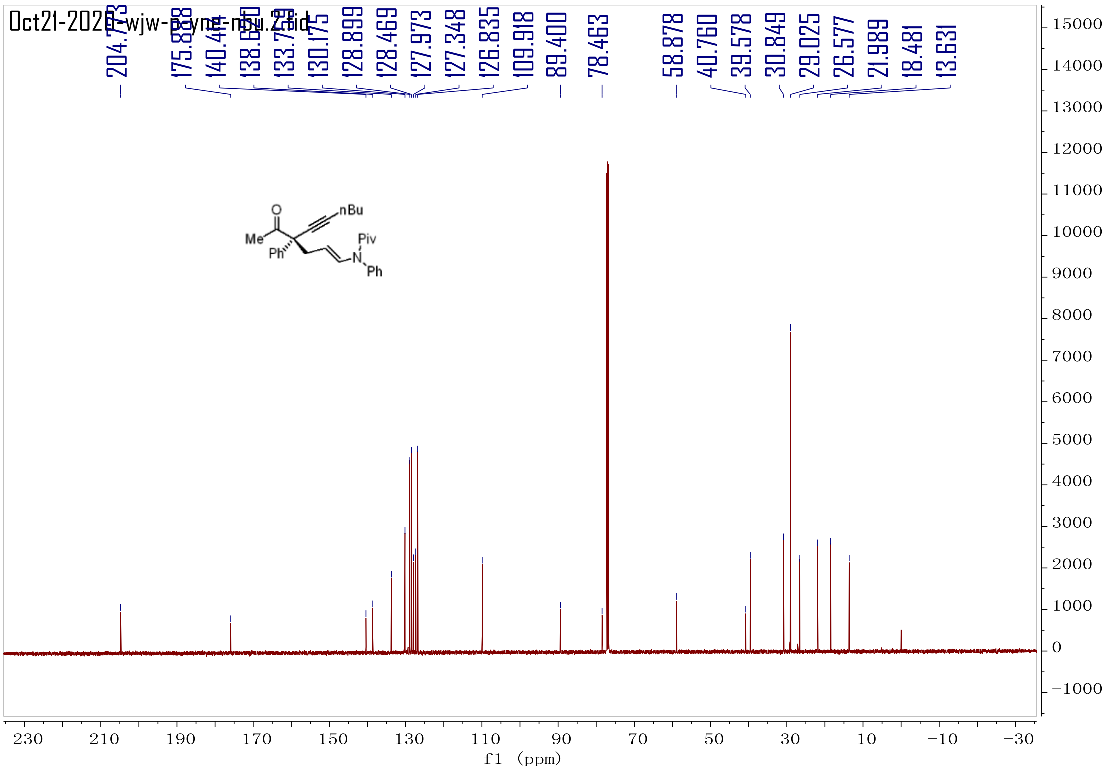

Supplementary Fig. 172 ^{13}C NMR spectrum of **3m**

(*R*,*E*)-N-(4-acetyl-4-phenylhex-1-en-5-yn-1-yl)-N-phenylpivalamide (**3n**)

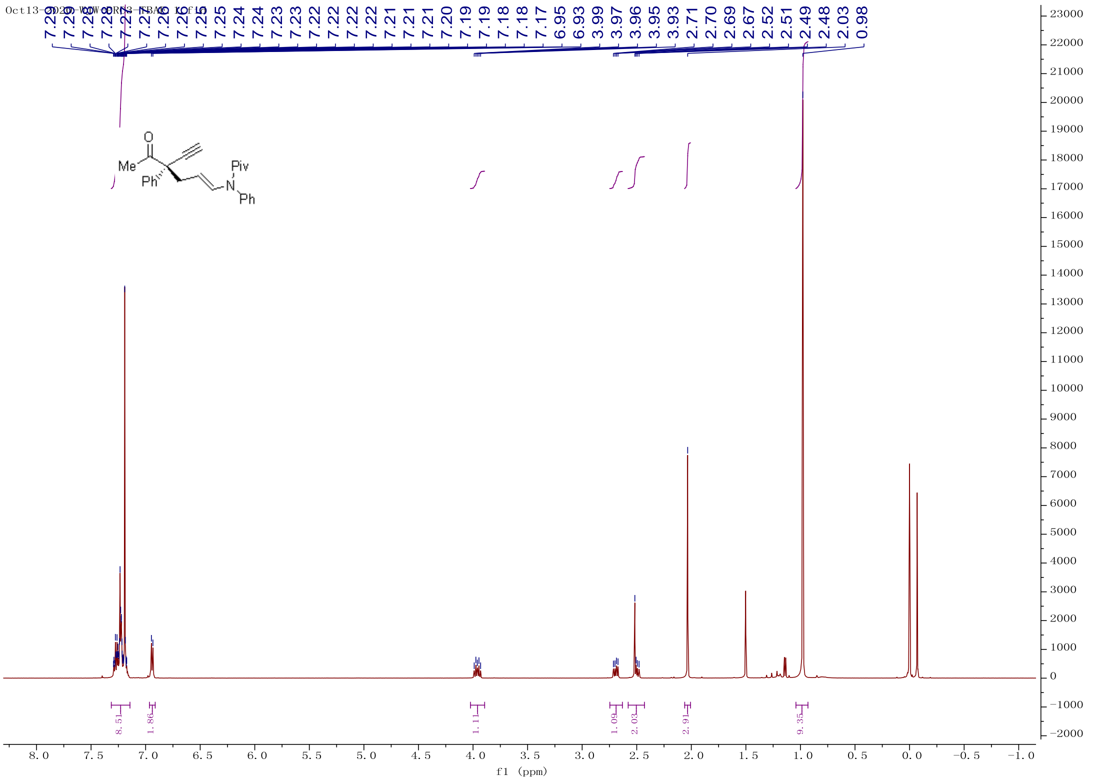

Supplementary Fig. 173 ^{1}H NMR spectrum of **3n**

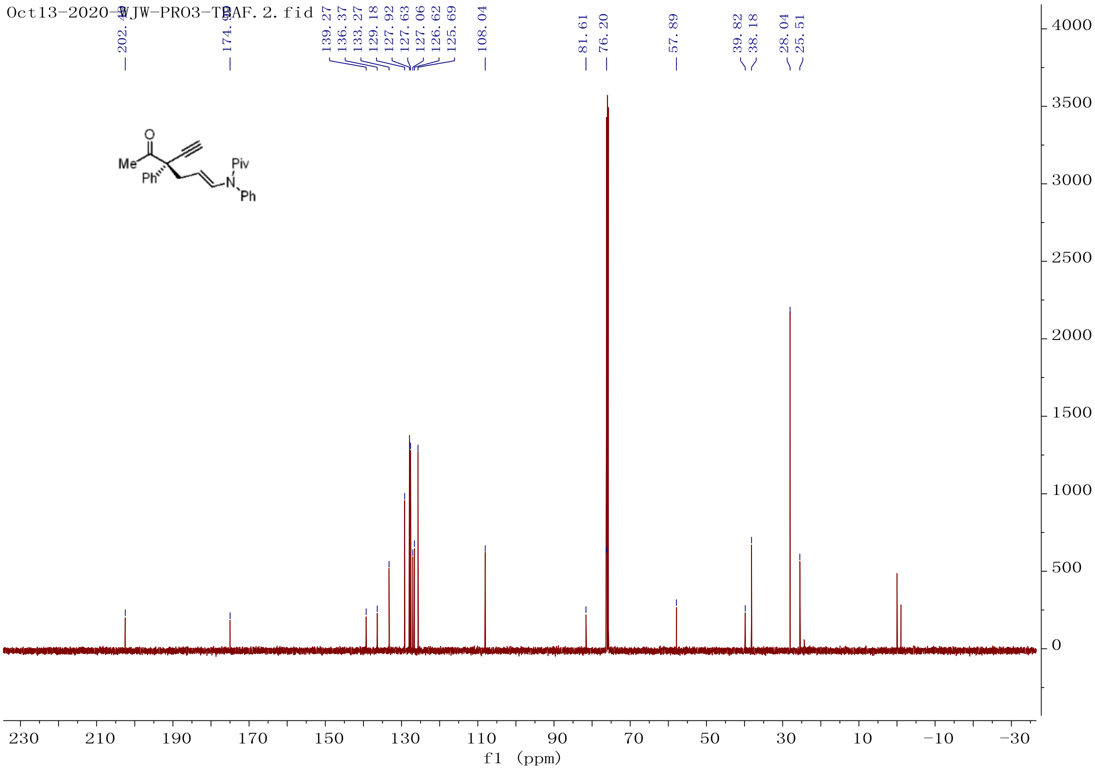

Supplementary Fig. 174 ^{13}C NMR spectrum of **3n**

(*S*,*E*)-N-(5-oxo-4-phenyl-4-((triisopropylsilyl)ethynyl)hept-1-en-1-yl)-N-phenylpivalamide (**3o**)

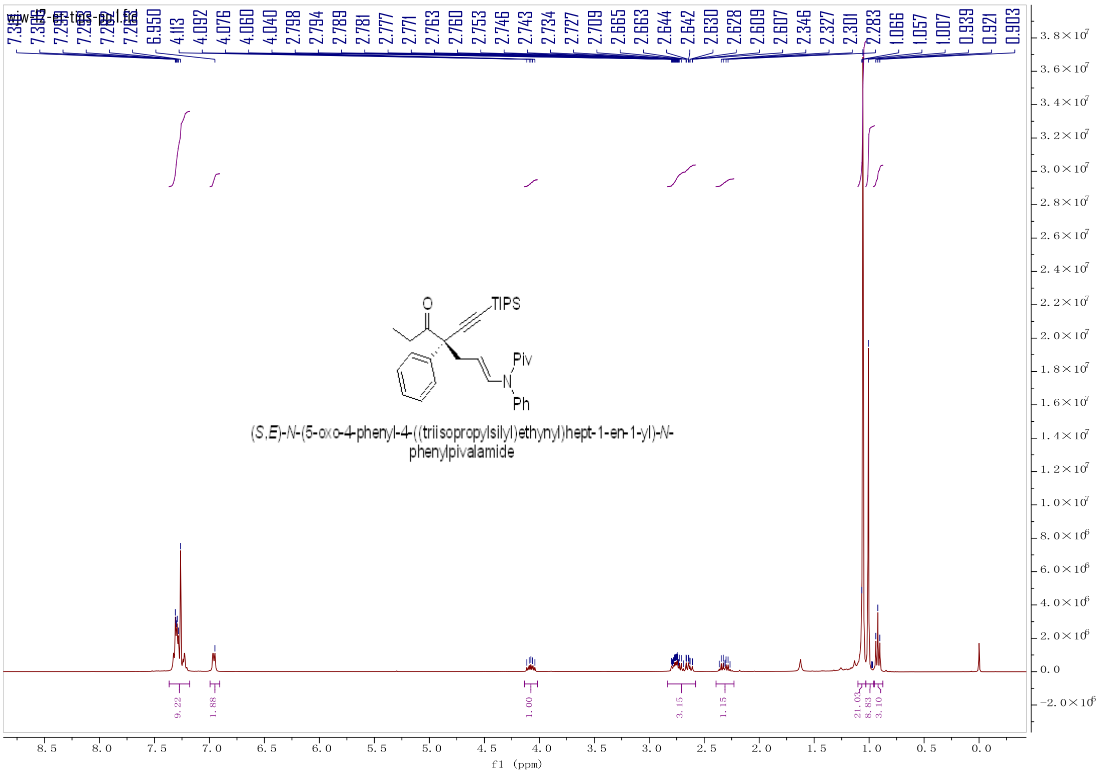

Supplementary Fig. 175 ^{1}H NMR spectrum of **3o**

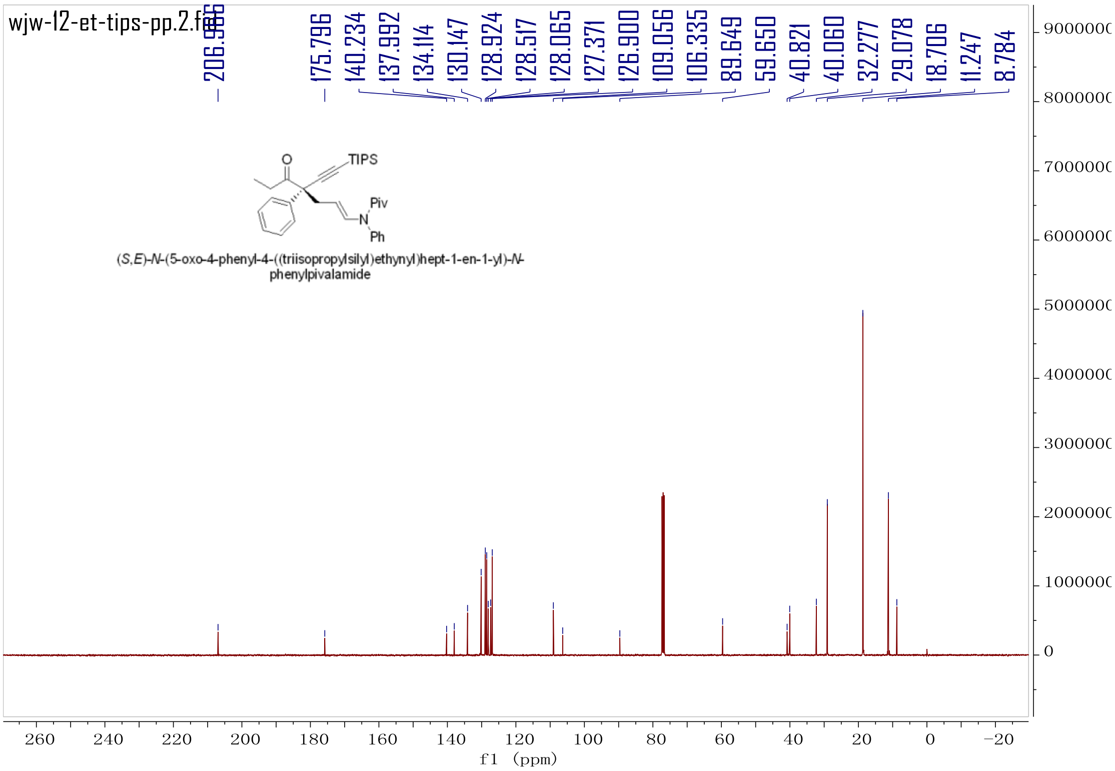

Supplementary Fig. 176 ^{13}C NMR spectrum of **3o**

(*S*,*E*)-N-(5-oxo-4-phenyl-4-((triisopropylsilyl)ethynyl)non-1-en-1-yl)-N-phenylpivalamide (**3p**)

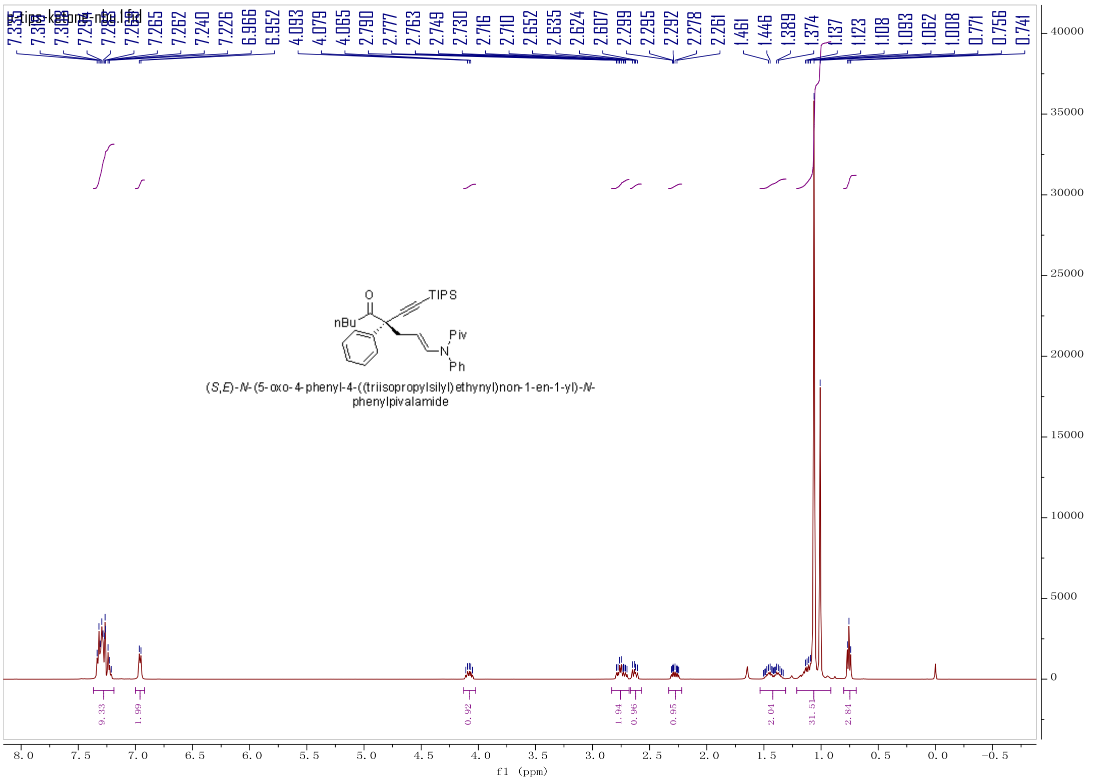

Supplementary Fig. 177 ^{1}H NMR spectrum of **3p**

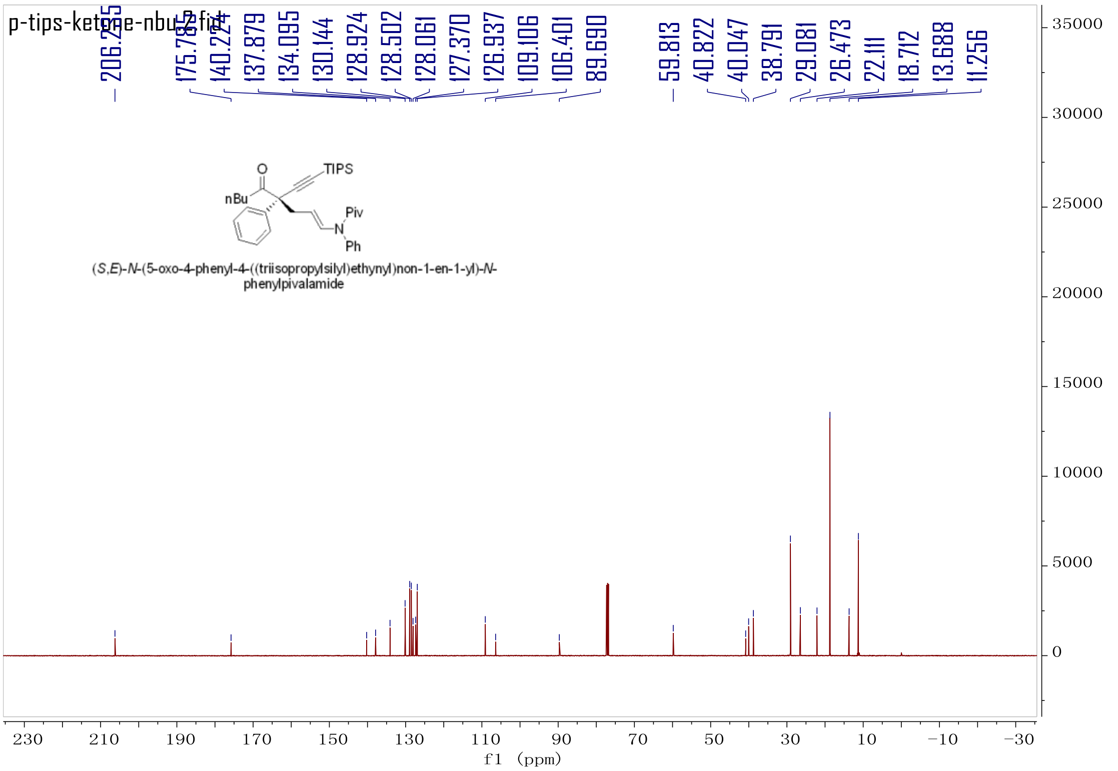

Supplementary Fig. 178 ^{13}C NMR spectrum of **3p**

(*S*,*E*)-N-(6-methyl-5-oxo-4-phenyl-4-((triisopropylsilyl)ethynyl)hept-1-en-1-yl)-N-phenylpivalamide (**3q**)

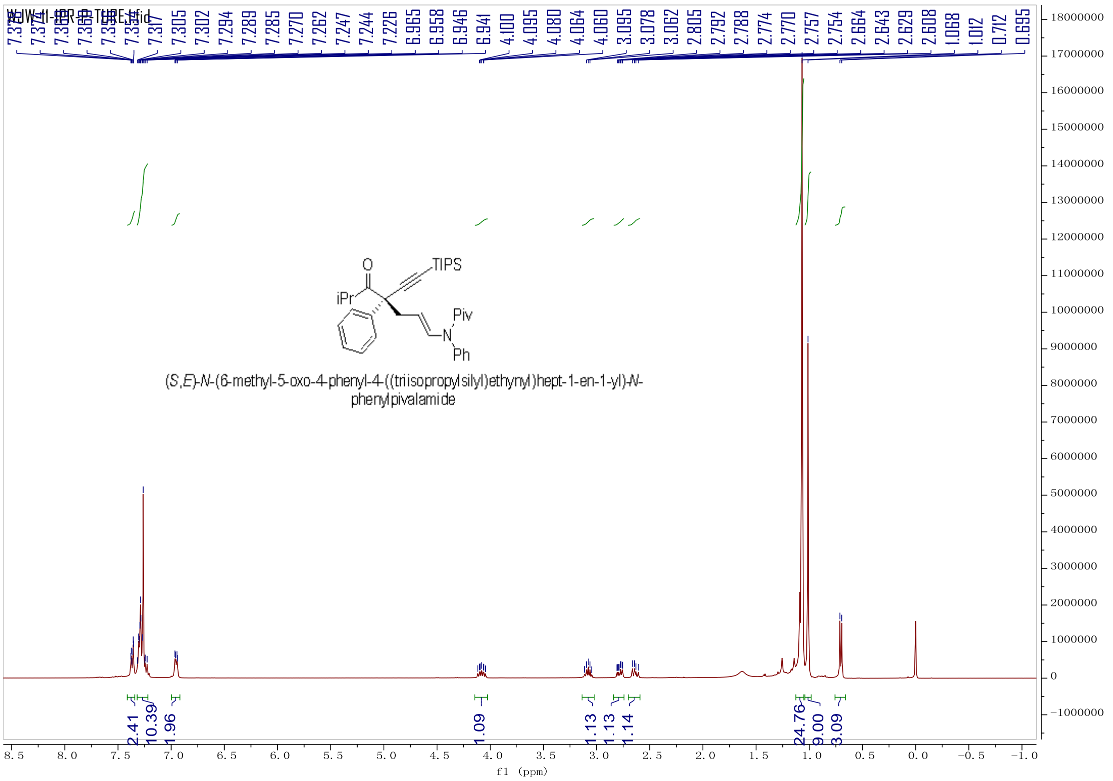

Supplementary Fig. 179 ^{1}H NMR spectrum of **3q**

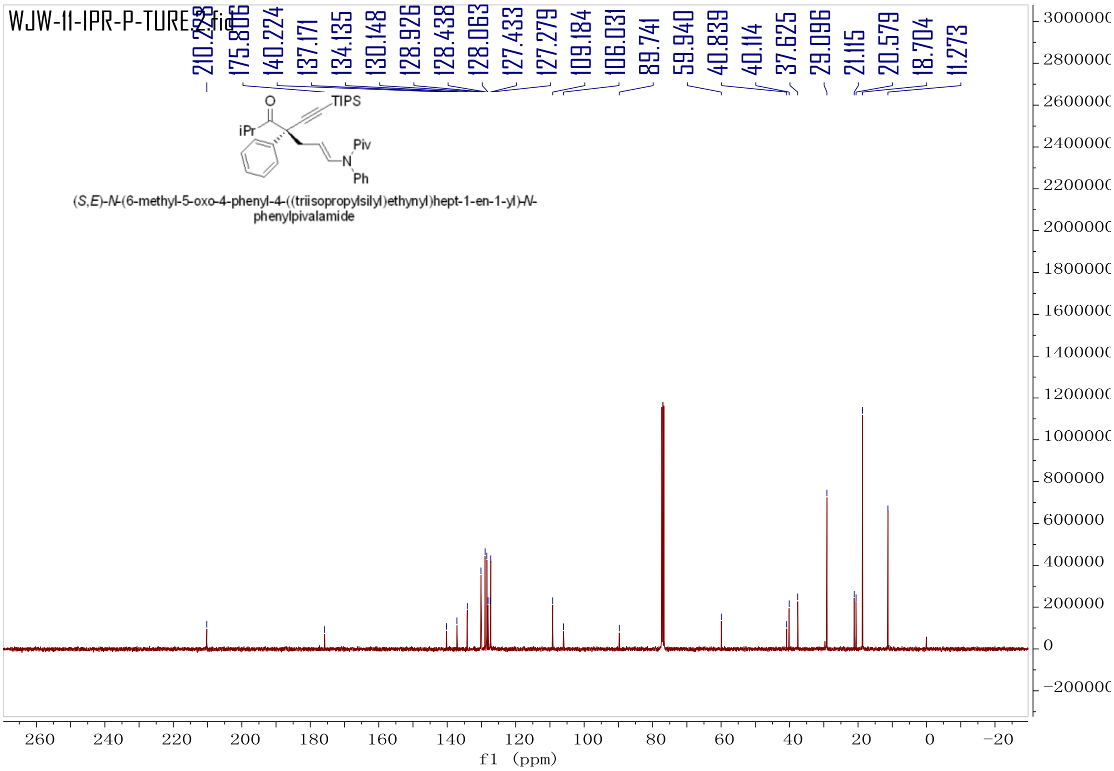

Supplementary Fig. 180 ^{13}C NMR spectrum of **3q**

(*S*,*E*)-N-(4-acetyl-4-phenyl-6-(triisopropylsilyl)hex-1-en-5-yn-1-yl)-N-(p-tolyl)pivalamide (**3r**)

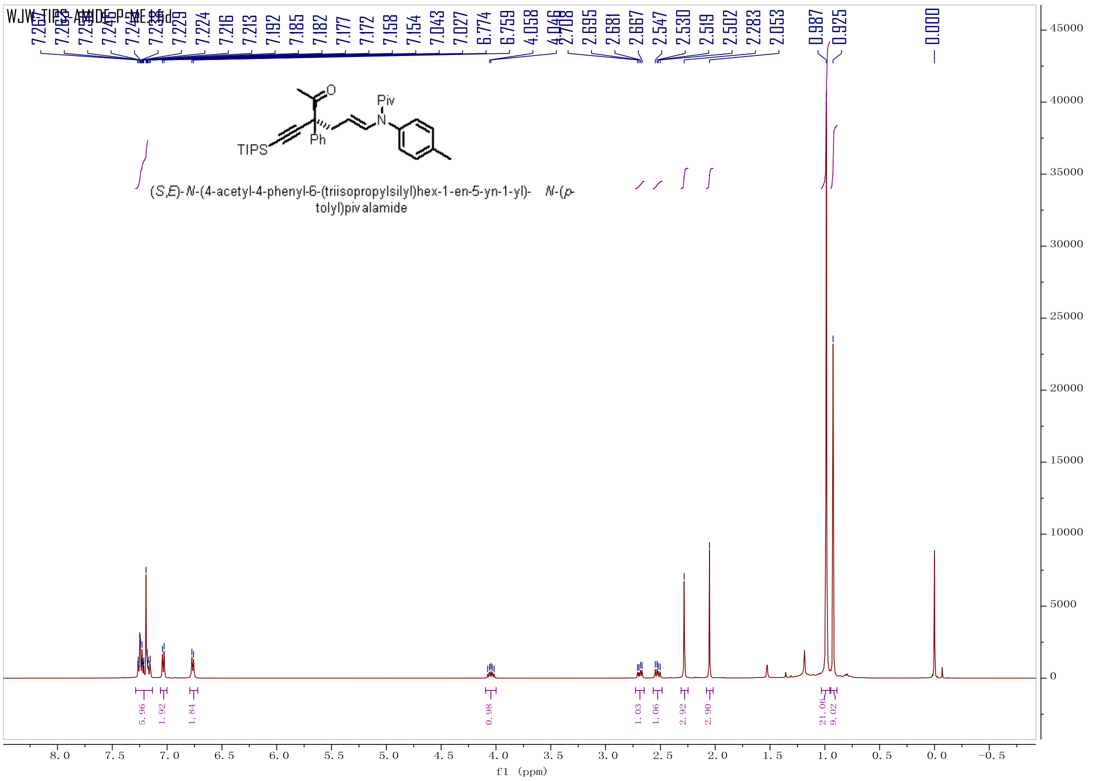

Supplementary Fig. 181 ^{1}H NMR spectrum of **3r**

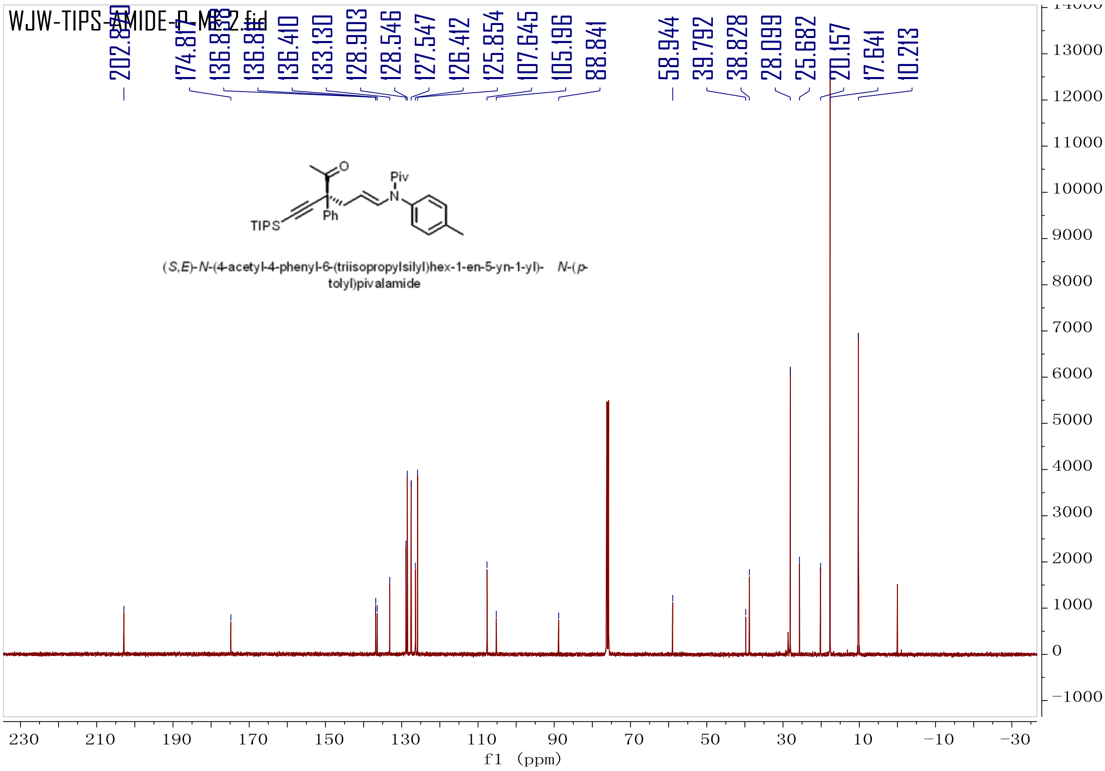

Supplementary Fig. 182 ^{13}C NMR spectrum of **3r**

(*S*,*E*)-N-(4-acetyl-4-phenyl-6-(triisopropylsilyl)hex-1-en-5-yn-1-yl)-N-(4-methoxyphenyl)pivalamide (**3s**)

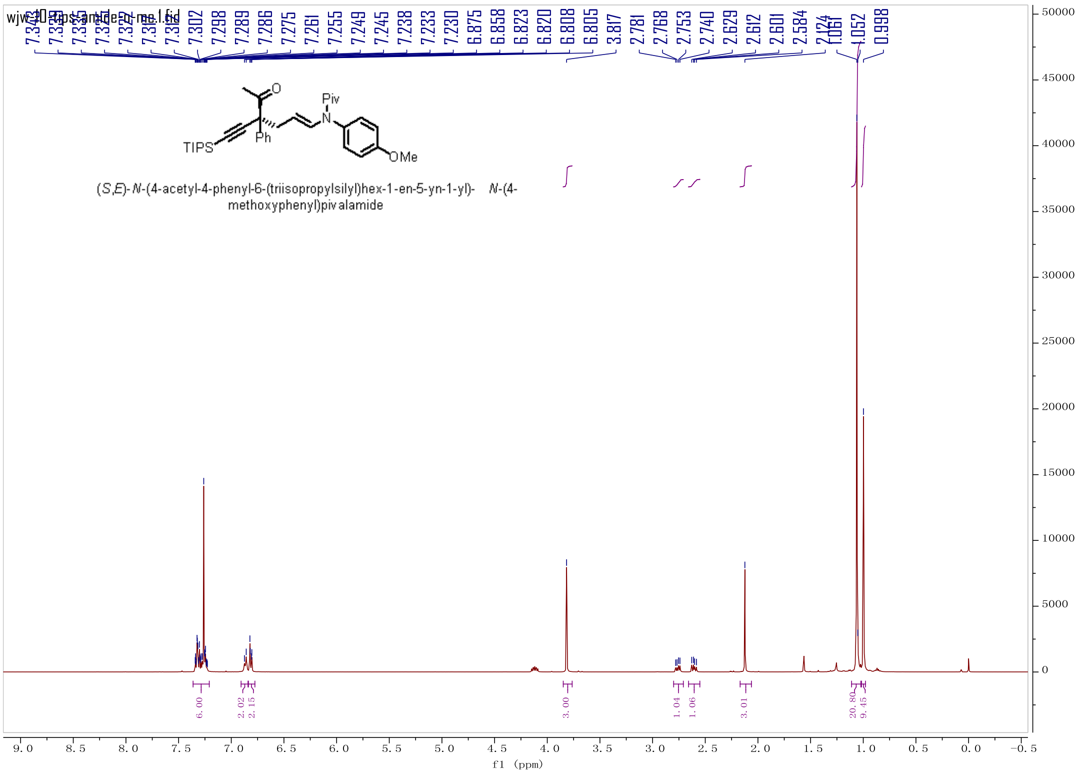

Supplementary Fig. 183 ^{1}H NMR spectrum of **3s**

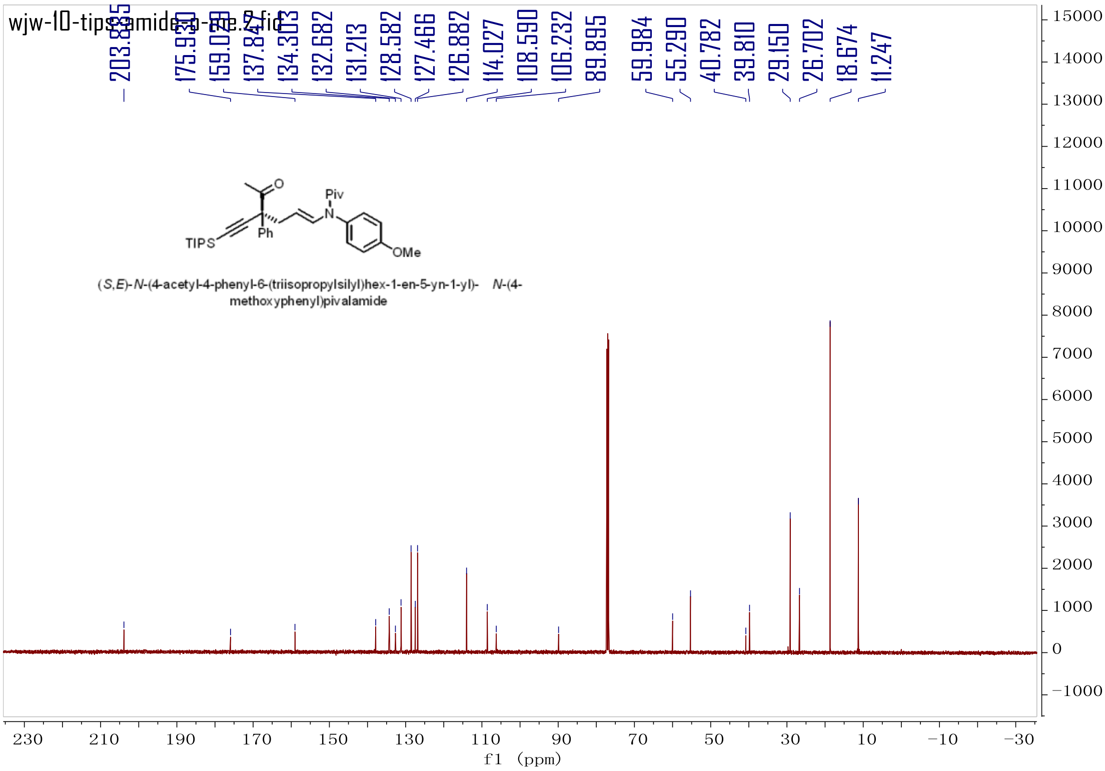

Supplementary Fig. 184 ^{13}C NMR spectrum of **3s**

(*S*,*E*)-N-(4-acetyl-4-phenyl-6-(triisopropylsilyl)hex-1-en-5-yn-1-yl)-N-(4-chlorophenyl)pivalamide (**3t**)

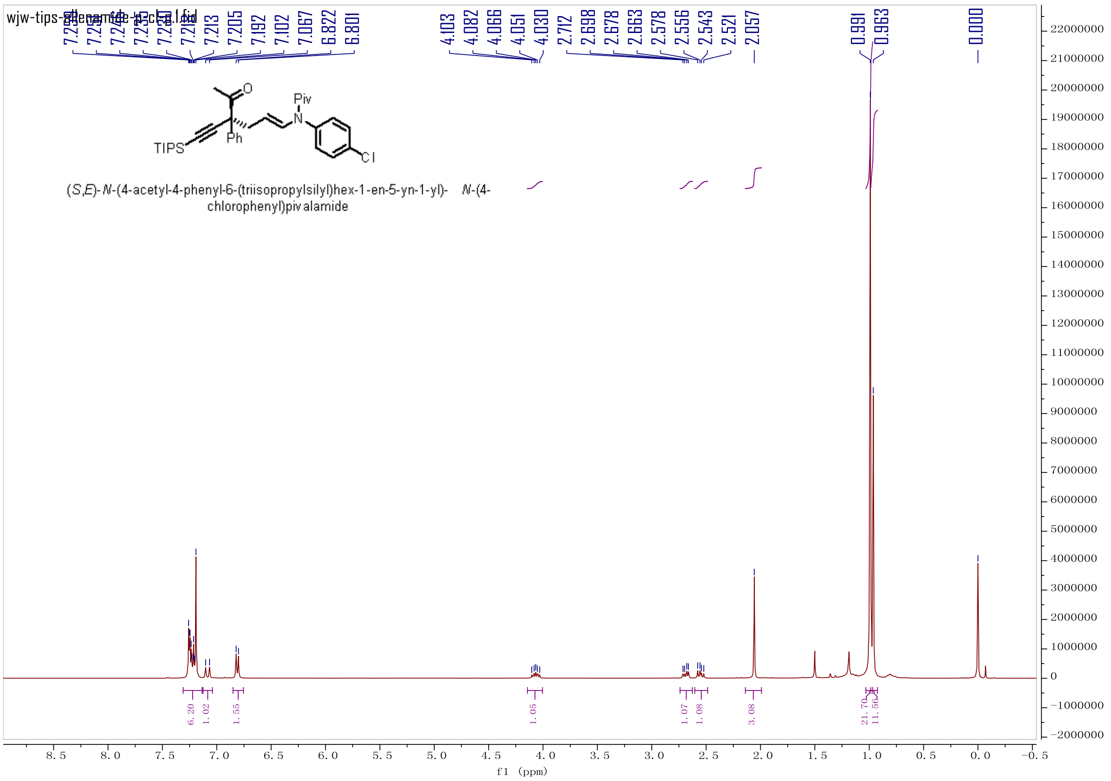

Supplementary Fig. 185 ^{1}H NMR spectrum of **3t**

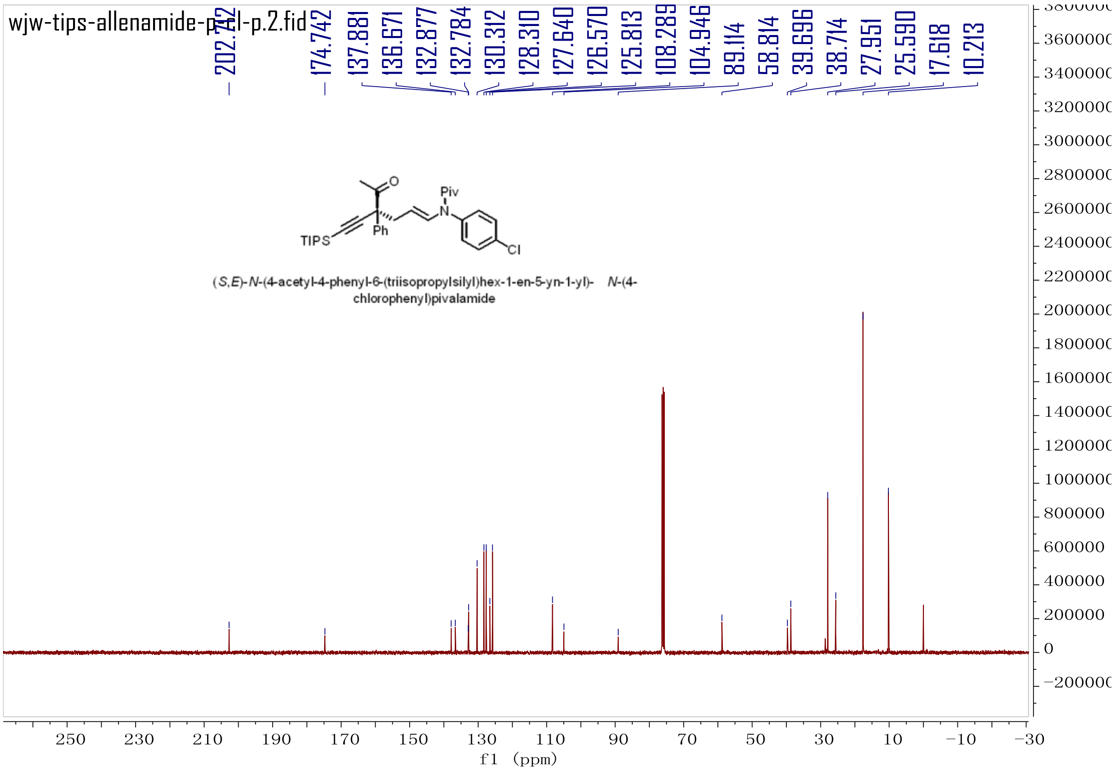

Supplementary Fig. 186 ^{13}C NMR spectrum of **3t**

(*S*,*E*)-N-(4-acetyl-4-phenyl-6-(triisopropylsilyl)hex-1-en-5-yn-1-yl)-N-(4-(trifluoromethyl)phenyl) pivalamide (**3u**)

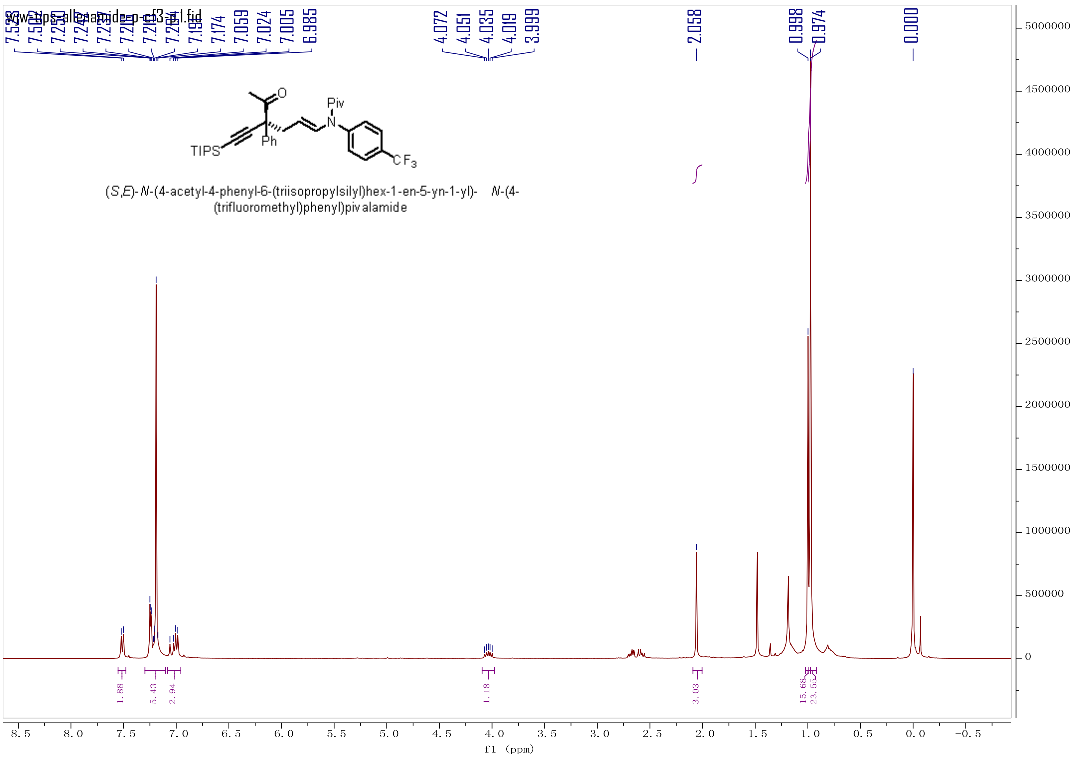

Supplementary Fig. 187 ^{1}H NMR spectrum of **3u**

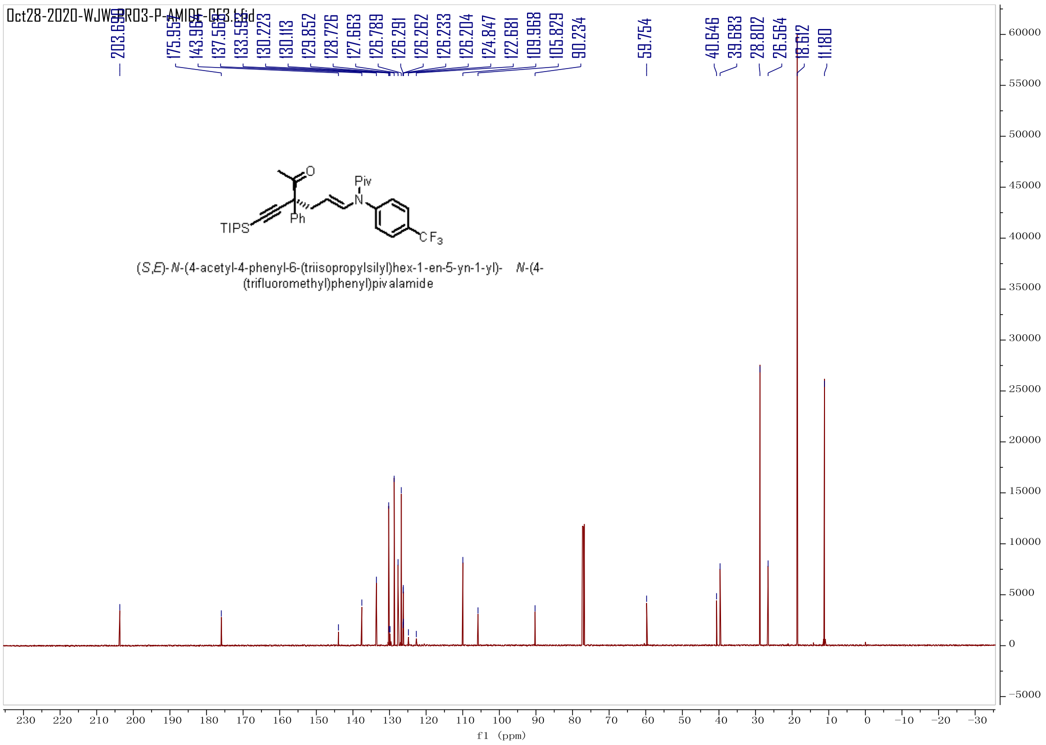

Supplementary Fig. 188 ^{13}C NMR spectrum of **3u**

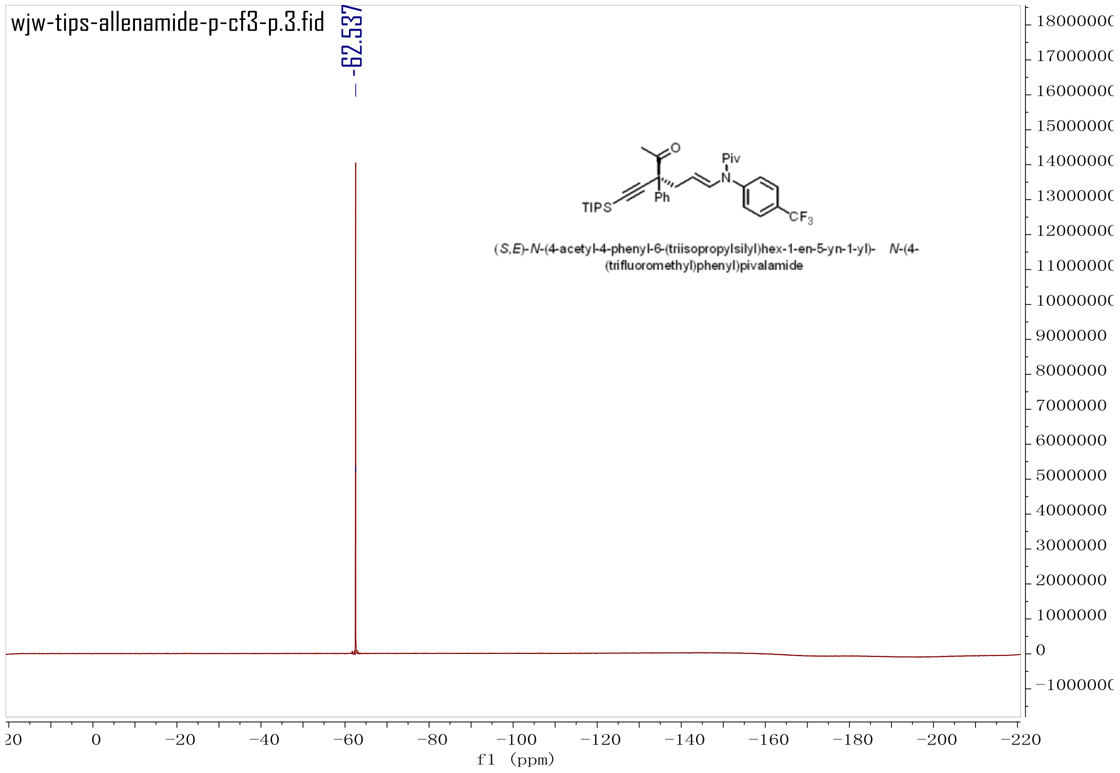

Supplementary Fig. 189 ^{19}F NMR spectrum of **3u**

(*S*,*E*)-N-(4-acetyl-4-phenyl-6-(triisopropylsilyl)hex-1-en-5-yn-1-yl)-N-(3-methoxyphenyl)pivalamide (**3v**)

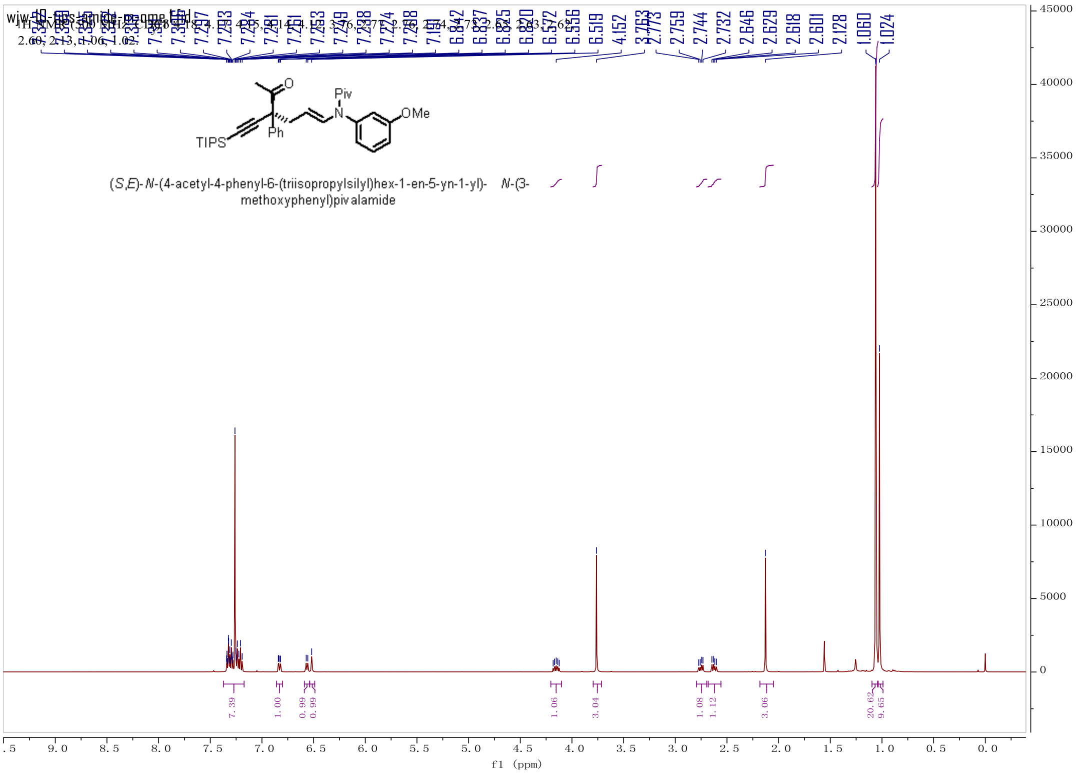

Supplementary Fig. 190 ^{1}H NMR spectrum of **3v**

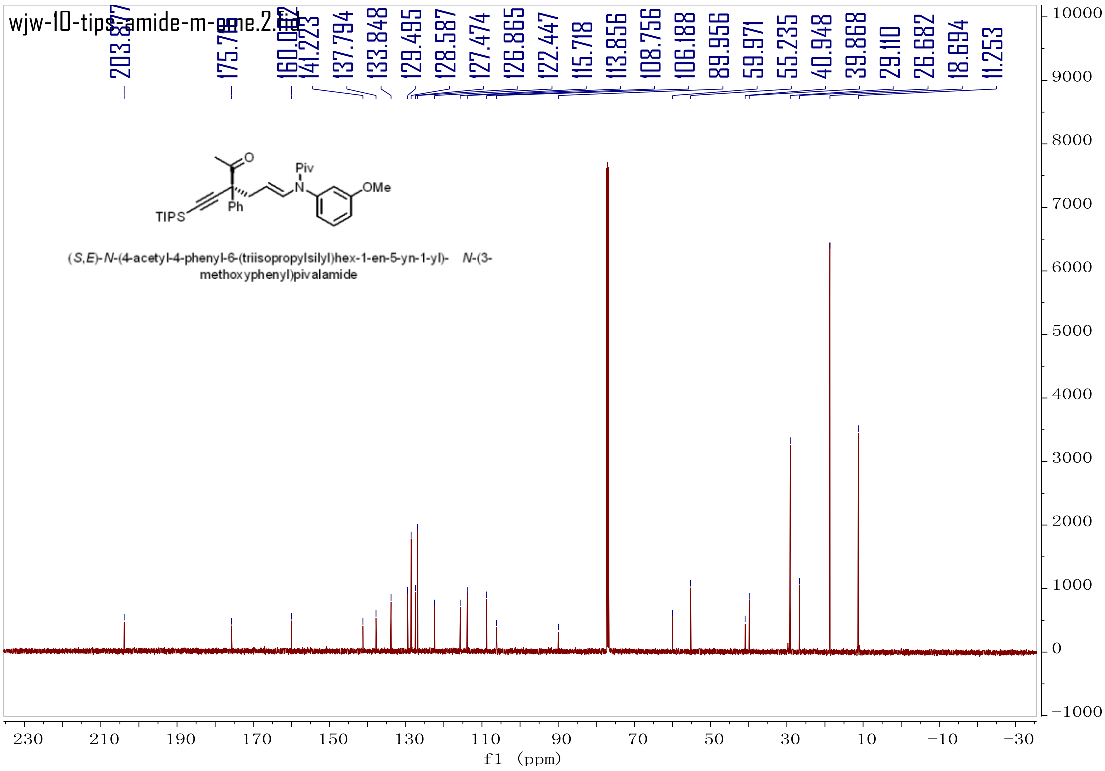

Supplementary Fig. 191 ^{13}C NMR spectrum of **3v**

(*S*,*E*)-N-(4-acetyl-4-phenyl-6-(triisopropylsilyl)hex-1-en-5-yn-1-yl)-N-(3-fluorophenyl)pivalamide (**3w**)

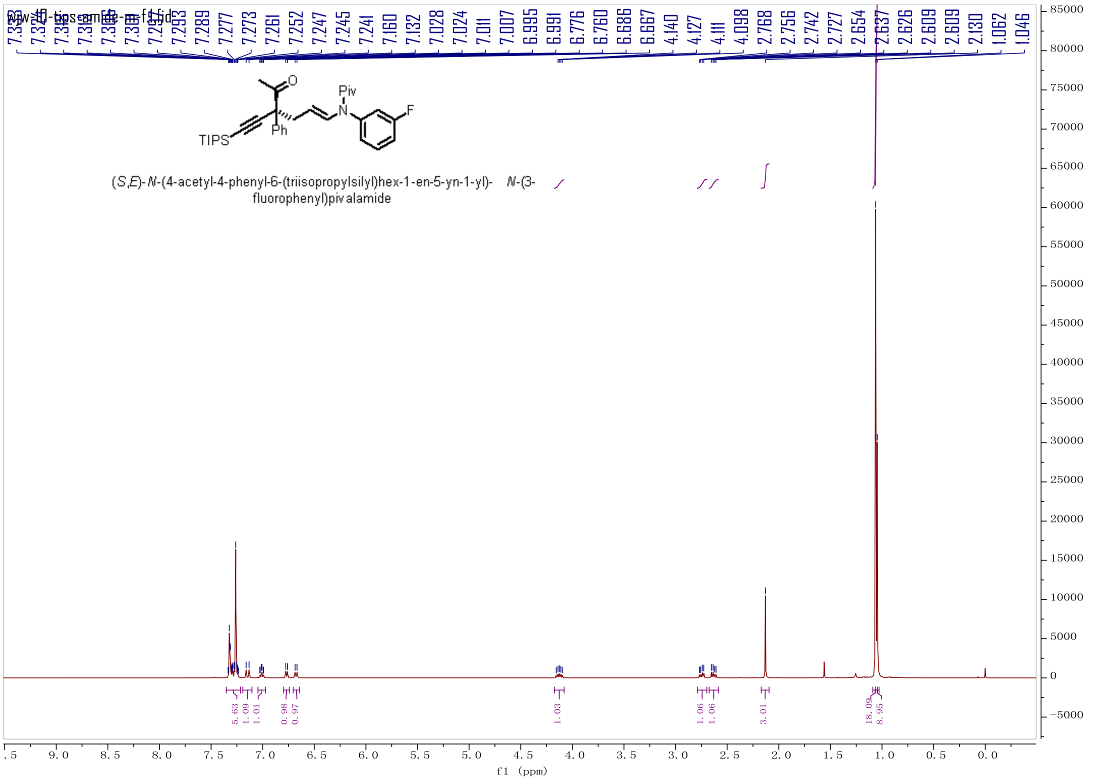

Supplementary Fig. 192 ^{1}H NMR spectrum of **3w**

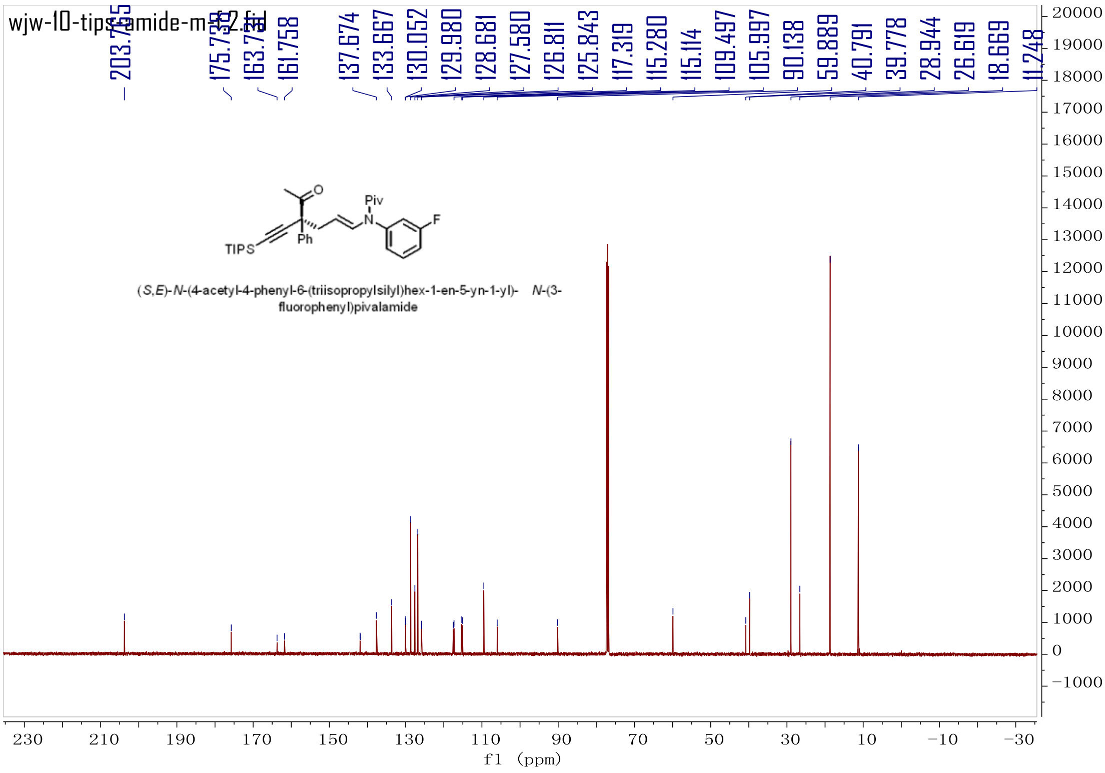

Supplementary Fig. 193 ^{13}C NMR spectrum of **3w**

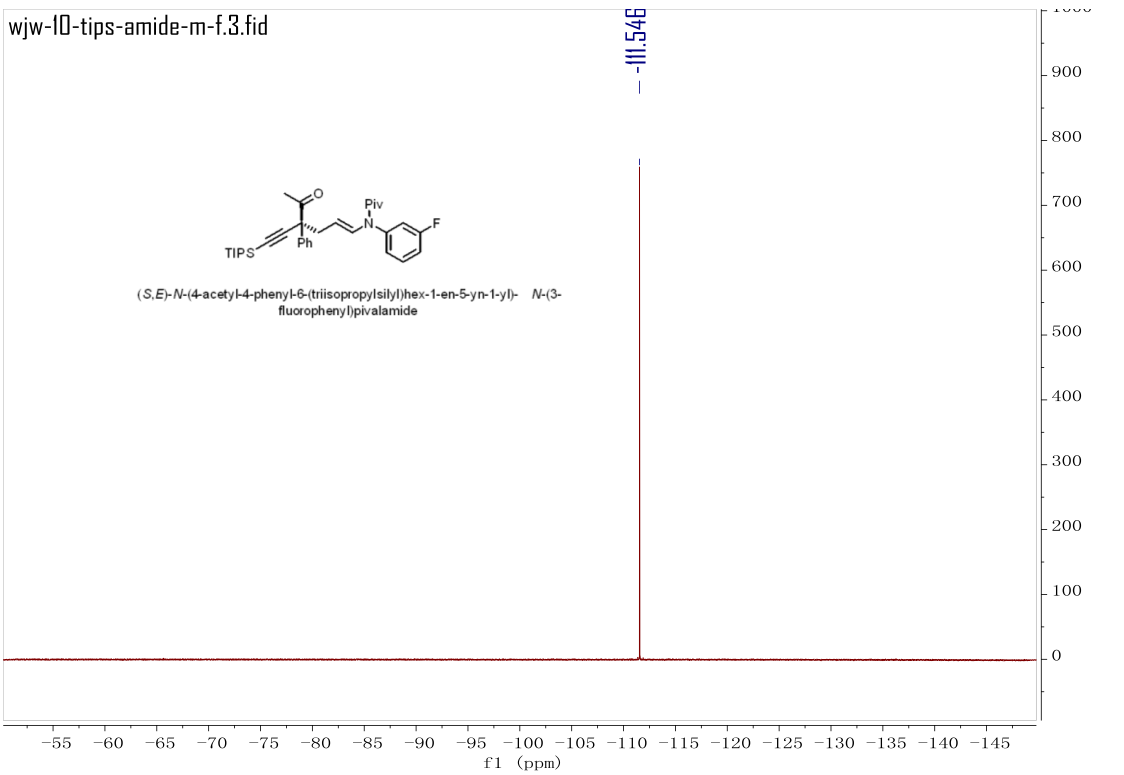

Supplementary Fig. 194 ^{19}F NMR spectrum of **3w**

(*S*,*E*)-N-(4-acetyl-4-phenyl-6-(triisopropylsilyl)hex-1-en-5-yn-1-yl)-N-(naphthalen-2-yl)pivalamide (**3x**)

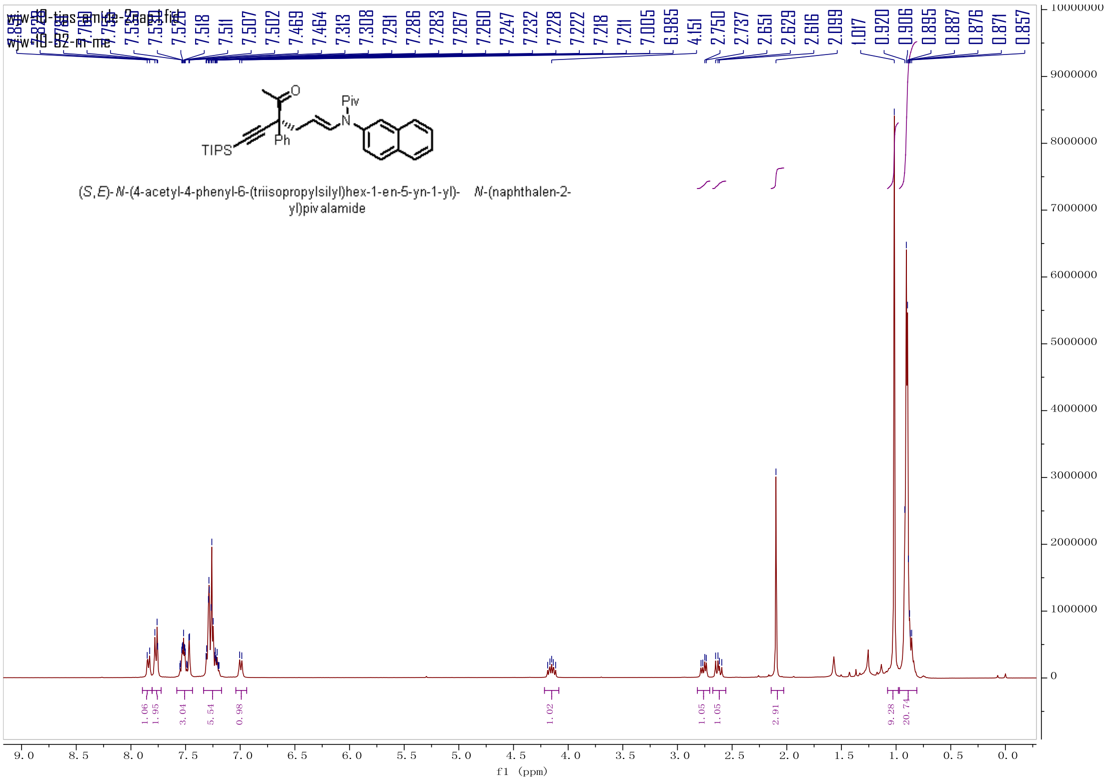

Supplementary Fig. 195 ^{1}H NMR spectrum of **3x**

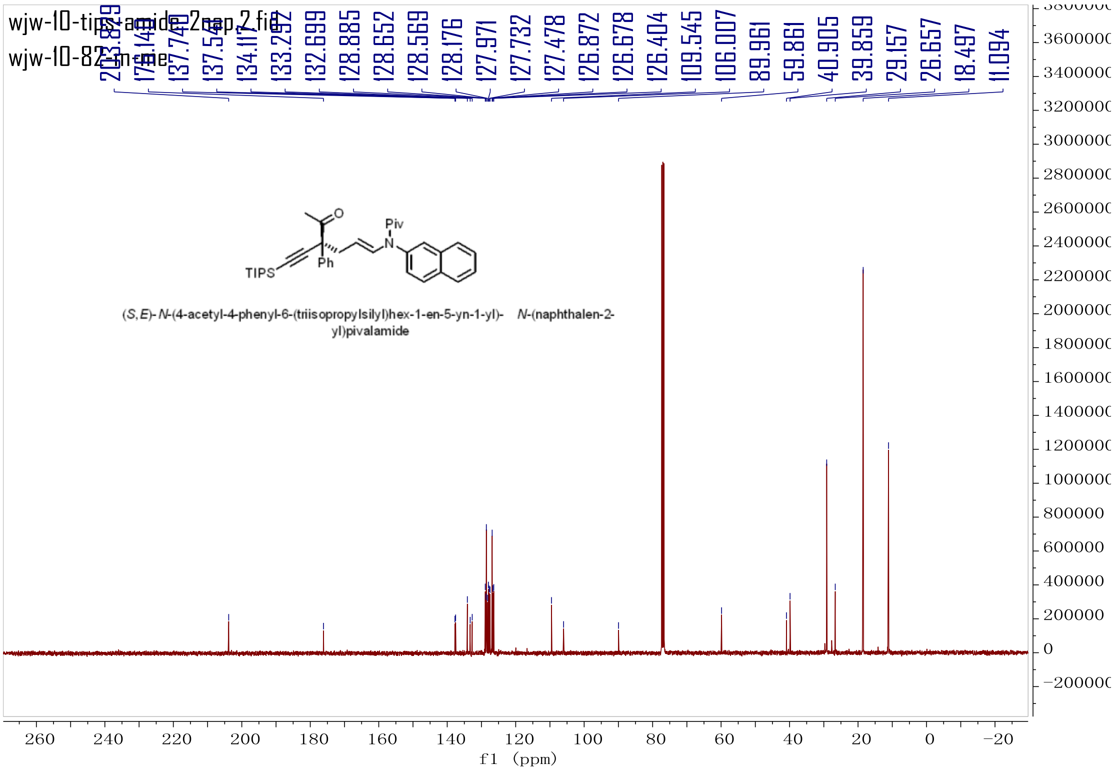

Supplementary Fig. 196 ^{13}C NMR spectrum of **3x**

(*S*,*E*)-N-(4-acetyl-4-phenyl-6-(triisopropylsilyl)hex-1-en-5-yn-1-yl)-N-phenylisobutyramide (**3y**)

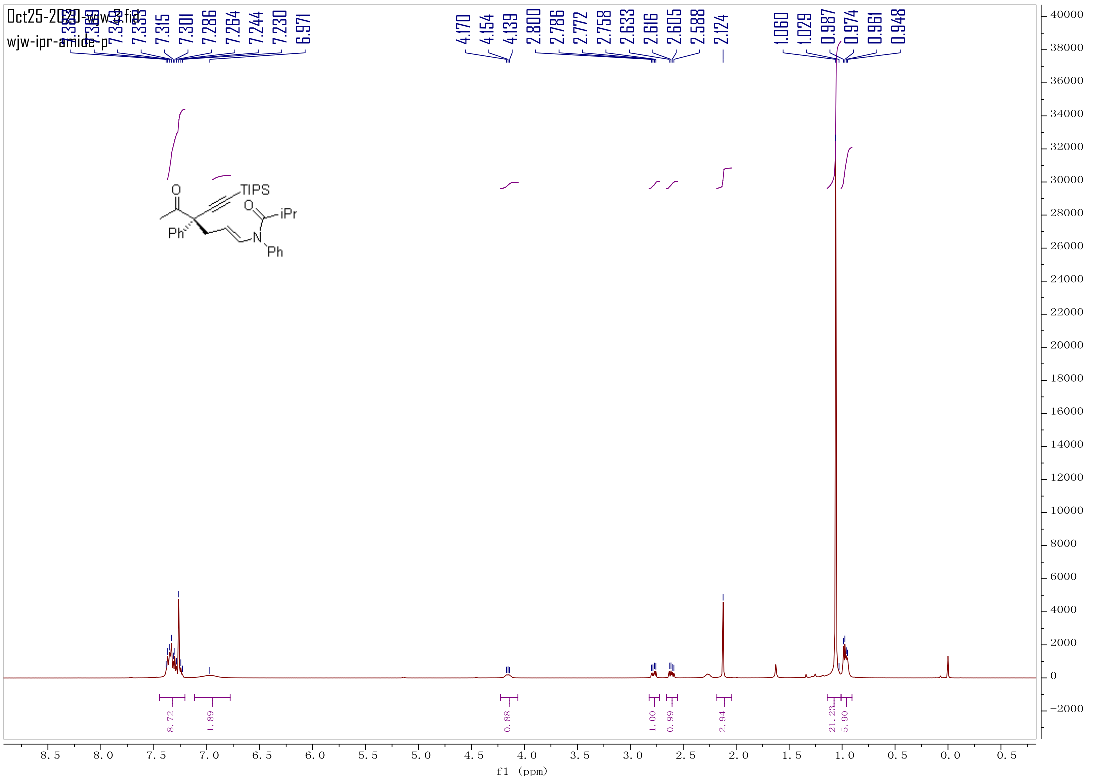

Supplementary Fig. 197 ^{1}H NMR spectrum of **3y**

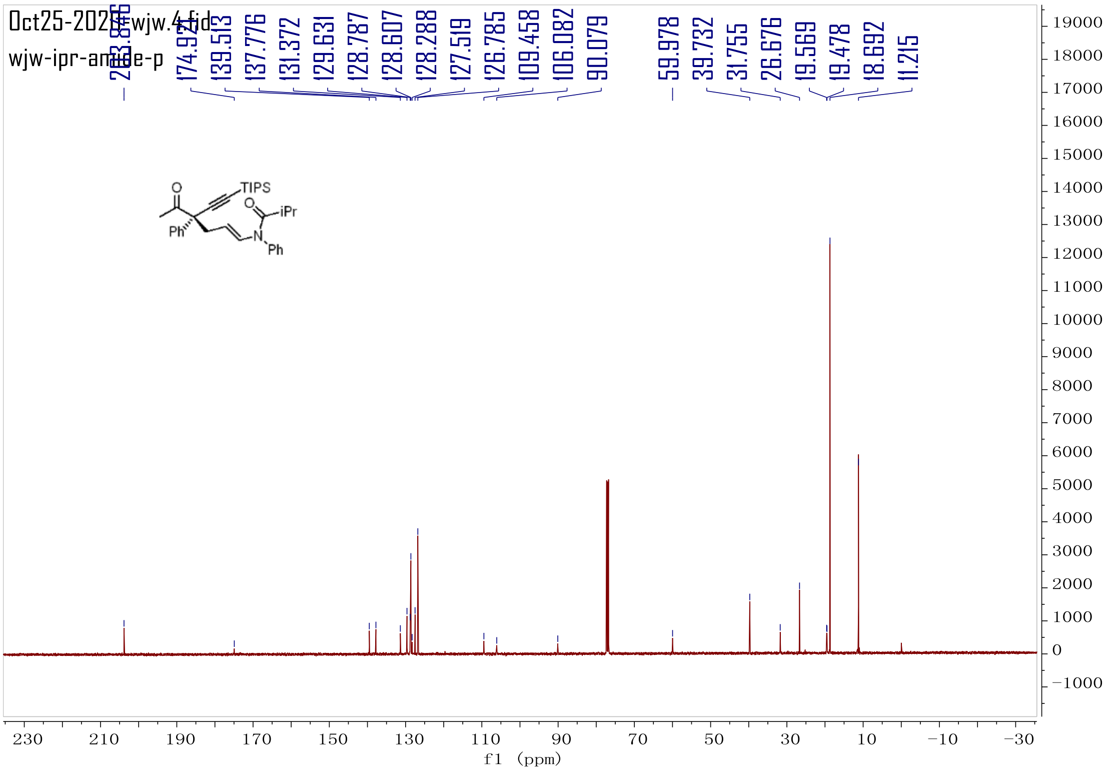

Supplementary Fig. 198 ^{13}C NMR spectrum of **3y**

(*S*,*E*)-N-(4-acetyl-4-phenyl-6-(triisopropylsilyl)hex-1-en-5-yn-1-yl)-N-phenylacetamide (**3z**)

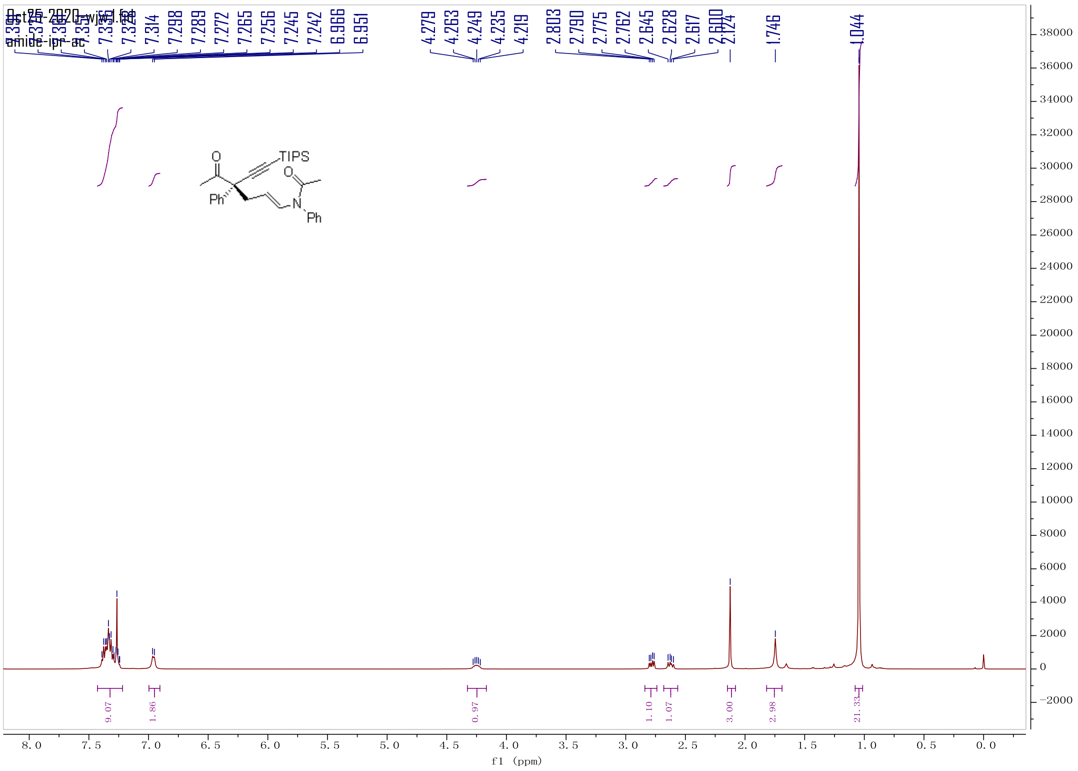

Supplementary Fig. 199 ^{1}H NMR spectrum of **3z**

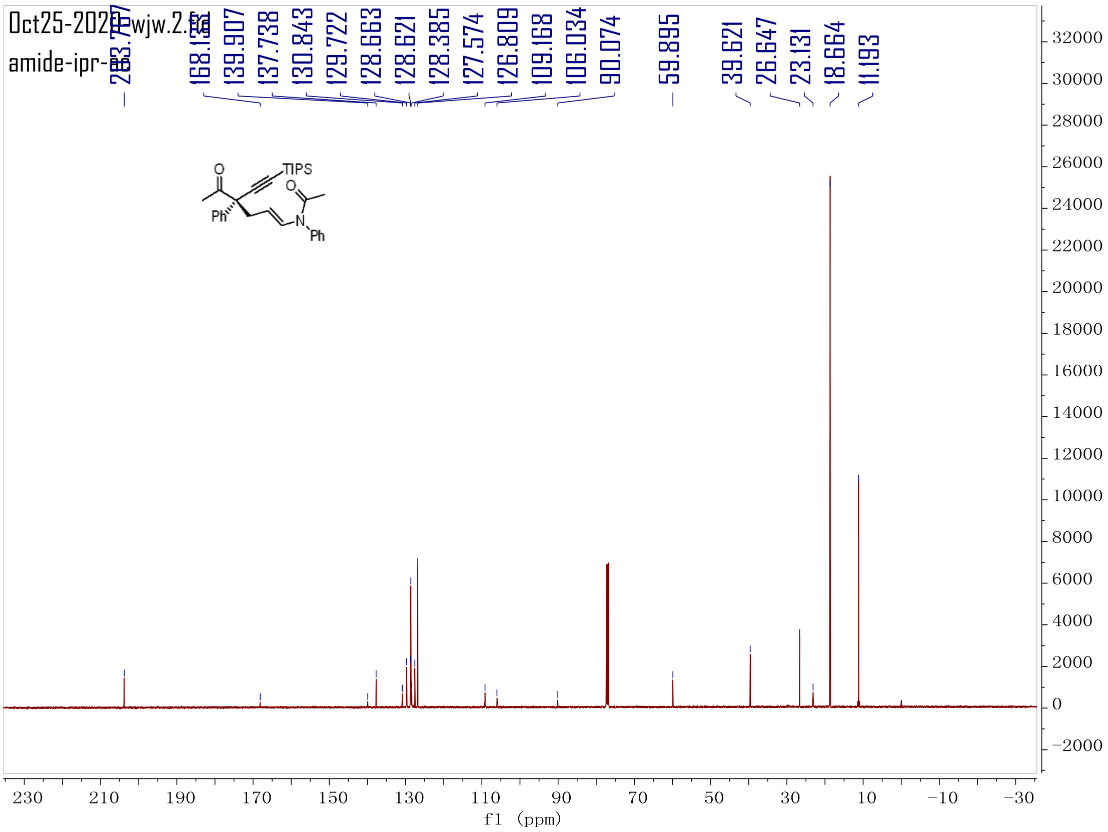

Supplementary Fig. 200 ^{13}C NMR spectrum of **3z**

N-((R,1E,5E)-4-acetyl-4,6-diphenylhexa-1,5-dien-1-yl)-N-phenylpivalamide (**5a**)

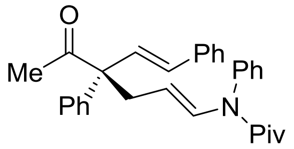

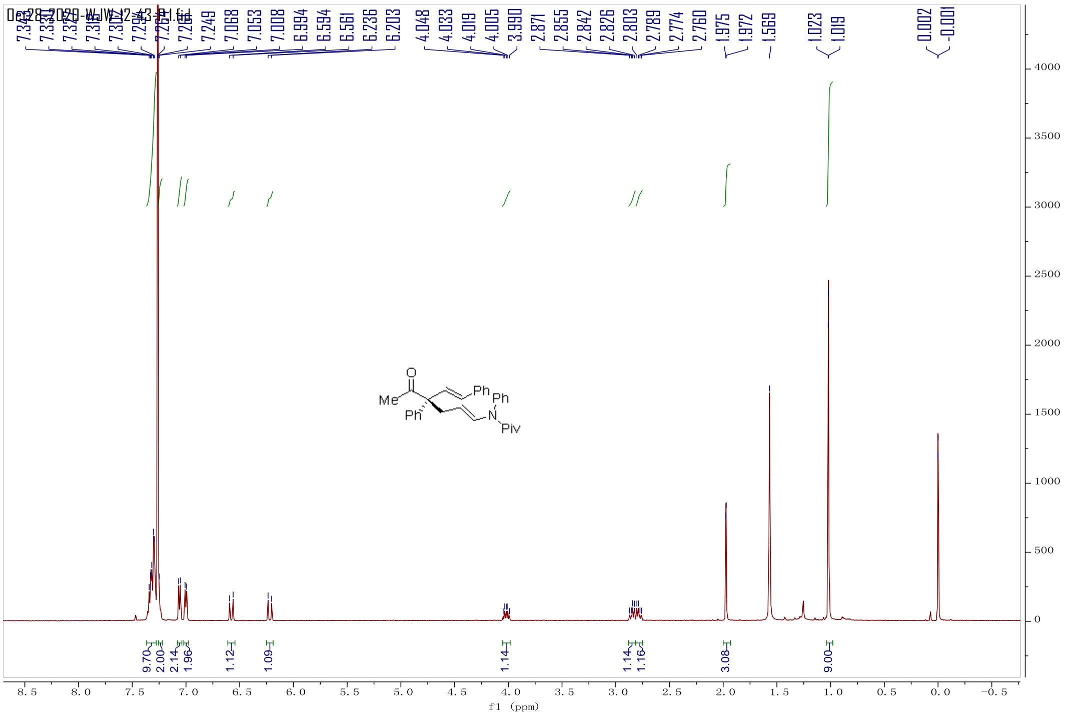

Supplementary Fig. 201 ^{1}H NMR spectrum of **5a**

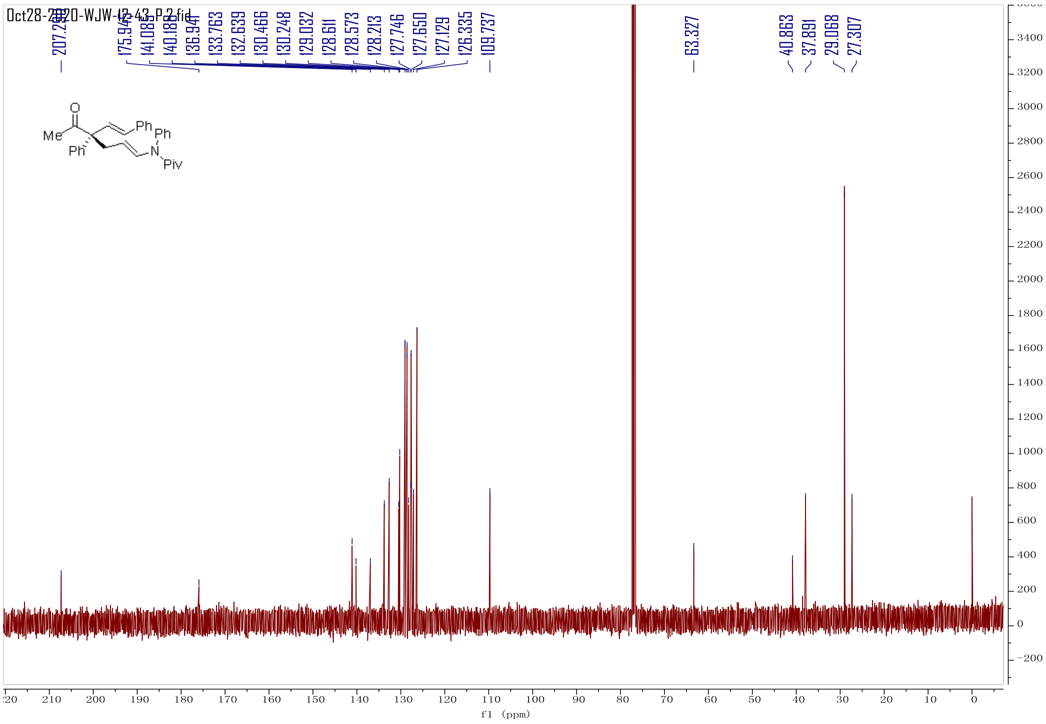

Supplementary Fig. 202 ^{13}C NMR spectrum of **5a**

ethyl (*E*)-2-acetyl-2-phenyl-5-(N-phenylpivalamido)pent-4-enoate (**5d**)

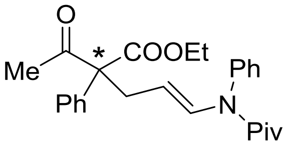

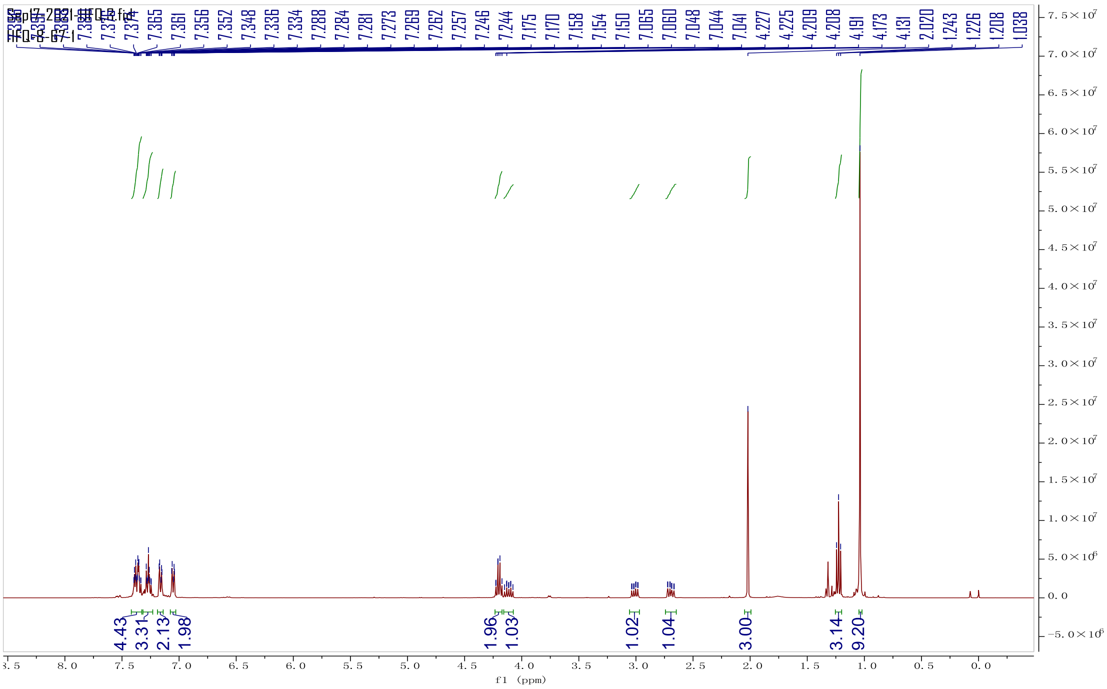

Supplementary Fig. 203 ^{1}H NMR spectrum of **5d**

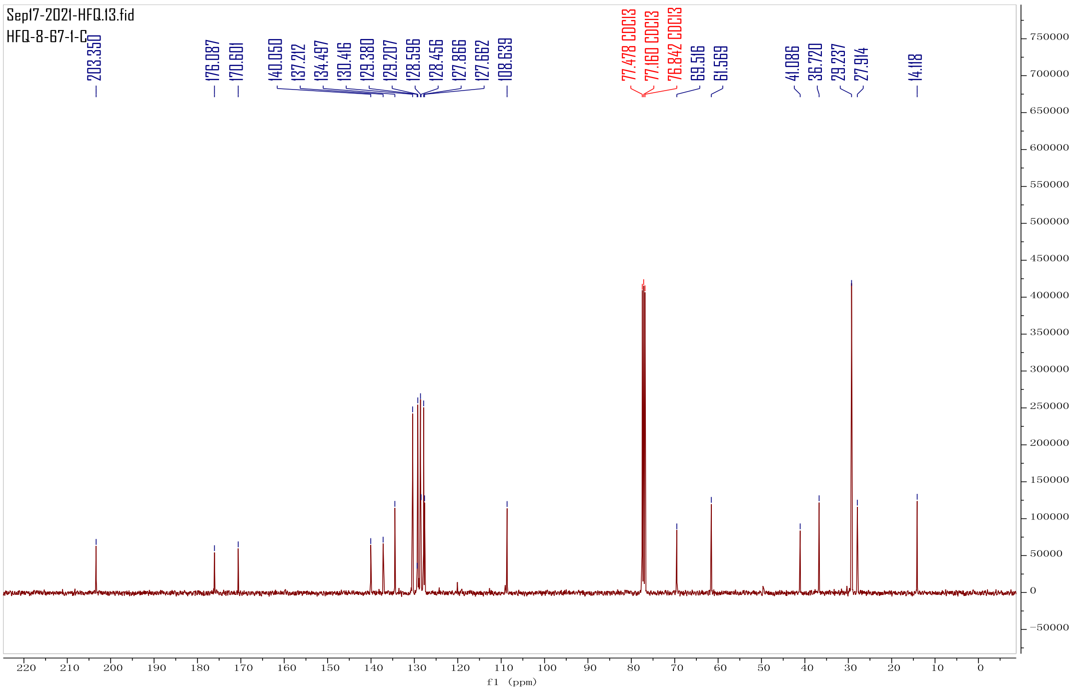

Supplementary Fig. 204 ^{13}C NMR spectrum of **5d**

(E)-N-(3-fluorophenyl)-N-(3-((4-methoxybenzyl)oxy)prop-1-en-1-yl)pivalamide (**6a**)

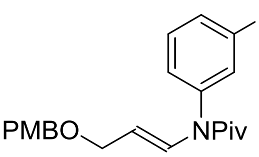

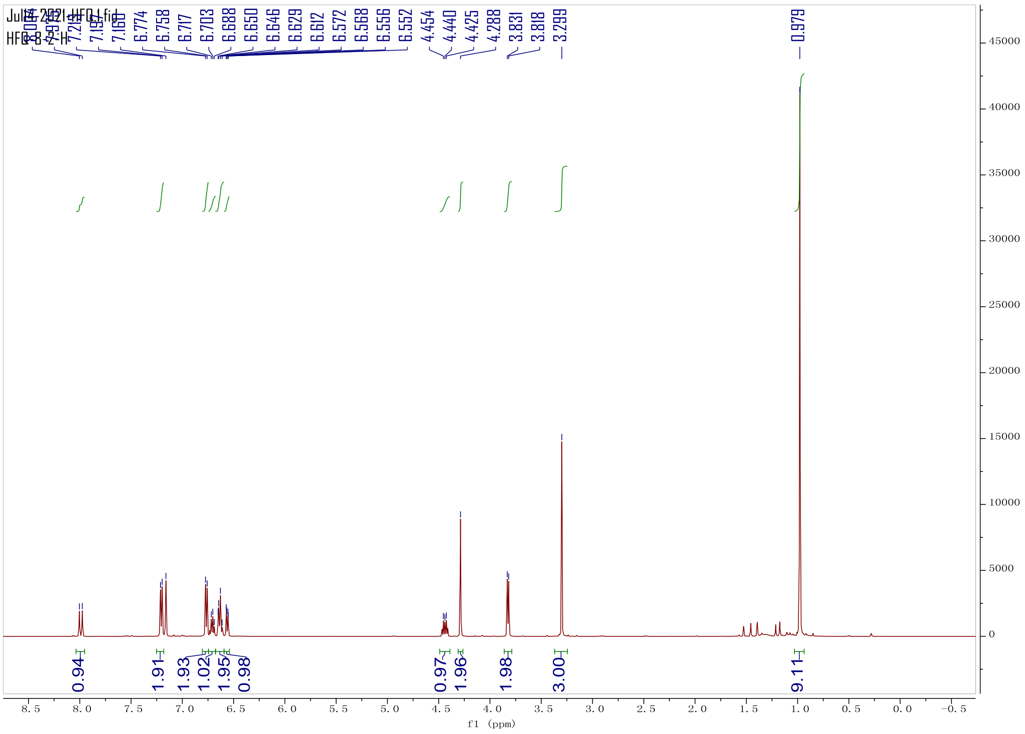

Supplementary Fig. 205 ^{1}H NMR spectrum of **6a**

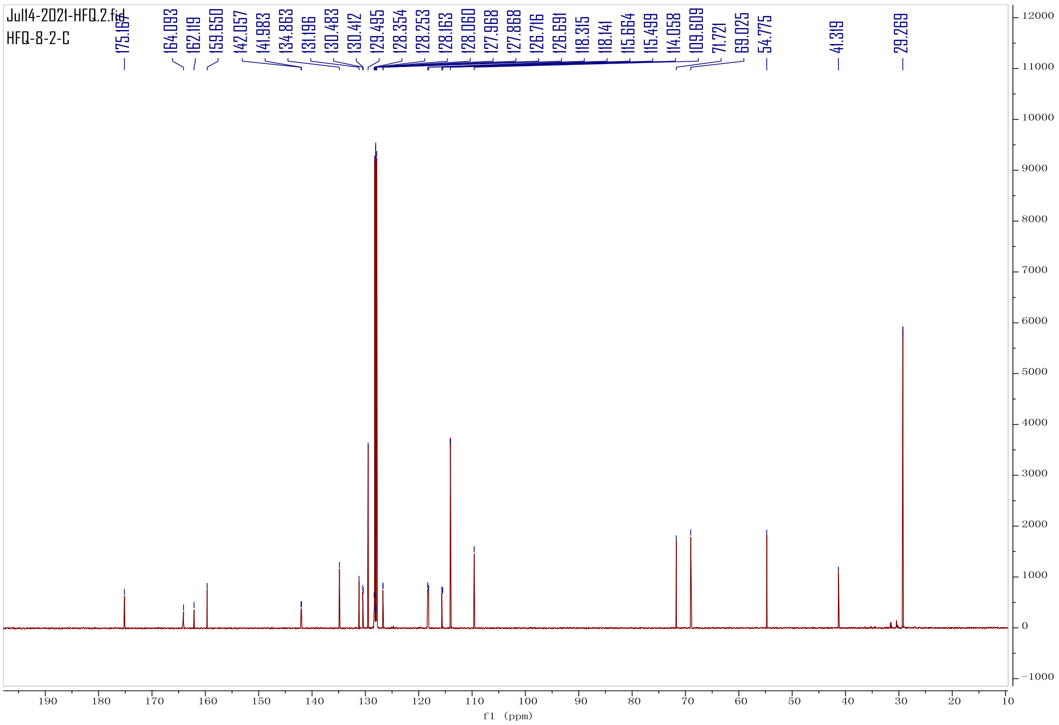

Supplementary Fig. 206 ^{13}C NMR spectrum of **6a**

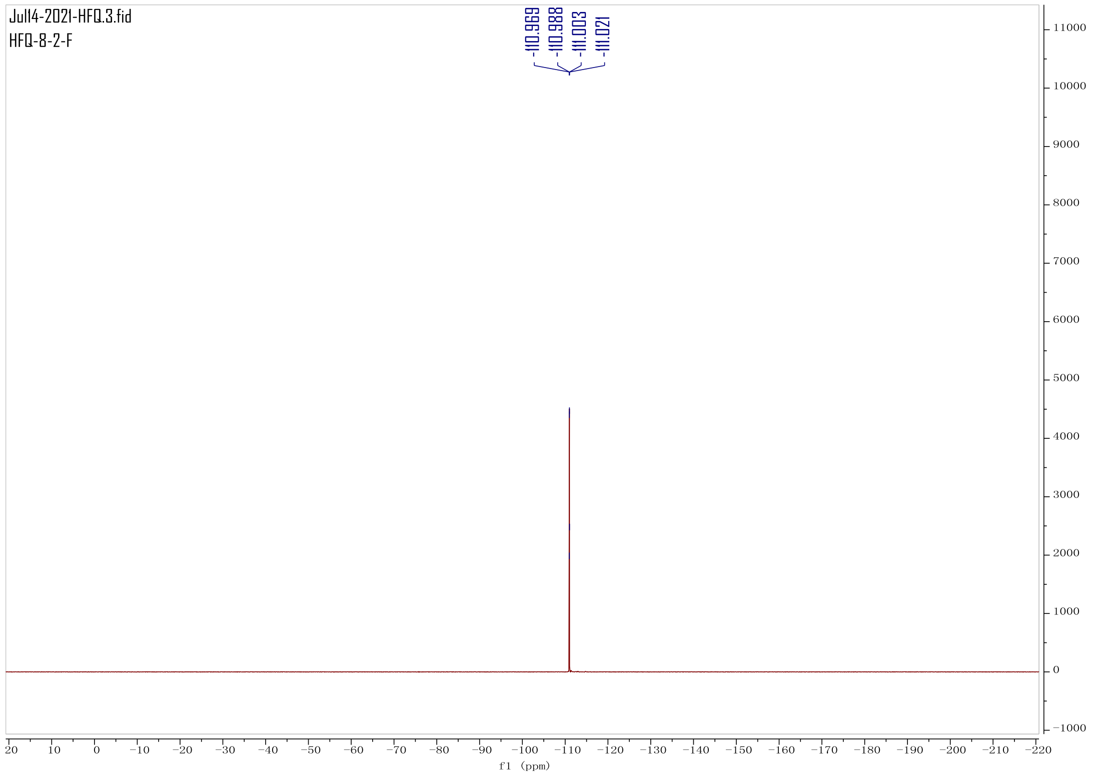

Supplementary Fig. 207 ^{19}F NMR spectrum of **6a**

(*S*)-N-(4-acetyl-4-phenyl-6-(triisopropylsilyl)hex-5-yn-1-yl)-N-phenylpivalamide (**7a**)

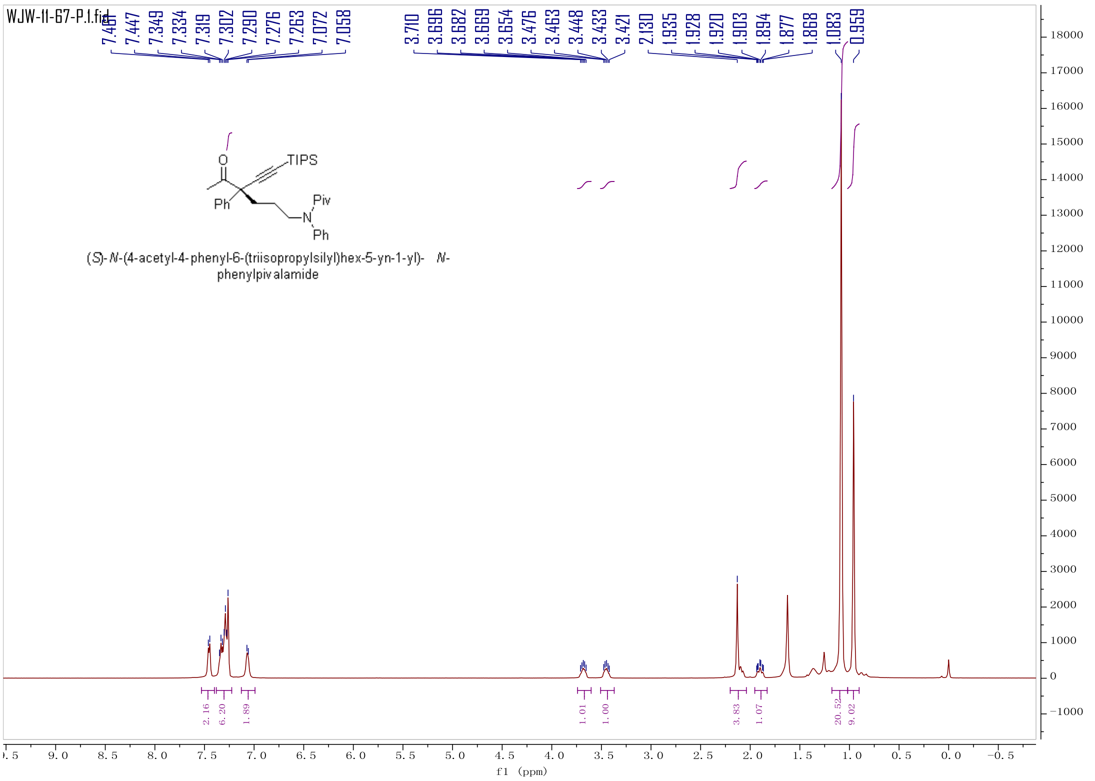

Supplementary Fig. 208 ^{1}H NMR spectrum of **7a**

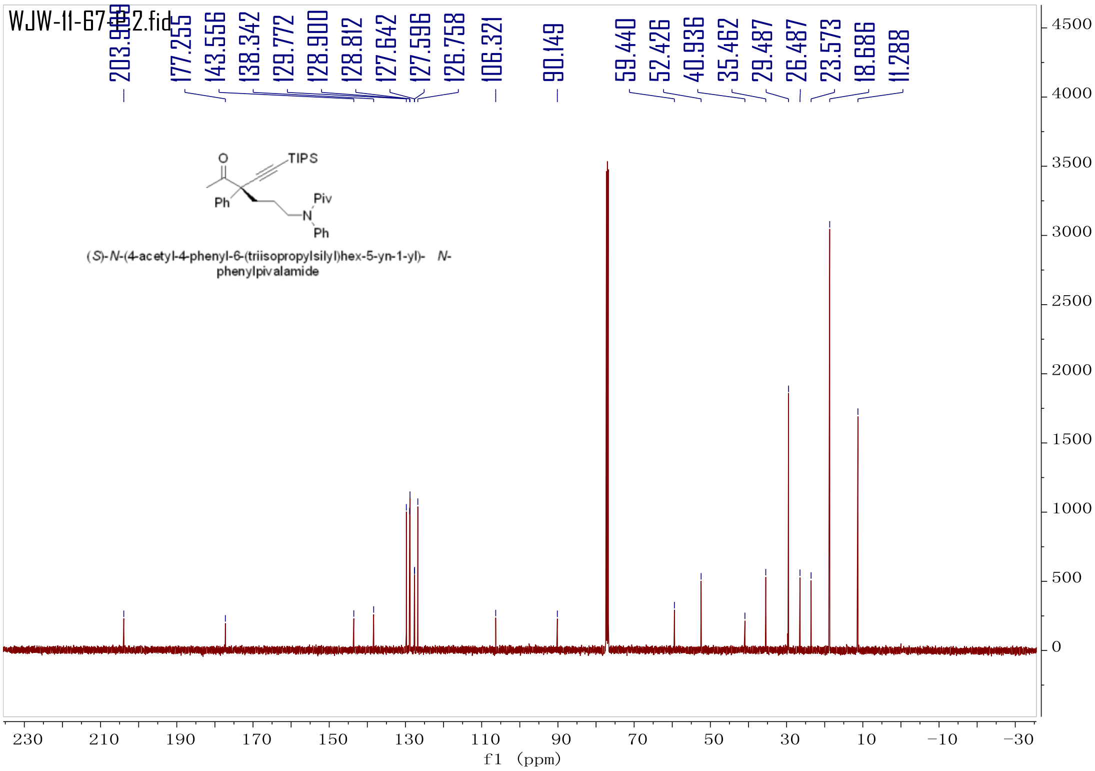

Supplementary Fig. 209 ^{13}C NMR spectrum of **7a**

(*R*)-N-(4-acetyl-4-phenyl-6-(triisopropylsilyl)hexyl)-N-phenylpivalamide (**8a**)

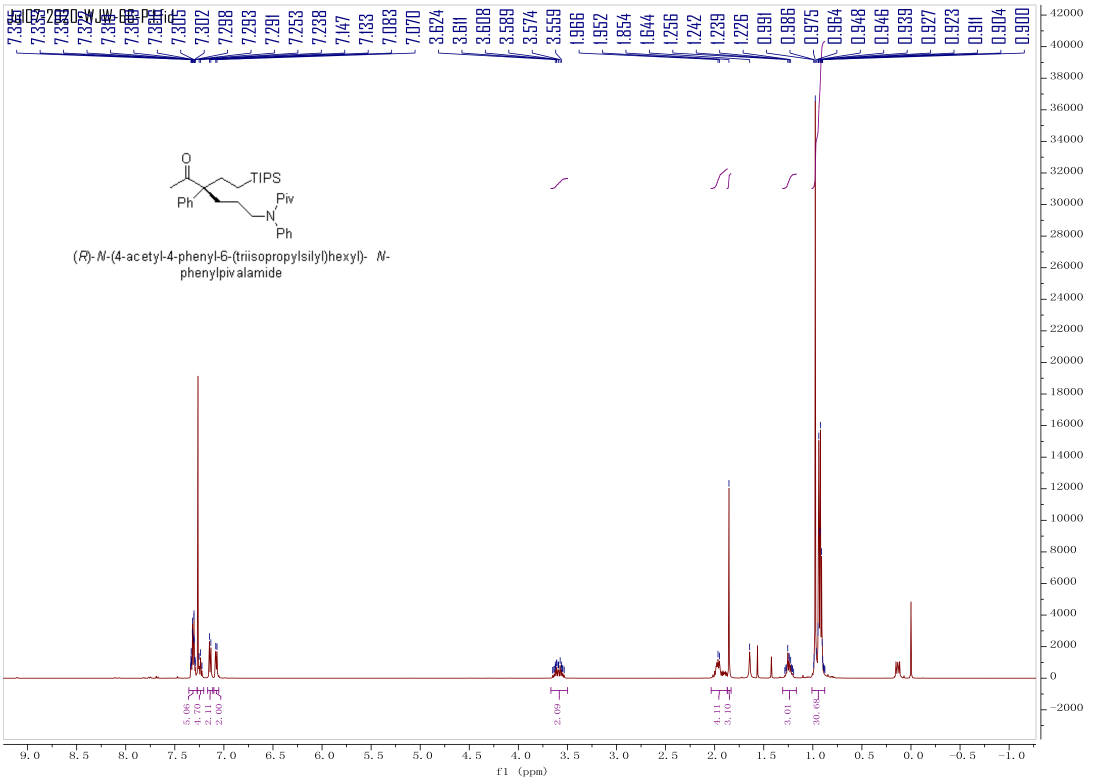

Supplementary Fig. 210 ^{1}H NMR spectrum of **8a**

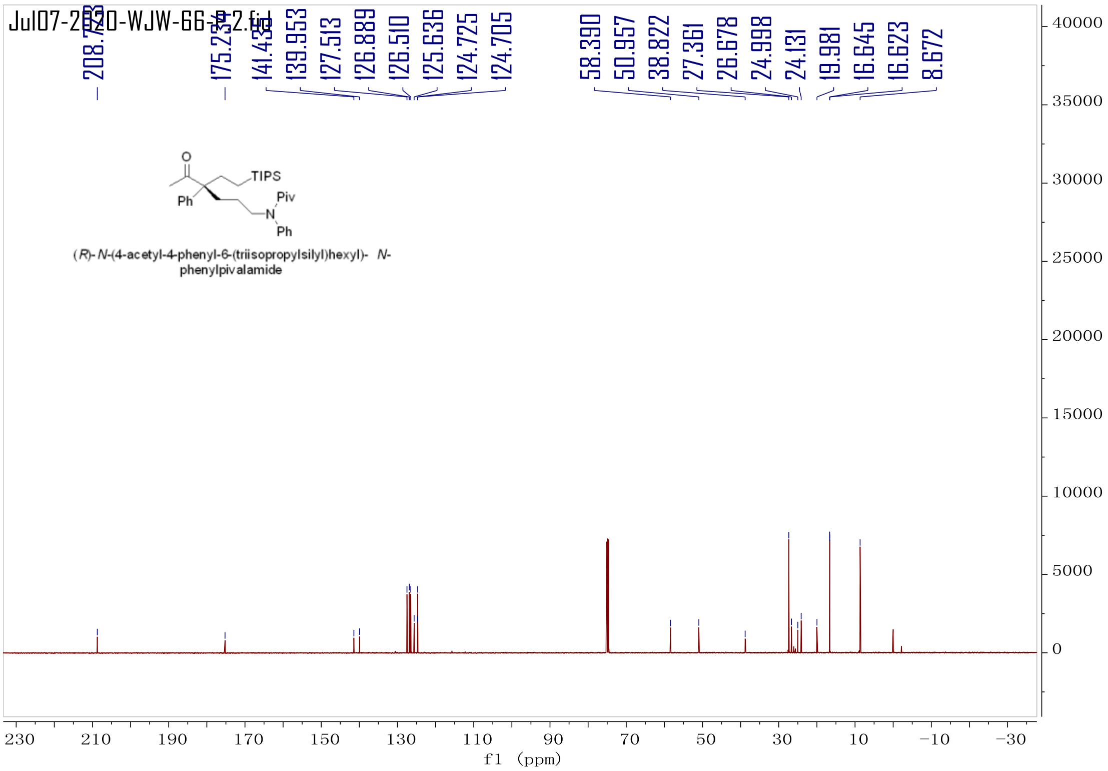

Supplementary Fig. 211 ^{13}C NMR spectrum of **8a**

(*S*,*E*)-N-(4-(1-benzyl-1H-1,2,3-triazol-4-yl)-5-oxo-4-phenylhex-1-en-1-yl)-N-phenylpivalamide (**9m**)

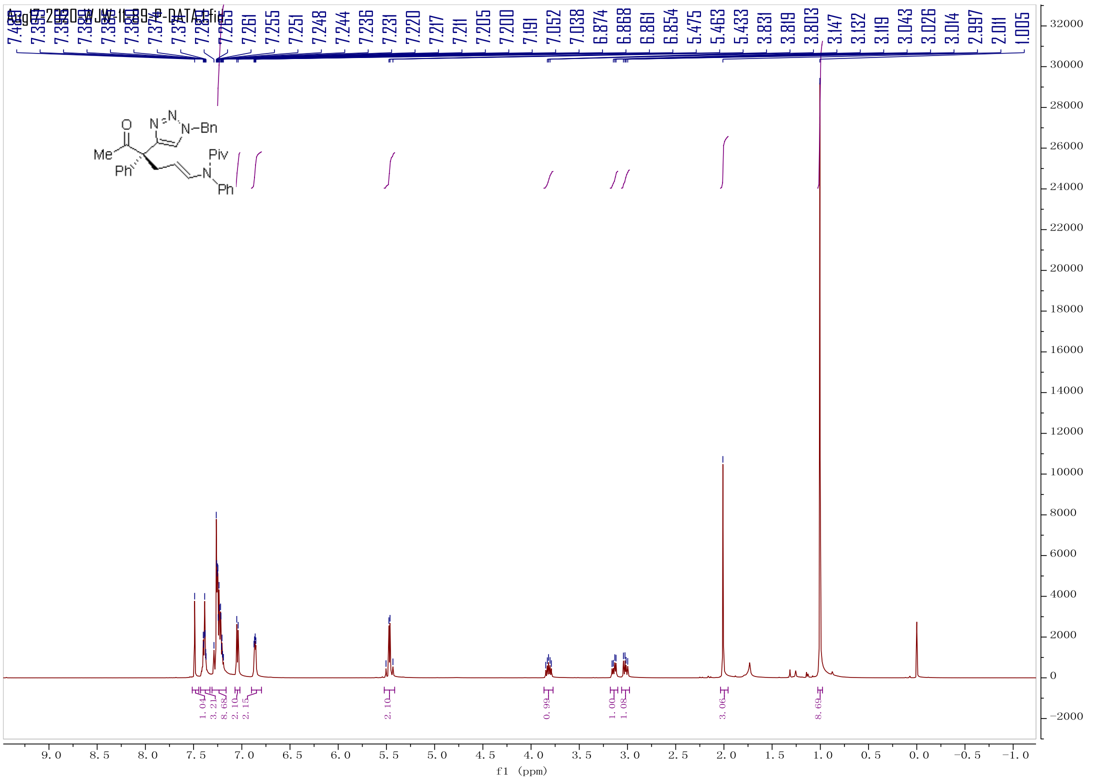

Supplementary Fig. 212 ^{1}H NMR spectrum of **9m**

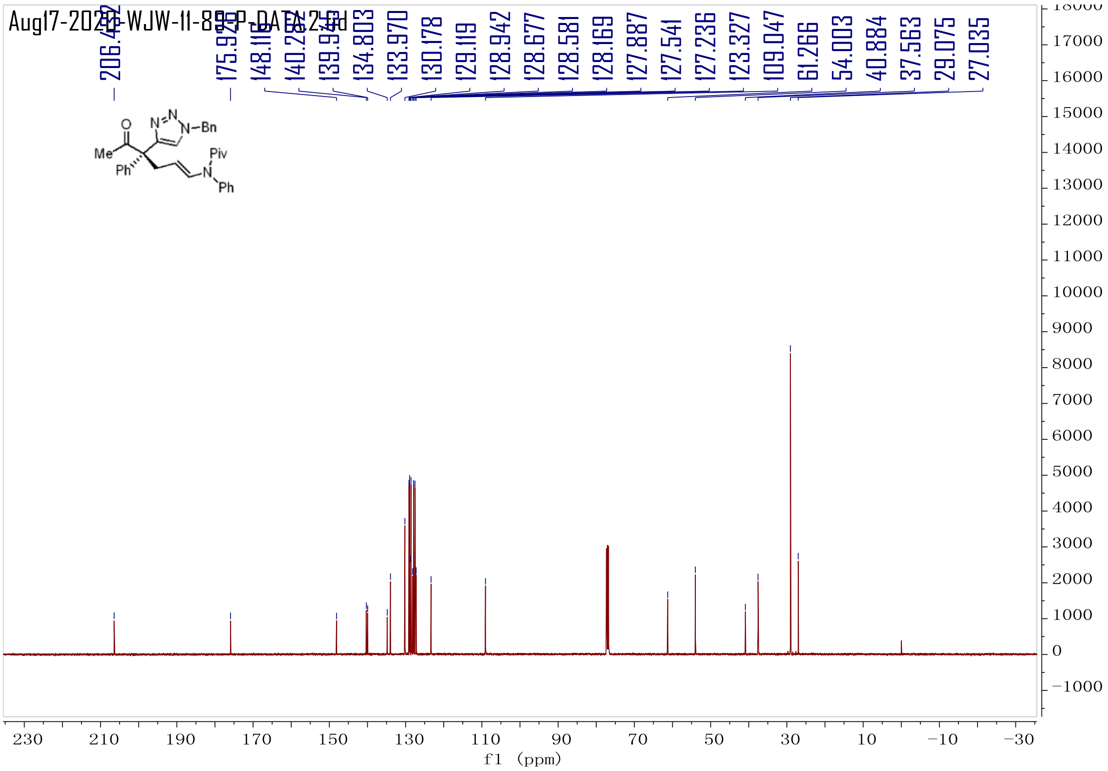

Supplementary Fig. 213 ^{13}C NMR spectrum of **9m**

(*S*)-4-acetyl-4-phenyl-6-(triisopropylsilyl)hex-5-ynal (**10a**)

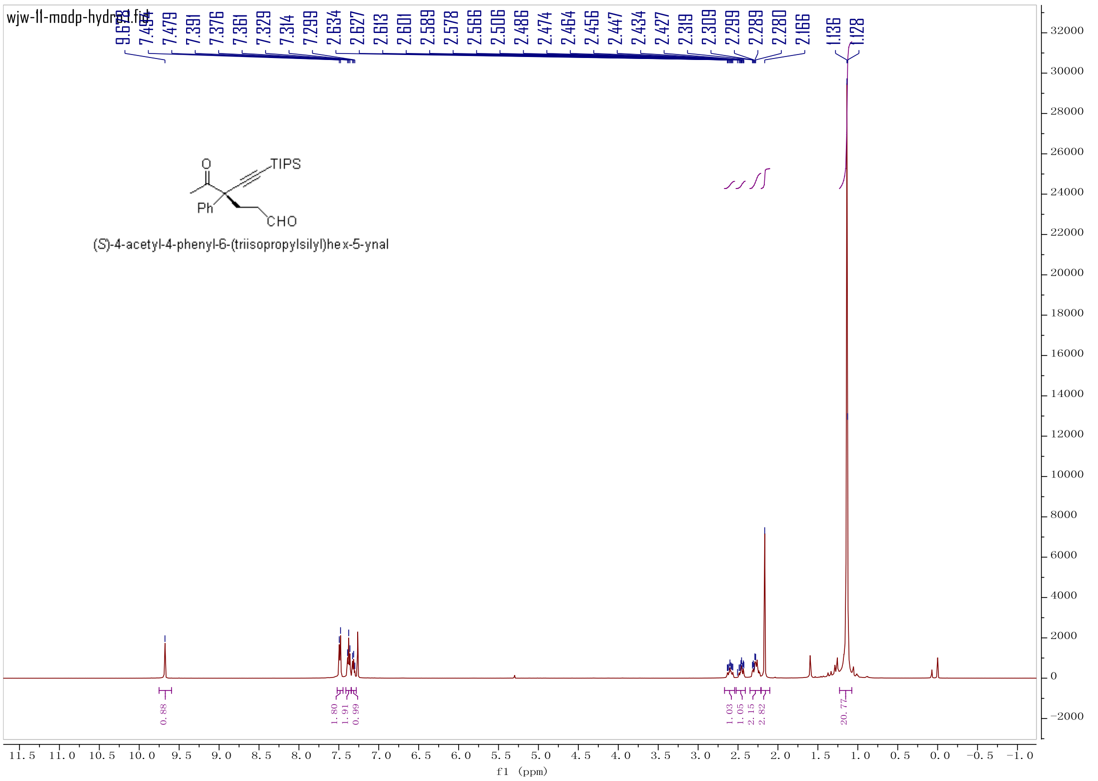

Supplementary Fig. 214 ^{1}H NMR spectrum of **10a**

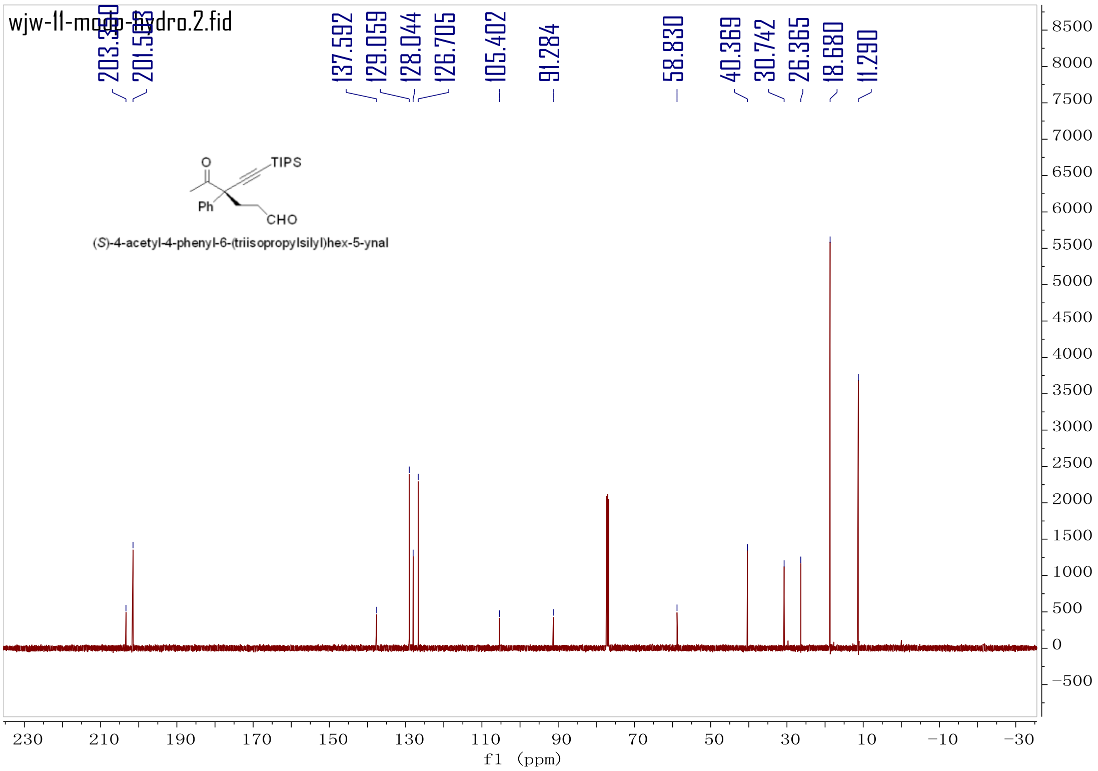

Supplementary Fig. 215 ^{13}C NMR spectrum of **10a**

(*S*)-1-((triisopropylsilyl)ethynyl)-5,6-dihydro-[1,1'-biphenyl]-2(1H)-one (**11a**)

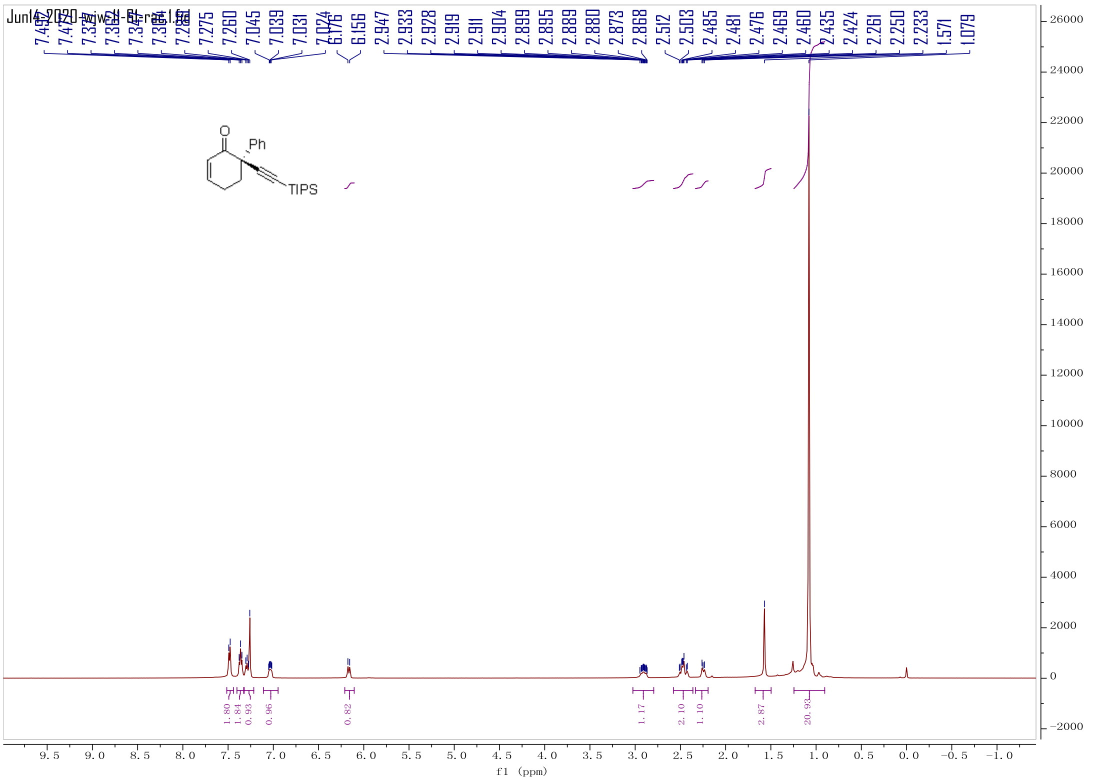

Supplementary Fig. 216 ^{1}H NMR spectrum of **11a**

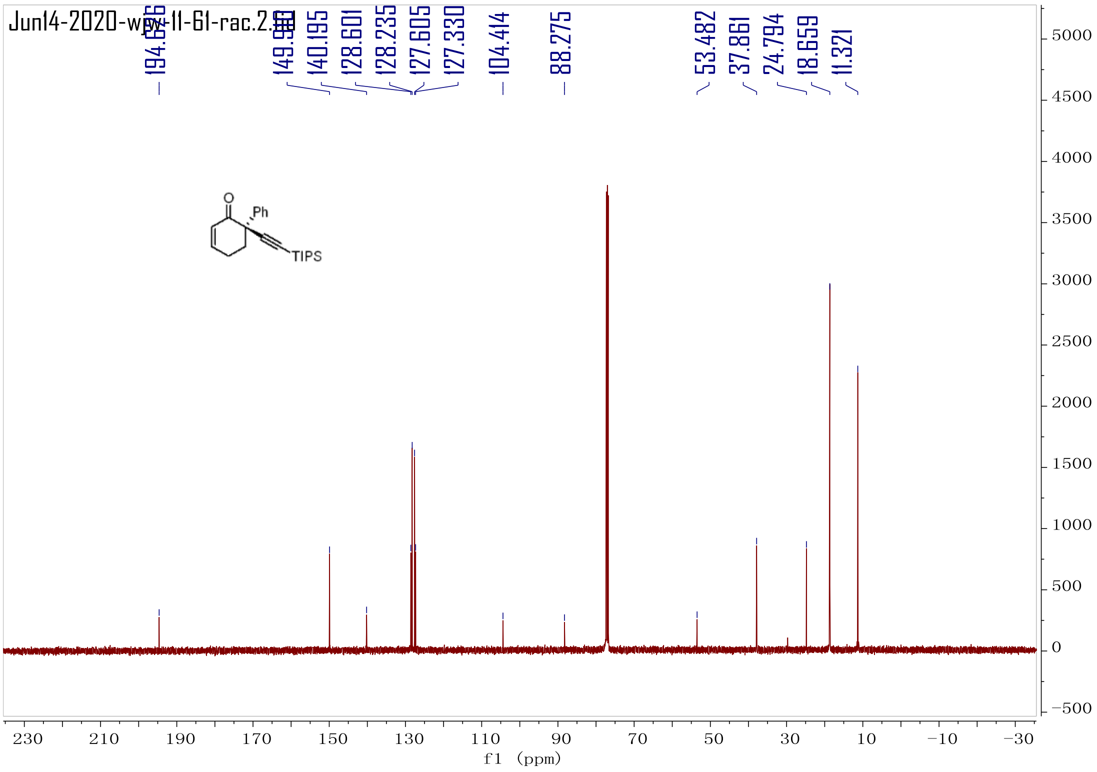

Supplementary Fig. 217 ^{13}C NMR spectrum of **11a**

N-((4*S*,*E*)-4-(1-hydroxyethyl)-4-phenyl-6-(triisopropylsilyl)hex-1-en-5-yn-1-yl)-N-phenylpivalamide (**12a**)

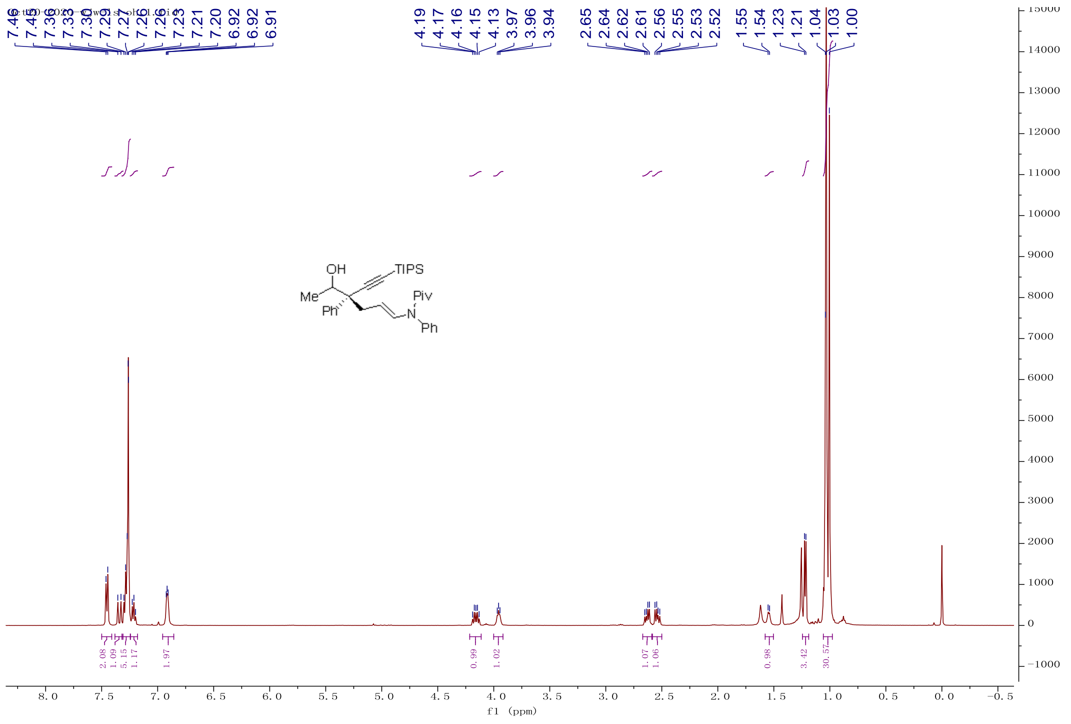

Supplementary Fig. 218 ^{1}H NMR spectrum of **12a**

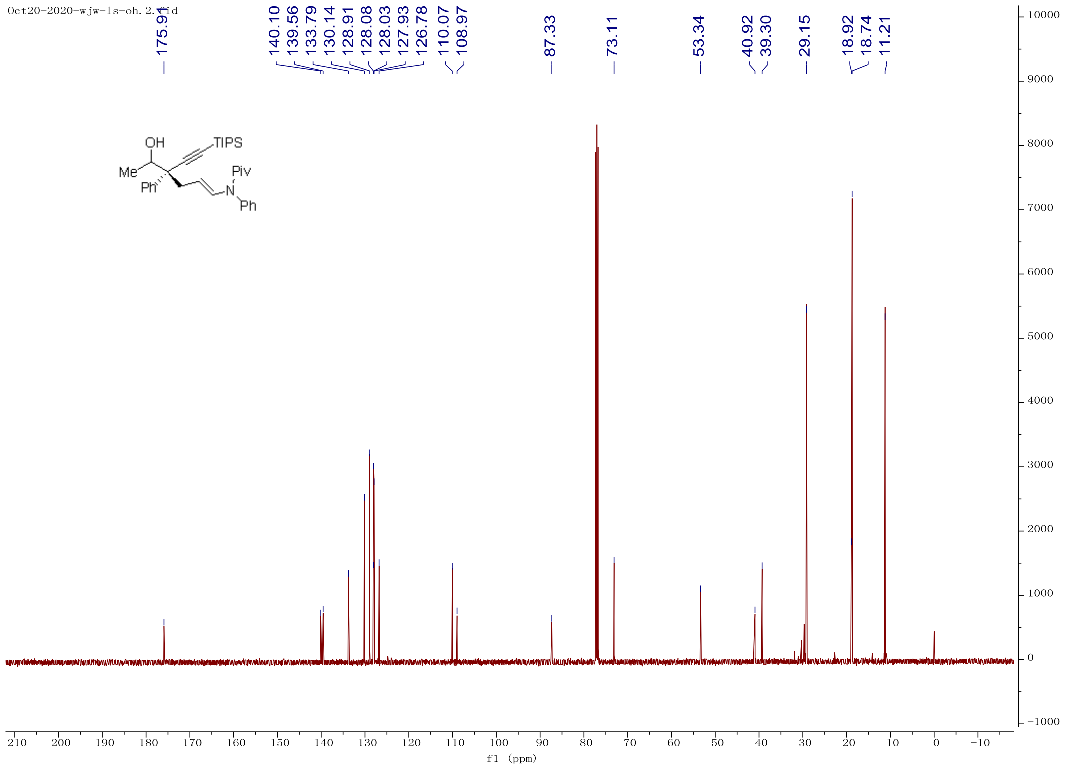

Supplementary Fig. 219 ^{13}C NMR spectrum of **12a**

Triisopropyl(((2*R*,3*S*)-2-methyl-3-phenyl-3,4-dihydro-2H-pyran-3-yl)ethynyl)silane (**13a**)

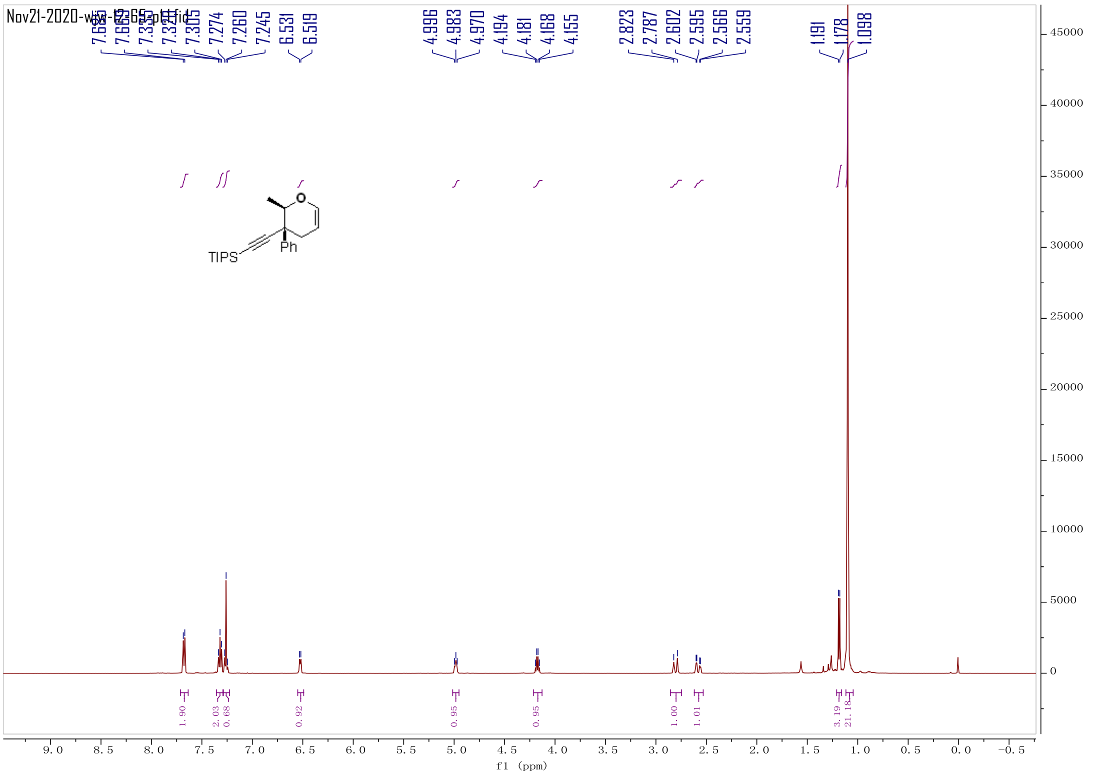

Supplementary Fig. 220 ^{1}H NMR spectrum of **13a**

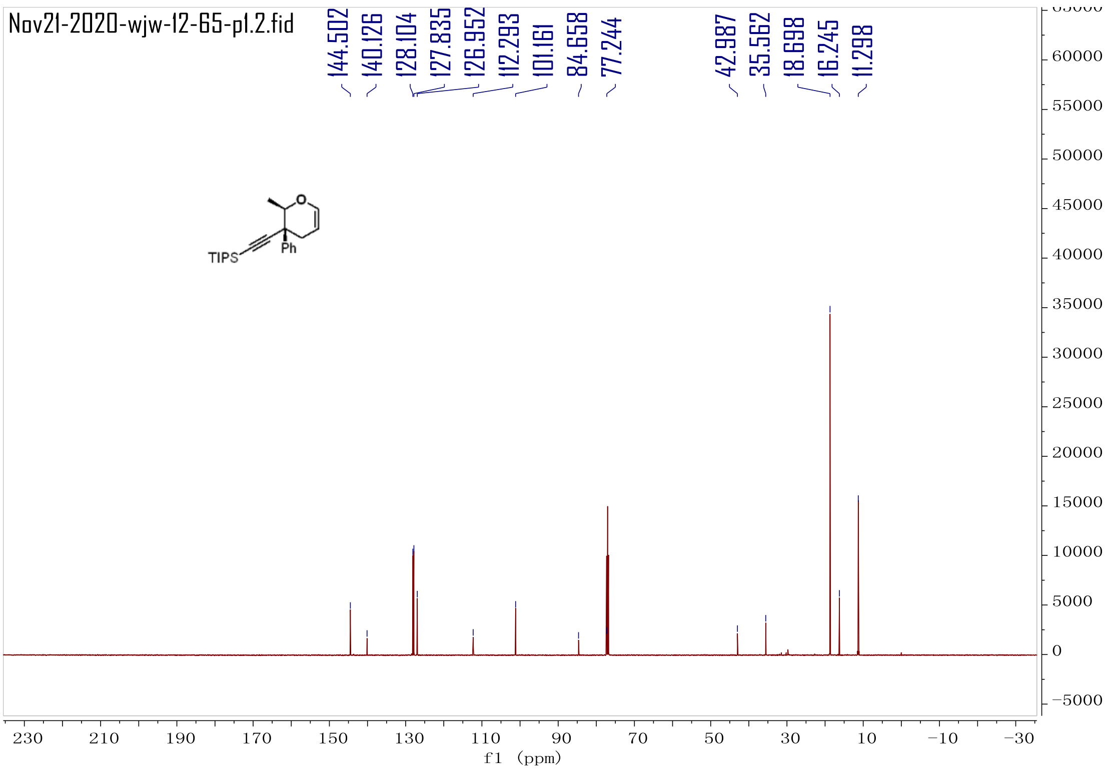

Supplementary Fig. 221 ^{13}C NMR spectrum of **13a**

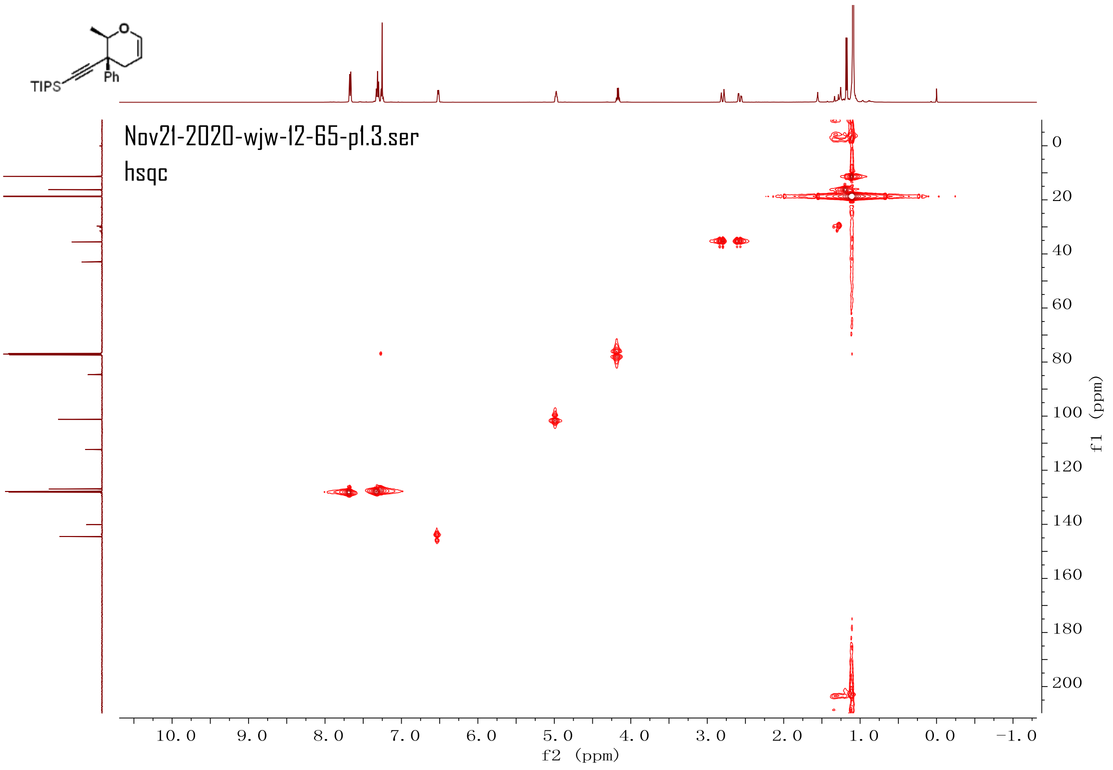

Supplementary Fig. 222 HSQC NMR spectrum of **13a**

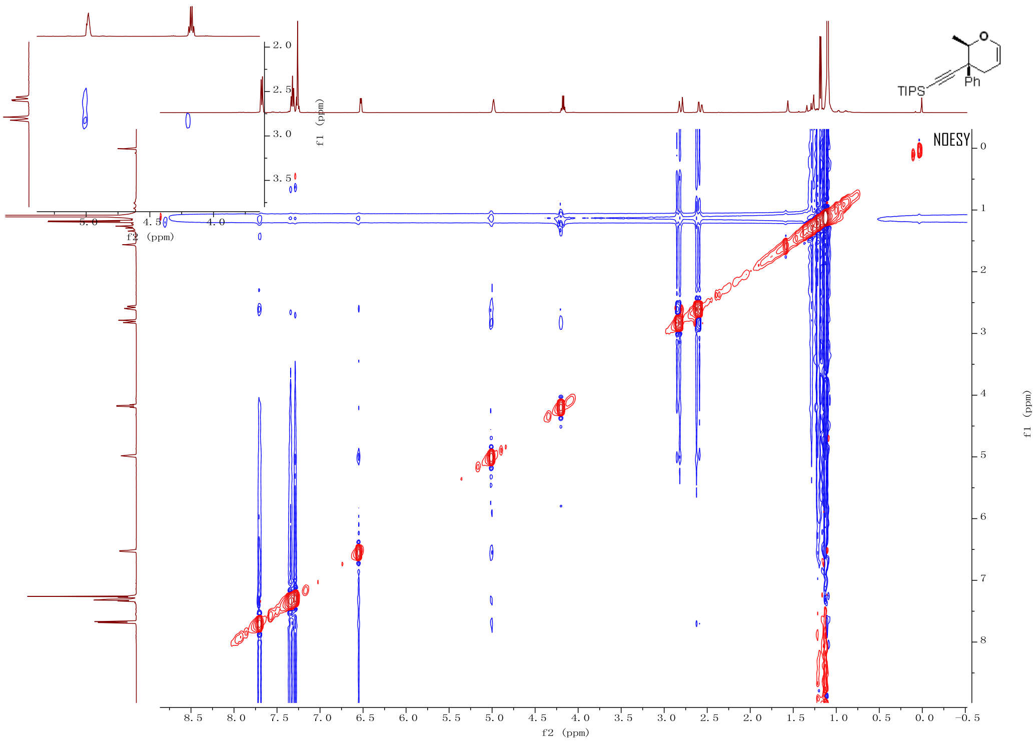

Supplementary Fig. 223 NOESY NMR spectrum of **13a**

N-((*2R,5S,6R*)-6-methyl-5-phenyl-5-((triisopropylsilyl)ethynyl)tetrahydro-2H-pyran-2-yl)-N-phenylpivalamide (**13b**)

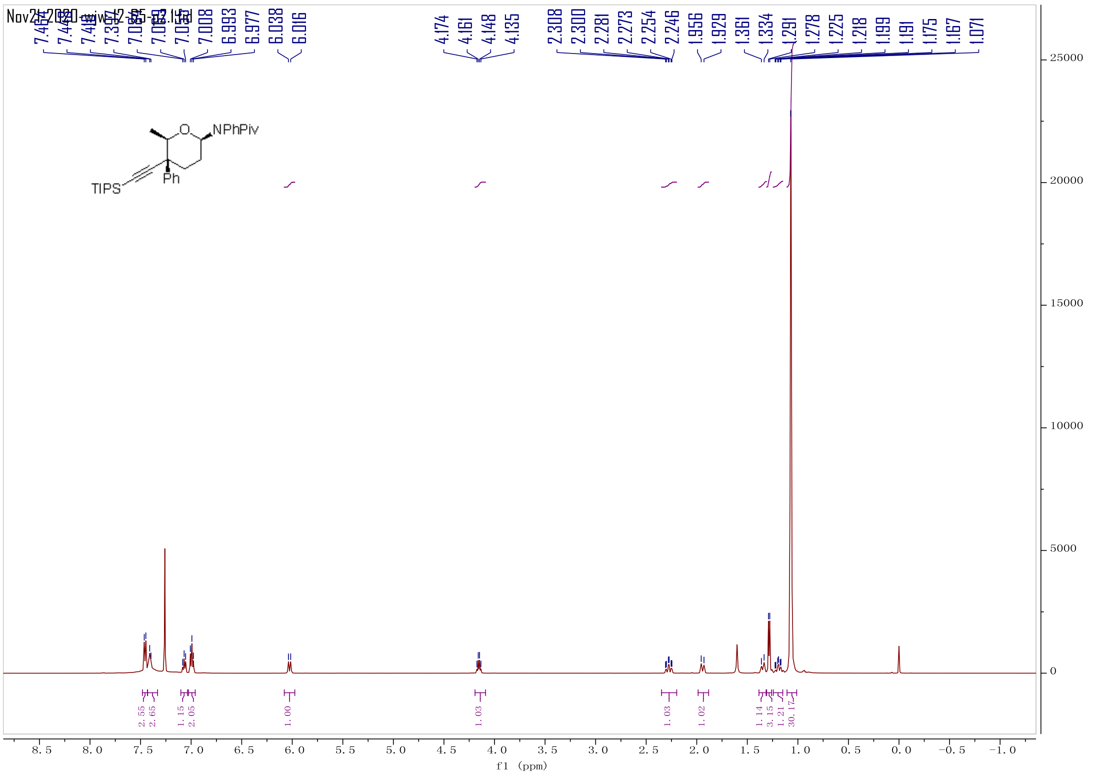

Supplementary Fig. 224 ^{1}H NMR spectrum of **13b**

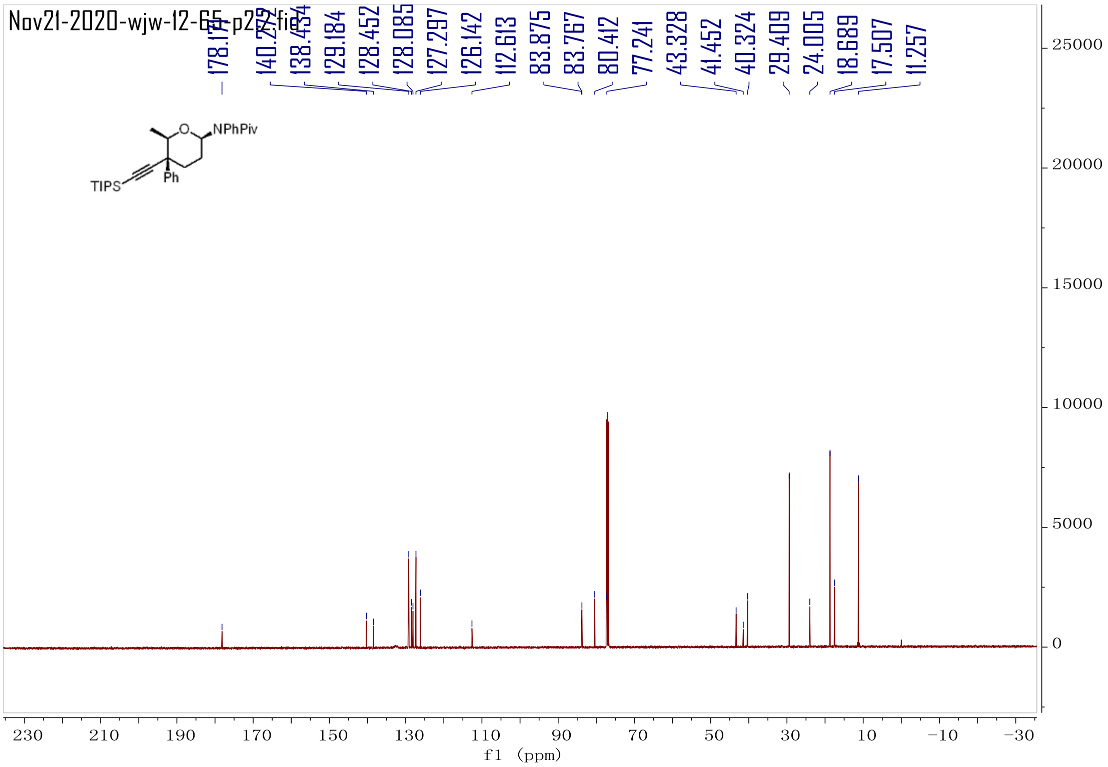

Supplementary Fig. 225 ^{13}C NMR spectrum of **13b**

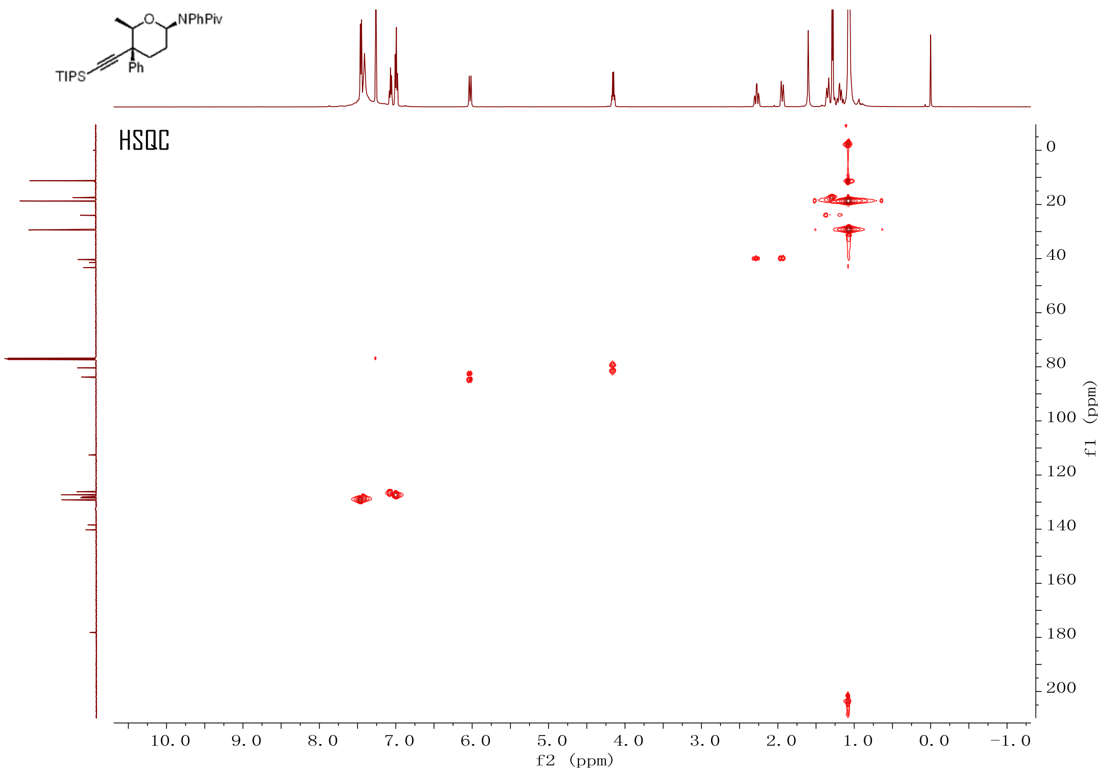

Supplementary Fig. 226 HSQC NMR spectrum of **13b**

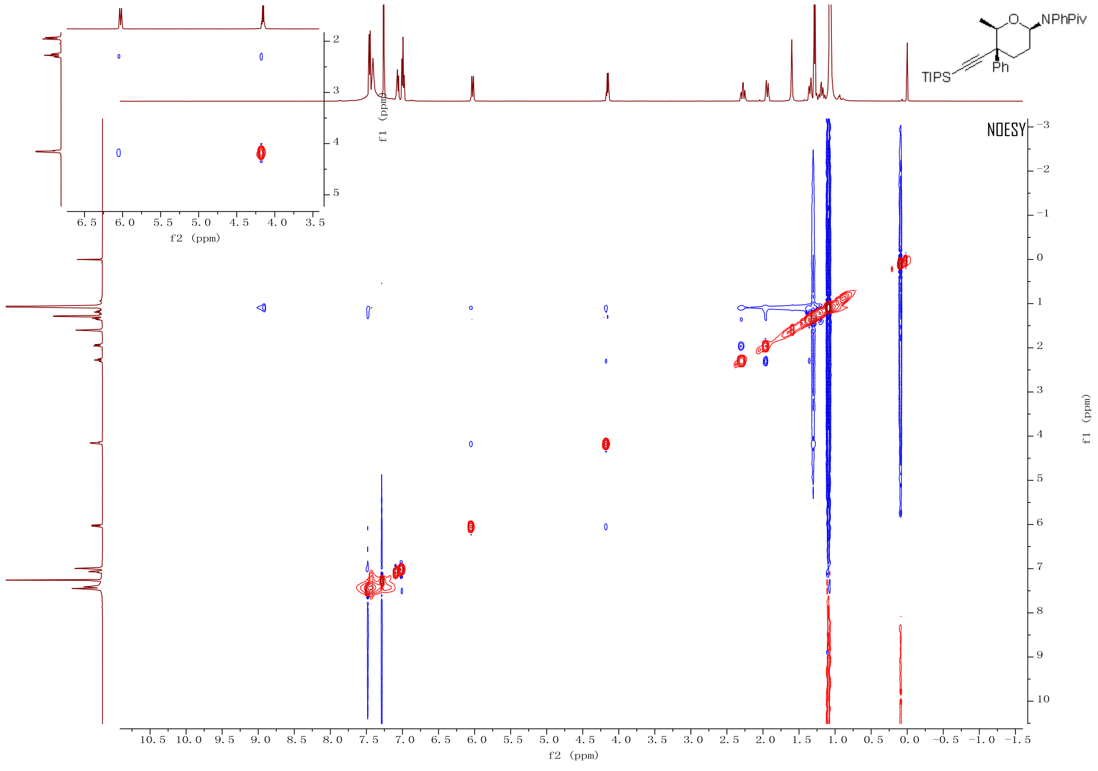

Supplementary Fig. 227 NOESY NMR spectrum of **13b**

Suppelmentary References

[1] W. Liu, T. Leischner, W. Li, K. Junge, M. Beller, *Angew. Chem. Int. Ed.* **2020**, *59*, 11321-11324.

[2] P. O. Miranda, M. A. Ramírez, V. S. Martín, J. I. Padrón, *Org. Lett.* **2006**, *8*, 1633-1636.

[3] X. Yang, F. D. Toste, *Chem. Sci.* **2016**, *7*, 2653-2656.

[4] L. García, J. Sendra, N. Miralles, E. Reyes, J. J. Carbó, J. L. Vicario, E. Fernández, *Chem. Eur. J.* **2018**, *24*, 14059-14063.

[5] T. Deng, W. Mazumdar, R. L. Ford, N. Jana, R. Izar, D. J. Wink, T. G. Driver, *J. Am. Chem. Soc.* **2020**, *142*, 4456-4463.

[6] F. Schömberg, Y. Zi, I. Vilotijevic, *Chem. Commun.* **2018**, *54*, 3266-3269.

[7] T. Furuta, H. Torigai, T. Osawa, M. Iwamura, *J. Chem. Soc., Perkin Trans. 1* **1993**, 3139-3142.
